# Nutrition-specific and nutrition-sensitive factors associated with mid-upper arm circumference as a measure of nutritional status in pregnant Ethiopian women: Implications for programming in the first 1000 days

**DOI:** 10.1371/journal.pone.0214358

**Published:** 2019-03-26

**Authors:** Shibani Ghosh, Kathryn Spielman, Meghan Kershaw, Kidane Ayele, Yitbarek Kidane, Krista Zillmer, Leslie Wentworth, Ashish Pokharel, Jeffrey K. Griffiths, Tefera Belachew, Eileen Kennedy

**Affiliations:** 1 Friedman School of Nutrition Science and Policy, Tufts University, Boston, United States of America; 2 Nevin Scrimshaw International Nutrition Foundation, Boston, United States of America; 3 School of Medicine, Tufts University, Boston, United States of America; 4 Jimma University, Jimma, Ethiopia; Boston University School of Public Health, UNITED STATES

## Abstract

Poor nutritional status in pregnancy expressed as low mid-upper arm circumference (MUAC) is associated with low birth weight. The study aims were to assess the nutritional status of pregnant Ethiopian women using MUAC and examine association with nutrition-specific and nutrition-sensitive factors, using baseline data of a prospective longitudinal observational birth cohort study conducted in three rural districts in the Oromia region of Ethiopia. Recruitment into the cohort was rolling over a period of nine months, and the data used for this analysis were collected while the women were between 12–32 weeks of gestation. Detailed household socio-demographics, agricultural production, women’s health, morbidity and diets, with weights, heights and MUAC, and anemia prevalence (HemoCue) were collected. The prevalence of low MUAC (< 23 cm) was 41%. Controlling for location and clustering, wealth quintile (OR = 0.88, CI = 0.82 to 0.96, p<0.01) was associated with decreased risk of low MUAC, while trimester (OR = 1.31, CI = 1.16 to 1.48, p<0.001) was associated with increased risk of low MUAC. The only significant factor amenable to nutrition-specific interventions was altitude-adjusted anemia, which was associated with increased risk of low MUAC (OR = 1.28, CI = 1.09 to 1.49, p<0.01). Significant factors amenable to nutrition-sensitive factors and associated with higher odds of low MUAC were household food insecurity (OR = 1.04, CI = 1.02 to 1.06, p<0.001), distance to the clinic in minutes (OR = 1.01, CI = 1.0 to 1.01, p<0.0001) and season of recruitment (lean versus non lean) (OR = 1.30, CI = 1.10 to 1.54, p<0.01). Literacy (OR = 0.85, CI = 0.74 to 0.98, p<0.05) and numeracy (OR = 0.75, CI = 0.62 to 0.91, p<0.01) were also significantly associated with lower odds of low MUAC. Poor nutritional status in pregnancy expressed as percent with low MUAC was high in Ethiopian women. It was associated with several nutrition-specific and -sensitive factors indicating the importance of multisectoral actions in improving outcomes within the first 1000 days.

## Introduction

Poor nutritional status in pregnancy as expressed as mid-upper arm circumference (MUAC) has been significantly associated with low birth weight in infants in both Asia and Africa [1–4]. This is particularly important since low birth weight is directly associated with higher rates of morbidity, mortality, stunting and poor cognitive development. In particular, MUAC in the mother has been shown to be correlated with gestational weight gain and birth weight [1, 5–9]. While literature points to the association of low MUAC with adverse birth outcomes, little is known about the factors that affect the MUAC in pregnancy. In Ethiopia, stunting in infants and young children under 24 months of age has been found to be associated with wealth, birth weight, maternal height and maternal BMI [10]. Poor diets, WASH (water, hygiene and sanitation) practices and access, and anemia are also significant contributors to maternal and child morbidity in Ethiopia [11–13].

Improving maternal nutritional status either through direct nutrition-specific actions or indirectly through nutrition-sensitive actions using and multisectoral approaches is considered crucial for the alleviation of malnutrition associated with poor linear growth [14, 15]. While timely nutrition-specific interventions can have a dramatic impact on reducing malnutrition, they are unlikely to achieve nutritional targets such as the Sustainable Development Goals [16] within the next decade. However, together with nutrition-sensitive actions, they have an enormous potential of enhancing the effectiveness of nutrition investments worldwide [4, 14, 15, 17]. A recent maternal and child health series paper by Ruel and Alderman defined nutrition specific interventions as approaches “that address immediate determinants of fetal and child nutrition and development–adequate food and nutrient intake, feeding, caregiving and parenting practices, and low burden of infectious diseases.” [15] Examples of nutrition-specific actions include improving maternal diets, improving access to health care during pregnancy, maternal micronutrient supplementation and disease prevention. Nutrition-sensitive interventions are approaches that target underlying determinants of fetal and child nutrition and development and include a focus on nutrition objectives. Examples of nutrition-sensitive approaches are improved access to nutritious foods (through agriculture), improved food security, access to education, access to improved water, hygiene and sanitation facilities. The United Nations' Decade of Action on Nutrition (2016–2025) [18] highlights both direct and nutrition-sensitive approaches as essential for alleviating malnutrition.

In order to improve nutritional status of vulnerable populations, Ethiopia is implementing interventions that target factors amenable to nutrition specific and sensitive actions. The National Nutrition Program of the Government of Ethiopia has included a menu of nutrition-specific actions to include access to adequate energy, protein, fats and micronutrients, access and availability of high quality care during prenatal, postnatal and delivery period, optimal weight again during pregnancy, prevention/treatment of anemia or any infections during pregnancy and achieving of optimal dietary diversity. Nutrition-sensitive actions include improving access to safe drinking water, hand-washing, appropriate sanitation practices, and access and availability to nutrient dense foods through diversified household production of crops and livestock [19–21], which in turn affect nutrition-specific actions such as reduced infections and optimal dietary diversity [4, 14, 15, 17].

The aims of this study were twofold: (1) to estimate the prevalence of low MUAC in pregnant women recruited in an observational birth cohort study in Oromia region of Ethiopia and (2) investigate factors amenable to nutrition-specific and -sensitive actions and ascertain the strength of their association with low MUAC in the study sample. Understanding which factors within these two areas of intervention would allow for better planning of multisectoral programs as well as better targeting and utilization of resources. Specifically this study examined nutrition-specific (e.g. use and access of health services, dietary diversity) and nutrition-sensitive (e.g. access and availability of nutrient dense foods as represented by the household’s crop and livestock production diversity as well as livestock ownership diversity, household food security, and access, use and practice of appropriate water/hygiene and sanitation practices) factors that would potentially explain the risk/odds of a low MUAC in a pregnant women in a select population in Ethiopia.

## Materials and methods

### Study design and data collection

The study population consists of pregnant women recruited as part of a longitudinal birth cohort study (implemented from 2014 to 2016) conducted in the three districts (called woredas) of Woliso, Goma and Tiro Afeta in the Oromia region of Ethiopia. The overall aim of the longitudinal study was to assess the effectiveness on maternal and child nutrition outcomes of an integrated nutrition/agriculture program (Empowering New Generations in Nutrition and Education—ENGINE) supported by the United States International Agency for Development (USAID). The study had an open cohort design and allowed rolling enrollment of pregnant women identified by health workers at the community level and at health posts from March 2014 to January 2015. The sample size was estimated to detect a 0.14 (1 standard-deviation) change in length-for-age z-score from birth to 12 months of age (in infants born to the recruited women), at 80% power at the 0.05 level of significance, allowing for 30% attrition. Length-for-age z-score was used as the measure for sample size computations as the programmatic intervention aimed to reduce the prevalence of stunting. Recruitment was conducted at the lowest administrative cluster level within the district (called a kebele). Using an intra-cluster correlation of 0.03, a total of 117 clusters (kebeles) with a total sample size of 4680 (n = 40 per cluster) was estimated. All kebeles within a district were sampled–with the exception of a few excluded due to inaccessibility–for a total of 1,560 pregnant women recruited in each of the three districts. The study received ethical approval from Jimma University in Ethiopia (approval reference number RPGC/264/2013) and from Tufts University in the United States (IRB reference number 11088). Informed consent was obtained for every woman recruited in this study for themselves and their unborn infant.

All data were collected in assessments administered at the household. Following receipt of informed consent, women were administered a urine test to confirm pregnancy, were tested for malaria using a Rapid Diagnostic tests (RDT) (Malascan) and also tested for anemia using HemoCue to determine inclusion. Exclusions and referrals were made for pregnant women who were found to be severely malnourished (MUAC < 18.5), with severe anemia (< 7 g/dl hemoglobin) or malaria, or with pregnancy induced hypertension. Multiple births were excluded.

The age range was 15 to 50 years old with gestation age between 12 to 32 weeks. A significant proportion of married women within these districts were below 18 years of age and it was considered necessary to include these women for routine follow ups. Both IRB committees provided approval for recruiting women under 18 years of age as, under Ethiopian law, married women (irrespective of age) are considered as emancipated. Data collection started in pregnancy and ended at 12 months postpartum. Data were collected in pregnancy, at the infant’s birth, and every 3 months until 12 months postpartum. Data on maternal and household characteristics were collected at every time point whereas infant data were collected at birth and across four time points until the end of the study. The data used in this analysis are from the pregnancy timepoint of this longitudinal study. These include pregnancy data, household characteristics, access to services, diet and food security. The methods for the overall study are further described in another publication by the same research group [22].

### Analytical approach

The data for this paper are limited to a cross-sectional round of the longitudinal study, i.e., the baseline/enrollment timepoint during pregnancy. A total of 4,560 women had complete data that are utilized in the analysis included in this paper. Explanatory variables identified through literature are described in Table 1 and include individual, household, and district-level agricultural and service related variables. The dependent variable is prevalence of low MUAC which is defined as the percentage of women with MUAC as less than 23 cm at the time of study enrollment [23].

**Table 1:** Descriptive characteristics.

**Descriptive Characteristic**	** **	**Mean/median+/- SD/IQR** **or % (Frequency)**
**Sample size**		4560
**Maternal Characteristics**		
Maternal height (cm)		157.4±5.9
Maternal weight (kg)		53.3±6.5
Prevalence of Low MUAC (< 23 cm)		40.64% (1853)
Age		26.41± 5.57
Literate		29.41% (1341)
Numerate		88.44% (4033)
Dietary Diversity Score		2.37 ± 0.75
Anemic		24.17% (1102)
Trimester	1^st^	3.55% (162)
	2nd	64.25% (2930)
	3rd	32.19% (1468)
Pregnant in previous 2 years		30.75% (1402)
Number of previous pregnancies		3.27 ± 2.52
Hand washing score (Women)		4.26± 1.27
**Household Characteristics**		
Household Food Insecurity Access Score		4.62± 5.14
Household size		4.82± 2.0
Religion	Muslim	67.32% (3070)
	Orthodox	22.57% (1029)
Type of Household (Female-headed)		1.43% (65)
Wealth Index (Quintiles)	1st	20.59% (939)
	2nd	19.71% (899)
	3rd	19.52% (890)
	4th	21.36% (974)
	5th	18.82% (858)
Access to protected water source		70.75% (3226)
**Agricultural Characteristics**		
Crop Production Diversity Score		2.95±1.61
Livestock Ownership Diversity Score		1.78 ± 1.6
**Season of recruitment**: Percent recruited in lean months (June-Sept)		42.13% (1921)
**Access and Use of Services**		
Market distance (minutes)^Ɨ^		30±45
Health clinic distance (minutes) ^Ɨ^		25±25
Received health worker home visits in past year		9.78%
Visited health facility in past year		68.16%

^Ɨ^ denotes median and IQR

While other factors within the domains of access and use of services and agricultural characteristics improved the model, with the exception of health clinic distance, none of the other variables increased nor decreased significantly the odds of a low MUAC.

Explanatory variables that were computed and tested against the dependent variable were classified into the domains of maternal characteristics (trimester, time of recruitment, age, literacy, numeracy, anemia, trimester, months since last pregnancy, number of prior pregnancies, minimum dietary diversity score, hand washing score), household characteristics (household food insecurity access score, household size, household religion, type of household- female headed, wealth quintile, access to protected water source), agricultural characteristics (crop production diversity score, livestock ownership diversity score,) and access and use of services (market distance, health clinic distance, health worker home visits, and number of visits to a health facility in the past year). These variables are amenable to both nutrition-specific and nutrition-sensitive interventions. Control variables included location and clustering at the kebele level.

### Variable generation and definitions

#### Season of recruitment

Women are more likely to have poor nutritional status (as reflected by the low MUAC) in the lean season [24]. Women were asked the number of months that their households were unable to meet food needs according to the questions for the Months of Adequate Food Provisioning (MAHFP) [25]. We found that across the period of 12 months, 40–80% of households were unable to meet food needs from June through September. The rest of the year the percentage of households not meeting food need ranged from 1–18%. Assuming these as the most likely months as lean for the households and subsequently to the pregnant woman, we created a binary variable to indicate recruitment/data collection in the lean months (June-September) compared to recruitment in the non-lean season (October-May).

#### Maternal characteristics

Either age or date of birth was reported by the woman at recruitment, and age was calculated (if necessary). Weight was estimated as an average of three measurements using a SECA measuring scale with a precision to the nearest 100g. Height was an average of three measurements using a Standiometer wooden measuring board, with a precision of 0.1 cm. MUAC was derived as an average of three measurements to the nearest millimeter, using a flexible non-elastic tape, midway between the tip of the shoulder and the tip of the elbow of the left arm with the arm hanging freely by the woman’s side. Literacy and numeracy variables were computed for each participant. Women were classified as literate if they were able to read specific sentences in Amharic and numerate if they correctly answered a simple math problem [26]. Prevalence of anemia (altitude-adjusted) and the mean hemoglobin levels were estimated. Hemoglobin levels were measured with the HemoCue system for mobile screening. Hemoglobin cutoff values were adjusted for altitude (GPS) and trimester according to method described by Cohen and Hass [27]. Adjusted hemoglobin cutoffs produced using this method range from 104.8 to 130.5 g/L. The average kebele altitude was imputed for missing altitude values. Women with hemoglobin levels below the adjusted cutoff point were classified as anemic. The LMP (last menstrual period) method was utilized to ascertain the gestational age/trimester of recruitment. Enumerators asked the women for the date of the last menstrual period, which was used to estimate the number of weeks pregnant. Trimester cutoffs were coded as 12 weeks and below for first, 13–27 weeks for second, and 28 and above for third trimester [28]. In order to capture birth spacing, women were asked how many months since their last pregnancy. Because recommended birth spacing is at least 24 months [29], we created a binary variable to capture women who gave birth within the previous 24 months. Women were asked the number of previous pregnancies, excluding the current pregnancy, they had and this number was used as a continuous measure in the model.

Women’s dietary diversity was estimated using the food group categories outlined by FAO for computing the Minimum Dietary Diversity Score for Women (MDD-W) [30]. The MDD-W is a proxy indicator for nutrient adequacy of the diet and was derived from a qualitative 24-hour dietary recall. While we used the method to determine food groups and a count of food groups, we did not compute the MDD-W preferring to utilize the score as a continuous variable. The multi-pass method was used for the 24-hour recall: first, all foods and drinks consumed during a 24-hour period were listed; next, amount consumed using portion size estimation with models; thirdly, more details on the food (color, type, size, brand) were gathered; and finally, all information was reviewed and verified. Dietary data was coded in Excel and grouped into the following categories: 1) all starchy staple foods, 2) beans and peas, 3) nuts and seeds, 4) dairy, 5) flesh foods, 6) eggs, 7) vitamin A-rich dark green leafy vegetables, 8) other vitamin-A-rich vegetables and fruits, 9) other vegetables, 10) other fruits. Due to very low dietary diversity, the continuous score was used instead of the minimum cut point of 5 out of 10 food groups [30]. A hand-washing score was calculated based on a count of seven responses to questions about important times for hand-washing (when dirt is visible, prior to eating, before food preparation, before serving a meal, before feeding a child, after using the toilet, after cleaning a child following defecation).

#### Household characteristics

Household food insecurity access scale (HFIAS) score was constructed using the method described by Coates et al 2007 [31]. The woman was asked whether a series of nine conditions related to food security occurred in the previous four weeks (yes or no), and if yes, how frequently the condition occurred (rarely, sometimes, or often). In order to create the HFIAS continuous score, the frequency-of-occurrence responses were coded to 0 for each condition that did not occur, and frequency-of-occurrence responses were summed [31]. Household size was estimated by the number of members of all ages recorded in the household roster as reported by the index woman. A wealth index was created using polychoric principal component analysis. Variables included in the wealth index estimation were main type of walls, roof, floor, toilet, cooking fuel, source of energy, drinking water source, other household water source, and water treatment method [32]. The wealth index was then divided into quintiles. In order to capture water quality, households reporting their most common source of water to be piped, public tap, tube well or borehole, protected well or spring, or rainwater were coded as using a protected water source; households reporting other water sources such as an unprotected well or spring, river, or pond were coded as using an unprotected water source [33].

#### Agricultural characteristics

Crop production diversity was calculated as a simple count of the crop groups produced annually by the household [19]. Crops were grouped as cereals, roots and tubers, legumes, cash crops, vegetables, fruits, oil seeds, and spices for a maximum score of 8. A livestock ownership diversity score was generated as a count of the types of livestock that households reported currently owning within the following categories: 1) cattle, 2) camel, 3) sheep, 4) goat, 5) donkey, 6) horse, 7) mule, 8) chicken, 9) other poultry, 10) bee 11) other livestock.

#### Use and access to services

Time in minutes to the nearest major and local markets were reported by the household head. The market distance variable was constructed by time in minutes to the nearest of the two (major or local) for each household. Access to health clinic is measured by the time in minutes to nearest health clinic/post. Healthcare service usage was measured by asking women if they had visited a health facility and/or if they had received a home visit by a health worker in the past year. Two separate binary variables were created, with women reporting having visited a health facility in the past year, and women reporting receiving a home visit by a health worker in the past year.

### Statistical analysis

Descriptive statistics were generated that allowed for finalization of the variables to be included in the final model. These included generating means and standard deviations and prevalence estimates and frequencies. All analyses were conducted using StataSE 14 software. Tests of normality were conducted using the Shapiro-Wilk test. All explanatory variables were tested for associations with the dependent variable (low MUAC) and for associations with each other. For bivariate comparisons of means, we used Student’s t-test, and for comparison of categorical variables, chi-square tests were used. Explanatory variables that were associated with low MUAC at the bivariate level but were non-significant, duplicative or collinear with other explanatory variables were discarded. Multivariate logistic regression analyses included woreda fixed effects to control for characteristics common by region and standard errors were clustered at the kebele level using the vce command in STATA. Model fit tests were conducted.

## Results

Prevalence of low MUAC in women in the three woredas was 40.6% with the mean MUAC± SD of 23.50 ± 2.16 (n = 4560). Descriptive statistics for the key explanatory variables/factors are presented in Table 1. The mean age (± SD) was 26.4 ± 5.57, 29.4% were literate and 88.4% were numerate. Just over 24% of women were anemic. Dietary diversity was low with a mean score of 2.40 ± 0.75 out of a possible score of 10.0. Most women were in their second (64.3%) or third trimester (32.2%). About 31% had been pregnant within the past two years and the mean number of previous pregnancies (irrespective of birth outcome) was 3.21 ± 2.53. The handwashing score was 4.26 ±1.3 (out of 7).

Examining the household characteristics, the mean household food insecurity access score was 4.62 ± 5.14 and household size was about 4.82 ± 2.00. The percentage of households by wealth index ranged from 20.6% in the 1^st^ (lowest) wealth quintile to 18.8% in the fifth (highest) wealth quintile and about 70% of households had access to protected water source. A total of 42% of women were recruited from the periods of June-September (the period with the most percentage of households reporting inability to meet food needs). Access and use of services was measured using market distance, health clinic distance and use of health services. Median distance to markets and health clinics were 30 (IQR = 45) and 25 (IQR = 25) minutes, respectively, while only 10% of women had received a health worker visit (home visit) though about 68% had visited a health facility in the past year.

### Factors associated with poor nutritional status in pregnancy

Odds ratios computed using multivariate logistic regressions are presented in Table 2 with standard errors and 95% confidence intervals. Adjusting for clustering and location, factors that increased the odds of a low MUAC and thus poor nutritional status include trimester of pregnancy, anemia, food insecurity, season of recruitment and distance to the health clinic. Factors that reduced the odds of a low MUAC were higher literacy, numeracy and wealth quintile.

**Table 2:** Factors associated with low MUAC (n = 4560).

Variable		Robust			
Maternal Characteristics		Odds Ratio	SE	95% CI	
Age		0.99	0.01	0.97	1.01
Literate		0.85*	0.06	0.74	0.98
Numerate		0.75**	0.07	0.62	0.91
Dietary Diversity Score		0.95	0.05	0.86	1.06
Anemic		1.28**	0.10	1.09	1.49
Trimester period		1.31***	0.08	1.16	1.48
Pregnant in previous 2 years		0.91	0.07	0.79	1.05
Number of previous pregnancies		0.99	0.02	0.94	1.04
Handwashing score (Women)		1.04	0.04	0.96	1.12
**Household Characteristics**					
Household Food Insecurity Access Score		1.04***	0.01	1.02	1.06
Household size		1.00	0.03	0.95	1.05
Religion (Reference: Other)	Muslim	1.54*	0.29	1.07	2.22
	Orthodox	1.26	0.17	0.97	1.64
Type of Household (Female-headed)		1.08	0.26	0.68	1.72
Wealth Index (quintile)		0.88***	0.03	0.82	0.95
Access to protected water source		1.03	0.08	0.88	1.20
**Agricultural Characteristics**					
Crop Production Diversity Score		0.97	0.03	0.91	1.03
Livestock Ownership Diversity Score (Ownership)		1.03	0.03	0.98	1.08
**Recruited in lean season**		1.30**	0.11	1.10	1.54
**Access and Use of Services**					
Market distance (minutes)		1.00	0.00	1.00	1.00
Health clinic distance (minutes)		1.01***	0.00	1.00	1.01
Received health worker home visits in past year		1.01	0.13	0.78	1.30
Visited health facility in past year		1.09	0.07	0.96	1.24
**Constant**		0.46*	0.18	0.22	0.97

* p<0.05

** p<0.01

***p<0.001

Model includes fixed effects for woreda and controls for clustering by kebele

## Discussion

The aims of this study were to assess the nutritional status of pregnant Ethiopian women using mid upper arm circumference as a measure of nutritional status and determine which nutrition specific versus nutrition sensitive factors were associated with the prevalence of low MUAC. Specifically in this study we examined factors that are targets of nutrition-specific interventions (pregnancy status, number of pregnancies to reflect parity, anemia, use and access of health services, dietary diversity) and nutrition-sensitive interventions (household food security, education, income and wealth, access and availability of nutrient dense foods as represented by the household’s crop production diversity and livestock ownership diversity, and access, use and practice of appropriate water/hygiene and sanitation practices) that could potentially explain the risk/odds of a low MUAC as a pregnant woman in a select population in Ethiopia.

Globally, there is recognition that direct nutrition interventions alone are unlikely to achieve nutrition targets such as the Sustainable Development Goals [16] within the next decade. Thus the United Nations' Decade of Action on Nutrition [18] emphasizes both direct and nutrition sensitive approaches for alleviating malnutrition. Ethiopia is one country implementing multi sector interventions to improve nutrition. In Ethiopia, despite vast improvements in stunting rates, going from 58% in 2000 to 40% in 2011 and 38% in 2016, thus rates still remain high [33, 34]. A significant proportion of this stunting is associated with low birth size in Ethiopian infants [10].

The NNP of the GOE has included a menu of nutrition specific interventions to include access to adequate micronutrients, access and availability of high-quality care during prenatal, postnatal and delivery period, optimal weight again during pregnancy, prevention/treatment of anemia or any infections during pregnancy and achieving of optimal dietary diversity. Nutrition sensitive elements revolve around access to safe drinking water, hand washing, appropriate sanitation practices, and access and availability to nutrient dense foods through diversified household production of crops and livestock [35].

Prevalence of low MUAC was high in the study population with over 40% of the pregnant women classified as having a low MUAC. We examined a combination of different nutrition-specific and -sensitive factors (i.e. factors that are amenable to nutrition-specific or sensitive interventions) and found that poor nutritional status in pregnancy expressed as low MUAC was associated with household food insecurity, increasing trimester of pregnancy, anemia, lean season of recruitment and distance to the health clinic. Factors associated with reduced odds of low MUAC were literacy, numeracy and wealth quintile. Thus, a combination of different nutrition-specific and nutrition-sensitive factors seemed to mediate nutritional status among this specific population.

Women who were anemic were also more likely to have a low MUAC. This relationship of MUAC and anemia is consistent with findings of another recent study in Ethiopia, which demonstrated that a MUAC measurement above 23 cm reduced odds of anemia by 0.41 [36]. That trimester period was associated with increased odds of a low MUAC is interesting as this implies that nutritional status worsens as pregnancy progresses. The nutritional burden of pregnancy may be taking a toll in late gestation, as women are not able to keep up with their growing needs. Our findings are supported by evidence of the lowering of MUAC by almost 0.4 cm through pregnancy into postpartum in a study of over 3000 women in Nepal [37]. In normal pregnancies, increases or decreases in MUAC are generally less than 0.05 cm, thus reflecting women’s pre-pregnancy or early pregnancy nutritional status as well as potentially an energy restricted environment [4, 29, 37]. Our findings reinforce the growing recognition that interventions to reach women before and during pregnancy are essential. Effective delivery systems, beyond the health system, need to be explored.

Our analysis controlled for religion in order to capture fasting differences across women who were Muslim, Orthodox, or other religions. Being Muslim in this sample was associated with higher odds of low MUAC compared to being neither Muslim nor Orthodox. It should be noted, however, that the majority (about 90%) of the sample is either Muslim or Orthodox and the reference category comprises only about 10% of the study sample. Furthermore, in a model with a binary variable to capture Muslim compared to non-Muslim (including Orthodox), Muslim was not significant.

While household food insecurity was associated with increased odds of low MUAC, dietary diversity (despite being low) was not. One explanation for the lack of association between low MUAC and diet diversity is that while this construct measures diet quality, it may not accurately reflect optimal energy intake [38]. Another explanation is the very low variability in dietary diversity across the population (Table 1).

In Ethiopia, distance from health clinic and posts has been identified as a key issue that impedes the use of services [39, 40]. Studies have found a significant association between distance to clinic, age and education, place of residence, ethnicity, parity and women’s autonomy. Our findings add to this literature indicating increased odds of poor nutritional status in pregnant women with increasing distance to the health clinic. While there is significant emphasis in Ethiopia to make health services accessible, our findings indicate that there is a need for alternative approaches for reaching pregnant and pre-pregnant women either at the household or community level.

Our findings linking household food insecurity to poor nutrition in pregnancy are in line with other studies which have shown associations between adverse diet and nutrition outcomes in both pregnant women and their offspring [41, 42]. Similarly, we found a positive association between household wealth and nutritional status in pregnancy, a finding that is supported by several different studies in Ethiopia and elsewhere in Africa and across the globe [1, 43, 44]. We also found a positive association between literacy and numeracy and nutritional status of our study subjects, findings supported by several other studies in Africa and elsewhere [45].

Recruitment in the lean season was associated with increased odds of low MUAC irrespective of the trimester of involvement. This is consistent with a large body of literature documenting that seasonality (lean season) negatively impacts nutritional status in rural women in Africa and elsewhere [46]. From a programmatic perspective the selective use of balanced protein/energy supplements could offset the nutritional insults that can occur during the lean season. Special attention would need to be devoted to identifying an effective delivery mechanism for reaching the most vulnerable during the lean season.

We did not find a relationship with other nutrition-sensitive variables such as water, hygiene and sanitation (WASH). A systematic review has shown an association between poor sanitation and inadequate water access and high maternal mortality[47] but we found no literature to support or contradict our findings. Reviewing the descriptive statistics, we found that there is a low variability of these factors within the population and/or that the wealth quintile serves as a proxy for the water, hygiene and sanitation variables. With respect to nutrition and production diversity linkages, while diversity of crop production and livestock ownership has been shown to be associated with dietary diversity in women in an analysis including Ethiopian data, this relationship was not significant [19]. Data from Ethiopia on children’s dietary diversity shows that the relationship is modulated by market access [48]. We found no studies examining the association of maternal nutritional status and production diversity (crop and livestock) and thus can only conclude that the relationship within our population is not significant.

Despite the finding that not all nutrition-sensitive variables were significant, our analysis is relevant given that maternal undernutrition contributes to fetal growth restriction and low birth weight (LBW), increasing the risk of neonatal mortality and long-term morbidities such as stunting. Low MUAC is indicative of a woman’s nutritional status in pre-pregnancy and pregnancy and also has a strong association with birth outcomes. A systematic review found that eight of 11 cross-sectional studies conducted across different countries in Asia and Africa on pregnant women examined the association between low MUAC and birth outcomes [49]. Six of these eight studies reported significantly higher risk of poor birth outcomes (infant LBW or preterm labor) in women with low versus normal MUAC, with risk ratios ranging from 1.6 to 6 across a wide range of MUAC cutoffs from 19 through 29 cm. In addition, low MUAC was significantly associated with maternal anemia and post-partum endometritis-myometritis. The same review found 12 longitudinal studies that focused on pregnant women and the association between low MUAC and birth outcomes, of which six studies found a significant association between low MUAC and infant LBW. While data are not shown, our ongoing analyses indicate that low maternal MUAC is significantly associated with low birth weight and length in this sample. Specifically in Ethiopia, an observational cohort study of 1,295 pregnant women in Kersa, Ethiopia found LBW incidence of 28%, with LBW being significantly associated with poverty, MUAC of less than 23 cm, not attending antenatal clinics, mother’s experience of physical violence during pregnancy and longer time to walk to a health facility [1].

From a programmatic perspective, our findings are relevant as they highlight the continuing importance of nutrition-specific interventions targeting anemia, energy and protein restriction in pregnancy, among others. They also highlight that nutrition-sensitive interventions such as those focusing on social safety nets, improving access to education, livelihoods and those targeting poverty reduction have potential in improving the nutritional status of women.

The results from this study indicate that attention needs to be given to women both during and pre-pregnancy and that a combination of both nutrition-specific or nutrition-sensitive factors are associated with poor nutritional status in Ethiopian women thereby justifying the call for multisectoral approaches in targeting malnutrition during the most crucial stages of the first 1000 days.

## Supporting information

S1 File GhoshEtAl_MUAC_2018-11-16.dta. Data in Stata format used for the analysis.
(DTA)

S2 File Questionnaires English.zip. English questionnaires used in the study for data collection.
(ZIP)

S3 File Questionnaires Afan-Oromo.zip. Questionnaires in Afan-Oromo, the language of questionnaire administration.
(ZIP)
