# Supplementary material for: Nutrition-specific and nutrition-sensitive factors associated with mid-upper arm circumference as a measure of nutritional status in pregnant Ethiopian women: Implications for programming in the first 1000 days
Source: PLoS One. 2019 Mar 26;14(3):e0214358. doi: 10.1371/journal.pone.0214358 (PMC6435172; doi:10.1371/journal.pone.0214358)
Supplement: S2 File — English questionnaires used in the study for data collection. (ZIP) [file pone.0214358.s002.zip › USAID GC╠oo╠é ENGINE HH HEAD_V3_Cleaned _4.23.14.docx]

USAID – ENGINE Birth Cohort Study Household Questionnaires

## Household Head Interview

## Table of Contents

Household Head Interview 1

Table of Contents 1

Module 1: Household Information& Characteristics 3

Section 1: Interview Information 3

Section 2: Literacy/Numeracy for Household Head 3

Section 3: Water & Sanitation 3

Module 8: Household Food Security 4

Section 1: Months of Adequate Household Food Provisioning 4

Section 2: Household Food Insecurity Access Scale 4

Module 9: Gender and Decision Making 6

Section 1: Access, ownership, and control of agricultural production 6

Section 2: Access, ownership, and control of durable goods 7

Section 3: Gender and Time Allocation 8

Module 10: Social Participation and Access to Information 9

Section 4: Program Exposure &Uptake: Agricultural Production Activities- HH Head 9

Section 5: Access and Satisfaction with Extension Services 13

Section 6: Access to Infrastructure 14

Module 11 – Agricultural Production 14

Section 1: Land Ownership and Use 14

Section 2: Crop Production 16

Section 3: Crop Production Expenses 19

Section 4: Estimation of Livestock Ownership 21

Section 5: Livestock production 22

Section 6: Livestock Expenses 24

Section 7: Crop and Livestock Product Markets 26

Section 8: Agricultural Technologies and Management Practices 26

Section 9: Farm Labour for All Farm Activities 27

Module 12 – Income & Expenditure 29

Section 1: Other Household Income Sources 29

Section 2: Savings & Banking 31

Section 3: Other Expenditure 32

#

# Module 1: Household Information& Characteristics

## Section 1: Interview Information

**Table 1.1**

| **Number** | **Question** | **Response** | **Variable name** |
| --- | --- | --- | --- |
|  | Date of interview (dd/mm/yyyy) | // | HDATEINTA |
|  | Data Time Point (1,7,9) |  | HDTIMEPTA |
|  | Household ID |  | HHIDA |
|  | Woreda | *(drop list: Woliso, Goma, TiroAfeta)* | HWOREDAA |
|  | Kebele | *(text)* | HKEBELEA |
|  | Gote / Gere | *(text)* | HGOTEA |
|  | GPS | “Get GPS Coordinates” (button) | HHGPSA |
|  | Name of Household Head | (First, Fathers, Grandfathers) | HHDNAMA |
|  | Is Index Woman HH Head? | 1=yes 0=no | HHDINDWA |
|  | Interviewer’s ID 1 |  | HID1A |
|  | Interviewer’s ID 2 |  | HID2A |
|  | Supervisor’s ID |  | HSIDA |
|  | Outcome of Interview | 1. completed 2. incomplete  3. absent 4. refused  5. could not locate | HINTOUTA |

## Section 2: Literacy/Numeracy for Household Head

|  | Can the HH Head write their name in local language? (Please test) | 1=yes, 0=no, 98 = not tested | HIMWRITE |
| --- | --- | --- | --- |
| 1.2.2 | Can the HH head read the following sentences?  (picture of local text) | 1=yes, 0=no, 98 = not tested | HIMREAD |
|  | Can the HH head correctly answer the following numeracy test?  "If you sell eggs for 30 Birr and chicks for 50 Birr, how many Birr do you have?" | 1=yes, 0=no, 98=not tested | HIMNUM |

## Section 3: Water & Sanitation

| Under what circumstances do you wash your hands? (Do not read the responses below. Allow respondent to answer, then fill each item below.) | | | |  |
| --- | --- | --- | --- | --- |
| 1.3.1 | Not at all | 1=Yes  0=No 98 =DK | WMNIL | |
| 1.3.2 | When dirt is visible | 1=Yes  0=No 98 =DK | WMDIRT | |
| 1.3.3 | After toilet use/defecation/urination | 1=Yes  0=No 98 =DK | WMTOILETUSE | |
| 1.3.4 | After cleaning child following defecation | 1=Yes  0=No 98 =DK | WMCLEANCHILD | |
| 1.3.5 | Before preparing the food | 1=Yes  0=No 98 =DK | WMFOOD | |
| 1.3.6 | Before serving a meal | 1=Yes  0=No 98 =DK | WMMEAL | |
| 1.3.7 | Before eating | 1=Yes  0=No 98 =DK | WMEAT | |
| 1.3.8 | Before feeding a child | 1=Yes  0=No 98 =DK | WMFEEDBABY | |
| 1.3.9 | When I am reminded to do so | 1=Yes  0=No 98 =DK | WMREMIND | |

# Module 8: Household Food Security

## Section 1: Months of Adequate Household Food Provisioning

Now I would like to ask you about your household’s food supply during different months of the year. When responding to these questions, please think back over the last 12 months, starting with the current month until the same time last year.

**Table 8.1: Period of adequate household food provision**

|  | **Question** | **Responses** | **Var. name** |
| --- | --- | --- | --- |
|  | Were there month(s), in the past 12 months, in which you did not have enough food to meet your family’s needs? | 1=Yes  0=No 98=Don’t Know | SGOODM |
| If yes, which were the months in the past 12 months during which you did not have enough food to meet your family’s needs? | | | |

*To the DC: This includes any kind of food from any source, such as own production, purchase or exchange, food aid, or borrowing. Do not read the list of months aloud. Use a seasonal calendar if needed to help respondent remember the different months. Probe to make sure the respondent has thought about the entire past 12 months.*

|  | **Month** | **Response** |  |
| --- | --- | --- | --- |
|  | January | 1=Yes  0=No 98=Don’t Know | SJANM |
|  | February | 1=Yes  0=No 98=Don’t Know | SFEBM |
|  | March | 1=Yes  0=No 98=Don’t Know | SMARM |
|  | April | 1=Yes  0=No 98=Don’t Know | SAPRM |
|  | May | 1=Yes  0=No 98=Don’t Know | SMAYM |
|  | June | 1=Yes  0=No 98=Don’t Know | SJUNM |
|  | July | 1=Yes  0=No 98=Don’t Know | SJULM |
|  | August | 1=Yes  0=No 98=Don’t Know | SAUGM |
|  | September | 1=Yes  0=No 98=Don’t Know | SEPTM |
|  | October | 1=Yes  0=No 98=Don’t Know | SOCTM |
|  | November | 1=Yes  0=No 98=Don’t Know | SNOVM |
|  | December | 1=Yes  0=No 98=Don’t Know | SDECM |

## Section 2: Household Food Insecurity Access Scale

I am going to ask you questions about your household’s food supply over the past four weeks. Food supply includes staples, sauces, and any other foods in your diet and the diets of all members of your household.

**Table 8.2: Household Food Insecurity Access Scale**

|  | **Question** | **Response** | **Var name** |  |
| --- | --- | --- | --- | --- |
| 8.2.1 | In the past four weeks, did you worry that your household would not have enough food? | 1=Yes  0=No  98=Don’t Know | SWORRYM | |
| 8.2.2 | If yes, how often did this happen? | 1 = Rarely (once or twice in the past four weeks)  2 = Sometimes (3 to 10 times in the past four weeks)  3 = Often (more than 10 times in the past four weeks) | SWORRYFRQM | |
| 8.2.3 | In the past four weeks, were you or any household member not able to eat the kinds of foods you preferred because of a lack of resources? | 1=Yes  0=No  98=Don’t Know | SKINDM | |
| 8.2.4 | If yes, how often did this happen? | 1 = Rarely (once or twice in the past four weeks)  2 = Sometimes (3 to 10 times in the past four weeks)  3 = Often (more than 10 times in the past four weeks) | SKINDFRQM | |
| 8.2.5 | In the past four weeks, did you or any household member have to eat a limited variety of foods due to a lack of resources? | 1=Yes  0=No  98=Don’t Know | SLIMITEDM | |
| 8.2.6 | If yes, how often did this happen? | 1 = Rarely (once or twice in the past four weeks)  2 = Sometimes (3 to 10 times in the past four weeks)  3 = Often (more than 10 times in the past four weeks) | SLIMITEDFRQM | |
| 8.2.7 | In the past four weeks, did you or any household member have to eat some foods that you really did not want to eat because of a lack of resources to obtain other types of food? | 1=Yes  0=No  98=Don’t Know | SDISLIKEM | |
| 8.2.8 | If yes, how often did this happen? | 1 = Rarely (once or twice in the past four weeks)  2 = Sometimes (3 to 10 times in the past four weeks)  3 = Often (more than 10 times in the past four weeks) | SDISLIKEFRQM | |
| 8.2.9 | In the past four weeks, did you or any household member have to eat a smaller meal than you felt you needed because there was not enough food? | 1=Yes  0=No  98=Don’t Know | SMALLM | |
| 8.2.10 | If yes, how often did this happen? | 1 = Rarely (once or twice in the past four weeks)  2 = Sometimes (3 to 10 times in the past four weeks)  3 = Often (more than 10 times in the past four weeks) | SMALLFRQM | |
| 8.2.11 | In the past four weeks, did you or any household member have to eat fewer meals in a day because there was not enough food? | 1=Yes  0=No  98=Don’t Know | SFEWM | |
| 8.2.12 | If yes, how often did this happen? | 1 = Rarely (once or twice in the past four weeks)  2 = Sometimes (3 to 10 times in the past four weeks)  3 = Often (more than 10 times in the past four weeks) | SFEWFRQM | |
| 8.2.13 | In the past four weeks, was there ever no food to eat of any kind in your household because of lack of resources to get food? | 1=Yes  0=No  98=Don’t Know | SNOFOODM | |
| 8.2.14 | If yes, how often did this happen? | 1 = Rarely (once or twice in the past four weeks)  2 = Sometimes (3 to 10 times in the past four weeks)  3 = Often (more than 10 times in the past four weeks) | SNOFOODFRQM | |
| 8.2.15 | In the past four weeks, did you or any household member go to sleep at night hungry because there was not enough food? | 1=Yes  0=No  98=Don’t Know | SLEEPM | |
| 8.2.16 | If yes, how often did this happen? | 1 = Rarely (once or twice in the past four weeks)  2 = Sometimes (3 to 10 times in the past four weeks)  3 = Often (more than 10 times in the past four weeks) | SLEEPFRQM | |
| 8.2.17 | In the past four weeks, did you or any household member go a whole day and night without eating anything because there was not enough food? | 1=Yes  0=No  98=Don’t Know | SNODAYM | |
| 8.2.18 | If yes, how often did this happen? | 1 = Rarely (once or twice in the past four weeks)  2 = Sometimes (3 to 10 times in the past four weeks)  3 = Often (more than 10 times in the past four weeks) | SNODAYFRQM | |

# Module 9: Gender and Decision Making

## Section 1: Access, ownership, and control of agricultural production

Now I will ask you about some items that your household may have. First, I would like to know if your household has these items, currently or in the past season (the past 6 months). Then I would like to know who in your household works with or uses these items, who owns them, and who makes decisions about them.

Table 9.1

| **Question number** | **Item** | **Available** (if no, skip columns to the right) | **Who Owns** | **Control (Decision to purchase/use/sell)** | **Decision on use of income** |
| --- | --- | --- | --- | --- | --- |
|  |  | 1. Yes 0. No 98 = don’t know | *Coded Below** | *Coded Below** | *Coded Below** |
| 9.1.1. | Agricultural Land | GLANDA | GLANDO | GLANDC | GLANDD |
| 9.1.2. | Agricultural Land use |  |  | GLANDUSEC |  |
| 9.1.3. | Land for Home Garden | GGARDA | GGARDO | GGARDC | GGARDD |
| 9.1.4. | Land for Non Agricultural Purpose (commercial, residential) | GNALANDA | GNALANDO | GNALANDC | GNALANDD |
| 9.1.5. | Cereals | GCEREALA | GCEREALO | GCEREALC | GCEREALD |
| 9.1.6. | Fruit Trees | GBANANA | GBANANAO | GBANANAC | GBANANAD |
| 9.1.7. | Roots, tubers | GROOTSA | GROOTSO | GROOTSC | GROOTSD |
| 9.1.8 | Cash crops (coffee, chat, etc.) | GCASHCA | GCASHCO | GCASHCC | GCASHCD |
| 9.1.9. | Vegetables | GVEGA | GVEGO | GVEGC | GVEGD |
| 9.1.10. | Pulses | GPULSA | GPULSO | GPULSC | GPULSD |
| 9.1.11 | Oil Seeds | GOILSA | GOILSO | GOILSC | GOILSD |
| 9.1.12 | Spices/Herbs | GSPICA | GSPICO | GSPICC | GSPICD |
| 9.1.13 | Cattle | GCATTLEA | GCATTLEO | GCATTLEC | GCATTLED |
| 9.1.14 | Horse/mule/donkey/camels | GMULEA | GMULEO | GMULEC | GMULED |
| 9.1.15 | Sheep/goats | GRUMINA | GRUMINO | GRUMINC | GRUMIND |
| 9.1.16 | Poultry | GPOULTRYA | GPOULTRYO | GPOULTRYC | GPOULTRYD |
| 9.1.17 | Beehives | GBEEHA | GBEEHO | GBEEHC | GBEEHD |
| 9.1.18 | House and other structures | GHOUSEA | GHOUSEO | GHOUSEC | GHOUSED |

***Code: 1. Head of Household 2. HH Father 3.HH First Wife 4.HH Mother 5.HH Second wife 6.Other 7.All 8. N/A**

***(Select as many as apply)***

## Section 2: Access, ownership, and control of durable goods

Now I will ask you about some other household items. Please let me know if these items are currently available to your household and, if so, who uses them, owns them, and has control over their usage.

|  |  | **Available** | **Quantity**  **Available** | **Quantity**  **Functional** | **Ownership**  **(Non-Functional** | **Ownership**  **(Functional)** | **Access**  **(Functional)** | **Control of usage (Functional)** |
| --- | --- | --- | --- | --- | --- | --- | --- | --- |
|  | **Good** | (1=yes,0=no, 98 = DK) if no, skip columns to right | *(Numeric)* | *(Numeric)* | *Coded Below** | *Coded Below** | *Coded Below** | *Coded Below** |
| 9.2.1 | Radio | GRADIOA | GRADIOQ | GRADIOQF | GRADIONF | GRADIOO | GRADIOS | GRADIOC |
| 9.2.2 | TV | GTVA | GTVQ | GTVQF | GTVNF | GTVO | GTVS | GTVC |
| 9.2.3 | Telephone – fixed line | GTELEA | GTELEQ | GTELEQF | GTELENF | GTELEO | GTELES | GTELEC |
| 9.2.4 | Mobile phone | GMOBILA | GMOBILQ | GMOBILQF | GMOBILNF | GMOBILO | GMOBILS | GMOBILC |
| 9.2.5 | Bicycle | GBICA | GBICQ | GBICQF | GBICNF | GBICO | GBICS | GBICC |
| 9.2.6 | Motorcycle | GMOTOA | GMOTOQ | GMOTOQF | GMOTONF | GMOTOO | GMOTOAS | GMOTOC |
| 9.2.7 | Three wheeler (Bajaj) | GBAJAJAA | GBAJAJQ | GBAJAJQF | GBAJAJNF | GBAJAJO | GBAJAJS | GBAJAJC |
| 9.2.8 | Cart | GCARTA | GCARTQ | GCARTQF | GCARTNF | GCARTO | GCARTS | GCARTC |

***Code: 1. Head of Household 2. HH Father 3.HH First Wife 4.HH Mother 5.HH Second wife 6.Other 7.All 8. N/A**

***(Select as many as apply)***

## Section 3: Gender and Time Allocation

I am going to ask how you used your time yesterday.

How did you spend your time yesterday, from the time you woke up to the time you went to sleep?

| ***Select one option:***  **1.       Crop Agric work**  **2.       Livestock Agric Work**  **3. Non Agric work (Own)**  **4. Non Agric work (Paid)**  **5. Travel, commuting**  **6. Shopping/Services (Inc. Health)**  **7. Education/Training**  **8.       Household (domestic) and work with children/elderly/sick**  **9.       Personal time (Rest, leisure, social, religious, eating etc.)** | |
| --- | --- |
| **Time** | **Main man of the HH** |
| 6:00 (midnight) | GTIMEM00 |
| 7:00 | GTIMEM01 |
| 8:00 | GTIMEM02 |
| 9:00 | GTIMEM03 |
| 10:00 | GTIMEM04 |
| 11:00 | GTIMEM05 |
| 12:00 (morning) | GTIMEM06 |
| 1:00 | GTIMEM07 |
| 2:00 | GTIMEM08 |
| 3:00 | GTIMEM09 |
| 4:00 | GTIMEM10 |
| 5:00 | GTIMEM11 |
| 6:00 | GTIMEM12 |
| 7:00 (afternoon) | GTIMEM13 |
| 8:00 | GTIMEM14 |
| 9:00 | GTIMEM15 |
| 10:00 | GTIMEM16 |
| 11:00 | GTIMEM17 |
| 12:00 (evening) | GTIMEM18 |
| 1:00 | GTIMEM19 |
| 2:00 | GTIMEM20 |
| 3:00 | GTIMEM21 |
| 4:00 | GTIMEM22 |
| 5:00 | GTIMEM23 |

# Module 10: Social Participation and Access to Information

Section 1: Social Participation
 Do you or any member of your household currently participate in any of the following social groups?

**Table 10.1**

|  | **Group** | Main man (1=Yes 0=No) 98=Don’t Know | Other members (1=Yes 0=No) 98=Don’t Know |
| --- | --- | --- | --- |
| 10.1.1. | Rural savings and credit cooperative | GRSCCRMM | GRSCCRMOM |
| 10.1.2. | Mother Support group | GWOMM | GWOMOM |
| 10.1.3. | A religious group | GRELIGM | GRELIGOM |
| 10.1.4. | A youth group | GYOUTHM | GYOUTHOM |
| 10.1.5. | Producer/Farmer association | GHPRODASSMM | GHPRODASSOM |
| 10.1.6. | Kebele Committee(Administration, Health, Other) | GKEBELEM | GKEBELEOM |
| 10.1.7. | Village Saving and Loans (Edir, Ekub, small scale financial group etc-Village Saving and Loans) | GVSLAM | GVSLAM |
| 10.1.8 | Other group (specify___________) GOTHSPE | GOTHM | GOTHOM |

## Section 4: Program Exposure &Uptake: Agricultural Production Activities- HH Head

Table 10.4

| A. Did you or anyone in your household participate in the following activity in the past two years?  0=No  1=Yes  98=Don’t  Know  If “Yes” or “Don’t  Know” skip to C | | B. If No, why?  (Select all that apply)  Activity not available=0  Insufficient time=1  Too far=2  Too expensive=3  No interest=4  Insufficient benefit=5  Other=6  Don’t know=98 | C. Source of service/information/training?(Select all that apply   1. Development Agent 2. Agricultural Officer 3. Veterinary officer 4. Community Conversations 5. Social/religious group 6. NGO 7. Radio 8. TV 9. Newspaper 10. Mobile phone 11. Poster/flyers/leaflets 12. Friend/Relative 13. Other 14. Don’t know | D. How often did you or someone in your household participate or receive [ACTIVITY/ITEM] in the past 2 years? | | | E. Did you change any behaviors or use inputs and/or advice provided? (specific questions by section)  0=No  1=Yes  98=Don’t  Know | F. If No, why?  *(Select all that apply)*  Insufficient time=1  Too expensive=2  No interest=3  Insufficient benefit=4  Already practicing behavior = 5  Other=6  Don’t know=98 | |
| --- | --- | --- | --- | --- | --- | --- | --- | --- | --- |
|  |  |  |  | Number  (98 if Don’t Know) | Code:  1=Week  2=Month  3=In the past 6 months  4= In the past year  5=In the past 2 years | |  |  |  |
| Have you heard of the Ethiopian Agricultural Growth program? If yes, go to C. |  |  |  |  | |  |  | |  |
| If yes, is your household a direct beneficiary of AGP? |  |  |  |  | |  |  | |  |
| Did you or your household receive any information/trainings on agriculture and agricultural innovations? |  |  |  |  | |  |  | |  |
| Did you receive information/training on the new seed varieties: |  |  |  |  | |  | Did you use the new variety based on the information? | |  |
| *Teff* |  |  |  |  | |  |  | |  |
| *Barley* |  |  |  |  | |  |  | |  |
| *Maize* |  |  |  |  | |  |  | |  |
| *Sorghum* |  |  |  |  | |  |  | |  |
| *Beans or peas* |  |  |  |  | |  |  | |  |
| *Onions* |  |  |  |  | |  |  | |  |
| *Tomatoes* |  |  |  |  | |  |  | |  |
| *Carrots* |  |  |  |  | |  |  | |  |
| *Cabbage* |  |  |  |  | |  |  | |  |
| *Other (please specify)* |  |  |  |  | |  |  | |  |
| Did you receive information/training on: |  |  |  |  | |  |  | |  |
| *Production of honey (Apiary management/apiculture* |  |  |  |  | |  |  | |  |
| *Small ruminant fattening (sheep/goats)* |  |  |  |  | |  |  | |  |
| *Cattle fattening* |  |  |  |  | |  |  | |  |
| *Livestock management (veterinary practices, feed management* |  |  |  |  | |  |  | |  |
| *Production of livestock products (ghee, milk, butter, yogurt)* |  |  |  |  | |  |  | |  |
| *Water management and irrigation* |  |  |  |  | |  |  | |  |
| *Homestead gardens* |  |  |  |  | |  |  | |  |
| *Integrated Pest management* |  |  |  |  | |  |  | |  |
| *Soil conversation measures* |  |  |  |  | |  |  | |  |
| *- Practicing FanyaJuu (embarkment along the contour which is made of soil or stones)* |  |  |  |  | |  |  | |  |
| *- Stone bunds* |  |  |  |  | |  |  | |  |
| *- soil bunds* |  |  |  |  | |  |  | |  |
| *Soil fertility management* |  |  |  |  | |  |  | |  |
| *Soil erosion* |  |  |  |  | |  |  | |  |
| *Use of chemical fertilizers* |  |  |  |  | |  |  | |  |
| *Row planting* |  |  |  |  | |  |  | |  |
| *On Market/collection center set up* |  |  |  |  | |  |  | |  |
| *On marketing strategies* |  |  |  |  | |  |  | |  |
| *Price monitoring* |  |  |  |  | |  |  | |  |
| *On set up of cooperatives/farmer associations/VSLA* |  |  |  |  | |  |  | |  |
| Have you participated/observed/visited in any of the following: |  |  |  |  | |  |  | |  |
| *Farmer Training sessions* |  |  |  |  | |  |  | |  |
| *Farmer demonstration plots* |  |  |  |  | |  |  | |  |
| Have you received new seed varieties for agriculture? |  |  |  |  | |  | Have you used the varieties or are you planning to use the varieties? | |  |
| *Teff* |  |  |  |  | |  |  | |  |
| *Barley* |  |  |  |  | |  |  | |  |
| *Maize* |  |  |  |  | |  |  | |  |
| *Sorghum* |  |  |  |  | |  |  | |  |
| *Beans or peas* |  |  |  |  | |  |  | |  |
| *Onions* |  |  |  |  | |  |  | |  |
| *Tomatoes* |  |  |  |  | |  |  | |  |
| *Carrots* |  |  |  |  | |  |  | |  |
| *Cabbage* |  |  |  |  | |  |  | |  |
| *Other (please specify)* |  |  |  |  | |  |  | |  |
| *Have you received any of the following:* |  |  |  |  | |  |  | |  |
| *Chemical fertilizers* |  |  |  |  | |  |  | |  |
| *Chickens* |  |  |  |  | |  |  | |  |
| *Goats* |  |  |  |  | |  |  | |  |
| *Cattle* |  |  |  |  | |  |  | |  |
| *Other livestock (specify)* |  |  |  |  | |  |  | |  |
| *Other inputs (specify)* |  |  |  |  | |  |  | |  |
| *Irrigation inputs* |  |  |  |  | |  |  | |  |
| *Apiculture/Apiary inputs* |  |  |  |  | |  |  | |  |
| *Micro-credit loans for agriculture* |  |  |  |  | |  |  | |  |
| *Micro-credit loans for other purposes* |  |  |  |  | |  |  | |  |
| *Have you or your community received support/facilitation for the following:* |  |  |  |  | |  |  | |  |
| *Market set up* |  |  |  |  | |  |  | |  |
| *Marketing strategies* |  |  |  |  | |  |  | |  |
| *Price monitoring* |  |  |  |  | |  |  | |  |
| *Set up Associations (VSLA, farmer)* |  |  |  |  | |  |  | |  |
| *Set up micro credit enterprises* |  |  |  |  | |  |  | |  |

## Section 5: Access and Satisfaction with Extension Services

**Read aloud:** Now I would like to ask you about your access to agricultural/livestock development agent

| **Number** | **Question** | **Response** | **Variable Name** |
| --- | --- | --- | --- |
| 10.5.1 | Have you (yourself) met with any agriculture development agent in the past 12 months? | Yes = 1 | *HHLOSDA* |
|  |  | No = 2 |  |
| 10.5.2 | How many times have you met with any agricultural development agent in the past 12 months? | Number of visits | *HHLOSDAV* |
|  |  |  |  |
| 10.5.3 | Are you satisfied with the visits? | Yes.............1 | *HHLOSDAS* |
|  |  | **No..............0** |  |
| 10.5.4 | If no, why not? | 1.        Lack of time | *HHLOSDASR* |
|  |  | 2.        Insufficient information |  |
|  |  | 3.        Insufficient number of visits |  |
|  |  | 98.      Don’t know |  |
| 10.5.5 | What was the gender of the agriculture development agent whom you last met | Male = 1 | *HHLOSDAG* |
|  |  | Female = 2 |  |
|  |  | Both =3 |  |
| 10.5.6 | Have you (yourself) met with any livestock development agent in the past 12 months? | Yes = 1 | *HHLOSLDA* |
|  |  | No = 2 |  |
| 10.5.7 | How many times have you met with any livestock development agent in the past 12 months? | Number of visits | *HHLOSLDAV* |
|  |  |  |  |
| 10.5.8 | Are you satisfied with the visits? | Yes.............1 | *HHLOSLDAS* |
|  |  | **No..............0** |  |
| 10.5.9 | If no, why not? | 1.        Lack of time | *HHLOSLDASS* |
|  |  | 2.        Insufficient information |  |
|  |  | 3.        Insufficient number of visits |  |
|  |  | 98.      Don’t know |  |
| 10.5.10 | What was the gender of the livestock development agent whom you last met | Male = 1 | *HHLOSLDAG* |
|  |  | Female = 2 |  |

## Section 6: Access to Infrastructure

| How long does it take for you to travel from your home to the following locations, on foot, in a single **ONE WAY** trip? | |
| --- | --- |
|  | **Time Taken [Hr: Minute]** |
| Nearest primary School |  |
| Nearest health clinic/post |  |
| Nearest veterinary clinic |  |
| Nearest major road |  |
| Nearest local road |  |
| Nearest local market |  |
| Nearest major market |  |
| Nearest DA office |  |
| Nearest Farmer training center |  |
| Nearest woreda town |  |
| Nearest commercial bank |  |
| Nearest microfinance institution |  |

# Module 11 – Agricultural Production

## Section 1: Land Ownership and Use

First ask the respondent to describe the household’s land use from January – June 2013 and enter responses in the table below. Then ask the respondent to describe the household’s land use for the period July to December 2013, and enter the responses in the table.

|  |  | **January – June 2013**  **Value Unit *** | | **July – December 2013**  **Value Unit *** | |
| --- | --- | --- | --- | --- | --- |
| 11.1.1. | Own Land Cultivated | LANDOWN6 | LANDOWN6U | LANDOWN12 | LANDOWN12U |
| 11.1.2. | Land Rented or Borrowed Cultivated | LANDRENTIN6 | LANDRENTIN6U | LANDRENTIN12 | LANDRENTIN12U |
| 11.1.3. | Shared Land Cultivated | LANDRENTOUT6 | LANDRENTOUT6U | LANDRENTOUT12 | LANDRENTOUT12U |
| 11.1.4. | Total Cultivated Land | *LANDSHARE6* | *LANDSHARE6U* | *LANDSHARE12* | *LANDSHARE12U* |
| 11.1.5. | Fallowed Land Owned | LANDARAB6 | LANDARAB6U | LANDARAB12 | LANDARAB12U |
| 11.1.6 | Land Rented Out | LANDCULT6 | LANDCULT6U | LANDCULT12 | LANDCULT12U |
| 11.1.7 | Homestead Garden Land | LANDGARD6 | LANDGARD6U | LANDGARD12 | LANDGARD12U |
| 11.1.8 | Total Land Owned | *LANDTOT6* | *LANDTOT6U* | *LANDTOT12* | *LANDTOT12U* |

*Unit Choices include: Hectares, Sq. Meters, Timad, Fechasa, Senga, Other(specify)

## Section 2: Crop Production

To the RA: First, identify which crops the household grew in the year from July 2012 to June 2013. Then, ask the respondent to think about crop production from July to December 2013 (last season) and fill in the table below. Then ask the respondent to think about the January to June 2013 period, and fill in the table again for that time period.

**Table 11.2: Crop production**

| **Group** | **Question number** | **Crop** | **Cultivated in past 12 mo’s? (1=yes 0=no)** | **Season** | **Area (Value)** | **Area (unit)** | **Intercropped (1=yes0=no)** | **If yes, main 2nd crop?** | **HARVEST** | | | | |  | **DISPOSITION** | | | | | |  | |
| --- | --- | --- | --- | --- | --- | --- | --- | --- | --- | --- | --- | --- | --- | --- | --- | --- | --- | --- | --- | --- | --- | --- |
|  |  |  |  |  |  |  |  |  | **Percentage of Crop not harvested**  **%** | **Number of units harvested** | **What size sack used?**  ***Choose 1:***  **1.100kg**  **2.50kg**  **3.25kg**  **4. Piece**  **5. Zorba**  **6. Other** | **Weight of each unit (kg)** | **Total Harvest (kg)** |  | **Number units consumed by HH** | **Number units sold** | **Number units damaged/lost used for animal feed** | **Number units saved for seed or in storage** | **Number of units given away/gift** | **Number of units given as in-kind payment/exchange** | **Value of each unit at market (Birr)** | **Total value of production (Birr)** |
| Cereals | 11.2.1 | Maize |  | 1 |  |  |  |  | |  | |  |  |  |  |  |  |  |  |  |  |  |
|  |  |  |  | 2 |  |  |  |  | |  | |  |  |  |  |  |  |  |  |  |  |  |
|  | 11.2.2 | Teff |  | 1 |  |  |  |  | |  | |  |  |  |  |  |  |  |  |  |  |  |
|  |  |  |  | 2 |  |  |  |  | |  | |  |  |  |  |  |  |  |  |  |  |  |
|  | 11.2.3 | Wheat |  | 1 |  |  |  |  | |  | |  |  |  |  |  |  |  |  |  |  |  |
|  |  |  |  | 2 |  |  |  |  | |  | |  |  |  |  |  |  |  |  |  |  |  |
|  | 11.2.4 | Barley |  | 1 |  |  |  |  | |  | |  |  |  |  |  |  |  |  |  |  |  |
|  |  |  |  | 2 |  |  |  |  | |  | |  |  |  |  |  |  |  |  |  |  |  |
|  | 11.2.5 | Sorghum |  | 1 |  |  |  |  | |  | |  |  |  |  |  |  |  |  |  |  |  |
|  |  |  |  | 2 |  |  |  |  | |  | |  |  |  |  |  |  |  |  |  |  |  |
|  | 11.2.6 | Oats/”Aja” |  | 1 |  |  |  |  | |  | |  |  |  |  |  |  |  |  |  |  |  |
|  |  |  |  | 2 |  |  |  |  | |  | |  |  |  |  |  |  |  |  |  |  |  |
|  | 11.2.9 | Other cereal *specify_____*  *(LCLOTH)* |  | 1 |  |  |  |  | |  | |  |  |  |  |  |  |  |  |  |  |  |
|  |  |  |  | 2 |  |  |  |  | |  | |  |  |  |  |  |  |  |  |  |  |  |
| Tubers Roots & Bulbs | 11.2.10 | Beets |  | 1 |  |  |  |  | |  | |  |  |  |  |  |  |  |  |  |  |  |
|  |  |  |  | 2 |  |  |  |  | |  | |  |  |  |  |  |  |  |  |  |  |  |
|  | 11.2.11 | Potato |  | 1 |  |  |  |  | |  | |  |  |  |  |  |  |  |  |  |  |  |
|  |  |  |  | 2 |  |  |  |  | |  | |  |  |  |  |  |  |  |  |  |  |  |
|  | 11.2.13 | Sweet potato– orange |  | 1 |  |  |  |  | |  | |  |  |  |  |  |  |  |  |  |  |  |
|  |  |  |  | 2 |  |  |  |  | |  | |  |  |  |  |  |  |  |  |  |  |  |
|  | 11.2.14 | Sweet potato – white |  | 1 |  |  |  |  | |  | |  |  |  |  |  |  |  |  |  |  |  |
|  |  |  |  | 2 |  |  |  |  | |  | |  |  |  |  |  |  |  |  |  |  |  |
|  | 11.2.15 | Godere/Taro/Tania |  | 1 |  |  |  |  | |  | |  |  |  |  |  |  |  |  |  |  |  |
|  |  |  |  | 2 |  |  |  |  | |  | |  |  |  |  |  |  |  |  |  |  |  |
|  | 11.2.16 | Yam |  | 1 |  |  |  |  | |  | |  |  |  |  |  |  |  |  |  |  |  |
|  |  |  |  | 2 |  |  |  |  | |  | |  |  |  |  |  |  |  |  |  |  |  |
|  | 11.2.17 | Garlic |  | 1 |  |  |  |  | |  | |  |  |  |  |  |  |  |  |  |  |  |
|  |  |  |  | 2 |  |  |  |  | |  | |  |  |  |  |  |  |  |  |  |  |  |
|  | 11.2.18 | Other root/tuber *specify______*  *(LROTH)* |  | 1 |  |  |  |  | |  | |  |  |  |  |  |  |  |  |  |  |  |
|  |  |  |  | 2 |  |  |  |  | |  | |  |  |  |  |  |  |  |  |  |  |  |
| Legumes | 11.2.19 | Horse Beans |  | 1 |  |  |  |  | |  | |  |  |  |  |  |  |  |  |  |  |  |
|  |  |  |  | 2 |  |  |  |  | |  | |  |  |  |  |  |  |  |  |  |  |  |
|  | 11.2.20 | Haricot Beans |  | 1 |  |  |  |  | |  | |  |  |  |  |  |  |  |  |  |  |  |
|  |  |  |  | 2 |  |  |  |  | |  | |  |  |  |  |  |  |  |  |  |  |  |
|  | 11.2.21 | Chick pea |  | 1 |  |  |  |  | |  | |  |  |  |  |  |  |  |  |  |  |  |
|  |  |  |  | 2 |  |  |  |  | |  | |  |  |  |  |  |  |  |  |  |  |  |
|  | 11.2.22 | Field Pea |  | 1 |  |  |  |  | |  | |  |  |  |  |  |  |  |  |  |  |  |
|  |  |  |  | 2 |  |  |  |  | |  | |  |  |  |  |  |  |  |  |  |  |  |
|  | 11.2.23 | Lentils |  | 1 |  |  |  |  | |  | |  |  |  |  |  |  |  |  |  |  |  |
|  |  |  |  | 2 |  |  |  |  | |  | |  |  |  |  |  |  |  |  |  |  |  |
|  | 11.2.26 | Soya beans |  | 1 |  |  |  |  | |  | |  |  |  |  |  |  |  |  |  |  |  |
|  |  |  |  | 2 |  |  |  |  | |  | |  |  |  |  |  |  |  |  |  |  |  |
|  | 11.2.27 | Fenugreek |  | 1 |  |  |  |  | |  | |  |  |  |  |  |  |  |  |  |  |  |
|  |  |  |  | 2 |  |  |  |  | |  | |  |  |  |  |  |  |  |  |  |  |  |
|  | 11.2.28 | Other legume *specify_____*  *(LLOTH)* |  | 1 |  |  |  |  | |  | |  |  |  |  |  |  |  |  |  |  |  |
|  |  |  |  | 2 |  |  |  |  | |  | |  |  |  |  |  |  |  |  |  |  |  |
| Cash crops | 11.2.29 | Coffee |  | 1 |  |  |  |  | |  | |  |  |  |  |  |  |  |  |  |  |  |
|  |  |  |  | 2 |  |  |  |  | |  | |  |  |  |  |  |  |  |  |  |  |  |
|  | 11.2.31 | Chat |  | 1 |  |  |  |  | |  | |  |  |  |  |  |  |  |  |  |  |  |
|  |  |  |  | 2 |  |  |  |  | |  | |  |  |  |  |  |  |  |  |  |  |  |
|  | 11.2.32 | Cotton |  | 1 |  |  |  |  | |  | |  |  |  |  |  |  |  |  |  |  |  |
|  |  |  |  | 2 |  |  |  |  | |  | |  |  |  |  |  |  |  |  |  |  |  |
|  | 11.2.33 | Sugar cane |  | 1 |  |  |  |  | |  | |  |  |  |  |  |  |  |  |  |  |  |
|  |  |  |  | 2 |  |  |  |  | |  | |  |  |  |  |  |  |  |  |  |  |  |
|  | 11.2.34 | Enset |  | 1 |  |  |  |  | |  | |  |  |  |  |  |  |  |  |  |  |  |
|  |  |  |  | 2 |  |  |  |  | |  | |  |  |  |  |  |  |  |  |  |  |  |
|  | 11.2.35 | Hops/ Gesho |  | 1 |  |  |  |  | |  | |  |  |  |  |  |  |  |  |  |  |  |
|  |  |  |  | 2 |  |  |  |  | |  | |  |  |  |  |  |  |  |  |  |  |  |
|  | 11.2.37 | Tobacco |  | 1 |  |  |  |  | |  | |  |  |  |  |  |  |  |  |  |  |  |
|  |  |  |  | 2 |  |  |  |  | |  | |  |  |  |  |  |  |  |  |  |  |  |
|  | 11.2.38 | Other cash crop *specify_____*  *(LCCOTH)* |  | 1 |  |  |  |  | |  | |  |  |  |  |  |  |  |  |  |  |  |
|  |  |  |  | 2 |  |  |  |  | |  | |  |  |  |  |  |  |  |  |  |  |  |
| Vegetables | 11.2.39 | Tomato |  | 1 |  |  |  |  | |  | |  |  |  |  |  |  |  |  |  |  |  |
|  |  |  |  | 2 |  |  |  |  | |  | |  |  |  |  |  |  |  |  |  |  |  |
|  | 11.2.40 | Pepper |  | 1 |  |  |  |  | |  | |  |  |  |  |  |  |  |  |  |  |  |
|  |  |  |  | 2 |  |  |  |  | |  | |  |  |  |  |  |  |  |  |  |  |  |
|  | 11.2.41 | Pumpkin |  | 1 |  |  |  |  | |  | |  |  |  |  |  |  |  |  |  |  |  |
|  |  |  |  | 2 |  |  |  |  | |  | |  |  |  |  |  |  |  |  |  |  |  |
|  | 11.2.42 | Carrot |  | 1 |  |  |  |  | |  | |  |  |  |  |  |  |  |  |  |  |  |
|  |  |  |  | 2 |  |  |  |  | |  | |  |  |  |  |  |  |  |  |  |  |  |
|  | 11.2.43 | Onion |  | 1 |  |  |  |  | |  | |  |  |  |  |  |  |  |  |  |  |  |
|  |  |  |  | 2 |  |  |  |  | |  | |  |  |  |  |  |  |  |  |  |  |  |
|  | 11.2.44 | Lettuce |  | 1 |  |  |  |  | |  | |  |  |  |  |  |  |  |  |  |  |  |
|  |  |  |  | 2 |  |  |  |  | |  | |  |  |  |  |  |  |  |  |  |  |  |
|  | 11.2.45 | Cauliflower |  | 1 |  |  |  |  | |  | |  |  |  |  |  |  |  |  |  |  |  |
|  |  |  |  | 2 |  |  |  |  | |  | |  |  |  |  |  |  |  |  |  |  |  |
|  | 11.2.46 | Cabbage |  | 1 |  |  |  |  | |  | |  |  |  |  |  |  |  |  |  |  |  |
|  |  |  |  | 2 |  |  |  |  | |  | |  |  |  |  |  |  |  |  |  |  |  |
|  | 11.2.48 | Kale/ Ethiopian cabbage |  | 1 |  |  |  |  | |  | |  |  |  |  |  |  |  |  |  |  |  |
|  |  |  |  | 2 |  |  |  |  | |  | |  |  |  |  |  |  |  |  |  |  |  |
|  | 11.2.49 | Moringa/Shiferaw |  | 1 |  |  |  |  | |  | |  |  |  |  |  |  |  |  |  |  |  |
|  |  |  |  | 2 |  |  |  |  | |  | |  |  |  |  |  |  |  |  |  |  |  |
|  | 11.2.50 | Swiss Chard |  | 1 |  |  |  |  | |  | |  |  |  |  |  |  |  |  |  |  |  |
|  |  |  |  | 2 |  |  |  |  | |  | |  |  |  |  |  |  |  |  |  |  |  |
|  | 11.2.51 | Other dark green leafy vegetable *specify_____ (LDGOTH)* |  | 1 |  |  |  |  | |  | |  |  |  |  |  |  |  |  |  |  |  |
|  |  |  |  | 2 |  |  |  |  | |  | |  |  |  |  |  |  |  |  |  |  |  |
|  | 11.2.52 | Other light green leafy vegetable *specify_____ (LLGOTH)* |  | 1 |  |  |  |  | |  | |  |  |  |  |  |  |  |  |  |  |  |
|  |  |  |  | 2 |  |  |  |  | |  | |  |  |  |  |  |  |  |  |  |  |  |
|  | 11.2.53 | Other vegetable *specify______*  *(LVOTH)* |  | 1 |  |  |  |  | |  | |  |  |  |  |  |  |  |  |  |  |  |
|  |  |  |  | 2 |  |  |  |  | |  | |  |  |  |  |  |  |  |  |  |  |  |
| Fruits | 11.2.54 | Mangoes |  | 1 |  |  |  |  | |  | |  |  |  |  |  |  |  |  |  |  |  |
|  |  |  |  | 2 |  |  |  |  | |  | |  |  |  |  |  |  |  |  |  |  |  |
|  | 11.2.56 | Avocado |  | 1 |  |  |  |  | |  | |  |  |  |  |  |  |  |  |  |  |  |
|  |  |  |  | 2 |  |  |  |  | |  | |  |  |  |  |  |  |  |  |  |  |  |
|  | 11.2.57 | Banana |  | 1 |  |  |  |  | |  | |  |  |  |  |  |  |  |  |  |  |  |
|  |  |  |  | 2 |  |  |  |  | |  | |  |  |  |  |  |  |  |  |  |  |  |
|  | 11.2.60 | Pineapple |  | 1 |  |  |  |  | |  | |  |  |  |  |  |  |  |  |  |  |  |
|  |  |  |  | 2 |  |  |  |  | |  | |  |  |  |  |  |  |  |  |  |  |  |
|  | 11.2.61 | Oranges |  | 1 |  |  |  |  | |  | |  |  |  |  |  |  |  |  |  |  |  |
|  |  |  |  | 2 |  |  |  |  | |  | |  |  |  |  |  |  |  |  |  |  |  |
|  | 11.2.62 | Apples |  | 1 |  |  |  |  | |  | |  |  |  |  |  |  |  |  |  |  |  |
|  |  |  |  | 2 |  |  |  |  | |  | |  |  |  |  |  |  |  |  |  |  |  |
|  | 11.2.64 | Papaya |  | 1 |  |  |  |  | |  | |  |  |  |  |  |  |  |  |  |  |  |
|  |  |  |  | 2 |  |  |  |  | |  | |  |  |  |  |  |  |  |  |  |  |  |
|  | 11.2.65 | Lime |  | 1 |  |  |  |  | |  | |  |  |  |  |  |  |  |  |  |  |  |
|  |  |  |  | 2 |  |  |  |  | |  | |  |  |  |  |  |  |  |  |  |  |  |
|  | 11.2.66 | Mandarin |  | 1 |  |  |  |  | |  | |  |  |  |  |  |  |  |  |  |  |  |
|  |  |  |  | 2 |  |  |  |  | |  | |  |  |  |  |  |  |  |  |  |  |  |
|  | 11.2.67 | Roman/Pomogranate |  | 1 |  |  |  |  | |  | |  |  |  |  |  |  |  |  |  |  |  |
|  |  |  |  | 2 |  |  |  |  | |  | |  |  |  |  |  |  |  |  |  |  |  |
|  | 11.2.70 | Citron/Tringo |  | 1 |  |  |  |  | |  | |  |  |  |  |  |  |  |  |  |  |  |
|  |  |  |  | 2 |  |  |  |  | |  | |  |  |  |  |  |  |  |  |  |  |  |
|  | 11.2.71 | Guava/Zeiytu |  | 1 |  |  |  |  | |  | |  |  |  |  |  |  |  |  |  |  |  |
|  |  |  |  | 2 |  |  |  |  | |  | |  |  |  |  |  |  |  |  |  |  |  |
|  | 11.2.72 | Gishita/Custardapple |  | 1 |  |  |  |  | |  | |  |  |  |  |  |  |  |  |  |  |  |
|  |  |  |  | 2 |  |  |  |  | |  | |  |  |  |  |  |  |  |  |  |  |  |
|  | 11.2.81 | Other fruit *specify_____*  *(LFOTH)* |  | 1 |  |  |  |  | |  | |  |  |  |  |  |  |  |  |  |  |  |
|  |  |  |  | 2 |  |  |  |  | |  | |  |  |  |  |  |  |  |  |  |  |  |
| Oilseeds | 11.2.82 | Castor Beans |  | 1 |  |  |  |  | |  | |  |  |  |  |  |  |  |  |  |  |  |
|  |  |  |  | 2 |  |  |  |  | |  | |  |  |  |  |  |  |  |  |  |  |  |
|  | 11.2.83 | Flax/Linseed |  | 1 |  |  |  |  | |  | |  |  |  |  |  |  |  |  |  |  |  |
|  |  |  |  | 2 |  |  |  |  | |  | |  |  |  |  |  |  |  |  |  |  |  |
|  | 11.2.84 | Nigerseed/neug |  | 1 |  |  |  |  | |  | |  |  |  |  |  |  |  |  |  |  |  |
|  |  |  |  | 2 |  |  |  |  | |  | |  |  |  |  |  |  |  |  |  |  |  |
|  | 11.2.85 | Cotton Seed |  | 1 |  |  |  |  | |  | |  |  |  |  |  |  |  |  |  |  |  |
|  |  |  |  | 2 |  |  |  |  | |  | |  |  |  |  |  |  |  |  |  |  |  |
|  | 11.2.86 | Ground nuts |  | 1 |  |  |  |  | |  | |  |  |  |  |  |  |  |  |  |  |  |
|  |  |  |  | 2 |  |  |  |  | |  | |  |  |  |  |  |  |  |  |  |  |  |
|  | 11.2.87 | Rapeseed |  | 1 |  |  |  |  | |  | |  |  |  |  |  |  |  |  |  |  |  |
|  |  |  |  | 2 |  |  |  |  | |  | |  |  |  |  |  |  |  |  |  |  |  |
|  | 11.2.88 | Safflower |  | 1 |  |  |  |  | |  | |  |  |  |  |  |  |  |  |  |  |  |
|  |  |  |  | 2 |  |  |  |  | |  | |  |  |  |  |  |  |  |  |  |  |  |
|  | 11.2.89 | Sesame |  | 1 |  |  |  |  | |  | |  |  |  |  |  |  |  |  |  |  |  |
|  |  |  |  | 2 |  |  |  |  | |  | |  |  |  |  |  |  |  |  |  |  |  |
|  | 11.2.90 | Other oil seeds specify______  (LOSOTH) |  | 1 |  |  |  |  | |  | |  |  |  |  |  |  |  |  |  |  |  |
|  |  |  |  | 2 |  |  |  |  | |  | |  |  |  |  |  |  |  |  |  |  |  |
| Spices |  | *Ginger* |  | 1 |  |  |  |  | |  | |  |  |  |  |  |  |  |  |  |  |  |
|  |  | *Turmeric* |  | 2 |  |  |  |  | |  | |  |  |  |  |  |  |  |  |  |  |  |
|  |  | *Cardamom* |  | 1 |  |  |  |  | |  | |  |  |  |  |  |  |  |  |  |  |  |
|  |  | *Coriander* |  | 2 |  |  |  |  | |  | |  |  |  |  |  |  |  |  |  |  |  |
|  |  | *Other Spices Specify _______* |  | 1 |  |  |  |  | |  | |  |  |  |  |  |  |  |  |  |  |  |

## Section 3: Crop Production Expenses

Please tell me all the expenses on purchased inputs that your household incurred on **CROP Production** during January 2013-December 2013.

Table 11.3

|  | **Input type** | **Used Jan ‘13-Dec ‘13 (1=yes,0=no 98=Don’t Know)** | **Acquired Jan ‘13-Dec ‘13 (1=yes,0=no 98=Don’t Know)** | | **Crop 1** | **Cost 1** | **Source*1** | **Crop 2** | **Cost 2** | | **Source* 2** | **Crop 3** | **Cost 3** | | **Source* 3** | **Total cost (Birr)** |
| --- | --- | --- | --- | --- | --- | --- | --- | --- | --- | --- | --- | --- | --- | --- | --- | --- |
| 11.3.1 | Seed - Local | ESEED12U | ESEED12 | | ESEED1 | ESEED1C | ESEED1S | ESEED2 | ESEED2C | | ESEED2S | ESEED3 | ESEED3C | | ESEED3C | ESEEDTOT |
|  | Seed – Improved | ESEEDI12U | ESEEDI12 | | ESEEDI1 | ESEEDI1C | ESEEDI1S | ESEEDI2 | ESEEDI2C | | ESEEDI2S | ESEEDI3 | ESEEDI3C | | ESEEDI3C | ESEEDITOT |
|  | Seed – hybrid | ESEEDH12U | ESEEDH12 | | ESEEDH1 | ESEEDH1C | ESEEDH1S | ESEEDH2 | ESEEDH2C | | ESEEDH2S | ESEEDH3 | ESEEDH3C | | ESEEDH3C | ESEEDHTOT |
| 11.3.2 | Seedling or plantlets (local) | EPLANT12U | EPLANT12 | | EPLANT1 | EPLANT1C | EPLANT1S | EPLANT2 | EPLANT2C | | EPLANT2S | EPLANT3 | EPLANT3C | | EPLANT3C | EPLANTTOT |
|  | Seedling or plantlets (improved) | EPLANTI12U | EPLANTI12 | | EPLANTI1 | EPLANTI1C | EPLANTI1S | EPLANTI2 | EPLANTI2C | | EPLANTI2S | EPLANTI3 | EPLANTI3C | | EPLANTI3C | EPLANTITOT |
| 11.3.3 | Inorganic fertilizer | EINORG12U | EINORG12 | | EINORG1 | EINORG1C | EINORG1S | EINORG2 | EINORG2C | | EINORG2S | EINORG3 | EINORG3C | | EINORG3C | EINORGTOT |
| 11.3.4 | Foliar fertilizers | EFOL12U | EFOL12 | | EFOL1 | EFOL1C | EFOL1S | EFOL2 | EFOL2C | | EFOL2S | EFOL3 | EFOL3C | | EFOL3C | EFOLT |
| 11.3.5 | Manures | EMANURE12U | EMANURE12 | | EMANURE1 | EMANURE1C | EMANURE1S | EMANURE2 | EMANURE2C | | EMANURE2S | EMANURE3 | EMANURE3C | | EMANURE3C | EMANURETOT |
| 11.3.6 | Irrigation | EIRRIG12U | EIRRIG12 | | EIRRIG1 | EIRRIG1C | EIRRIG1S | EIRRIG2 | EIRRIG2C | | EIRRIG2S | EIRRIG3 | EIRRIG3C | | EIRRIG3C | EIRRIGTOT |
| 11.3.7 | Mulches | EMULCH12U | EMULCH12 | | EMULCH1 | EMULCH1C | EMULCH1S | EMULCH2 | EMULCH2C | | EMULCH2S | EMULCH3 | EMULCH3C | | EMULCH3C | EMULCHTOT |
| 11.3.8 | Cover crops | ECOVER12U | ECOVER12 | | ECOVER1 | ECOVER1C | ECOVER1S | ECOVER2 | ECOVER2C | | ECOVER2S | ECOVER3 | ECOVER3C | | ECOVER3C | ECOVERTOT |
| 11.3.9 | Pesticides | EPEST12U | EPEST12 | | EPEST1 | EPEST1C | EPEST1S | EPEST2 | EPEST2C | | EPEST2S | EPEST3 | EPEST3C | | EPEST3C | EPESTTOT |
| 11.3.10 | Herbicides | EHERB12U | EHERB12 | | EHERB1 | EHERB1C | EHERB1S | EHERB2 | EHERB2C | | EHERB2S | EHERB3 | EHERB3C | | EHERB3C | EHERBTOT |
| 11.3.11 | Land rented in | ERENT12U | ERENT12 | | ERENT1 | ERENT1 C | ERENT1S | ERENT2 | ERENT2C | | ERENT2S | ERENT3 | ERENT3C | | ERENT3C | ERENTTOT |
| 11.3.12 | Storage | ESTORAGE12U | ESTORAGE12 | | ESTORAGE1 | ESTORAGE1C | ESTORAGE1S | ESTORAGE2 | ESTORAGE2C | | ESTORAGE2S | ESTORAGE3 | ESTORAGE3C | | ESTORAGE3C | ESTORAGETOT |
| 11.3.13 | Processing | EPROCESS12U | EPROCESS12 | | EPROCESS1 | EPROCESS1C | EPROCESS1S | EPROCESS2 | EPROCESS2C | | EPROCESS2S | EPROCESS3 | EPROCESS3C | | EPROCESS3C | EPROCESSTOT |
| 11.3.14 | Transportation of produce – field to house | ETPORT12U | ETPORT12 | | ETPORT1 | ETPORT1C | ETPORT1S | ETPORT2 | ETPORT2C | | ETPORT2S | ETPORT3 | ETPORT3C | | ETPORT3C | ETPORTTOT |
| 11.3.15 | Transportation of produce –house to processing/market | ETPMARK12U | ETPMARK12 | | ETPMARK1 | ETPMARK1C | ETPMARK1S | ETPMARK2 | ETPMARK2C | | ETPMARK2S | ETPMARK3 | ETPMARK3C | | ETPMARK3C | ETPMARKTOT |
| 11.3.16 | Hired animal traction | EANIM12U | EANIM12 | | EANIM1 | EANIM1C | EANIM1S | EANIM2 | EANIM2C | | EANIM2S | EANIM3 | EANIM3C | | EANIM3C | EANIMTOT |
| 11.3.17 | Hired farm implements (e.g., tractor, knapsack sprayer) | EIMP12U | EIMP12 | | EIMP1 | EIMP1C | EIMP1S | EIMP2 | EIMP2C | | EIMP2S | EIMP3 | EIMP3C | | EIMP3C | EIMPTOT |
| 11.3.18 | Others (specify_____) EOTHSPE | EOTH12U | EOTH12 | | EOTH1 | EOTH1C | EOTH1S | EOTH2 | EOTH2C | | EOTH2S | EOTH3 | EOTH3C | | EOTH3C | EOTHTOT |
| Source Codes:  Agriculture Research Center  Agriculture Office | | | | Neighbour/Friend  University  Shop | | | | | | Borrowed  Other | | | |  | | |

## Section 4: Estimation of Livestock Ownership

Please tell me of the livestock your household currently owns. *[HELP THE RESPONDENT TO ESTIMATE THE PRESENT VALUE OF LIVESTOCK IF HE/SHE WERE TO SELL]*

| 11.4.1 | Did your householdraise/buy/own/sell any livestock during the past year? (including beekeeping) | 1. Yes0. No 98. Don’t know | HHLIVESTOCK |
| --- | --- | --- | --- |

Table 11.4 – Current Ownership

|  | **Animal type** | **Quantity**  (if 0, skip columns to right) | **Total Value (Birr)** | **Owned by (codes below)** |
| --- | --- | --- | --- | --- |
| 11.4.2 | Local oxen | ELBULLQ | ELBULLV | ELBULLO |
| 11.4.3 | Exotic oxen | EBULLQ | EBULLV | EBULLO |
| 11.4.4 | Crossbreed bulls | ECBULLQ | ECBULLV | ECBULLO |
| 11.4.5 | Local cows | ELCOWQ | ELCOWV | ELCOWO |
| 11.4.6 | Exotic cows | ECOWQ | ECOWV | ECOWO |
| 11.4.7 | Local heifers | ELHEIQ | ELHEIV | ELHEIO |
| 11.4.8 | Exotic heifers | EHEIQ | EHEIV | EHEIO |
| 11.4.9 | Crossbreed cows | ECCOWQ | ECCOWV | ECCOWO |
| 11.4.10 | Local calves | ELCALVQ | ELCALVV | ELCALVO |
| 11.4.11 | Exotic calves | ECALVQ | ECALVV | ECALVO |
| 11.4.12 | Crossbreed calves | ECCALVQ | ECCALVV | ECCALVO |
| 11.4.13 | Camels | ECAMELQ | ECAMELV | ECAMELO |
| 11.4.14 | Sheep | ESHEEPQ | ESHEEPV | ESHEEPO |
| 11.4.15 | Local goats | ELGOATQ | ELGOATV | ELGOATO |
| 11.4.16 | Exotic goats | EGOATQ | EGOATV | EGOATO |
| 11.4.17 | Donkeys | EDONKQ | EDONKV | EDONKO |
| 11.4.18 | Horse | EHORSQ | EHORSV | EHORSO |
| 11.4.19 | Mule | EMULEQ | EMULEV | EMULEO |
| 11.4.20 | Local chicken | ELCHICKQ | ELCHICKV | ELCHICKO |
| 11.4.21 | Improved chicken | EIMCHICKQ | EIMCHICKV | EIMCHICKO |
| 11.4.22 | Other poultry (specify___) EOTHPOU | EOTHPOUQ | EOTHPOUV | EOTHPOUO |
| 11.4.23 | Traditional Beehives | EBEEQ | EBEEV | EBEEO |
| 11.4.24 | Modern Beehives | EMBEEQ | EMBEEV | EMBEEO |
| 11.4.25 | Other livestock (specify___) EOTHLIV | EOTHLIVQ | EOTHLIVV | EOTHLIVO |

Owned by: ***1. Head of Household 2.HH Father 3.HH First Wife 4.HH Mother 5.HH Second wife 6.Other 7.All 8. N/A*(Select as many as apply)***

## Section 5: Livestock production

Table 11.4 Please tell us about your household’s livestock production during January 2013-December 2013.

|  | **Type** | **Product** | **Unit** | **No. of units produced/born** | **No. of units consumed by household** | **No. of units sold** | **No. of units given away/lost/stolen** | **No. of units died** | **Unit value (Birr)** |
| --- | --- | --- | --- | --- | --- | --- | --- | --- | --- |
| 11.5.1 | Cattle | Live animals |  | LCANIB |  | LCANIS | LCANIL | LCANID | LCANIV |
| 11.5.2 |  | Beef | LCBEEFU | LCBEEFP | LCBEEFC | LCBEEFS | LCBEEFL |  | LCBEEFV |
| 11.5.3 |  | Milk | LCMILKU | LCMILKP | LCMILKC | LCMILKS | LCMILKL |  | LCMILKV |
| 11.5.4 |  | Butter | LCBUTU | LCBUTP | LCBUTC | LCBUTS | LCBUTL |  | LCBUTV |
| 11.5.5 |  | Cheese | LCCHEEU | LCCHEEP | LCCHEEC | LCCHEES | LCCHEEL |  | LCCHEEV |
| 11.5.6 |  | Hides | LCHIDESU | LCHIDESP | LCHIDESC | LCHIDESS | LCHIDESL |  | LCHIDESV |
| 11.5.7 |  | Cattle Manure | LCCATTU | LCCATTP | LCCATTC | LCCATTS | LCCATTL |  | LCCATTV |
| 11.5.8 |  | Yoghurt/sour milk | LCYOGU | LCYOGP | LCYOGC | LCYOGS | LCYOGL |  | LCYOGV |
| 11.5.9 |  | Others (specify_______) LCOTHSPE | LCOTHU | LCOTHP | LCOTHC | LCOTHS | LCOTHL |  | LCOTHV |
| 11.5.10 | Camels | Live Animals | LCAMU | LCAMP | LCAMC | LCAMS | LCAML | LCAMD | LCAMV |
| 11.5.11 | Sheep | Live animals |  | LSANIB |  | LSANIS | LSANIL | LSANID | LSANIV |
| 11.5.12 |  | Meat | LSMEATU | LSMEATP | LSMEATC | LSMEATS | LSMEATL |  | LSMEATV |
| 11.5.13 |  | Wool | LSWOOLU | LSWOOLP | LSWOOLC | LSWOOLS | LSWOOLL |  | LSWOOLV |
| 11.5.14 |  | Skin/Hides | LSKINU | LSKINP | LSKINC | LSKINS | LSKINL |  | LSKINV |
| 11.5.15 |  | Sheep manure | LSMANU | LSMANP | LSMANC | LSMANS | LSMANL |  | LSMANV |
| 11.5.16 | Goats | Live animals |  | LGANIB |  | LGANIS | LGANIL | LGANID | LGANIV |
| 11.5.17 |  | Meat | LGMEATU | LGMEATP | LGMEATC | LGMEATS | LGMEATL |  | LGMEATV |
| 11.5.18 |  | Milk | LGMILKU | LGMILKP | LGMILKC | LGMILKS | LGMILKL |  | LGMILKV |
| 11.5.19 |  | Skin/Hides | LGSKINU | LGSKINP | LGSKINC | LGSKINS | LGSKINL |  | LGSKINV |
| 11.5.20 |  | Goat manure | LGMANU | LGMANP | LGMANC | LGMANS | LGMANL |  | LGMANV |
| 11.5.21 | Poultry | Live birds |  | LPOBIRDB | LPOBIRDC | LPOBIRDS | LPOBIRDL | LPOBIRDD | LPOBIRDV |
| 11.5.22 |  | Eggs | LPOEGGSU | LPOEGGSP | LPOEGGSC | LPOEGGSS | LPOEGGSL | LPOEGGSD | LPOEGGSV |
| 11.5.23 |  | Bird manure | LPOMANU | LPOMANP | LPOMANC | LPOMANS | LPOMANL |  | LPOMANV |
| 11.5.24 | Donkeys | Live animals |  | LDANIB |  | LDANIS | LDANIL | LDANILD | LDANIV |
| 11.5.25 | Mules | Live Animals |  | LMANIB |  | LMANIS | LMANIL | LMANID | LMANIV |
| 11.5.26 | Horse | Live Animals |  | LHANIB |  | LHANIS | LHANIL | LHANID | LHANIV |
| 11.5.27 |  | Donkey, Horse, or Mule Manure | LDMANU | LDMANP | LDMANC | LDMANS | LDMANL |  | LDMANV |
| 11.5.28 | Beekeeping | Honey | LHONEYU | LHONEYP | LHONEYC | LHONEYS | LHONEYL |  | LHONEYV |
| 11.5.29 |  | Wax | LBWAXU | LBWAXP | LBWAXC | LBWAXS | LBWAXL |  | LBWAXV |
| 11.5.30 |  | Colony |  | LBCOLP |  | LBCOLS | LBCOLL | LBCOLD | LBCOLV |
| 11.5.31 |  | Propolis | LBPROPU | LBPROPP |  | LBPROPS | LBPROPS |  | LBPROPS |
| 11.5.32 | Others (specify_______) LOTHSPE | | LOTHU | LOTHP | LOTHC | LOTHS | LOTHL | LOTHD | LOTHL |

## Section 6: Livestock Expenses

Please tell me all the expenses that your household incurred on ALL LIVESTOCK ACTIVITIES during January 2013- December 2013.

Table 11.6

|  | **Activity** | **Input category** | **Input type** | **Frequency per year** | **Qty used each time** | **Units** | **Unit value (Birr)** | **Total value (Birr)** |
| --- | --- | --- | --- | --- | --- | --- | --- | --- |
| 11.6.1 | Feeding | Purchased feed or fodder | Straw | LFSTRAWF | LFSTRAWQ | LFSTRAWU | LFSTRAWV | LFSTRAWS |
| 11.6.2 |  |  | Hay | LFHAYF | LFHAYQ | LFHAYU | LFHAYV | LFHAYS |
| 11.6.3 |  |  | Grain | LFGRAINF | LFGRAINQ | LFGRAINU | LFGRAINV | LFGRAINS |
| 11.6.4 |  |  | Concentrate (incl. maize bran) | LFCONCF | LFCONCQ | LFCONCU | LFCONCV | LFCONCS |
| 11.6.5 |  |  | Salt | LFSALTF | LFSALTQ | LFSALTU | LFSALTV | LFSALTS |
| 11.6.6 |  |  | Crop residue | LFCRRESF | LFCRRESQ | LFCRRESU | LFCRRESV | LFCRRESS |
| 11.6.7 |  |  | Grass | LFGRASSF | LFGRASSQ | LFGRASSU | LFGRASSV | LFGRASSS |
| 11.6.8 |  |  | Other (specify) LFOTHSPE | LFOTHF | LFOTHQ | LFOTHU | LFOTHV | LFOTHS |
|  | Beekeeping | Structure | Beehive | LBEEHF | LBEEHQ | LBEEHU | LBEEHV | LBEEHS |
|  |  |  | Frames | LFRAMEF | LFRAMEQ | LFRAMEU | LFRAMEV | LFRAMS |
|  |  |  | Queen Excluder | LQUEXF | LQUEXQ | LQUEXU | LQUEXV | LQUEXS |
|  |  | Feed | Sugar | LSUGF | LSUGQ | LSUGU | LSUGV | LSUGS |
|  |  |  | Flower seeds | LFLOWSF | LFLOWSQ | LFLOWSU | LFLOWSV | LFLOWSS |
|  |  | Pest Control | Pest Control Supplies | LPESTF | LPESTQ | LPESTU | LPESTV | LPESTS |
|  |  | Harvesting | Protective Gear | LPROCTF | LPROCTQ | LPROCTU | LPROCTV | LPROCTS |
|  |  |  | Smoking Kit, | LSMOKEF | LSMOKEQ | LSMOKEU | LSMOKEV | LSMOKES |
|  |  |  | Purifying Centrifuge | LSMOKEF | LSMOKEQ | LSMOKEU | LSMOKEV | LSMOKES |
|  |  | Bees | Colony | LCOLONF | LCOLONQ | LCOLONU | LCOLONV | LCOLONS |
| 11.6.9 | Livestock health care | Medicines | Deworming | LHMED1F | LHMED1Q | LHMED1U | LHMED1V | LHMED1S |
| 11.6.10 |  |  | LHMED2T | LHMED2F | LHMED2Q | LHMED2U | LHMED2V | LHMED2S |
| 11.6.11 |  |  | LHMED3T | LHMED3F | LHMED3Q | LHMED3U | LHMED3V | LHMED3S |
| 11.6.12 |  | Vaccines | LVACC1T | LVACC1F | LVACC1Q | LVACC1U | LVACC1V | LVACC1S |
| 11.6.13 |  |  | LVACC2T | LVACC2F | LVACC2Q | LVACC2U | LVACC2V | LVACC2S |
| 11.6.14 |  | Veterinary services | Acaricides | LVET1F | LVET1Q | LVET1U | LVET1V | LVET1S |
| 11.6.15 |  |  | LVET2T | LVET2F | LVET2Q | LVET2U | LVET2V | LVET2S |
| 11.6.16 |  |  | LVET3T | LVET3F | LVET3Q | LVET3U | LVET3V | LVET3S |
| 11.6.17 | Artificial insemination |  |  | LSEM1F | LSEM1Q | LSEM1U | LSEM1V | LSEM1S |
| 11.6.18 |  |  |  | LSEM2F | LSEM2Q | LSEM2U | LSEM2V | LSEM2S |
| 11.6.19 | Bull service |  |  | LBULLF | LBULLQ | LBULLU | LBULLV | LBULLS |
| 11.6.20 | Purchase of livestock |  | LPL1T | LPL1F | LPL1Q | LPL1U | LPL1V | LPL1S |
| 11.6.21 |  |  | LPL2T | LPL2F | LPL2Q | LPL2U | LPL2V | LPL2S |
| 11.6.22 |  |  | LPL3T | LPL3F | LPL3Q | LPL3U | LPL3V | LPL3S |
| 11.6.23 |  |  | LPL4T | LPL4F | LPL4Q | LPL4U | LPL4V | LPL4S |
| 11.6.24 | Construction of barns/”beret” |  | LBARNT | LBARNF | LBARNQ | LBARNU | LBARNV | LBARNS |
| 11.6.25 | Cleaning barns/”beret”, etc |  | LMAINT1T | LMAINT1F | LMAINT1Q | LMAINT1U | LMAINT1V | LMAINT1S |
| 11.6.26 |  |  | LMAINT2T | LMAINT2F | LMAINT2Q | LMAINT2U | LMAINT2V | LMAINT2S |
| 11.6.27 | Transport of livestock/products |  | LTRANSPT | LTRANSPF | LTRANSPQ | LTRANSPU | LTRANSPV | LTRANSPS |
| 11.6.28 | Processing of dairy products |  | LPROCEST | LPROCESF | LPROCESQ | LPROCESU | LPROCESV | LPROCESS |
| 11.6.29 | Other (specify) LOTH1SPE |  | LOTH1T | LOTH1F | LOTH1Q | LOTH1U | LOTH1V | LOTH1S |
| 11.6.30 | Other (specify) LOTH2SPE |  | LOTH2T | LOTH2F | LOTH2Q | LOTH2U | LOTH2V | LOTH2S |

## Section 7: Crop and Livestock Product Markets

I am going to ask you about places where your household usually sold crop or livestock production over the 12-month period of January 2013 to December 2013.

Table 11.7

|  | **Question** | **1=at farm gate**  **2=at nearby market**  **3=at more distant/larger market**  **4=through farmers’ group**  **5= other**  **6 = N/A**  **7 – DON’T KNOW** | **Var. Name** |
| --- | --- | --- | --- |
| 11.7.1 | Where do you mostly sell your food crops? |  | PFOOD |
| 11.7.2 | Where do you mostly sell your traditional cash crops (coffee, chat, tea, cotton, etc.)? |  | PCROP |
| 11.7.3 | Where do you mostly sell your live animals/poultry? |  | PANIMAL |
| 11.7.4 | Where do you mostly sell your milk? |  | PMILK |
| 11.7.5 | Where do you mostly sell your meat? |  | PMEAT |
| 11.7.6 | Where do you mostly sell your non-food animal products? E.g. Hides, skins, etc |  | PNONFOOD |

## Section 8: Agricultural Technologies and Management Practices

Have you or other members of your household used these practices or tools in the 12-month period of January 2013 to December 2013?

Table 11.8

|  | **Practice** | **Household/any member used**(1=Yes 0=No 98=Don’t Know) | **Var. Name** |
| --- | --- | --- | --- |
| 11.8.1 | Allowed land to go fallow for one or more seasons |  | TFALLOW |
| 11.8.2 | Used burning to clear land |  | TBURN |
| 11.8.3 | Planted crops in rows |  | TROWS |
| 11.8.4 | Used intercropping |  | TINTERCROP |
| 11.8.5 | Used crop rotation |  | TCROPROTA |
| 11.8.6 | Used improved seeds/crops |  | TIMPROSEED |
| 11.8.7 | Grew drought-tolerant crops |  | TDROUGHTC |
| 11.8.8 | Grew non-traditional crops |  | TNONTRADCROP |
| 11.8.9 | Tended non-improved livestock |  | TNONIMPSTOCK |
| 11.8.10 | Tended improved livestock |  | TIMPSTOCK |
| 11.8.11 | Used hoe with long handle |  | TLONGHOE |
| 11.8.12 | Used a wheelbarrow |  | TWBARROW |
| 11.8.13 | Used another farm implement (spade, etc.) |  | TANOTHER |
| 11.8.14 | Used inorganic fertilizer |  | TINORGAN |
| 11.8.15 | Used organic fertilizer |  | TORGANIC |
| 11.8.16 | Used agrochemicals in the field (e.g., pesticides, herbicides) |  | TAGROCHEMFI |
| 11.8.17 | Used agrochemicals for storage/post-harvest (e.g. pesticides) |  | TAGROCHEMST |
| 11.8.18 | Local pest management practices (e.g., ash, urine, pepper) |  | TLOCALPEST |
| 11.8.19 | Used integrated pest management |  | TPESTMAG |
| 11.8.20 | Used integrated soil fertility management |  | TSOILINT |
| 11.8.21 | Used irrigation |  | TIRRIGAT |
| 11.8.23 | Used animal traction for plowing |  | TANPLOW |
| 11.8.24 | Used animal traction for weeding |  | TANWEED |
| 11.8.25 | Used mechanized plowing |  | TMECPLOW |
| 11.8.26 | Used mechanized harvesting |  | TMECHARV |
| 11.8.27 | Vaccinated livestock or poultry |  | TVACPOUTRY |
| 11.8.28 | Used improved feed for livestock or poultry (either purchased or prepared at home) |  | TIMPFEED |
| 11.8.29 | Used aquaculture |  | TAQUACUL |
| 11.8.30 | Used improved drying methods (e.g., mats, tarpaulins, racks, concrete) |  | TIMPDRY |
| 11.8.31 | Used improved storage techniques (e.g., improved granaries, cribs, silos) |  | TIMPSTORA |
| 11.8.32 | Used improved processing technologies (e.g., threshers) |  | TIMPPROC |
| 11.8.33 | Added value to any agricultural production (crop or livestock) |  | TADDVAL |
| 11.8.34 | Used improved means of transportation to move water, firewood, or crop or livestock production (e.g., bicycle, motorbike, wheelbarrow) |  | TIMPTRANS |
| 11.8.35 | Used improved marketing (group marketing or sale at market rather than farm gate) |  | TIMPMARKT |
| 11.8.36 | Used modern, deck system for beekeeping |  | TIMPBEEHV |
| 11.8.37 | Practiced planting forage or providing sugar as feed for bees |  | TIMPFEEDB |
| 11.8.38 | Practiced pest management for beehives |  | TIMPIMPBEEH |
| 11.8.39 | Other (specify___________) TOTHSPE |  | TOTH |

## Section 9: Farm Labour for All Farm Activities

Table 11.9

| 11.9.1 | In the last 12 months, did your household hire any farm labour for either livestock or crop production? | 1=Yes  0=No 98 = don’t know | LABOUR |
| --- | --- | --- | --- |

Please tell me all the labour resources that your household used during this period.

|  | **Activity** | **Did you use family labor for this activity?** (1=yes, 0=no, 98=Don’t Know) | **If yes, estimate man days**  **(partial days allowed)** | **Did you use cooperative labor (debo) for this activity?**  (1=yes, 0=no, 98=Don’t Know) | **If yes, estimate man days**  **(partial days allowed)** | **Did you hire labor for this activity?** (1=yes, 0=no, 98=Don’t Know) | **If yes, estimate man days**  **(partial days allowed)** | **If yes (hire), total cost (Birr)** |
| --- | --- | --- | --- | --- | --- | --- | --- | --- |
| **Crop related activities** | | | | | | | | |
| 11.9.2 | Land preparation before planting (incl. bush clearing, first ploughing, etc) | LCPREPF | LCPREPFD | LCPREPE | LCPREPED | LCPREPH | LCPREPHD | LCPREPV |
| 11.9.3 | Planting | LCPLANTF | LCPLANTFD | LCPLANTE | LCPLANTED | LCPLANTH | LCPLANTHD | LCPLANTV |
| 11.9.4 | Weeding (manual or chemical) | LCWEEDF | LCWEEDFD | LCWEEDE | LCWEEDED | LCWEEDH | LCWEEDHD | LCWEEDV |
| 11.8.5 | Harvesting, threshing, etc | LCHARVF | LCHARVFD | LCHARVE | LCHARVED | LCHARVH | LCHARVHD | LCHARVV |
| 11.9.6 | Irrigating | LCIRRIF | LCIRRIFD | LCIRRIE | LCIRRIED | LCIRRIH | LCIRRIHD | LCIRRIV |
|  | Application of manures or agrochemicals (herbicide, pesticide, insecticide) | LCCHEMF | LCCHEMFD | LCCHEME | LCCHEMED | LCCHEMH | LCCHEMHD | LCCHEMV |
|  | IPM | LCCIPMF | LCCIPMFD | LCCIPME | LCCIPMED | LCCIPMH | LCCIPMHD | LCCIPMV |
| 11.9.7 | Other (specify_____) LCROTHSPE | LCROTHF | LCROTHFD | LCROTHE | LCROTHED | LCROTHH | LCROTHHD | LCROTHV |
| **Livestock related activities** | | | | | | | | |
| 11.9.8 | Grazing animals | LLGRAZF | LLGRAZFD | LLGRAZE | LLGRAZED | LLGRAZEH | LLGRAZEHD | LLGRAZEV |
| 11.9.9 | Watering animals | LLWATF | LLWATFD | LLWATE | LLWATED | LLWATERH | LLWATERHD | LLWATERV |
| 11.9.10 | Spraying/Dipping/Washing | LLSPRF | LLSPRFD | LLSPRE | LLSPRED | LLSPRAYH | LLSPRAYHD | LLSPRAYV |
| 11.9.11 | Milking or milk vending/processing | LLMILKF | LLMILKFD | LLMILKE | LLMILKED | LLMILKH | LLMILKHD | LLMILKV |
|  | Beekeeping Activities | LLBEESF | LLBEESFD | LLBEESE | LLBEESED | LLBEESH | LLBEESHD | LLBEESV |
| 11.9.12 | Other (specify_____) LLOTHSPE | LLOTHF | LLOTHFD | LLOTHE | LLOTHED | LLOTHH | LLOTHHD | LLOTHV |

#

# Module 12 – Income & Expenditure

## Section 1: Other Household Income Sources

*Now, please tell me about other sources of income that your household received during January 2013- December 2013. Please include income from all members of your household.*

Table 12.1

|  | **Type of income received** | **Income from this activity? (1=Yes, 0=No)** (if no, skip to next row) | **Costs involved in securing this income?** (1=Yes 0=No) | **If yes, mention costs incurred** | **If yes, total value of costs (Birr)** | **Form of income received 1=*Cash 2= Non Cash*** | **Amount (e.g. no. of sacks, tins)** | **Units (if not cash)** | **Unit value (Birr)** | **Total value (Birr)** | **Final income (Birr)** |
| --- | --- | --- | --- | --- | --- | --- | --- | --- | --- | --- | --- |
| 12.1.1 | Agricultural labor on other's farm (crop or livestock) | MAGI | MAGC | MAGT | MAGCV | MAGF | MAGA | MAGU | MAGUV | MAGTV | MAGTVC |
| 12.1.2 | Non-agricultural employment – casual, temporary, or formal/salary (incl. house help, construction, etc.) | MNOAGI | MNOAGC | MNOAGT | MNOAGCV | MNOAGF | MNOAGA | MNOAGU | MNOAGUV | MNOAGTV | MNOAGTVC |
| 12.1.3 | Pension | MPENSI | MPENSC | MPENST | MPENSCV | MPENSF | MPENSA | MPENSU | MPENSUV | MPENSTV | MPENSTVC |
| 12.1.4 | Assistance from Govt/NGO/UN (including food, seed, or livestock aid) | MASSTI | MASSTC | MASSTT | MASSTCV | MASSTF | MASSTA | MASSTU | MASSTUV | MASSTTV | MASSTTVC |
| 12.1.5 | Remittance income | MREMITI | MREMITC | MREMITT | MREMITCV | MREMITF | MREMITA | MREMITU | MREMITUV | MREMITTV | MREMITTVC |
| 12.1.6 | Assistance from relatives or friends | MASRELI | MASRELC | MASRELT | MASRELCV | MASRELF | MASRELA | MASRELU | MASRELUV | MASRELTV | MASRELTVC |
| 12.1.7 | Gifts | MGIFTI | MGIFTC | MGIFTT | MGIFTCV | MGIFTF | MGIFTA | MGIFTU | MGIFTUV | MGIFTTV | MGIFTTVC |
| 12.1.8 | Rent out (land, animals, tools/goods) | MRENTI | MRENTC | MRENTT | MRENTCV | MRENTF | MRENTA | MRENTU | MRENTUV | MRENTTV | MRENTTVC |
| 12.1.9 | Sale of fuel wood | MWOODI | MWOODC | MWOODT | MWOODCV | MWOODF | MWOODA | MWOODU | MWOODUV | MWOODTV | MWOODTVC |
| 12.1.10 | Sale of poles/trees | MPOLEI | MPOLEC | MPOLET | MPOLECV | MPOLEF | MPOLEA | MPOLEU | MPOLEUV | MPOLETV | MPOLETVC |
| 12.1.11 | Sale of charcoal | MCHARCI | MCHARCC | MCHARCT | MCHARCCV | MCHARCF | MCHARCA | MCHARCU | MCHARCUV | MCHARCTV | MCHARCTVC |
| 12.1.12 | Sale of dung (fuel) | MDUNGI | MDUNGC | MDUNGT | MDUNGCV | MDUNGF | MDUNGA | MDUNGU | MDUNGUV | MDUNGCTV | MDUNGTVC |
| 12.1.13 | Sale of handicrafts | MCRAFTI | MCRAFTC | MCRAFTT | MCRAFTCV | MCRAFTF | MCRAFTA | MCRAFTU | MCRAFTUV | MCRAFTTV | MCRAFTTVC |
| 12.1.14 | Sale of prepared food/drinks/ operating a restaurant | MFOODI | MFOODC | MFOODT | MFOODCV | MFOODF | MFOODA | MFOODU | MFOODUV | MFOODTV | MFOODTVC |
| 12.1.15 | Sale of beverages or local brew | MBREWI | MBREWC | MBREWT | MBREWCV | MBREWF | MBREWA | MBREWU | MBREWUV | MBREWTV | MBREWTVC |
| 12.1.16 | Shop keeping | MSHOPI | MSHOPC | MSHOPT | MSHOPCV | MSHOPF | MSHOPA | MSHOPU | MSHOPUV | MSHOPTV | MSHOPTVC |
| 12.1.17 | Transportation (own work or rental out) | MBODAI | MBODAC | MBODAT | MBODACV | MBODAF | MBODAA | MBODAU | MBODAUV | MBODATV | MBODATVC |
| 12.1.18 | Sales of hay/crop residue/compost/grass/fodder | MHAYI | MHAYC | MHAYT | MHAYCV | MHAYF | MHAYA | MHAYU | MHAYUV | MHAYTV | MHAYTVC |
| 12.1.19 | Sale of animal manure | MMANI | MMANC | MMANT | MMANCV | MMANF | MMANA | MMANU | MMANUV | MMANTV | MMANTVC |
| 12.1.20 | Sale of game meat | MGAMEI | MGAMEC | MGAMET | MGAMECV | MGAMEF | MGAMEA | MGAMEU | MGAMEUV | MGAMETV | MGAMETVC |
| 12.1.21 | Sale of wild nuts/fruits | MWILDI | MWILDC | MWILDT | MWILDCV | MWILDF | MWILDA | MWILDU | MWILDUV | MWILDTV | MWILDTVC |
| 12.1.22 | Mining (salt, gold, sand, clay, etc) | MMINEI | MMINEC | MMINET | MMINECV | MMINEF | MMINEA | MMINEU | MMINEUV | MMINETV | MMINETVC |
| 12.1.23 | Quarrying stones | MSTONEI | MSTONEC | MSTONET | MSTONECV | MSTONEF | MSTONEA | MSTONEU | MSTONEUV | MSTONETV | MSTONETVC |
| 12.1.24 | Brick making | MBRICKI | MBRICKC | MBRICKT | MBRICKCV | MBRICKF | MBRICKA | MBRICKU | MBRICKUV | MBRICKTV | MBRICKTVC |
| 12.1.25 | Processing/milling machine | MGMILLI | MGMILLC | MGMILLT | MGMILLCV | MGMILLF | MGMILLA | MGMILLU | MGMILLUV | MGMILLTV | MGMILLTVC |
| 12.1.26 | Other petty trade | MPETTYI | MPETTYC | MPETTYT | MPETTYCV | MPETTYF | MPETTYA | MPETTYU | MPETTYUV | MPETTYTV | MPETTYTVC |
| 12.1.27 | Other (specify_____) MOTH1SPE | MOTH1I | MOTH1C | MOTH1T | MOTH1CV | MOTH1F | MOTH1A | MOTH1U | MOTH1UV | MOTH1TV | MOTH1TVC |
| 12.1.28 | Other (specify_____) MOTH2SPE | MOTH2I | MOTH2C | MOTH2T | MOTH2CV | MOTH2F | MOTH2A | MOTH2U | MOTH2UV | MOTH2TV | MOTH2TVC |

## Section 2: Savings & Banking

*I am going to ask you about your household’s saving and banking.*

Table 12.2: In the past 12 months, has any member of your household:

|  | **Question** | **Response** | **Var. name** |
| --- | --- | --- | --- |
| 12.2.1 | Held a bank account? **(if no, skip to 12.2.4)** | 1=Yes  0=No 98=Don’t Know | BACCOUNT |
| 12.2.2 | Put money in the bank? | 1=Yes  0=No 98=Don’t Know | BBANKED |
| 12.2.3 | Withdrawn money from the bank? | 1=Yes  0=No98=Don’t Know | BDREW |
|  | Used another form of saving within the household? | 1=Yes  0=No98=Don’t Know | BDSAVH |
| 12.2.4 | Sought a loan? | 1=Yes  0=No98=Don’t Know | BLOAN |
| 12.2.5 | Received a loan? | 1=Yes  0=No98=Don’t Know | BRECLOAN |
| 12.2.6 | From where (most recent if more than one)? | _______________ | BSOURCE |
| 12.2.7 | At what interest rate (most recent if more than one)? | . % | BINTEREST |
| 12.2.8 | Total Loan debt? | birr | BDEBT |
| 12.2.9 | Made a payment for an existing loan? | 1=Yes  0=No98=Don’t Know  2=Household does not have existing loan | BPAID |
| 12.2.10 | Contributed money to a social group such as edir or equb?  (if yes, specify type of group___________) BCONTRIBUSPE | 1=Yes  0=No 98=Don’t Know | BCONTRIBU |
| 12.2.11 | Received money from a social group such as edir or equb?  (If yes, specify type of group__________) BRECEIVSPE | 1=Yes  0=No 98=Don’t Know | BRECEIV |

##

## Section 3: Other Expenditure

Table 12.3

|  | Over the past one month, did your household purchase or pay for any [ITEM]?  YES…1  NO…0 ►NEXT ITEM  98=Don’t Know►NEXT ITEM | | How much did your household pay in total?  BIRR |
| --- | --- | --- | --- |
| 1 | Matches | HEMATCH | HEMATCHB |
| 2 | Batteries | HEBATT | HEBATTB |
| 3 | Candles (tua’af), incense | HECANDL | HECANDLB |
| 4 | Laundry soap/OMO/endod/besana leaves | HESOAPL | HESOAPLB |
| 5 | Hand soap | HESOAPH | HESOAPHB |
| 6 | Other personal care goods (incl. sendel, matent,) | HEPERSCR | HEPERSCRB |
| 7 | Charcoal | HECHARC | HECHARCB |
| 8 | Firewood | HEFIREWD | HEFIREWDB |
| 9 | Kerosene | HEKEROS | HEKEROSB |
| 10 | Cigarettes, tobacco, suret, gaya | HETOBAC | HETOBACB |
| 11 | Transport | HETRANSP | HETRANSPB |

Table 12.3b

|  | Over the past 12 months, did your household purchase or pay for any [ITEM]?  YES…1  NO…0 ►NEXT ITEM  98=Don’t Know►NEXT ITEM | | How much did your household pay in total?  BIRR |
| --- | --- | --- | --- |
| 1 | Clothes/shoes/fabric for MEN | HECLOTHM | HECLOTHMB |
| 2 | Clothes/shoes/fabric for WOMEN | HECLOTHW | HECLOTHWB |
| 3 | Clothes/shoes/fabric for BOYS | HECLOTHB | HECLOTHBB |
| 4 | Clothes/shoes/fabric for GIRLS | HECLOTHG | HECLOTHGB |
| 5 | Kitchen equipment (cooking pots, etc.) | HEKITCH | HEKITCHB |
| 6 | Linens (sheets, towels,blankets) | HELINEN | HELINENB |
| 7 | Furniture | HEFURN | HEFURNB |
| 8 | Lamp/torch | HELAMP | HELAMPB |
| 9 | Ceremonial expenses | HECEREM | HECEREMB |
| 10 | Contributions to IDDIR | HEIDDIR | HEIDDIRB |
| 11 | Donations to the church | HECHURCH | HECHURCHB |
| 12 | School Fees | HESCHO | HESCHOB |
| 13 | Health Care Costs | HEHELYR | HEHELYRB |
