# Supplementary material for: Nutrition-specific and nutrition-sensitive factors associated with mid-upper arm circumference as a measure of nutritional status in pregnant Ethiopian women: Implications for programming in the first 1000 days
Source: PLoS One. 2019 Mar 26;14(3):e0214358. doi: 10.1371/journal.pone.0214358 (PMC6435172; doi:10.1371/journal.pone.0214358)
Supplement: S2 File — English questionnaires used in the study for data collection. (ZIP) [file pone.0214358.s002.zip › IW TP1 Questionnaire 7.14.16.docx]

USAID – ENGINE Birth Cohort Study Household Questionnaires

Index Woman Interview

*Time Point 1 – Prenatal 1*

Table of Contents

Module 1: Household Information& Characteristics 3

Section 1: Interview Information 3

Section 2: Household Information 4

Section 3: Literacy/Numeracy for Index Woman 5

Section 4: Socioeconomic Characteristics 5

Module 2: Water, Hygiene and Sanitation 6

Section 1: Water Source & Use 6

Module 14: Psychosocial Stress Measurement Scales 8

Section 1: List of Threatening Experiences 8

Section 3: Patient Health Questionnaire (PHQ)-9 and treatment 10

Module 3: Index Woman Diet & Food Security 12

Section 1a: Index Woman Diet – 24 Hour Recall 12

Section 2: Months of Adequate Household Food Provisioning 15

Section 3: Household Food Insecurity Access Scale 16

Section 4: Food Taboos during Pregnancy & Lactation 18

Section 5: Chat Consumption 20

Module 14: Psychosocial Stress Measurement Scales 21

Section 6: Maternity Social Support Scale (MSSS) 21

Section 7: HITS Tool for Intimate Partner Violence Screening 21

Module 4: Index Woman Health Status & Pregnancy 22

Section 1: Health Status Assessment 22

Section 3: Former Pregnancies 23

Section 4: Current Pregnancy 24

Module 14: Psychosocial Stress Measurement Scales 25

Section 4: Six-Item Version of State Trait Anxiety Scale 25

Section 5: Pregnancy Related Anxiety 26

Module 15: Nutrition Knowledge Assessment 27

Module 14: Psychosocial Stress Measurement Scales 28

Section 2: Perceived Stress Scale 28

Module 9: Gender and Decision Making 29

Section 1: Access, ownership, and control of agricultural production 29

Section 2: Access, ownership, and control of durable goods 30

Section 3: Gender and Time Allocation 31

Module 10: Social Participation and Access to Information 32

Section 4: Program Exposure & Uptake: MHCN and ENGINE activities 33

Section 5: Service Delivery and Level of Satisfaction 37

Module 12 – Income & Expenditure 38

Section 1: Other Household Income Sources 38

Section 3: Other Expenditure 41

Section 4: Food Expenditure 42

Module 13: Laboratory Tests and Anthropometric Measurements 45

Section 1: Malaria Testing – Index Woman 45

Section 2: Index Woman Anthropometry 45

Section 5 – Hemocue Measurements (Index Woman) 46

# Module 1: Household Information& Characteristics

## Section 1: Interview Information

| **Number** | **Question** | **Response** | **Variable name** |
| --- | --- | --- | --- |
| 1.1.1 | Date of interview (dd/mm/yyyy) | // | HDATEINT |
| 1.1.2 | Data Time Point  (1-7) |  | HDTIMEPT |
| 1.1.3 | Household ID |  | HHID |
| 1.1.4 | Woreda | *Drop list: Woliso, Goma, Tiro Afeta* | HWOREDA |
| 1.1.5 | Kebele | *Text* | HKEBELE |
| 1.1.6 | Gote / Gere | *Text* | HGOTE |
| 1.1.7 | GPS | “Get GPS Coordinates” (button) | HHGPS |
| 1.1.8 | Interviewer’s ID 1 |  | HID1 |
| 1.1.9 | Interviewer’s ID 2 |  | HID2 |
| 1.1.10 | Supervisor’s ID |  | HSID |
| 1.1.11 | Outcome of Interview | 1. Completed  2. Incomplete  3. Absent  4. Refused  5. Could not locate | HINTOUT |

## Section 2: Household Information

I am going to start by asking you questions about yourself and your household.

*To the enumerator:* **Start with the index woman first***, then the head of household, and any other household members*

| **Full Name** (First, Father’s, Grandfather’s) | **Relation to HH Head**  1. HH Head  2. Father  3. Mother  4. Wife  5. Child  6. Mother-in-law  7. Father-in-law  8. 2^nd^ wife  9. 3^rd^ wife  10. 4^th^ wife  11. Grandchild  12. Grandparent  13. Sibling  14. Uncle  15. Aunt  16. Child of other adult  17. Other adult | **Does the person stay at home at least 3 days out of the week and eat from the family pot?**  0. No  1. Yes  98. Don’t know | **Sex**  0. Male  1. Female | **Age**  *Enter months if less than 2 years of age* | | **Marital Status**  1. Married, monogamous  2. Married, polygamous  3.Cohabiting  4. Single 5.Widowed  6.Divorced 7.Separated  8. N/A | **Years of Completed Schooling**  *Integer*  98 = don’t know | **Religion**  1. Orthodox  2. Catholic  3. Protestant  4. Muslim  5. Traditional  6. Pagan  7. Other | **Pregnant?**  0. No  1. Yes  98. Don’t know  *Only appears if female and over 12 yrs old* |
| --- | --- | --- | --- | --- | --- | --- | --- | --- | --- |
|  |  |  |  | **Years** | **Months** |  |  |  |  |
| HNAME | HRELAHD | HSTAYHOME | HSEX | HAGEYRS | HAGEMOS | HMARTS | HEDUC | HRELIGION | HPREGNANT |
|  |  |  |  |  | |  |  |  |  |
|  |  |  |  |  | |  |  |  |  |

## Section 3: Literacy/Numeracy for Index Woman

|  | **Question** | **Response** | **Variable name** |
| --- | --- | --- | --- |
| 1.3.1 | Can the Index Woman write her name in local language? *Please test* | 1=Yes  0=No  98 = Not tested | HIWWRITE |
| 1.3.2 | Can the Index Woman read the following sentences?  *Picture of local text* | 1=Yes  0=No  98 = Not tested | HIWREAD |
| 1.3.3 | Can the female respondent correctly answer the following numeracy test?  *"If you sell eggs for 30 Birr and chicks for 50 Birr, how many Birr do you have?"* | 1=Yes  0=No  98 = Not tested | HIWNUM |

##

## Section 4: Socioeconomic Characteristics

I am going to ask you questions about the type of house you live in.

*(To the RA: confirm responses with your observation.)*

|  | **Question** | **Responses** | **Variable name** | **Did you confirm?**  0. No  1. Yes |
| --- | --- | --- | --- | --- |
| 1.4.1 | Main type of walls | 1. Wood and mud 2. Mud bricks or burnt bricks 3. Concrete blocks 4. Wood 5. Grass/bamboo 6. Other (specify ________) | HWALL  HWALLSPE | HWALLCF |
| 1.4.2 | Main type of roof | 1. Grass or leaf thatched  2. Corrugated iron sheets  3. Tiles  4. Other (specify _______) | HROOF  HROOFSPE | HROOFCF |
| 1.4.3 | Main type of floor | 1.Mud or dirt  2. Brick/stones/cement  3. Tiles  4. Other (specify*______________*) | HFLOOR  HFLOORSPE | HFLOORCF |
| 1.4.4 | Type of toilet used by the household most of the time | 1. None/bush/garden  2.Unimproved pit latrine  3. Improved pit latrine  4. Flush toilet  5. Community owned latrine  6. Other (specify ______________) | HTOILET  HTOILSPE | HTOILETCF |
| 1.4.5 | Does your household have running water? | 1=Yes  0=No | HRWATER | HRWATERCF |
| 1.4.6 | Does your household have grid electricity? | 1=Yes  0=No | HELECT | HELECTCF |
| 1.4.7 | What is the most common cooking fuel used in this household? | 1. Wood  2. Charcoal  3. Gas or biogas  4. Electricity  5. Kerosene/Paraffin  6. Cows dung (kubet)  7. Other (specify__________) | HFUEL  HFUELSPE |  |
| 1.4.8 | What is your household’s main source of fuel or energy for lighting? | - - 1. Electricity     2. Solar     3. Gas     4. Lantern or fanos     5. Kuraz     6. Open firewood place     7. Other (specify__________) | HLIGHT  HLIGHTSPE |  |

#

# Module 2: Water, Hygiene and Sanitation

## Section 1: Water Source & Use

|  | **Questions** | **Responses** | **Var. name** |
| --- | --- | --- | --- |
| 2.1.1 | What is your household’s most commonly used source of water? | 1. Piped water  2. Public tap  3. Tube well or borehole  4. Protected well or spring  5. Unprotected well or spring  6. Rainwater  7. River or pond  8. Bottled  9. Other (specify_______)  98. Don’t know | WSOURCE  WSOURSPE |
| 2.1.2 | What is your household’s most commonly used source of other household water (non agri)? | 1. Piped water  2. Public tap  3. Tube well or borehole  4. Protected well or spring  5. Unprotected well or spring  6. Rainwater  7. River or pond  8. Bottled  9. Other (specify_______)  98. Don’t know | WSRCOTH  WSROTHSPE |
| 2.1.3 | What is the distance from your household to this source of water (one-way)? *If two sources, refer to the furthest distance* | __________ kilometers  *If the source of water is in the compound, put 0*  98 = Don’t know | WDISTANC |
| 2.1.4 | How much time does it take to bring water (one round trip, including waiting time, by usual means) from the furthest source? | _______ minutes  98 = don’t know | WTIME |
| 2.1.5 | Does the household do any rainwater harvesting?  *For domestic use* | 1=Yes  0=No  98=Don’t know | WRAIN |
| 2.1.6 | Do you do anything to your household water before drinking it?  *Select all that apply* | 1. Do nothing  2. Boiling  3. Use traditional herbs  4. Use chemicals (water guard, liquid, Wuha Agar/Bishangari)  5. Filter/sieve  6. Decant  7. Other (specify___________)  98. Don’t know | WTREAT  WTREATSPE |
| 2.1.7 | Do you store drinking water separately from your other household water? | 1=Yes  0=No  98=Don’t know | WWATSTR |
| 2.1.8 | Where do you store your household drinking water? | 1. Traditional pot with cover  2. Traditional pot without cover  3. Plastic jerry can with cover  4. Plastic jerry can without cover  5. Other (specify__________)  98. Don’t know | WSTORE  WSTORESPE |
| 2.1.9 | How much water does your household usually use in one day? | ______ liters  98 = Don’t know | WJCANS |

**Section 2: Sanitation & Hygiene**

| **How do you dispose of household rubbish?**  *Do not read the responses. Allow respondent to answer, then fill each item below.* | | | | |
| --- | --- | --- | --- | --- |
| 2.3.1 | Garbage pit | 1=Yes  0=No | | WPIT |
| 2.3.2 | Discard in garden | 1=Yes  0=No | | WGARDEN |
| 2.3.3 | Discard in bush | 1=Yes  0=No | | WBUSH |
| 2.3.4 | Open burning | 1=Yes  0=No | | WBURN |
| 2.3.5 | Other  (Specify_______) | 1=Yes  0=No | | WOTHER  WOTHERSPE |
| 2.3.6 | How do you most commonly store prepared food?  *Do not read the responses. Allow respondent to answer, then fill in response appropriately* | 1. Uncovered container or plate  2. Covered container or plate  3. On fire or hot ash  4. Other (specify_____)  98. Don’t know | | WFDSTORE  WFDSTORESPE |
| 2.3.7 | Where do you most commonly store clean dishes? | - 1. Shelf   2. Floor   3. Covered container   4. N/A   5. Other (specify _______)   98. Don’t know | | WDISHSTOR  WDISHSOTH |
| **Under what circumstances do you wash your hands?**  *Do not read the responses below. Allow respondent to answer, then fill each item below* | | | | |
| 2.3.8 | Not at all | | 1=Yes  0=No | WNIL |
| 2.3.9 | When dirt is visible | | 1=Yes  0=No | WDIRT |
| 2.3.10 | After toilet use/defecation/urination | | 1=Yes  0=No | WTOILETUSE |
| 2.3.11 | After cleaning child following defecation | | 1=Yes  0=No | WCLEANCHILD |
| 2.3.12 | Before preparing the food | | 1=Yes  0=No | WFOOD |
| 2.3.13 | Before serving a meal | | 1=Yes  0=No | WMEAL |
| 2.3.14 | Before eating | | 1=Yes  0=No | WEAT |
| 2.3.15 | Before feeding a child | | 1=Yes  0=No | WFEEDBABY |
| 2.3.16 | When I am reminded to do so | | 1=Yes  0=No | WREMIND |

# Module 14: Psychosocial Stress Measurement Scales

## Section 1: List of Threatening Experiences

NOTE: module moved between versions 2-3. This is the location of this section in V3 of the survey.

Which of the following major threatening events have you experienced during the previous year? Please indicate whether it has happened since you became pregnant.

|  | **Question** | **Response** | **Variable name** |
| --- | --- | --- | --- |
| 14.1.1 | (In the last 12 months) have you yourself suffered a serious illness, injury or an assault? | 1. Yes  0. No  98. Don't Know  99. Refused | LEILL |
| 14.1.2 | *If yes to 14.1.1 then ask:*  Did this happen since you became pregnant? | 1. Yes  0. No  98. Don't Know  99. Refused | LEILLPR |
| 14.1.3 | (In the past 12 months) has a serious illness, injury or assault happened to a close relative? | 1. Yes  0. No  98. Don't Know  99. Refused | LEILREL |
| 14.1.4 | *If yes to 14.1.3 then ask:*  Did this happen since you became pregnant? | 1. Yes  0. No  98. Don't Know  99. Refused | LEILRPR |
| 14.1.5 | (In the past 12 months) has your spouse, parent or child died? | 1. Yes  0. No  98. Don't Know  99. Refused | LEBER |
| 14.1.6 | *If yes to 14.1.5 then ask:*  Did this happen since you became pregnant? | 1. Yes  0. No  98. Don't Know  99. Refused | LEBERPR |
| 14.1.7 | (In the past 12 months) has a close family friend or another relative died? | 1. Yes  0. No  98. Don't Know  99. Refused | LEBERF |
| 14.1.8 | *If yes to 14.1.7 then ask:*  Did this happen since you became pregnant? | 1. Yes  0. No  98. Don't Know  99. Refused | LEBERFPR |
| 14.1.9 | (In the past 12 months) have you had a separation due to marital difficulties? | 1. Yes  0. No  98. Don't Know  99. Refused | LEMAR |
| 14.1.10 | *If yes to 14.1.9 then ask:*  Did this happen since you became pregnant? | 1. Yes  0. No  98. Don't Know  99. Refused | LEMARPR |
| 14.1.11 | (In the past 12 months) have you broken off a steady friendship or relationship? | 1. Yes  0. No  98. Don't Know  99. Refused | LEREL |
| 14.1.12 | *If yes to 14.1.11 then ask:*  Did this happen since you became pregnant? | 1. Yes  0. No  98. Don't Know  99. Refused | LERELPR |
| 14.1.13 | (In the past 12 months) have you had a serious problem with a close friend, neighbor or relative? | 1. Yes  0. No  98. Don't Know  99. Refused | LEFRIE |
| 14.1.14 | *If yes to 14.1.13 then ask:*  Did this happen since you became pregnant? | 1. Yes  0. No  98. Don't Know  99. Refused | LEFRIEPR |
| 14.1.15 | (In the past 12 months) have you had a major financial crisis (serious money worries)? | 1. Yes  0. No  98. Don't Know  99. Refused | LEFIN |
| 14.1.16 | *If yes to 14.1.15 then ask:*  Did this happen since you became pregnant? | 1. Yes  0. No  98. Don't Know  99. Refused | LEFINPR |
| 14.1.17 | (In the past 12 months) have you lost or had anything stolen which mattered a lot to you? | 1. Yes  0. No  98. Don't Know  99. Refused | LETHEF |
| 14.1.18 | *If yes to 14.1.17 then ask:*  Did this happen since you became pregnant? | 1. Yes  0. No  98. Don't Know  99. Refused | LETHEFPR |
| 14.1.19 | (In the past 12 months) have you had any problems with the police or courts? | 1. Yes  0. No  98. Don't Know  99. Refused | LEPOL |
| 14.1.20 | *If yes to 14.1.19 then ask:*  Did this happen since you became pregnant? | 1. Yes  0. No  98. Don't Know  99. Refused | LEPOLPR |
| 14.1.21 | (In the past 12 months) has your husband been unemployed? | 1. Yes  0. No  98. Don't Know  99. Refused | LEUNEMP |
| 14.1.22 | *If yes to 14.1.21 then ask:*  Did this happen since you became pregnant? | 1. Yes  0. No  98. Don't Know  99. Refused | LEUNEMPR |
| 14.1.23 | (In the past 12 months) has anything else seriously upset you? | 1. Yes  0. No  98. Don't Know  99. Refused | LEOTH |
| 14.1.24 | *If yes to 14.1.23 then ask:*  Did this happen since you became pregnant? | 1. Yes  0. No  98. Don't Know  99. Refused | LEOTHPR |

## Section 3: Patient Health Questionnaire (PHQ)-9 and treatment

NOTE: module moved between versions 2-3. This is the location of this section in V3 of the survey.

Over the last two weeks, how often have you been bothered by any of the following problems?

|  | **Question** | **Response** | **Variable name** |
| --- | --- | --- | --- |
| 14.3.1 | Over the last two weeks, how often have you bothered by little interest or pleasure in doing things? | 0. Not at all  1. Several days  2. More than half the days  3. Nearly every day  98. Don’t know | PHQ1A |
| 14.3.2 | Over the last two weeks, how often have you bothered by feeling down, depressed, or hopeless | 0. Not at all  1. Several days  2. More than half the days  3. Nearly every day  98. Don’t know | PHQ2A |
| 14.3.3 | Over the last two weeks, how often have you bothered by trouble falling/staying asleep, sleeping too much? | 0. Not at all  1. Several days  2. More than half the days  3. Nearly every day  98. Don’t know | PHQ3A |
| 14.3.4 | Over the last two weeks, how often have you bothered by feeling tired or having little energy? | 0. Not at all  1. Several days  2. More than half the days  3. Nearly every day  98. Don’t know | PHQ4A |
| 14.3.5 | Over the last two weeks, how often have you bothered by poor appetite or overeating? | 0. Not at all  1. Several days  2. More than half the days  3. Nearly every day  98. Don’t know | PHQ5A |
| 14.3.6 | Over the last two weeks, how often have you bothered by feeling bad about yourself – or that you are ⁮a failure or have let yourself or your family down? | 0. Not at all  1. Several days  2. More than half the days  3. Nearly every day  98. Don’t know | PHQ6A |
| 14.3.7 | Over the last two weeks, how often have you bothered by trouble concentrating on things, such as reading the newspaper or watching television? | 0. Not at all  1. Several days  2. More than half the days  3. Nearly every day  98. Don’t know | PHQ7A |
| 14.3.8 | Over the last two weeks, how often have you bothered by moving or speaking so slowly that other people could have noticed – or the opposite – being so fidgety or restless that you have been moving around a lot more than usual? | 0. Not at all  1. Several days  2. More than half the days  3. Nearly every day  98. Don’t know | PHQ8A |
| 14.3.9 | Over the last two weeks, how often have you bothered by thoughts that you would be better off dead or of hurting yourself in some way? | 0. Not at all  1. Several days  2. More than half the days  3. Nearly every day  98. Don’t know | PHQ9A |
| Total for PHQ1-PHQ9 *calculated* | | | |
| 14.3.10 | Apart from these past two weeks, during the past 12 months, did you have other episodes of two weeks or more when you felt depressed or uninterested in most things, and had most of the problems we just talked about? | 0. No  1. Yes | PHQ10 |
| 14.3.11 | If said “yes” to any of the problems, how difficult have these problems made it for you to do your work, take care of things at home or get along with other people? | 0. Not difficult at all  1. Somewhat difficult  2. Very difficult  3. Extremely difficult | PHQ11 |

#

# Module 3: Index Woman Diet & Food Security

NOTE: This section (3.1a) only appears in version 1 &2 at TP1. Enumerators administering version 3 or 4 did not see this section.

## Section 1a: Index Woman Diet – 24 Hour Recall

Now I am going to ask you about what you ate and drank yesterday, from the time you woke up to the time you slept. If there were foods that were shared please tell me who else shared these foods with you.

|  | **Question** | **Response** | **Variable name** |
| --- | --- | --- | --- |
| 3.1.1 | Day of recall | 1. Sunday  2. Monday  3. Tuesday  4. Wednesday  5. Thursday  6. Friday  7. Saturday | RECALLDAY |
| 3.1.2 | Are you fasting today? | 0. No  1. Yes  3. N/A  99. Refused | IWFASTING |
| 3.1.3 | Are you fasting from **all** foods for some of the day? | 0. No  1.Yes, until noon  2. Yes, until 3pm  3.Yes, until sundown  99. Refused | IWFASTTIME |
| 3.1.4 | Does this represent a normal eating day for you? | 0. No  1.Yes  99. Refused | IWNORMEAT |
| 3.1.5 | If no, explain:  *Probe about sickness, festival, holiday, etc.* | *Text* | IWNORMEXP |

| **Food or Drink**  *Text* | **Time of day**  *Select one* | **Description**  *Text* | **Menu or single food** | **Total Amount Prepared?**  *Decimal* | **Food Measurement Unit**  V1 entered as text; V2-4 selected from preset options  *Select one* | **Number of Portions Served?**  *Decimal* | **Was this shared?** | **If shared, how many of the following groups shared:**  *Integer* | **Was any left over?** | **If yes, how many units?**  *Decimal* |
| --- | --- | --- | --- | --- | --- | --- | --- | --- | --- | --- |
|  | 1. Morning (sunrise yesterday to ~5:30)  2. Afternoon (~5:30-sunset),  3. Evening/ night time (sunset to just before sunrise this morning)  4. Don’t remember |  | 1. Menu  2.Single  3. Don’t know |  | **A1**: Injera  **A2**: Injera  **A3**: Injera  **B1**: Round  **B2**: Round  **B3**: Round  **C1**: Triangle  **C2**: Triangle  **C3**: Triangle  **D1**: Square  **D2**: Square  **D3**: Square  **E**: 1 Birr  **F1**: Ladle  **F2**: Ladle  **F3**: Ladle  **G1**: Metal pots  **G2**: Metal pots  **G3**: Metal pots  **G4**: Metal pots  **H0**:Glasses  **H1**:Glasses  **H2**:Glasses  **H3**:Glasses  **I1**: Spoons  **I2**: Spoons  **I3**: Spoons  **Pcs SM:** Pieces  **Pcs MD:** Pieces  **Pcs LG:** Pieces | 98 = Don’t know | 0. No  1. Yes  98. Don’t know | **IWDMF12mo**  M/F: <12mo  **IWDMF1235mo**  M/F: 12-35mo  **IWDMF36mo6yr**  M/F: 36mo-6yr  **IWDMF79yr**  M/F: 7-9yrs  **IWDM1012yr**  M: 10-12yr  **IWDF1012yr**  F: 10-12yr  **IWDM1315yr**  M: 13-15yrs  **IWDF1315yr**  F: 13-15yrs  **IWDM1619yr**  M: 16-19yrs  **IWDF1619yr**  F:16-19yrs  **IWDM20yr**  M: ≥20yrs  **IWDF20yr**  F: ≥20yrs | 0. No  1. Yes  98. Don’t know | 98 = Don’t know |
| **IWFODR** | **IWDTIMEDAY** | **IWDDESC** | **IWDMS** | **IWDPREPARED** | **IWDPORSIZEPRE** | **IWDNUMPOR** | **IWDSHARED** |  | **IWDLEFTOVER** | **IDWNUMLEFTOVER** |

| After all foods are entered, **IF Column 4 (MENU OR SINGLE FOOD) = 1 (Menu**,) after listing all food, go back and ask the ingredients | | | | | |
| --- | --- | --- | --- | --- | --- |
| **Food or Drink** | **Ingredient**  *Text* | **Description**  *Text* | **Measurement**  V1 entered as text; V2-4 selected from preset options  **B1**: Round  **B2**: Round  **B3**: Round  **C1**: Triangle  **C2**: Triangle  **C3**: Triangle  **D1**: Square  **D2**: Square  **D3**: Square  **E**: 1 Birr  **F1**: Ladle  **F2**: Ladle  **F3**: Ladle  **G1**: Clay pots  **G2**: Clay pots  **G3**: Clay pots  **H0**: Glasses  **H1** : Glasses  **H2**: Glasses  **H3**: Glasses  **I1**: Spoons  **I2**: Spoons  **I3**: Spoons  **Pcs SM:** Pieces  **Pcs MD:** Pieces  **Pcs LG:** Pieces | **Amount**  *Decimal* | **Cooked or raw?**  1. Cooked  2. Raw  98. Don’t know |
| IWFODR | IWDINGREDIENT | IWDINGREDIENTDESC | IWDMODEL | IWDAMOUNT | IWDCORA |
|  |  |  |  |  |  |
|  |  |  |  |  |  |
|  |  |  |  |  |  |

## Section 2: Months of Adequate Household Food Provisioning

Now I would like to ask you about your household’s food supply during different months of the year. When responding to these questions, please think back over the last 12 months, starting with the current month until the same time last year. ***(Asked to the Index Woman)***

|  | **Question** | **Responses** | **Variable name** |
| --- | --- | --- | --- |
|  | Were there month(s), in the past 12 months, in which you did not have enough food to meet your family’s needs? | 1=Yes  0=No  98=Don’t Know | WSGOOD |

If yes, which were the months in the past 12 months during which you did not have enough food to meet your family’s needs?

*To the RA: This includes any kind of food from any source, such as own production, purchase or exchange, food aid, or borrowing. Do not read the list of months aloud. Use a seasonal calendar if needed to help respondent remember the different months. Probe to make sure the respondent has thought about the entire past 12 months.*

|  | **Month** | **Response** | **Variable name** |
| --- | --- | --- | --- |
|  | January | 1=Yes  0=No 98=Don’t Know | WSJAN |
|  | February | 1=Yes  0=No 98=Don’t Know | WSFEB |
|  | March | 1=Yes  0=No 98=Don’t Know | WSMAR |
|  | April | 1=Yes  0=No 98=Don’t Know | WSAPR |
|  | May | 1=Yes  0=No 98=Don’t Know | WSMAY |
|  | June | 1=Yes  0=No 98=Don’t Know | WSJUN |
|  | July | 1=Yes  0=No 98=Don’t Know | WSJUL |
|  | August | 1=Yes  0=No 98=Don’t Know | WSAUG |
|  | September | 1=Yes  0=No 98=Don’t Know | WSEPT |
|  | October | 1=Yes  0=No 98=Don’t Know | WSOCT |
|  | November | 1=Yes  0=No 98=Don’t Know | WSNOV |
|  | December | 1=Yes  0=No 98=Don’t Know | WSDEC |

## Section 3: Household Food Insecurity Access Scale

Now I am going to ask you questions about your household’s food supply over the past four weeks.

*Asked to the Index Woman*

|  | **Question** | **Response** | **Variable name** |
| --- | --- | --- | --- |
| 3.3.1 | In the past four weeks, did you worry that your household would not have enough food? | 1=Yes  0=No  98=Don’t Know | WSWORRY |
| 3.3.2 | If yes, how often did this happen? | 1. Rarely (once or twice in the past four weeks)  2. Sometimes (3 to 10 times in the past four weeks)  3. Often (more than 10 times in the past four weeks) | WSWORRYFRQ |
| 3.3.3 | In the past four weeks, were you or any household member not able to eat the kinds of foods you preferred because of a lack of resources? | 1=Yes  0=No  98=Don’t Know | WSKIND |
| 3.3.4 | If yes, how often did this happen? | 1. Rarely (once or twice in the past four weeks)  2. Sometimes (3 to 10 times in the past four weeks)  3. Often (more than 10 times in the past four weeks) | WSKINDFRQ |
| 3.3.5 | In the past four weeks, did you or any household member have to eat a limited variety of foods due to a lack of resources? | 1=Yes  0=No  98=Don’t Know | WSLIMITED |
| 3.3.6 | If yes, how often did this happen? | 1. Rarely (once or twice in the past four weeks)  2. Sometimes (3 to 10 times in the past four weeks)  3. Often (more than 10 times in the past four weeks) | WSLIMITEDFRQ |
| 3.3.7 | In the past four weeks, did you or any household member have to eat some foods that you really did not want to eat because of a lack of resources to obtain other types of food? | 1=Yes  0=No  98=Don’t Know | WSDISLIKE |
| 3.3.8 | If yes, how often did this happen? | 1. Rarely (once or twice in the past four weeks)  2. Sometimes (3 to 10 times in the past four weeks)  3. Often (more than 10 times in the past four weeks) | WSDISLIKEFRQ |
| 3.3.9 | In the past four weeks, did you or any household member have to eat a smaller meal than you felt you needed because there was not enough food? | 1=Yes  0=No  98=Don’t Know | WSMALL |
| 3.3.10 | If yes, how often did this happen? | 1. Rarely (once or twice in the past four weeks)  2. Sometimes (3 to 10 times in the past four weeks)  3. Often (more than 10 times in the past four weeks) | WSMALLFRQ |
| 3.3.11 | In the past four weeks, did you or any household member have to eat fewer meals in a day because there was not enough food? | 1=Yes  0=No  98=Don’t Know | WSFEW |
| 3.3.12 | If yes, how often did this happen? | 1. Rarely (once or twice in the past four weeks)  2. Sometimes (3 to 10 times in the past four weeks)  3. Often (more than 10 times in the past four weeks) | WSFEWFRQ |
| 3.3.13 | In the past four weeks, was there ever no food to eat of any kind in your household because of lack of resources to get food? | 1=Yes  0=No  98=Don’t Know | WSNOFOOD |
| 3.3.14 | If yes, how often did this happen? | 1. Rarely (once or twice in the past four weeks)  2. Sometimes (3 to 10 times in the past four weeks)  3. Often (more than 10 times in the past four weeks) | WSNOFOODFRQ |
| 3.3.15 | In the past four weeks, did you or any household member go to sleep at night hungry because there was not enough food? | 1=Yes  0=No  98=Don’t Know | WSLEEP |
| 3.3.16 | If yes, how often did this happen? | 1. Rarely (once or twice in the past four weeks)  2. Sometimes (3 to 10 times in the past four weeks)  3. Often (more than 10 times in the past four weeks) | WSLEEPFRQ |
| 3.3.17 | In the past four weeks, did you or any household member go a whole day and night without eating anything because there was not enough food? | 1=Yes  0=No  98=Don’t Know | WSNODAY |
| 3.3.18 | If yes, how often did this happen? | 1. Rarely (once or twice in the past four weeks)  2. Sometimes (3 to 10 times in the past four weeks)  3. Often (more than 10 times in the past four weeks) | WSNODAYFRQ |

## Section 4: Food Taboos during Pregnancy & Lactation

In your area are there **traditional practices** that prohibit pregnant and breastfeeding women from taking some foods or types of foods?

|  | **Question** | **Response** | **Variable name** |
| --- | --- | --- | --- |
| 3.4.1 | Pregnant | 0. No  1. Yes  98. Don’t know | FDTABOBF |
|  | Breastfeeding | 0. No  1. Yes  98. Don’t know | FDTABOLAC |

If yes, please list the foods or types of food that are prohibited (be specific as possible)

| **Food type**  *Text* | **When prohibited during pregnancy?**   1. Entire Pregnancy 2. Beginning 3. End 4. Middle 5. N/A   98. Don’t know | **Why prohibited?**  1.Fear of difficult labor (large baby)  2. Discoloration of fetus  3. Fear of abortion  4. Other (specify)  98. Don’t know | **Also prohibited during breastfeeding?**  0. No  1. Yes  98. Don’t know | **If yes, why?**  *Text* | **Do you normally eat this food?**  1. Regularly  2. Sometimes  3. Rarely  4. Never  98. Don’t know | **Do you eat this food during pregnancy/**  **lactation?**  1. Regularly  2. Sometimes  3. Rarely  4. Never  98. Don’t know | **Do you crave this food during pregnancy?**  0. No  1. Yes  98. Don’t know |
| --- | --- | --- | --- | --- | --- | --- | --- |
| FTPTYPD | FTPTYPD | FTPTYPW  *Specify (text):* FTPTYPWS | FTPTYPBF | FTPTYPBFS | FTPTYPNC | FTPTYPPC | FTPTYPCR |

|  | **Question** | **Response** | **Variable** |
| --- | --- | --- | --- |
| 3.4.3 | Are any other foods prohibited during breastfeeding not listed already? | *Text* | FTBF1  FTBF2  FTBF3 |
| 3.4.4 | In your area are there traditional practices that encourage pregnant or breastfeeding women to eat more of certain foods? | 0. No  1. Yes  98. Don’t know | FDTABMORE |

*If yes to 3.4.4, please list the foods or types of food that are encouraged:*

| **Food type**  *Text* | **When encouraged?**   1. Entire pregnancy 2. Beginning 3. End 4. Breastfeeding | **Why encouraged?**  1. Facilitates delivery  2. Prevents stillbirth  3. Prevents big baby  4. Health baby  5. Improves maternal health  6. Other | **Normally consumed?**  0. No  1. Yes |
| --- | --- | --- | --- |
| FTETYPE | FTPTYPD | FTETYPW  *Specify (text):*  FTETYPWS | FTETYPNC |

|  | **Question** | **Response** | **Variable name** |
| --- | --- | --- | --- |
| 3.4.6 | How has the amount of your food you eat changed after you became pregnant? | 1. I eat more food than normal 2. I eat less food than normal 3. The amount of food I eat has not changed 4. Don’t know | FTAMTCHANG |
| 3.4.7 | How has the variety of foods in your diet changed after becoming pregnant? | 1. I eat more types of food than normal 2. I eat less types of food than normal 3. I eat the same foods as normal   98. Don’t Know | FTVARCHG |
| 3.4.8  Question only appears in versions 3 & 4 | Do you observe Orthodox fasting practices during pregnancy?  *Select as many as apply* | 1. Yes, I fast from all animal products on fasting days  2. Yes, I fast all food until noon on fasting days  3. Yes, I fast all food until 3pm on fasting days  5. I only fast some of the designated fasting days  6. No, I do not fast at all during my pregnancy  98. Don’t know  99. Not Applicable | FTFASTING |
| 3.4.9  Question only appears in versions 3 & 4 | Do you observe fasting practices during Ramadan while pregnant? | 1. Yes, I fast sunrise to sunset for the full month  2. Yes, but not for the whole month  3. No, I do not fast during Ramadan while pregnant  98. Don’t know  99. Not Applicable | FTOBSERV |

## Section 5: Chat Consumption

|  | **Question** | **Response** | **Variable name** |
| --- | --- | --- | --- |
| 3.5.1 | Do you chew Chat? | 1. No 2. Yes   99. Refused | WCHATCH |
| 3.5.2 | If yes, how often? | ________times per  1. Day  2. Week  3. Month  4. Year | WCHATOFT  WCHATOFTPER |
| 3.5.3 | How much do you chew in one session? | _________ bundles | WCHATQUANT |
| 3.5.4 | Do you purchase from store or chew own production? | 1. Own production only  2. Purchase only  3. Combination  4. Gift  5. Other | WCHATSRC |
| 3.5.5 | Do you clean the chat before chewing? | 1. No 2. Yes   98. Don’t know | WCHATCLEAN |

# Module 14: Psychosocial Stress Measurement Scales

## Section 6: Maternity Social Support Scale (MSSS)

NOTE: module moved between versions 2-3. This is the location of this section in V3 of the survey.

We would like to ask you questions about the support you get from different people. For each of the following statements, please give your opinion as to which shows how you feel about the support you have now.

|  | **Questions** | **5= Always 4 = Most of the time 3 = Some of the times 2 = Rarely**  **1 = Never 98 = Don’t know** | **Var name** |
| --- | --- | --- | --- |
| 14.6.1 | How often do you feel you have good friends who support you? | 1 2 3 4 5 98 | MSSS1 |
| 14.6.2 | How often do you feel your family is always there for you? | 1 2 3 4 5 98 | MSSS2 |
| 14.6.3 | How often do you feel you husband/partner helps you? | 1 2 3 4 5 98 | MSSS3 |
| 14.6.4 | How often do you feel there is conflict with your husband/partner? | 1 2 3 4 5 98 | MSSS4 |
| 14.6.5 | How often do you feel controlled by your husband/partner? | 1 2 3 4 5 98 | MSSS5 |
| 14.6.6 | How often do you feel loved by your husband/partner? | 1 2 3 4 5 98 | MSSS6 |

## Section 7: HITS Tool for Intimate Partner Violence Screening

NOTE: module moved between versions 2-3. This is the location of this section in V3 of the survey.

Please listen to each of the following activities and tell me that best indicates the frequency with which your partner acts in the way depicted.

|  | **Question** | **1= Never 2= Rarely 3=Sometimes 4= Fairly often 5=Frequently 98 = Don’t know** | **Variable name** |
| --- | --- | --- | --- |
| How often does your partner: | | | |
| 14.7.1 | Physically hurt you | 1 2 3 4 5 98 | HITS1 |
| 14.7.2 | Insult or talk down to you | 1 2 3 4 5 98 | HITS2 |
| 14.7.3 | Threaten you with harm | 1 2 3 4 5 98 | HITS3 |
| 14.7.4 | Scream or curse at you | 1 2 3 4 5 98 | HITS4 |

# Module 4: Index Woman Health Status & Pregnancy

## Section 1: Health Status Assessment

I am going to ask you about your health. Please respond to the following questions about you yourself.

|  | **Question** | **Responses** | **Variable name** |
| --- | --- | --- | --- |
| 4.1.1 | In the past two weeks, have you had any illness?  *If no, skip to 4.1.6* | 1=Yes  0=No  98= Don’t know | MANYILL |
| 4.1.2 | If yes, how many days in the past two weeks were you unable to do your daily work because of illness? | ___days  *Allow partial days* | MDAYS |
| Please answer the following three questions regarding the last illness you ever had: | | | |
| 4.1.3 | Did you seek help outside the home? *If no, skip to 4.1.6* | 1=Yes  0=No  98= Don’t know | MTREAT |
| 4.1.4 | If yes, where did you seek help? | 1. Traditional healer  2. Holy water  3. Witchcraft  4. Health extension worker  5. Drug shop or private pharmacy  6. Private clinic  7. NGO health facility  8. Public health facility (HC, Hospital) | MTREATWHERE |
| 4.1.5 | How far was the furthest facility where you sought help? | _____ kilometers  98 = Don’t know | MDISTANCE |
| 4.1.6 | Did you sleep under a bed net last night? | 1=Yes  0=No  2 = Don’t own bed net  98= Don’t know | MNETCGIVER |

## Section 3: Former Pregnancies

|  | **Question** | **Response** | **Variable name** |
| --- | --- | --- | --- |
| 4.3.1 | How many pregnancies have you ever had?  *Do not include current if pregnant. If response is “0”, skip to next section* | ____ pregnancies  98 = Don’t know | MPREGNA |
| 4.3.2 | How many live born children have you ever had? | ______ children  98 = Don’t know | MALIVEBORN |
| 4.3.3 | Have you ever lost a child due to death (including stillbirths)? | 1=Yes  0=No  98= Don’t know | MCHILDIE |
| 4.3.4 | If yes, how many? | ___ children  98 = Don’t know | MNOCHILDIE |
| 4.3.5 | Have you ever lost a child due to miscarriage (spontaneous abortion)? | 1=Yes  0=No  98= Don’t know | MMISCARRY |
| 4.3.6 | If yes, how many? | ________children  98 = Don’t know | MNOMISCARY |
| 4.3.7 | How long ago was your last pregnancy? | ___ months  98 = Don’t know | MLASTPRG |
| 4.3.8 | How many antenatal care visits occurred during your last pregnancy?  *Enter “0” if none* | ______visits  98 = Don’t know | MNOANC |
| 4.3.9 | During that pregnancy, were you given or did you buy any iron tablets?  *Show the tablet* | 1=Yes  0=No  98= Don’t know | MANCIRON |
| 4.3.10 | If yes, during the whole pregnancy, for how many days did you take the tablets? | ____days  98 = Don’t know | MIRONDAYS |
| 4.3.11 | During the last pregnancy, did you receive any drug for intestinal worms? | 1=Yes  0=No  98= Don’t know | MTREATWORM |
| 4.3.12 | During the last pregnancy, were you offered HIV testing? | 1=Yes  0=No  98= Don’t know | MHIVTEST |
| 4.3.13 | During the last pregnancy, did you receive ART treatment? | 1=Yes  0=No  98= Don’t know | MART |
| 4.3.14 | What was the outcome of your last pregnancy?  *Do not read the options, allow the respondent to answer spontaneously* | 1. Live birth  2. Still birth  3. Spontaneous abortion/ miscarriage  4. Induced abortion  98. Don’t know | MPREGOUT |
| 4.3.15 | For the last pregnancy, where did you deliver? | 1. Public or gov’t health facility.  2.Private not-for-profit or NGO health facility  3. Private health facility  4. At traditional birth attendant’s  5. Home (own or other’s)  6. Other (specify__________) | MDELIVER  MDELIVSPE |
| 4.3.16 | During your last pregnancy, did you deliver by Caesarean section? | 1=Yes  0=No  98= Don’t know | MCAESAR |
| 4.3.17 | During your last pregnancy, did you deliver by instruments? | 1=Yes  0=No  98= Don’t know | MFORCEP |
| 4.3.18 | Have you ever been diagnosed with pregnancy induced hypertension? | 1=Yes  0=No  98= Don’t know | MPREGHYPT |

##

## Section 4: Current Pregnancy

|  | **Question** | **Response** | **Variable name** |
| --- | --- | --- | --- |
| 4.4.1 | Are you currently pregnant?  *If yes, skip to 4.4.3* | 1=Yes  0=No  98=Don’t Know | MCURPREG |
| 4.4.2 | If no, what terminated your pregnancy? | 1. Premature birth/stillborn  2. Spontaneous abortion/ miscarriage  3. Induced abortion  4. Don’t know | MPREGTERM |
| 4.4.3 | If yes, for how many weeks have you been pregnant?  *Prompt with date of last menstrual period* | __________ weeks  98=Don’t Know | MPREGWKS |
| 4.4.4 | Have you sought antenatal care during this pregnancy so far? | 1=Yes  0=No  98=Don’t Know | MANCC |
| 4.4.5 | How many antenatal care visits have occurred during this pregnancy *so far*? | ________visits  98 = Don’t know | MNOANCC |
| 4.4.6 | During this pregnancy, were you given or did you buy any iron tablets?  *Show the tablet. If no or don’t know, skip to 4.4.9* | 1=Yes  0=No  98=Don’t Know | MANCIRONC |
| 4.4.7 | If yes, during this pregnancy, for how many days have you taken the tablets so far? | ____days  98 = Don’t know | MIRONDAYSC |
| 4.4.8 | If yes, will you continue taking the tablets through this pregnancy? | 1=Yes  0=No  98=Don’t Know | MIRONRESTDAYS |
| 4.4.9 | During this pregnancy, did you receive any drug for intestinal worms? | 1=Yes  0=No  98=Don’t Know | MTREATWORMC |
| 4.4.10 | During this pregnancy, were you offered HIV testing? | 1=Yes  0=No  98=Don’t Know | MHIVTESTC |
| 4.4.11 | During this pregnancy, did you receive ART treatment? | 1=Yes  0=No  98=Don’t Know | MARTC |

# Module 14: Psychosocial Stress Measurement Scales

## Section 4: Six-Item Version of State Trait Anxiety Scale

I want to know how you feel right now, that is, at this very moment. Please listen to each question as I read and tell me whether or not you fell that feeling at this moment and then how strong that feeling is. Do not spend too much time on any one question to answer but give the answer which seems to describe your present feelings best.

|  | **Question** | 1. Not at all  2. Somewhat  3. Moderately  4. Very much  98. Don’t know | **Variable name** |
| --- | --- | --- | --- |
| 14.4.1 | How strongly do you feel calm right now? | 1 2 3 4 98 | STAS1 |
| 14.4.2 | How strongly are you are tense right now? | 1 2 3 4 98 | STAS2 |
| 14.4.3 | How strongly do you feel upset right now? | 1 2 3 4 98 | STAS3 |
| 14.4.4 | How strongly are you relaxed right now? | 1 2 3 4 98 | STAS4 |
| 14.4.5 | How strongly do you feel content right now? | 1 2 3 4 98 | STAS5 |
| 14.4.6 | How strongly are you worried right now? | 1 2 3 4 98 | STAS6 |

## Section 5: Pregnancy Related Anxiety

Following are some common fears that pregnant women have expressed in the past. No one is expected to have them all. Some women may have none of them. Please answer as honestly as you can. If you're not sure how to rate the intensity of the fear, do not worry about it, just make a quick judgment and tell me what seems about right.

|  | **Question** | 1. Never  2. Almost never  3. Sometimes  4. Fairly often  5. Very often  98. Don’t know | **Variable name** |
| --- | --- | --- | --- |
| 14.5.1 | How often are you worried about the pain of contractions and the pain during delivery? | 1 2 3 4 5 98 | PRAQ1 |
| 14.5.2 | How often are you anxious about the delivery because you have never experienced one before? | 1 2 3 4 5 98 | PRAQ2 |
| 14.5.3 | How often are you worried about not being able to control yourself during labour and fear that I will scream? | 1 2 3 4 5 98 | PRAQ3 |
| 14.5.4 | How often are you afraid that the baby will be mentally handicapped or will suffer from brain damage? | 1 2 3 4 5 98 | PRAQ4 |
| 14.5.5 | How often are you afraid that your baby will be stillborn, or will die during or immediately after delivery? | 1 2 3 4 5 98 | PRAQ5 |
| 14.5.6 | How often are you afraid that your baby will suffer from a physical defect or worry that something will be physically wrong with the baby? | 1 2 3 4 5 98 | PRAQ6 |
| 14.5.7 | How often do you think that your child will be in poor health or will be prone to illnesses? | 1 2 3 4 5 98 | PRAQ7 |
| 14.5.8 | How often are you worried about the fact that you will not regain your figure after delivery? | 1 2 3 4 5 98 | PRAQ8 |
| 14.5.9 | How often are you concerned about your unattractive appearance? | 1 2 3 4 5 98 | PRAQ9 |
| 14.5.10 | How often are you worried about your enormous weight gain? | 1 2 3 4 5 98 | PRAQ10 |
| 14.5.11 | How often are you afraid that you will die during or immediately after delivery? | 1 2 3 4 5 98 | PRAQ11 |
| 14.5.12 | How often do you afraid that you will be in poor health or will be prone to illnesses after delivery? | 1 2 3 4 5 98 | PRAQ12 |

# Module 15: Nutrition Knowledge Assessment

Food supply includes staples, sauces, and any other foods in your diet and the diets of all members of your household.

*To the Enumerator:* *Read each question and if the respondent gives the correct answer, select 0=Correct. If she does not give the correct answer, select 1 = Incorrect.*

|  | **Question** | **Response** | **Variable name** |
| --- | --- | --- | --- |
| 15.1 | Is a meal of injera and shiro every day a good example of a diversified diet?  *ANSWER: No* | 0 = Correct  1 = Incorrect  98 = Don’t know | NHK1 |
| 15.2 | Is eating three times a day during pregnancy an adequate frequency for the proper growth and development of the fetus?  *ANSWER: No* | 0 = Correct  1 = Incorrect  98 = Don’t know | NHK2 |
| 15.3 | Which is better: to begin breastfeeding within 12 hours after giving birth or within 1 hour after giving birth?  *ANSWER: Within 1 hour* | 0 = Correct  1 = Incorrect  98 = Don’t know | NHK3 |
| 15.4 | Does breast milk alone have all of the necessary nutrients and water that a baby needs until the end of the first year of life?  *ANSWER: No* | 0 = Correct  1 = Incorrect  98 = Don’t know | NHK4 |
| 15.5 | At what age should a mother begin giving foods other than breast milk to her babies?  *Answer: Six months* | 0 = Correct  1 = Incorrect  98 = Don’t know | NHK5 |

# Module 14: Psychosocial Stress Measurement Scales

## Section 2: Perceived Stress Scale

NOTE: This section (14.2) was moved between versions 2-3. This is where Module 14, Section 2 is placed in versions 3 &4.

The questions in this scale ask you about your feelings and thoughts during the last month. In each case, you will be asked to indicate by circling how often you felt or thought a certain way.

|  | **Question** | **Response**  0. Never  1. Almost Never  2. Sometimes  3. Fairly Often  4. Very Often  98. Don’t know | **Variable name** |
| --- | --- | --- | --- |
| 14.2.1 | In the last month, how often have you been upset of something that happened unexpectedly? | 0 1 2 3 4 98 | PSS1 |
| 14.2.2 | In the last month, how often have you felt that you were unable to control the important things in your life? | 0 1 2 3 4 98 | PSS2 |
| 14.2.3 | In the last month, how often have you felt nervous and stressed? | 0 1 2 3 4 98 | PSS3 |
| 14.2.4 | In the last month, how often have you felt confident about your ability to handle your personal problems? | 0 1 2 3 4 98 | PSS4 |
| 14.2.5 | In the last month, how often have you felt that things were going your way? | 0 1 2 3 4 98 | PSS5 |
| 14.2.6 | In the last month, how often have you found that you could not cope with all the things that you had to do? | 0 1 2 3 4 98 | PSS6 |
| 14.2.7 | In the last month, how often have you been able to control irritations in your life | 0 1 2 3 4 98 | PSS7 |
| 14.2.8 | In the last month, how often have you felt that you were on top of things? | 0 1 2 3 4 98 | PSS8 |
| 14.2.9 | In the last month, how often have you been angered because of things that were outside of your control? | 0 1 2 3 4 98 | PSS9 |
| 14.2.10 | In the last month, how often have you felt difficulties were piling up so high that you could not overcome them? | 0 1 2 3 4 98 | PSS10 |

# Module 9: Gender and Decision Making

## Section 1: Access, ownership, and control of agricultural production

Now I will ask you about some items that your household may have. First, I would like to know if your household has these items, currently or in the past season (the past 6 months). Then I would like to know who in your household works with or uses these items, who owns them, and who makes decisions about them.

|  | **Item** | **Available**  *If no, skip to next row*  0. No  1. Yes  98. Don’t know | **Who owns**  *Select all that apply* | **Control (decision to purchase/use/sell)**  *Select all that apply* | **Decision on use of income**  *Select all that apply* |
| --- | --- | --- | --- | --- | --- |
|  |  |  | 1. Head of household 2. HH father 3. HH first wife  4. HH Mother 5. HH second wife  6. Other adult female 7. Other adult male  8. N/A | | |
| 9.1.1 | Agricultural land | GLANDA | GLANDO | GLANDC | GLANDD |
| 9.1.2 | Agricultural land use | GLANDUSEA |  | GLANDUSEC |  |
| 9.1.3 | Land for home garden | GGARDA | GGARDO | GGARDC | GGARDD |
| 9.1.4 | Land for non-agricultural purpose (commercial, residential) | GNALANDA | GNALANDO | GNALANDC | GNALANDD |
| 9.1.5 | Cereals | GCEREALA | GCEREALO | GCEREALC | GCEREALD |
| 9.1.6 | Fruit trees | GBANANA | GBANANAO | GBANANAC | GBANANAD |
| 9.1.7 | Roots, tubers | GROOTSA | GROOTSO | GROOTSC | GROOTSD |
| 9.1.8 | Cash crops (coffee, chat, etc.) | GCASHCA | GCASHCO | GCASHCC | GCASHCD |
| 9.1.9 | Vegetables | GVEGA | GVEGO | GVEGC | GVEGD |
| 9.1.10 | Pulses | GPULSA | GPULSO | GPULSC | GPULSD |
| 9.1.11 | Oil seeds | GOILSA | GOILSO | GOILSC | GOILSD |
| 9.1.12 | Spices/herbs | GSPICA | GSPICO | GSPICC | GSPICD |
| 9.1.13 | Cattle | GCATTLEA | GCATTLEO | GCATTLEC | GCATTLED |
| 9.1.14 | Horse/mule/donkey/camels | GMULEA | GMULEO | GMULEC | GMULED |
| 9.1.15 | Sheep/goats | GRUMINA | GRUMINO | GRUMINC | GRUMIND |
| 9.1.16 | Poultry | GPOULTRYA | GPOULTRYO | GPOULTRYC | GPOULTRYD |
| 9.1.17 | Beehives | GBEEHA | GBEEHO | GBEEHC | GBEEHD |
| 9.1.18 | House and other structures | GHOUSEA | GHOUSEO | GHOUSEC | GHOUSED |
| 9.1.19 | Farm equipment (non- mechanized) | GFEQUIPNMA | GFEQUIPNMO | GFEQUIPNMC | GFEQUIPNMD |
| 9.1.20 | Farm equipment (mechanized) | GFEQUIPMA | GFEQUIPMO | GFEQUIPMC | GFEQUIPMD |
| 9.1.21 | Non-farm business equipment | GBUSEQUIPA | GBUSEQUIPO | GBUSEQUIPC | GBUSEQUIPD |

##

## Section 2: Access, ownership, and control of durable goods

Now I will ask you about some other household items. Please let me know if these items are currently available to your household and, if so, who uses them, owns them, and has control over their usage.

**Codes for ownership, access, and control:**

1. Head of Household 2. HH Father 3.HH First Wife 4.HH Mother 5.HH Second wife 6.Other adult female 7. Other adult male 8. N/A

|  | **Item** | **Available**  *If no, skip to next row*  0. No  1. Yes  98. Don’t know | **Quantity available**  *Integer* | **Quantity functional**  *Integer* | **Ownership (non-functional)**  *Select all that apply* | **Ownership**  **(functional)**  *Select all that apply* | **Access**  **(functional)**  *Select all that apply* | **Control of usage (functional)**  *Select all that apply* |
| --- | --- | --- | --- | --- | --- | --- | --- | --- |
| 9.2.1 | Radio | GRADIOA | GRADIOQ | GRADIOQF | GRADIONF | GRADIOO | GRADIOS | GRADIOC |
| 9.2.2 | TV | GTVA | GTVQ | GTVQF | GTVNF | GTVO | GTVS | GTVC |
| 9.2.3 | Telephone – fixed line | GTELEA | GTELEQ | GTELEQF | GTELENF | GTELEO | GTELES | GTELEC |
| 9.2.4 | Mobile phone | GMOBILA | GMOBILQ | GMOBILQF | GMOBILNF | GMOBILO | GMOBILS | GMOBILC |
| 9.2.5 | Bicycle | GBICA | GBICQ | GBICQF | GBICNF | GBICO | GBICS | GBICC |
| 9.2.6 | Motorcycle | GMOTOA | GMOTOQ | GMOTOQF | GMOTONF | GMOTOO | GMOTOAS | GMOTOC |
| 9.2.7 | Three wheeler (Bajaj) | GBAJAJAA | GBAJAJQ | GBAJAJQF | GBAJAJNF | GBAJAJO | GBAJAJS | GBAJAJC |
| 9.2.8 | Cart | GCARTA | GCARTQ | GCARTQF | GCARTNF | GCARTO | GCARTS | GCARTC |

##

## Section 3: Gender and Time Allocation

I am going to ask how you used your time yesterday.

How did you spend your time yesterday, from the time you woke up to the time you went to sleep?

| **Time** | **Response**  1.     Crop agric. work  2.     Livestock agric. work  3. Non-agric. work (own)  4. Non-agric. work (paid)  5. Travel, commuting  6. Shopping/services (including health)  7. Education/training  8.     Household (domestic) and work with children/elderly/sick  9. Personal time (rest, leisure, social, religious, eating etc.) | **Variable name** |
| --- | --- | --- |
| 6:00 (midnight) | 1 2 3 4 5 6 7 8 9 | GTIMEF00 |
| 7:00 | 1 2 3 4 5 6 7 8 9 | GTIMEF01 |
| 8:00 | 1 2 3 4 5 6 7 8 9 | GTIMEF02 |
| 9:00 | 1 2 3 4 5 6 7 8 9 | GTIMEF03 |
| 10:00 | 1 2 3 4 5 6 7 8 9 | GTIMEF04 |
| 11:00 | 1 2 3 4 5 6 7 8 9 | GTIMEF05 |
| 12:00 (morning) | 1 2 3 4 5 6 7 8 9 | GTIMEF06 |
| 1:00 | 1 2 3 4 5 6 7 8 9 | GTIMEF07 |
| 2:00 | 1 2 3 4 5 6 7 8 9 | GTIMEF08 |
| 3:00 | 1 2 3 4 5 6 7 8 9 | GTIMEF09 |
| 4:00 | 1 2 3 4 5 6 7 8 9 | GTIMEF10 |
| 5:00 | 1 2 3 4 5 6 7 8 9 | GTIMEF11 |
| 6:00 | 1 2 3 4 5 6 7 8 9 | GTIMEF12 |
| 7:00 (afternoon) | 1 2 3 4 5 6 7 8 9 | GTIMEF13 |
| 8:00 | 1 2 3 4 5 6 7 8 9 | GTIMEF14 |
| 9:00 | 1 2 3 4 5 6 7 8 9 | GTIMEF15 |
| 10:00 | 1 2 3 4 5 6 7 8 9 | GTIMEF16 |
| 11:00 | 1 2 3 4 5 6 7 8 9 | GTIMEF17 |
| 12:00 (evening) | 1 2 3 4 5 6 7 8 9 | GTIMEF18 |
| 1:00 | 1 2 3 4 5 6 7 8 9 | GTIMEF19 |
| 2:00 | 1 2 3 4 5 6 7 8 9 | GTIMEF20 |
| 3:00 | 1 2 3 4 5 6 7 8 9 | GTIMEF21 |
| 4:00 | 1 2 3 4 5 6 7 8 9 | GTIMEF22 |
| 5:00 | 1 2 3 4 5 6 7 8 9 | GTIMEF23 |

# Module 10: Social Participation and Access to Information

Section 1: Social Participation
Do you or any member of your household currently participate in any of the following social groups?

|  | **Group** | **Index Woman**  1=Yes  0=No  98= Don’t know | **Other household members**  1=Yes  0=No  98= Don’t know |
| --- | --- | --- | --- |
| 10.1.1 | Rural savings and credit cooperative | GRSCCRMM | GRSCCRMOM |
| 10.1.2 | Mother support group | GWOMM | GWOMOM |
| 10.1.3 | A religious group | GRELIGM | GRELIGOM |
| 10.1.4 | A youth group | GYOUTHM | GYOUTHOM |
| 10.1.5 | Producer/farmer association | GHPRODASSMM | GHPRODASSOM |
| 10.1.6 | Kebele Committee (Administration, Health, Other) | GKEBELEM | GKEBELEOM |
| 10.1.7 | Village Saving and Loans (Edir, Ekub, small scale financial group etc.) | GVSLAM | GVSLAOM |
| 10.1.8 | Other group  Specify: ___________ | GOTHM  GOTHMSPE | GOTHOM  GOTHOMSPE |

## Section 4: Program Exposure & Uptake: MHCN and ENGINE activities

|  | **Question** | **Response** | **Variable name** |
| --- | --- | --- | --- |
| 10.4.1 | Have you heard of the Save the Children ENGINE project?  *If no, skip to 10.4.4* | 1=Yes  0=No  98= Don’t know | PEUENGINE |
| 10.4.1 | If yes, from where? | 1. Health Extension Agent  2. One-to-Five Network (Health Development Army or women development army)  3. Kebele health committee  4. Community conversations  5. Social/religious group  6. NGO  7. Radio  8. TV  9. Newspaper  10. Mobile phone  11. Poster/flyers/leaflets  12. Friend/relative  13. Other  98. Don’t know | PEUENGINESRC |
| 10.4.2 | If yes, are you a direct beneficiary of ENGINE? | 1=Yes  0=No  98= Don’t know | PEUENGINEBENEF |
| 10.4.3 | If yes, are you a member of the Most Vulnerable Group supported by ENGINE? | 1=Yes  0=No  98= Don’t know | PEUENGINEMVG |
| 10.4.4 | Did you or anyone in your household receive any information/training on health/nutrition?  *If no, skip next question* | 1=Yes  0=No  98= Don’t know | PEUINFO |
|  | Source of service/information? | 1. Health Extension Worker 2. One-to-Five Network (Health Development Army or women development army) 3. Kebele Health Committee 4. Community Conversations 5. Social/religious group 6. NGO 7. Radio 8. TV 9. Newspaper 10. Mobile phone 11. Poster/flyers/leaflets 12. Friend/Relative 13. Other   98. Don’t know | PEUINFOSRC |

| **Did you receive information/training on the following topics?** | | | | | | | |
| --- | --- | --- | --- | --- | --- | --- | --- |
|  | **A. Did you or anyone in your household participate in this activity in the past 2 years?**  0. No  1. Yes  98. Don’t Know  *If yes or don’t know, skip to C* | **B. If no, why?**  *Select all that apply*  0. Activity not available  1. Insufficient time  2. Too far  3. Too expensive  4. No interest  5. Insufficient benefit  6. Other  98. Don’t know | **C. Source of information?**  *Select all that apply*   1. Health Extension Worker 2. One-to-Five Network (Health Development Army or women development army) 3. Kebele Health Committee 4. Community Conversations 5. Social/religious group 6. NGO 7. Radio 8. TV 9. Newspaper 10. Mobile phone 11. Poster/flyers/leaflets 12. Friend/Relative 13. Other   98. Don’t know | **D. How often did you or someone in your household participate or receive [ACTIVITY/ITEM] in the past 2 years?** | | **E. Did you change your practice due to exposure to this activity?**  0. No  1. Yes  98. Don’t know  *If yes or don’t know, skip F* | **F. If no, why?**  *Select all that apply*  1. Insufficient time  2. Too expensive  3. No interest  4. Insufficient benefit  5. Already practicing behavior  6. Other  98. Don’t know |
|  |  |  |  | *Integer*  98= Don’t know | *Unit*  1. Week  2. Month  3. In the past 6 months  4. In the past year  5. In the past two years |  |  |
|  | PEUPRAC | PEUPRACWHYNOT | PEUPRACSRC | PEUPRACOFT | PEUPRACOFTU | PEUPRACCHANGE | PEUPRACCHANGEWHYNOT |
| How to feed your children for good health? |  |  |  |  |  |  |  |
| Breastfeeding? |  |  |  |  |  |  |  |
| Recognizing signs of illness in your children that require treatment? |  |  |  |  |  |  |  |
| Taking care of yourself during pregnancy? |  |  |  |  |  |  |  |
| Treating your children’s diarrhea? |  |  |  |  |  |  |  |
| Dietary diversity? |  |  |  |  |  |  |  |
| How to cook nutritious meals? |  |  |  |  |  |  |  |
| How to purify water? |  |  |  |  |  |  |  |
| Hand washing? |  |  |  |  |  |  |  |
| Using a latrine? |  |  |  |  |  |  |  |
| HIV/AIDS prevention? |  |  |  |  |  |  |  |

| **Have you participated in any of the following activities?** | | | | | | | |
| --- | --- | --- | --- | --- | --- | --- | --- |
|  | **A. Did you or anyone in your household participate in this activity in the past 2 years?**  0. No  1. Yes  98. Don’t Know  *If yes or don’t know, skip to C* | **B. If no, why?**  **(Select all that apply)**  0. Activity not available  1. Insufficient time  2. Too far  3. Too expensive  4. No interest  5. Insufficient benefit  6. Other  98. Don’t know | **C. Source of information?**  **(Select all that apply)**   1. Health Extension Worker 2. One-to-Five Network (Health Development Army or women development army) 3. Kebele Health Committee 4. Community Conversations 5. Social/religious group 6. NGO 7. Radio 8. TV 9. Newspaper 10. Mobile phone 11. Poster/flyers/leaflets 12. Friend/Relative 13. Other   98. Don’t know | **D. How often did you or someone in your household participate or receive [ACTIVITY/ITEM] in the past 2 years?** | | **E. Did you change your practice due to exposure to this activity?**  0. No  1. Yes  98. Don’t know  *If yes or don’t know, skip F* | **F. If no, why?**  **(Select all that apply)**  1. Insufficient time  2. Too expensive  3. No interest  4. Insufficient benefit  5. Already practicing behavior  6. Other  98. Don’t know |
|  |  |  |  | *Integer*  98= Don’t know | *Unit*  1. Week  2. Month  3. In the past 6 months  4. In the past year  5. In the past two years |  |  |
|  | PEUACT | PEUACTWHYNOT | PEUACTSRC | PEUACTOFT | PEUACTOFTU | PEUACTCHANGE | PEUACTCHANGEWHYNOT |
| Visited a health facility to receive Nutrition Assessment Counseling and Support (NACS)? |  |  |  |  |  |  |  |
| Listened to the *‘she qenat’* radio program? |  |  |  |  |  |  |  |
| Participated in community conversation sessions? |  |  |  |  |  |  |  |
| Attended a Women Support Group to learn about nutrition/personal hygiene? |  |  |  |  |  |  |  |
| Attended a cooking demonstration? |  |  |  |  |  |  |  |
| Attended a homestead gardening demonstration? |  |  |  |  |  |  |  |
| Participated in a women’s saving group? |  |  |  |  |  |  |  |

| Have you received any of the following? | | | | | | | | |
| --- | --- | --- | --- | --- | --- | --- | --- | --- |
|  | **A. Did you or anyone in your household receive this input?**  0. No  1. Yes  98.Don’t Know  *If yes or don’t know, skip to C* | **B. If no, why not?**  **(Select all that apply)**  0. Activity not available  1. Insufficient time  2. Too far  3. Too expensive  4. No interest  5. Insufficient benefit  6. Other  98. Don’t know | **C. Source of information?**  **(Select all that apply)**   1. Health Extension Worker 2. One-to-Five Network (Health Development Army or women development army) 3. Kebele Health Committee 4. Community Conversations 5. Social/religious group 6. NGO 7. Radio 8. TV 9. Newspaper 10. Mobile phone 11. Poster/flyers/leaflets 12. Friend/Relative 13. Other   98. Don’t know | **D. How often did you or someone in your household participate or receive [ACTIVITY/ITEM] in the past 2 years?** | | | **E. Are you using them?**  0. No  1. Yes  98. Don’t know  *If yes or don’t know, skip F* | **F. If no, why not?**  **(Select all that apply)**  1. Insufficient time  2. Too expensive  3. No interest  4. Insufficient benefit  5. Already practicing behavior  6. Other  98. Don’t know |
|  |  |  |  | *Integer*  98= Don’t know | *Unit*  1. Week  2. Month  3. In the past 6 months  4. In the past year  5. In the past two years | |  |  |
| Water purifiers? | PEUINPUT | PEUINPUTWHYNOT | PEUINPUTSRC | PEUINPUTOFT | | PEUINPUTOFTU | PEUINPUTCHANGE | PEUINPUTCHANGEWHYNOT |
| Iron supplements? |  |  |  |  | |  |  |  |
| Folate supplements? |  |  |  |  | |  |  |  |
| Deworming pills? |  |  |  |  | |  |  |  |
| Vitamin A? |  |  |  |  | |  |  |  |
| Iodized salt? |  |  |  |  | |  |  |  |
| Food rations? |  |  |  |  | |  |  |  |
| The Integrated Family Book from the Ministry of Health with 64 essential messages? |  |  |  |  | |  |  |  |
| Deworming pills? | *INFANTS 6 MONTHS AND OLDER ONLY* | | | | | | | |
| Vitamin A? | *INFANTS 6 MONTHS AND OLDER ONLY* | | | | | | | |

## Section 5: Service Delivery and Level of Satisfaction

|  | **Question** | **Response** | **Variable name** |
| --- | --- | --- | --- |
| 10.5.1 | Have you visited the health facility in the past year?  I*f no, skip to 10.5.2* | 1=Yes  0=No | IWLOSHF |
|  | If yes, type of facility visited: | 1. Health center  2. Private clinic  3. Health Post  4. Other (specify: __________________) | IWLOSHFTY  IWLOSHFTYSPE |
|  | Length of last visit | __________ minutes | IWLOSHFM |
| 10.5.2 | How long does it take you to get to the health facility (one way)? | __________ minutes | IWLOSTRV |
| 10.5.3 | Were you satisfied with your visit(s) overall?  *Only answer if responded yes to 10.5.1* | 1. Strongly agree  2. Agree  3. Neither agree nor disagree  4. Disagree  5. Strongly disagree  98. Don’t know | IWLOSHFSAT |
|  | If not, why not?  *Only answer if responded with 3, 4, or 5 to 10.5.3* | 1. Not sufficient time  2. Not enough information  3. Lack of resources (drugs, other)  4. Mistreatment by HF staff  98. Don’t know | IWLOSHFSR |
| 10.5.4 | Have you received home visits from a health worker in the past year?  *If no, skip to next section* | 1=Yes  0=No | IWLOSHV |
|  | Health worker type | 1. Health Extension Worker  2. One in Five Network  3. Health Worker  4. Development agent  5. Other (specify: ______________________) | IWLOSHVTY  IWLOSHVTYSPE |
|  | Length of last visit | ____________ minutes | IWLOSHVM |
| 10.5.5 | How often have you received the home visits in the past 3 months? | 1. 1x/week  2. 1-2x/month  3. 1x /2months  4. 1x /3months | IWLOSHVFR |
| 10.5.6 | Are you satisfied with the home visit? | 1. Strongly agree  2. Agree  3. Neither agree nor disagree  4. Disagree  5. Strongly disagree  98. Don’t know | IWLOSHVSAT |
|  | If not, why not? | 1. Not sufficient time  2. Not enough information  3. Not enough number of visits  98. Don’t know | IWLOSHVSR |

# Module 12 – Income & Expenditure

## Section 1: Other Household Income Sources

Now, please tell me about other sources of income that your household received during July 2012-June 2013. Please include income from all members of your household.

*(Continued on next page)*

|  | **Type of income received** | **Income from this activity?**  0. No  1. Yes  *If no, skip to next row* | **Costs involved in securing this income?**  0. No  1. Yes | **If yes, mention costs incurred**  *Text* | **If yes, total value of costs (Birr)**  *Decimal* | **Form of income received**  1. Cash  2. Non-cash  3.Combination | **Total cash income received**  *Decimal* | **Amount (e.g. no. of sacks, tins)**  *Decimal* | **Units (if not cash)**  *Text* | **Unit value (Birr)**  *Decimal* | **Total value (Birr)**  *Decimal* | **Final income (Birr)**  *Decimal* |
| --- | --- | --- | --- | --- | --- | --- | --- | --- | --- | --- | --- | --- |
| 12.1.1 | Agricultural labor on other's farm (crop or livestock) | MAGI | MAGC | MAGT | MAGCV | MAGF | MAGTCIR | MAGA | MAGU | MAGUV | MAGTV | MAGTVC |
| 12.1.2 | Non-agricultural employment – casual, temporary, or formal/salary (incl. house help, construction, etc.) | MNOAGI | MNOAGC | MNOAGT | MNOAGCV | MNOAGF | MNOAGTCIR | MNOAGA | MNOAGU | MNOAGUV | MNOAGTV | MNOAGTVC |
| 12.1.3 | Pension | MPENSI | MPENSC | MPENST | MPENSCV | MPENSF | MPENSTCIR | MPENSA | MPENSU | MPENSUV | MPENSTV | MPENSTVC |
| 12.1.4 | Assistance from Govt/NGO/UN (including food, seed, or livestock aid) | MASSTI | MASSTC | MASSTT | MASSTCV | MASSTF | MASSTTCIR | MASSTA | MASSTU | MASSTUV | MASSTTV | MASSTTVC |
| 12.1.5 | Remittance income | MREMITI | MREMITC | MREMITT | MREMITCV | MREMITF | MREMITTCIR | MREMITA | MREMITU | MREMITUV | MREMITTV | MREMITTVC |
| 12.1.6 | Assistance from relatives or friends | MASRELI | MASRELC | MASRELT | MASRELCV | MASRELF | MASRELTCIR | MASRELA | MASRELU | MASRELUV | MASRELTV | MASRELTVC |
| 12.1.7 | Gifts | MGIFTI | MGIFTC | MGIFTT | MGIFTCV | MGIFTF | MGIFTTCIR | MGIFTA | MGIFTU | MGIFTUV | MGIFTTV | MGIFTTVC |
| 12.1.8 | Rent out (land, animals, tools/goods) | MRENTI | MRENTC | MRENTT | MRENTCV | MRENTF | MRENTTCIR | MRENTA | MRENTU | MRENTUV | MRENTTV | MRENTTVC |
| 12.1.9 | Sale of fuel wood | MWOODI | MWOODC | MWOODT | MWOODCV | MWOODF | MWOODTCIR | MWOODA | MWOODU | MWOODUV | MWOODTV | MWOODTVC |
| 12.1.10 | Sale of poles/trees | MPOLEI | MPOLEC | MPOLET | MPOLECV | MPOLEF | MPOLETCIR | MPOLEA | MPOLEU | MPOLEUV | MPOLETV | MPOLETVC |
| 12.1.11 | Sale of charcoal | MCHARCI | MCHARCC | MCHARCT | MCHARCCV | MCHARCF | MCHARCTCIR | MCHARCA | MCHARCU | MCHARCUV | MCHARCTV | MCHARCTVC |
| 12.1.12 | Sale of dung (fuel) | MDUNGI | MDUNGC | MDUNGT | MDUNGCV | MDUNGF | MDUNGTCIR | MDUNGA | MDUNGU | MDUNGUV | MDUNGCTV | MDUNGTVC |
| 12.1.13 | Sale of handicrafts | MCRAFTI | MCRAFTC | MCRAFTT | MCRAFTCV | MCRAFTF | MCRAFTTCIR | MCRAFTA | MCRAFTU | MCRAFTUV | MCRAFTTV | MCRAFTTVC |
| 12.1.14 | Sale of prepared food/drinks/ operating a restaurant | MFOODI | MFOODC | MFOODT | MFOODCV | MFOODF | MFOODTCIR | MFOODA | MFOODU | MFOODUV | MFOODTV | MFOODTVC |
| 12.1.15 | Sale of beverages or local brew | MBREWI | MBREWC | MBREWT | MBREWCV | MBREWF | MBREWTCIR | MBREWA | MBREWU | MBREWUV | MBREWTV | MBREWTVC |
| 12.1.16 | Shop keeping | MSHOPI | MSHOPC | MSHOPT | MSHOPCV | MSHOPF | MSHOPTCIR | MSHOPA | MSHOPU | MSHOPUV | MSHOPTV | MSHOPTVC |
| 12.1.17 | Transportation (own work or rental out) | MBODAI | MBODAC | MBODAT | MBODACV | MBODAF | MBODATCIR | MBODAA | MBODAU | MBODAUV | MBODATV | MBODATVC |
| 12.1.18 | Sales of hay/crop residue/compost/grass/fodder | MHAYI | MHAYC | MHAYT | MHAYCV | MHAYF | MHAYTCIR | MHAYA | MHAYU | MHAYUV | MHAYTV | MHAYTVC |
| 12.1.19 | Sale of animal manure | MMANI | MMANC | MMANT | MMANCV | MMANF | MMANTCIR | MMANA | MMANU | MMANUV | MMANTV | MMANTVC |
| 12.1.20 | Sale of game meat | MGAMEI | MGAMEC | MGAMET | MGAMECV | MGAMEF | MGAMETCIR | MGAMEA | MGAMEU | MGAMEUV | MGAMETV | MGAMETVC |
| 12.1.21 | Sale of wild nuts/fruits | MWILDI | MWILDC | MWILDT | MWILDCV | MWILDF | MWILDTCIR | MWILDA | MWILDU | MWILDUV | MWILDTV | MWILDTVC |
| 12.1.22 | Mining (salt, gold, sand, clay, etc.) | MMINEI | MMINEC | MMINET | MMINECV | MMINEF | MMINETCIR | MMINEA | MMINEU | MMINEUV | MMINETV | MMINETVC |
| 12.1.23 | Quarrying stones | MSTONEI | MSTONEC | MSTONET | MSTONECV | MSTONEF | MSTONETCIR | MSTONEA | MSTONEU | MSTONEUV | MSTONETV | MSTONETVC |
| 12.1.24 | Brick making | MBRICKI | MBRICKC | MBRICKT | MBRICKCV | MBRICKF | MBRICKTCIR | MBRICKA | MBRICKU | MBRICKUV | MBRICKTV | MBRICKTVC |
| 12.1.25 | Processing/milling machine | MGMILLI | MGMILLC | MGMILLT | MGMILLCV | MGMILLF | MGMILLTCIR | MGMILLA | MGMILLU | MGMILLUV | MGMILLTV | MGMILLTVC |
| 12.1.26 | Other petty trade | MPETTYI | MPETTYC | MPETTYT | MPETTYCV | MPETTYF | MPETTYTCIR | MPETTYA | MPETTYU | MPETTYUV | MPETTYTV | MPETTYTVC |
| 12.1.27 | Other (specify_____) MOTH1SPE | MOTH1I | MOTH1C | MOTH1T | MOTH1CV | MOTH1F | MOTH1TCIR | MOTH1A | MOTH1U | MOTH1UV | MOTH1TV | MOTH1TVC |
| 12.1.28 | Other (specify_____) MOTH2SPE | MOTH2I | MOTH2C | MOTH2T | MOTH2CV | MOTH2F | MOTH2TCIR | MOTH2A | MOTH2U | MOTH2UV | MOTH2TV | MOTH2TVC |

## Section 3: Other Expenditure

|  | **Over the past one month, did your household purchase or pay for any [ITEM]?**   1. No 2. Yes   98. Don’t know | | ***If yes,* how much did your household pay in total? (Birr)** |
| --- | --- | --- | --- |
| 12.3.1 | Matches | HEMATCH | HEMATCHB |
| 12.3.2 | Batteries | HEBATT | HEBATTB |
| 12.3.3 | Candles (tua’af), incense | HECANDL | HECANDLB |
| 12.3.4 | Laundry soap/OMO/endod/besana leaves | HESOAPL | HESOAPLB |
| 12.3.5 | Hand soap | HESOAPH | HESOAPHB |
| 12.3.6 | Other personal care goods (incl. sendel, matent,) | HEPERSCR | HEPERSCRB |
| 12.3.7 | Charcoal | HECHARC | HECHARCB |
| 12.3.8 | Firewood | HEFIREWD | HEFIREWDB |
| 12.3.9 | Kerosene | HEKEROS | HEKEROSB |
| 12.3.10 | Cigarettes, tobacco, suret, gaya | HETOBAC | HETOBACB |
| 12.3.11 | Transport | HETRANSP | HETRANSPB |
| 12.3.12 | School fees | HESCHOOL | HESCHOOLB |
| 12.3.13 | Health care costs | HEHEALTH | HEHEALTHB |
|  | **Over the past 12 months, did your household purchase or pay for any [ITEM]?**   1. No 2. Yes   98. Don’t know | | ***If yes,* how much did your household pay in total? (Birr)** |
| 12.3.b.1 | Clothes/shoes/fabric for MEN | HECLOTHM | HECLOTHMB |
| 12.3.b.2 | Clothes/shoes/fabric for WOMEN | HECLOTHW | HECLOTHWB |
| 12.3.b.3 | Clothes/shoes/fabric for BOYS | HECLOTHB | HECLOTHBB |
| 12.3.b.4 | Clothes/shoes/fabric for GIRLS | HECLOTHG | HECLOTHGB |
| 12.3.b.5 | Kitchen equipment (cooking pots, etc.) | HEKITCH | HEKITCHB |
| 12.3.b.6 | Linens (sheets, towels, blankets) | HELINEN | HELINENB |
| 12.3.b.7 | Furniture | HEFURN | HEFURNB |
| 12.3.b.8 | Lamp/torch | HELAMP | HELAMPB |
| 12.3.b.9 | Ceremonial expenses | HECEREM | HECEREMB |
| 12.3.b.10 | Contributions to IDDIR | HEIDDIR | HEIDDIRB |
| 12.3.b.11 | Donations to the church | HECHURCH | HECHURCHB |
| 12.3.b.12 | School fees | HESCHO | HESCHOB |
| 12.3.b.13 | Health care costs | HEHELYR | HEHELYRB |

## Section 4: Food Expenditure

|  | **Over the past week (7 days) did you or others in your household consume any item?**  0. No  1. Yes  98. Don’t know | **How much in total did your household consume in the past week?**  *None = 0* | **How much came from purchases?**  *None = 0* | **How much did you spend?**  (V2, 3)  **How much did you spend per unit?** (V4)  (Birr) | **How much came from own production?**  *None = 0* | | **How much came from in-kind exchange?**  *None=0* | **How much came from gifts, food assistance and other programs?** | **Unit**  1. Gram 2. Cm  3. Cm^3^ 4. Number 5. Meter 6. Pair  7. Box 8. Zorba  11. Roll 12. Pack 20. Kg 31. Cup  32. Liter 33. Meter squared |
| --- | --- | --- | --- | --- | --- | --- | --- | --- | --- |
| **Cereals** | | | | | | | | | |
| Teff | FETEFF | FETEFF_TC | FETEFF_PR | FETEFF_S | FETEFF_OP | | FETEFF_G | FETEFF_FA | FETEFF_TCU |
| Wheat | FEWHEAT | FEWHEAT_TC | FEWHEAT_PR | FEWHEAT_S | FEWHEAT_OP | | FEWHEAT_G | FEWHEAT_FA | FEWHEAT_TCU |
| Barley | FEBARLEY | FEBARLEY_TC | FEBARLEY_PR | FEBARLEY_S | FEBARLEY_OP | | FEBARLEY_G | FEBARLEY_FA | FEBARLEY_TCU |
| Maize | FEMAIZE | FEMAIZE_TC | FEMAIZE_PR | FEMAIZE_S | FEMAIZE_OP | | FEMAIZE_G | FEMAIZE_FA | FEMAIZE_TCU |
| Sorghum/millet  V2, 3 only | FESORGHUM | FESORGHUM_TC | FESORGHUM_PR | FESORGHUM_S | FESORGHUM_OP | | FESORGHUM_G | FESORGHUM_FA | FESORGHUM_TCU |
| Sorghum  V4 only | FESORGHUM | FESORGHUM_TC | FESORGHUM_PR | FESORGHUM_S | FESORGHUM_OP | | FESORGHUM_G | FESORGHUM_FA | FESORGHUM_TCU |
| Millet  V4 only | FEMILLET | FEMILLET_TC | FEMILLET_PR | FEMILLET_S | FEMILLET_OP | | FEMILLET_G | FEMILLET_FA | FEMILLET_TCU |
| Rice | FERICE | FERICE_TC | FERICE_PR | FERICE_S | FERICE_OP | | FERICE_G | FERICE_FA | FERCIE_TCU |
| **Pulses** | | | | | | | | | |
| Horsebeans | FEHORESBEANS | FEHORSEBEANS_TC | FEHORSEBEANS_PR | FEHORSEBEANS_S | FEHORSEBEANS_OP | | FEHORSEBEANS_G | FEHORSEBEANS_FA | FEHORSEBEANS_TCU |
| Chickpea | FECHICKPEA | FECHICKPEA_TC | FECHICKPEA_PR | FECHICKPEA_S | FECHICKPEA_OP | | FECHICKPEA_G | FECHICKPEA_FA | FECHICKPEA_TCU |
| Field pea | FEFIELDPEA | FEFIELDPEA_TC | FEFIELDPEA_PR | FEFIELDPEA_S | FEFIELDPEA_OP | | FEFIELDPEA_G | FEFIELDPEA_FA | FEFIELDPEA_TCU |
| Lentils | FELENTILS | FELENTIS_TC | FELENTILS_PR | FELENTILS_S | FELENTILS_OP | | FELENTILS_G | FELENTILS_FA | FELENTILS_TCU |
| Haricot beans | FEHARICOTBEANS | FEHARICOTBEANS_TC | FEHARICOTBEANS_PR | FEHARICOTBEANS_S | FEHARICOTBEANS_OP | | FEHARICOTBEANS_G | FEHARICOTBEANS_FA | FEHARICOTBEANS_TCU |
| **Oil seeds** | | | | | | | | | |
| Niger Seed | FENIGERSEED | FENIGERSEED_TC | FENIGERSEED_PR | FENIGERSEED_S | FENIGERSEED_OP | | FENIGERSEED_G | FENIGERSEED_FA | FENIGERSEED_TCU |
| Linseed | FELINSEED | FELINSEED_TC | FELINSEED_PR | FELINSEED_S | FELINSEED_OP | | FELINSEED_G | FELINSEED_FA | FELINSEED_TCU |
| Sunflower  V4 only | FESUNFLOWER | FESUNFLOWER_TC | FESUNFLOWER_PR | FESUNFLOWER_S | FESUNFLOWER_OP | | FESUNFLOWER_G | FESUNFLOWER_FA | FESUNFLOWER_TCU |
| **Vegetables & Fruit** | | | | | | | | | |
| Onion | FEONION | FEONION_TC | FEONION_PR | FEONION_S | FEONION_OP | | FEONION_G | FEONION_FA | FEONION_TCU |
| Carrot | FECARROT | FECARROT_TC | FECARROT_PR | FECARROT_S | FECARROT_OP | | FECARROT_G | FECARROT_FA | FEONION_TCU |
| Tomato | FETOMATO | FETOMATO_TC | FETOMATO_PR | FETOMATO_S | FETOMATO_OP | | FETOMATO_G | FETOMATO_FA | FETOMATO_TCU |
| Cabbage | FECABBAGE | FECABBAGE_TC | FECABBAGE_PR | FECABBAGE_S | FECABBAGE_OP | | FECABBAGE_G | FECABBAGE_FA | FECABBAGE_TCU |
| Kale | FEKALE | FEKALE_TC | FEKALE_PR | FEKALE_S | FEKALE_OP | | FEKALE_G | FEKALE_FA | FEKALE_TCU |
| Spinach  V4 only | FESPINACH | FESPINACH_TC | FESPINACH_PR | FESPINACH_S | FESPINACH_OP | | FESPINACH_G | FESPINACH_FA | FESPINACH_TCU |
| Swiss Chard  V4 only | FESWISSCHARD | FESWISSCHARD_TC | FESWISSCHARD_PR | FESWISSCHARD_S | FESWISSCHARD_OP | | FESWISSCHARD_G | FESWISSCHARD_FA | FESWISSCHARD_TCU |
| Peppers | FEPEPPERS | FEPEPPERS_TC | FEPEPPERS_PR | FEPEPPERS_S | FEPEPPERS_OP | | FEPEPPERS_G | FEPEPPERS_FA | FEPEPPERS_TCU |
| Avocado | FEAVOCADO | FEAVOCADO_TC | FEAVOCADO_PR | FEAVOCADO_S | FEAVOCADO_OP | | FEAVOCADO_G | FEAVOCADO_FA | FEAVOCADO_TCU |
| Papaya | FEPAPAYA | FEPAPAYA_TC | FEPAPAYA_PR | FEPAPAYA_S | FEPAPAYA_OP | | FEPAPAYA_G | FEPAPAYA_FA | FEPAPAYA_TCU |
| Banana | FEBANANA | FEBANANA_TC | FEBANANA_PR | FEBANANA_S | FEBANANA_OP | | FEBANANA_G | FEBANANA_FA | FEBANANA_TCU |
| Other F/V | FEOTHERFV | FEOTHERFV_TC | FEOTHERFV_PR | FEOTHERFV_S | FEOTHERFV_OP | | FEOTHERFV_G | FEOTHERFV_FA | FEOTHERFV_TCU |
| **Tubers & Stems** | | | | | | | | | |
| Potato | FEPOTATO | FEPOTATO_TC | FEPOTATO_PR | FEPOTATO_S | FEPOTATO_OP | | FEPOTATO_G | FEPOTATO_FA | FEPOTATO_TCU |
| Kocho/Bula | FEKOCHOBULA | FEKOCHOBULA_TC | FEKOCHOBULA_PR | FEKOCHOBULA_S | FEKOCHOBULA_OP | | FEKOCHOBULA_G | FEKOCHOBULA_FA | FEKOCHOBULA_TCU |
| Sweet potato | FESWEETPOTATO | FESWEETPOTATO_TC | FESWEETPOTATO_PR | FESWEETPOTATO_S | FESWEETPOTATO_OP | | FESWEETPOTATO_G | FESWEETPOTATO_FA | FESWEETPOTATO_TCU |
| Garlic  V4 only | FEGARLIC | FEGARLIC_TC | FEGARLIC_PR | FEGARLIC_S | FEGARLIC_OP | | FEGARLIC_G | FEGARLIC_FA | FEGARLIC_TCU |
| **Others** | | | | | | | | | |
| Meat | FEMEAT | FEMEAT_TC | FEMEAT_PR | FEMEAT_S | | FEMEAT_OP | FEMEAT_G | FEMEAT_FA | FEMEAT_TCU |
| Milk | FEMILK | FEMILK_TC | FEMILK_PR | FEMILK_S | | FEMILK_OP | FEMILK_G | FEMILK_FA | FEMILK_TCU |
| Cheese | FECHEESE | FECHEESE_TC | FECHEESE_PR | FECHEESE_S | | FECHEESE_OP | FECHEESE_G | FECHEESE_FA | FECHEESE_TCU |
| Eggs | FEEGGS | FEEGGS_TC | FEEGGS_PR | FEEGGS_S | | FEEGGS_OP | FEEGGS_G | FEEGGS_FA | FEEGGS_TCU |
| Butter | FEBUTTER | FEBUTTER_TC | FEBUTTER_PR | FEBUTTER_S | | FEBUTTER_OP | FEBUTTER_G | FEBUTTER_FA | FEBUTTER_TCU |
| Oil | FEOIL | FEOIL_TC | FEOIL_PR | FEOIL_S | | FEOIL_OP | FEOIL_G | FEOIL_FA | FEOIL_TCU |
| Sugar | FESUGAR | FESUGAR_TC | FESUGAR_PR | FESUGAR_S | | FESUGAR_OP | FESUGAR_G | FESUGAR_FA | FESUGAR_TCU |
| Salt | FESALT | FESALT_TC | FESALT_PR | FESALT_S | | FESALT_OP | FESALT_G | FESALT_FA | FESALT_TCU |
| Other spices | FEOTHERSPICES | FEOTHERSPICES_TC | FEOTHERSPICES_PR | FEOTHERSPICES_S | | FEOTHERSPICES_OP | FEOTHERSPICES_G | FEOTHERSPICES_FA | FEOTHERSPICES_TCU |
| Soft drinks | FESOFTDRINKS | FESOFTDRINKS_TC | FESOFTDRINKS_PR | FESOFTDRINKS_S | | FESOFTDRINKS_OP | FESOFTDRINKS_G | FESOFTDRINKS_FA | FESOFTDRINKS_TCU |
| Other | FEOTHER | FEOTHER_TC | FEOTHER_PR | FEOTHER_S | | FEOTHER_OP | FEOTHER_G | FEOTHER_FA | FEOTHER_TCU |
| **Stimulants** | | | | | | | | | |
| Coffee | FECOFFEE | FECOFFEE_TC | FECOFFEE_PR | FECOFFEE_S | | FECOFFEE_OP | FECOFFEE_G | FECOFFEE_FA | FECOFFEE_TCU |
| Chat/Kat  V3 only | FECHATKAT | FECHATKAT_TC | FECHATKAT_PR | FECHATKAT_S | | FECHATKAT_OP | FECHATKAT_G | FECHATKAT_FA | FECHATKAT_TCU |

# Module 13: Laboratory Tests and Anthropometric Measurements

## Section 1: Malaria Testing – Index Woman

|  | **Question** | **Response** | **Variable** |
| --- | --- | --- | --- |
| 13.1.1 | Index woman result | C band 1=yes 0=no  Pan band 1=yes 0=no  Pf band 1=yes 0=no  0=negative  1=positive  2=test not done  (reason __________________) | LCCBCG  LCPANCG  LCPFCG  LCRAPCG  LCRAPCGR |
| 13.1.2 | If the result is positive, did the woman take treatment for malaria in the last 7 days?  *If no, skip to 13.1.4* | 1=Yes  0=No | LCTRTCG |
| 13.1.3 | If yes, did the woman complete the course of malaria treatment? | 1=Yes  0=No | LCMALATRTCG |
| *If the index woman tests positive for malaria and has finished a full course of treatment, refer to a nearby health facility. Otherwise, provide full course of 1^st^ line treatment.* | | | |
| 13.1.4 | What treatment is given for the woman? | 0=None  1=1^st^ line treatment  2=Referred | LCWHATRTCG |

## Section 2: Index Woman Anthropometry

|  | **Question** | **Response** | **Var. name** |
| --- | --- | --- | --- |
| 13.2.1 | Index woman’s initials | *Text* | AMINIT |
| 13.2.2 | Year of birth |  | AMYOB |
| 13.2.3 | Age in years |  | AMYRS |
| 13.2.4 | Pregnant | 1=Yes  0=No | AMPREG |
| 13.2.4a  V3 only | Fundal Height | . cm | AMFHCM |
| 13.2.5 | Weight 1 | . kg | AMWT1 |
| 13.2.6 | Weight 2 | . kg | AMWT2 |
| 13.2.7 | Weight 3 | . kg | AMWT3 |
| 13.2.8 | Height 1 | . cm | AMHT1 |
| 13.2.9 | Height 2 | . cm | AMHT2 |
| 13.2.10 | Height 3 | . cm | AMHT3 |
| 13.2.11 | MUAC 1 | . cm | AMMUAC1 |
| 13.2.12 | MUAC 2 | . cm | AMMUAC2 |
| 13.2.13 | MUAC 3 | . cm | AMMUAC3 |
| 13.2.14 | Blood pressure (systolic) | mm Hg | AMSYSBP |
| 13.2.15 | Blood pressure (diastolic) | mm Hg | AMDYABP |
| 13.2.16 | Referral given? | 1=Yes  0=No | AMREF |

## Section 5 – Hemocue Measurements (Index Woman)

|  | **Question** | **Index Woman** | **IW var. name** |
| --- | --- | --- | --- |
| 13.5.1 | Initials |  | BLCGINIT |
| 13.5.2 | HemoCue done | 1=Yes  0=No | BLHEAMCG |
| 13.5.3 | HemoCue result (g/dL)  *Skip if HemoCue not done* | . | BLHEAMCGRE |
| 13.5.4 | Reason for no HemoCue  *Skip if HemoCue not done* | *Text* | WHYNOHCG |
| 13.5.5 | Referral? | 1=Yes  0=No | REFCG |

End of survey for time point 1
