# Supplementary material for: Nutrition-specific and nutrition-sensitive factors associated with mid-upper arm circumference as a measure of nutritional status in pregnant Ethiopian women: Implications for programming in the first 1000 days
Source: PLoS One. 2019 Mar 26;14(3):e0214358. doi: 10.1371/journal.pone.0214358 (PMC6435172; doi:10.1371/journal.pone.0214358)
Supplement: S3 File — Questionnaires in Afan-Oromo, the language of questionnaire administration. (ZIP) [file pone.0214358.s003.zip › INDEX WOMEN time piont 1 AFAN OROMO-FINAL.docx]

Afgaafii Qorannoo Maatii Kan ENGINE - USAIDtiin Gaggeeffamu

**Gaaffiifi Deebii Dubartii Qoratamaa Jirtuuf**

Qabiyyee

[Mujulii 1: Odeeffannoo Fi Haala Maatii 3](#_Toc390254252)

[Kutaa 1: Odeeffannoo Gaafif Deebii 3](#_Toc390254253)

[Kutaa 2: Odeeffannoo Maatii 4](#_Toc390254254)

[Kutaa 3: Waa’ee Beekumsa Duubartii Qorannoo Kanaaf Barbaadamtuu 4](#_Toc390254255)

[Kutaa 4: Haala Hawaasummaa fi Diinagdee 5](#_Toc390254256)

[Mujulii 2: Bishaan Fi Qulqullina 6](#_Toc390254257)

[Kutaa 1: Itti Fayyadamaa Fi Madda Bishaanii 6](#_Toc390254258)

[Kutaa 2: Qulqullina [Sanitation & Hygiene] 7](#_Toc390254259)

[Mujulii 14 -Madaallii sadarkaa xinsammuuf hawaasummaa 8](#_Toc390254260)

[Kutaa 1. Tarreefama mudannoowwan yaaddessoo/cimaa/ ta’anii 8](#_Toc390254261)

[Kutaa 3: Gaaffiilee Fayyaa Dhukubsataa (GFD)-9 fi yaalii 12](#_Toc390254262)

[Mujulii 3: Soorata fi wabii nyaataa duubartii qorannoo kanaaf barbaadamtuu 15](#_Toc390254263)

[Kutaa 1a: Soorata Duubartii Qorannoo Kanaaf Barbaadamtuu- Kan Sa’aa 24 Yaadachuu 15](#_Toc390254264)

[Kutaa 1b: Haala Soorata Dubartii Qo’annoof Barbaadamtu 17](#_Toc390254265)

[Kutaa 2: Ji’oota Maatiin Nyaata Gahaa Itti Argatan 21](#_Toc390254266)

[Kutaa 3: Madaallii/Safartuu dhiheennatti argama fi dhabiinsa wabii nyaataa maatii 22](#_Toc390254267)

[Kutaa 4: Nyaata Yeroo Ulfaa Fi daa’ima Hoosisan Laguu ta’an 24](#_Toc390254268)

[Kutaa 5: Caatii Qama’uu 27](#_Toc390254269)

[Mujulii 14 -Madaallii sadarkaa xinsammuuf hawaasummaa 27](#_Toc390254270)

[Kutaa 6:- Madallii deeggarsa Hawaasaa Haadholii (MWHH) 27](#_Toc390254271)

[Kutaa 7:- Meeshaa HITS kan hubama/miidhaa/ hiriya dhihoo kan qoru 28](#_Toc390254272)

[Mujulii 4: Ulfaa Fi Haala FayyaaDubartii Qorannoof barbaadamtuu 28](#_Toc390254273)

[Kutaa 1: Gamaaggama/Qorannaa Haala Fayyaa 28](#_Toc390254274)

[Kutaa 3: Ulfa kanaan Duraa 29](#_Toc390254275)

[Kutaa 4: Ulfa Ammaa 30](#_Toc390254276)

[Mujulii 14 -Madaallii sadarkaa xinsammuuf hawaasummaa 31](#_Toc390254277)

[Kutaa 4 :-Madaalli amala muddannoowwan sodaachuu abbaa qabxii ja’a (six item state trait anxiety scale) 31](#_Toc390254278)

[Kutaa 5: Sodaa ulfa waliin wal-qabate 31](#_Toc390254279)

[MOdujulii 15: Hordoffii Beekumsa Nyaataa 33](#_Toc390254280)

[Mujulii 14 -Madaallii sadarkaa xinsammuuf hawaasummaa 33](#_Toc390254281)

[Kutaa 2: Madaallii sadarkaa dhiphinaa/yaaddoo 33](#_Toc390254282)

[Mujulii 9: Koornayaa Fi Murtee Kennuu 34](#_Toc390254283)

[Kutaa 1: Itti Dhiheenna, Abbummaa , Fi Too’annoo Oomisha Qonnaa 34](#_Toc390254284)

[Kutaa 2: Itti Dhiheenna, Abbummaa, Fi Too’annoo Meeshaalee Yeroo Dheeraaf Turan 36](#_Toc390254285)

[Kutaa 3: Koornayaa Fi Yeroo Ramaduu/Qooduu 38](#_Toc390254286)

[MuJulii 10: Hirmaannaa Hawaasummaa fiitti Dhiheenna Odeeffannoo 39](#_Toc390254287)

[kutaa 1: Hirmaannaa hawaasummaafi itti gamadinsa isaa 39](#_Toc390254288)

[Kutaa 4: Sagantaaf Saaxilamuu fi Fudhatamummaa Isaa –MHCN and ENGINE Activities 39](#_Toc390254289)

[Kutaa 5: Tajajila Fi Ittigamadinsa 42](#_Toc390254290)

[Mujulii 12 – Galii fi Baasii 43](#_Toc390254291)

[Kutaa 1: Madda Galii Matii Kan Biroo 43](#_Toc390254292)

[Kutaa 4: Baasiiwwan Biroo 46](#_Toc390254293)

[Mujulii 13: Qorannoo Laabraatorii fi Safara antirooppoomeetriikii 47](#_Toc390254294)

[[Anthropometric Measurements] 47](#_Toc390254295)

[Kutaa 1: Qorannoo Busaa – Dubartii qo’annaaf barbaadamtu/qo’atamtu 47](#_Toc390254296)

[Kutaa 2: Antirooppoomeetriikii Dubartii Qo’annaaf barbaadamtu [Index Woman Anthropometry] 47](#_Toc390254297)

[Kutaa 5: safara hemocue 48](#_Toc390254298)

# Mujulii 1: Odeeffannoo Fi Haala Maatii

## Kutaa 1: Odeeffannoo Gaafif Deebii

**Gabatee 1.1**

| **Lakkoofsa** | **Gaafii** | **Deebii** | **Variable name** |
| --- | --- | --- | --- |
|  | Guyyaa gaafif deebii (gg/jj/ww) | // | HDATEINT |
|  | Marsaa doo’annoo (1-9) |  | HDTIMEPT |
|  | Lakkoofsa manaa [HH ID] |  | HHID |
|  | Aanaa | *(galmee irraa ilaali/buusi: Wolisoo, Goommaa, XirooAfataa)* | HWOREDA |
|  | Ganda | *(barreessi )* | HKEBELE |
|  | Gooxii / Garee | *(barreessi)* | HGOTE |
|  | GPS | “Qindeessaa GPS Barbaadi”[biinkoo/ (button) | HHGPS |
|  | Eenyummaa gaafataa- 1 |  | HID1 |
|  | Eenyummaa gaafataa- 2 |  | HID2 |
|  | Eenyummaa Too’ataa |  | HSID |
|  | Bu’aa gaaffiif deebii | 1. Xumurte  2. Hin xumure  3.hin argamne  4. ni didde  5.eddoo ishee beekuu hin dandandeenne | HINT OUT |

## Kutaa 2: Odeeffannoo Maatii

Gaaffiilee waa’ee keetiif maatii keetii ilaalatu sigaafachuun fedha.

Ragaa sasaabduu/sasaabaafi: dura dubartii qorannoo kanaaf Deebii kennituun jalqabi sana boodaa waa’ee itti gaafatamaa/tuu maatiifi miseensaa maatii kamiinuu gaafadhu

| Maqaa guutuu hanga akaakayyuu | Walittidhufeenya  Itti gaafatamaa maatii woliin qaban  1=itti gaafatamaa maatii  2.Abbaa  3. Haadha  4. haadha manaa  5.ijoollee/daa’ima  6. soddaatii/amaatii  7.Soddaa  8. niitii lamataa  9. niitii sadaffaa  10. niitii arfaffaa  11.mucaa ilma/intala kootii  12.akkawoo/akaakayyuu  13.obboleewwan  14. abbeeraa/wasiila  15. adaadaa/akist  16. ijoollee miseensa maatii  17. ga’eessa biraa/other adult | Torbaanitti yoo diqqaate guyyaa sadiif isin wajjiin jiraachaa nyaata maatiif qophaa’e isin wajjiin kan nyaachaa ture jiraa?  1=eeyyee , 0=lakki 98=Hinbekuu | Saalaa  1=Dhalaa  0= Dhiira | Umrii(woggaadhaan)  Woggaa laamaa gadi yoo ta’e ji’aan barreessi | | Haala gaa’elaa  1=kanheerumte, tokko qofatti;  2= kanheerumte, dhirsa heddu;  3=osoo walii hin fuudhin kanwaliin jiraatan ; 4=kan hinheerumne; 5=kanjalaa du’e; 6=seeraan kanwalhiike;  7=kan addaan/gargar bahan | Sadarkaa baruumsa xumurtee  Woggaa_____  98. hin beeku | Amantaa  1. ortodoksii  2.Kaatolikii  3.prootestaantii  4. Islaama  5. kan aadaa  6.amantaa hinqabu  7. kan biroo | Ulfaa?  1= Eeyyee  0=Lakki  98=hin beeku  Duubaraafi kan umriin woggaa 12 oli ta’e qofaaf. |
| --- | --- | --- | --- | --- | --- | --- | --- | --- | --- |
|  |  |  |  | woggaa | Ji’a |  |  |  |  |
|  |  |  |  |  | |  |  |  |  |

## Kutaa 3: Waa’ee Beekumsa Duubartii Qorannoo Kanaaf Barbaadamtuu

| **Lakk.** | **Gaafii** | **Deebii** | **Variable name** |
| --- | --- | --- | --- |
| 1.3.1 | Dubartii Qorannoo Kanaaf Barbaadamtu maqaa ishee afaan naannootiin barreessuu dandeessii? (maaloo mee qoradhu) | 1=eeyyee , 0=lakki 98= hinqoratamne | HIWWRITE |
| 1.3.2 | Dubartii Qorannoo Kanaaf Barbaadamtu Hima kanatti aanu dubbisuu dandeessii?  (fakii barreeffama naannoo) | 1=eeyyee , 0=lakki 98= hinqoratamne | HIWREAD |
| 1.3.3 | Dubartii Qorannoo Kanaaf Barbaadamtu gaafii herregaa itti aanu sirriin deebisuu dandeessii?  *“osoo killee qarshii 30tti gurgurtee handaaqqos qarshii 50tti osoo gurgurte, walitti qarshii meeqa qabda?"* | 1=eeyyee, 0=lakki. 98= hinqoratamne | HIWNUM |

##

## Kutaa 4: Haala Hawaasummaa fi Diinagdee

Gaafilee waa’ee akaakuu mana ati keessa jiraattu sigaafachuu barbaada.

*(Ragaa Guuraaf: ilaaludhaan deebii mirkaneessi.)*

**Gabatee 1.4 Akaakuu Manaa**

| **Lakk.** | **Gaafii** | **Deebii** | **Variable name** | **Mirkaneessitee?** |
| --- | --- | --- | --- | --- |
| 1.4.1 | Akaakuu dhaaba manaa ijoo | 1. Mukaaf dhoqqee 2. Xuubii dhoqqee ykn xuubii waddame /gubame 3. Simintoodhaan /concrete block 4. Muka 5. Marga/leemman 6. kanbiraa (ibsi ________) | HWALL  HWALLSPE | Eeyyee /lakki  HWALLCF |
| 1.4.2 | Akaakuu baaxii /sora manaa ijoo | 1. kanmargaan yknbaalaan kabame  2. qorqoorroo  3. taayilii [Tile] waan laastikii ykn supheerraa tolfamuuf baaxii manaatti maxxanfamu  4. kanbiraa (ibsi_______) | HROOF  HROOFSPE | Eeyyee /lakki  HROOFCF |
| 1.4.3 | Akaakuu afaala manaa ijoo/floor | 1.dhoqqee ykn kosii  2. xuubii/dhakaa/Simintoo  3. taayilii [Tile] waan laastikii ykn supheerraa tolfamuuf baaxii manaatti maxxanfamu  4. kanbiraa (ibsi_______) | HFLOOR  HFLOORSPE | Eeyyee /lakki  HFLOORCF |
| 1.4.4 | Akaakuu mana fincaanii kanmaatiin yerooheddu itti fayyadamu: | 1. hinjiru/daggala/oddoo  2.mana fincaanii aadaa[kanhinfooyyofne]  3. mana fincaanii fooyya’aa  4. mana fincaanii bishaaniin hojjatu  5. mana fincaanii hawaasaa  6. kanbiraa (ibsi_______) | HTOILET  HTOILSPE | Eeyyee /lakki  HTOILETCF |
| 1.4.5 | Manni keessan bishaan ujummoo qabaa? | 1=eeyyee , 0=lakki | HRWATER | Eeyyee /lakki  HRWATERCF |
| 1.4.6 | Manni keessan/ korreentii/ ibsaa elektiriikii qabaa? | 1=eeyyee , 0=lakki | HELECT | Eeyyee /lakki  HELECTCF |
| 1.4.7 | Yeroo baay’ee nyaata bilcheeffachuuf maal fayyadamtu ? | 1. muka  2. kasala  3. boba’aa ykn baayoogaazii  4. korreentii/Electricity  5. bobaa adii/Kerosene  6.koboota  7. kanbiroo (ibsi_________) | HFUEL  HFUELSPE | Eeyyee /lakki  HFUELCF |
| 1.4.8 | Maddi ijoon boba’aa ykn anniisaa ibsaadhaa maatii keetii maali? | - - 1. korreentii/Electricity     2. Soolaarii     3. Boba’aa /gaazii     4. Maashoo /Faanosa     5. Kurraazii     6. Midijjaa /qoraan /kobotaan     7. kanbiraa (ibsi_______) | HLIGHT  HLIGHTSPE | Eeyyee /lakki  HLIGHTCF |

# Mujulii 2: Bishaan Fi Qulqullina

## Kutaa 1: Itti Fayyadamaa Fi Madda Bishaanii

Gabatee 2.1

|  | **Gaafii** | **Deebii** | **Var. name** |
| --- | --- | --- | --- |
| 2.1.1 | Maaddi bishaanii dhugaatiif maatiin keessan baay’naan fayyadamu maali? | 1=bishaan uujummoo, 2=boonoo, 3=bishaan biirii/boollaa , 4=burqaa/bishaan boollaa sirnaan eeggame,  5= burqaa/bishaan boollaa sirnaan hineeggamne, 6=bishaan roobaa, 7=laga ykn haroo, 8=bishaan samsame, 9=kanbiraa (ibsi_______) 98= hin beeku | WSOURCE  WSOURSPE |
| 2.1.2 | Maddi bishaanii maatiin keessan qonnaan ala baay’naan fayyadamu maali? | 1=bishaan dhuujummoo, 2=boonoo, 3=bishaan biirii/boollaa , 4=burqaa/bishaan boollaa sirnaan eeggame,  5= burqaa/bishaan boollaa sirnaan hineeggamne, 6=bishaan roobaa, 7=laga ykn haroo, 8=bishaan samsame, 9=kanbiraa (ibsi_______) 98= hin beeku | WSRCOTH  WSRCOTHSBE |
| 2.1.3 | Maddi bishaanii mana kee irraa hangam fagaata (gartokko qofa-dhaqa/gala qofa)? Yoo maddi lama ta’e isa fagoo filadhu. | __________ kiiloomeetiraan; maddi bishaanii mooraa keessa yoo ta’e, 0 barreessi) 98= hin beeku | WDISTANC |
| 2.1.4 | Al tokko Bishaan waraabdee deebi’uuf (waraabachuuf yeroo eegdu dabalatee, haala baramaan)? Isa fagoo filadhu. | Daqiiqaa _______  98= hin beeku | WTIME |
| 2.1.5 | Maatiin kee bishaan roobaa tajaajila mana keessaaf ni kuufataa? | 0=lakki  1=eeyyee  98= hin beeku | WRAIN |
| 2.1.6 | Bishaan osoo hindhugiin qulqullina isaa eeguuf wanti gootan jiraa? | 1=homaa hingoonu, 2= danfisuu, 3= qoricha aadaa fayyadamuu, 4=keemikaala fayyadamuu (eegduu bishaanii,dhangala’oo, wuhaa aggaar, bishaan gaarii), 5=dhinbiibuu, 6=bishaan waraabanii tursiisun qulqulluuf boora’a a addaan baasuu/decant, 7=kanbiraa (ibsi___________) 98= hin beeku | WTREAT  WTREATSPE |
| 2.1.7 | Bishaan dhugaatiif oolu bishaa biraaf fayyadamtu irraa addatti kuufattuu? | 1=Eeyyee  0=Lakki  98= hin beeku | WWATSTR |
| 2.1.8 | Maatiin kee Bishaan dhugamu maal keessatti kuufatu? | 1=hubboo/washoo aadaa qadaada qabu,  2= hubboo aadaa qadaada malee, 3=jeerikaana qadaada malee , 4= jeerikaana qadaada qabu,  5= kanbiraa (ibsi_________) 98= hin beeku | WSTORE  WSTORESPE |
| 2.1.9 | Maatiinkee guyyaa tokkotti Bishaan hangamii fayyadamu? | ___________litra  98= hin beeku | WJCANS |

## Kutaa 2: Qulqullina [Sanitation & Hygiene]

Gabatee 2.2

|  | Balfa/kosii manaa bahuu akkamitti maqsita? (filannoo hindubbisiin. Deebiikennaan akka deebisu godhi, sana booda deebii gaafii hundaa armaan gaditti guuti .) | | | | |
| --- | --- | --- | --- | --- | --- |
| 2.2.1 | Boola balfaa/kosii | 1=eeyyee  0=lakki | | | WPIT |
| 2.2.2 | Oddoottigatuu | 1= eeyyee  0=lakki | | | WGARDEN |
| 2.2.3 | Daggalatti gatuu | 1=eeyyee  0=lakki | | | WBUSH |
| 2.2.4 | Badheetti gubuu | 1= eeyyee  0= lakki | | | WBURN |
| 2.2.5 | kanbiroo (ibsi_______) | 1=eeyyee  0=lakki | | | WOTHER |
| 2.2.6 | Nyaata qophaa’e irra caalaa akkamitti kuufattu/kaawwattu ? ( (filannoo hindubbisiin. Deebiikennaan akka deebisu godhi, sana booda deebii gaafii hundaa armaan gaditti haalaan guuti .) | 1. meeshaa hinqadaadamne  2. meeshaa qadaadame 3. Abiddarratti ykn daaraa oo’aa 4. kanbiroo (ibsi_______)  98=hinbekuu | | | WFDSTORE  WFDSTORESPE |
| 2.2.7 | Yeroo baay’ee meeshaalee nyaata qulqullaa’an/dhiqaman eessatti kuufattu? | 1.zalangaa/girgijii/shelf  2.afaala/floor  3.meeshaa qadaadame  4.hin ilaallatu  5.kan biroo ibsi  98.hin beeku | | | WDISH STORE  WDISHSOTH |
| Harka kandhiqattu Yeroo kam ? (Deebiikennaan akka deebisu godhi, sana booda deebii gaafii hundaa armaan gaditti guuti .) | | | | | |
| 2.2.8 | Gonkumaa | | 1=eeyyee  0= lakki  98= hin beeku | WNIL | |
| 2.2.9 | Xuriin mul’atu yoo jiraate | | 1=eeyyee  0= lakki  98= hin beeku | WDIRT | |
| 2.2.10 | Erga manfincaanii fayyadamee booda/booliin/fincaaniin | | 1=eeyyee  0= lakki  98= hin beeku | WTOILETUSE | |
| 2.2.11 | Daa’ima boolii baye qulqulleessuun booda | | 1=eeyyee  0= lakki  98= hin beeku | WCLEANCHILD | |
| 2.2.12 | Nyaata qopheessuun dura | | 1=eeyyee  0= lakki  98= hin beeku | WFOOD | |
| 2.2.13 | Nyaata dhiheessuun dura | | 1=eeyyee  0= lakki  98= hin beeku | WMEAL | |
| 2.2.14 | Nyaachuun dura | | 1=eeyyee  0= lakki  98= hin beeku | WEAT | |
| 2.2.15 | Daa’ima nyaachisuun dura | | 1=eeyyee  0= lakki  98= hin beeku | WFEEDBABY | |
| 2.2.16 | Yeroo yaadadhu | | 1=eeyyee  0= lakki  98= hin beeku | WREMIND | |

#

# Mujulii 14 -Madaallii sadarkaa xinsammuuf hawaasummaa

## Kutaa 1. Tarreefama mudannoowwan yaaddessoo/cimaa/ ta’anii

Muddannoowwan yaaddeessoo gurguddaa ta’an kan armaan gadii keessaa waggaa/bara/ darbe keessa kan si quunname turee? Mee muddannoowwan kun kan si quunname wayiita ulfaa keessa ta’ee fi dhisee isaa adda baasii natti mul’isi.

|  | Waa’ee mudannoowwan ji’oota 12’n darban keessatti rawwatamanii ilaalchisee gaaffiilee yartuun si gaafadhun qaba | | | |
| --- | --- | --- | --- | --- |
| 14.1.1 | (Ji’oota 12’n darbaan keessatti) Ofuma mataa keetiif dhibee cimaan, miidhaamni qaamaa yookiin rukkuttaan si qunnamee ture? | Eeyyee  Lakki  Hin beeku  Deebii hin deebifne/dide/ | 1  2  88  99 | LEILL |
| 14.1.2 | yoo deebiin gaaffii 1A’f ‘eeyyee’ ta’e, gaaffii itti aanu gaafadhu:  Mudannoon kun kan si mudate erga ulfa taate booda dhaa? | Eeyyee  Lakki  Hin beeku/hin yaadadhu  Deebii hin deebifne/dide/ | 1  2  88  99 | LEILLPR |
| 14.1.3 | (Ji’oota 12’n darbaan keessatti) fira dhihoo kee irra dhibee cimaan, miidhaamni qaamaa yookiin rukkuttaan mudatee turee? | Eeyyee  Lakki  Hin beeku  Deebii hin deebifne/dide/ | 1  2  88  99 | LEILREL |
| 14.1.4 | yoo deebiin gaaffii 2A’f ‘eeyyee’ ta’e, gaaffii itti aanu gaafadhu:  Mudannoon kun kan si mudate erga ulfa taate booda dhaa? | Eeyyee  Lakki  Hin beeku  Deebii hin deebifne/dide/ | 1  2  88  99 | LEILRPR |
| 14.1.5 | (Ji’oota 12’n darban keessatti) abbaan manaa kee, maatii kee yookiin ijoollee kee keessaa namni du’e turee? | Eeyyee  Lakki  Hin beeku  Deebii hin deebifne/dide/ | 1  2  88  99 | LEBER |
| 14.1.6 | yoo deebiin gaaffii 3A’f ‘eeyyee’ ta’e, gaaffii itti aanu gaafadhu:  Mudannoon kun kan si mudate erga ulfa taate booda dhaa? | Eeyyee  Lakki  Hin beeku  Deebii hin deebifne/dide/ | 1  2  88  99 | LEBERPR |
| 14.1.7 | (Ji’oota 12’n darban keessatti) hiriyaan dhihoon maatii keetii yookiin fira biroo keessaa kan du’ee turee? | Eeyyee  Lakki  Hin beeku  Deebii hin deebifne/dide/ | 1  2  88  99 | LEBERF |
| 14.1.8 | yoo deebiin gaaffii 4A’f ‘eeyyee’ ta’e, gaaffii itti aanu gaafadhu:  Mudannoon kun kan si mudate erga ulfa taate booda dhaa? | Eeyyee  Lakki  Hin beeku/hin yaadadhu  Deebii hin deebifne/dide/ | 1  2  88  99 | LEBERFPR |
| 14.1.9 | (Ji’oota 12’n darban keessatti) sababa rakkoowwan waliin jireenyaa irraan kan ka’e, abbaa warraa kee waliin wal-hiiktee turtee? | Eeyyee  Lakki  Hin beeku  Deebii hin deebisuu dide | 1  2  88  99 | LEMAR |
| 14.1.10 | yoo deebiin gaaffii 5A’f ‘eeyyee’ ta’e, gaaffii itti aanu gaafadhu:  Mudannoon kun kan si mudate erga ulfa taate booda dhaa? | Eeyyee  Lakki  Hin beeku  Deebii hin deebisuu dide | 1  2  88  99 | LEMARPR |
| 14.1.11 | (Ji’oota 12’n darban keessatti) hiriyummaa yookiin walitti dhufeenyi cimaa ta’e addaan cite turee? | Eeyyee  Lakki  Hin beeku/hin yaadadhu  Deebii hin deebifne/dide/ | 1  2  88  99 | LEREL |
| 14.1.12 | yoo deebiin gaaffii 6A’f ‘eeyyee’ ta’e, gaaffii itti aanu gaafadhu:  Mudannoon kun kan si mudate erga ulfa taate booda dhaa? | Eeyyee  Lakki  Hin beeku/hin yaadadhu  Deebii hin deebifne/dide/ | 1  2  88  99 | LERELPR |
| 14.1.13 | (Ji’oota 12’n darban keessatti) hiriyaa dhihoo, ollaa yookiin fira kee irraa rakkoo cimaan si mudate turee? | Eeyyee  Lakki  Hin beeku  Deebii hin deebifne/dide/ | 1  2  88  99 | LEFRIE |
| 14.1.14 | yoo deebiin gaaffii 7A’f ‘eeyyee’ ta’e, gaaffii itti aanu gaafadhu:  Mudannoon kun kan si mudate erga ulfa taate booda dhaa? | Eeyyee  Lakki  Hin beeku  Deebii hin deebifne/dide/ | 1  2  88  99 | LEFRIEPR |
| 14.1.15 | (Ji’oota 12’n darban keessatti) rakkoon guddaan hir’ina qarshii (yaaddoo cimaan dhabiinsa qarshiin walqabate) si mudatee turee? | Eeyyee  Lakki  Hin beeku  Deebii hin deebifne/dide/ | 1  2  88  99 | LEFIN |
| 14.1.16 | yoo deebiin gaaffii 8A’f ‘eeyyee’ ta’e, gaaffii itti aanu gaafadhu:  Mudannoon kun kan si mudate erga ulfa taate booda dhaa? | Eeyyee  Lakki  Hin beeku  Deebii hin deebifne/dide/ | 1  2  88  99 | LEFINPR |
| 14.1.17 | (Ji’oota 12’n darban keessatti) waa si jalaa baduu yookiin wanti ta’e si jalaa hatamuu isaa irraan kan ka’e baayyee si dhiphise turee? | Eeyyee  Lakki  Hin beeku  Deebii hin deebifne/dide/ | 1  2  88  99 | LETHEF |
| 14.1.18 | yoo 9A’f eeyyee jette ta’e, kan armaan gadii gaafadhu:  Mudannoon kun kan raawwate erga ati ulfa taate booda dhaa? | Eeyyee  Lakki  Hin beeku  Deebii hin deebifne/dide/ | 1  2  88  99 | LETHEFPR |
| 14.1.19 | (Ji’oota 12’n darban keessatti) rakkoon karaa poolisii yookiin mana murtii irraa si mudate turee? | Eeyyee  Lakki  Hin beeku  Deebii hin deebifne/dide/ | 1  2  88  99 | LEPOL |
| 14.1.20 | yoo deebiin gaaffii 10A’f ‘eeyyee’ ta’e, gaaffii itti aanu gaafadhu:  Mudannoon kun kan si mudate erga ulfa taate booda dhaa? | Eeyyee  Lakki  Hin beeku  Deebii hin deebifne/dide/ | 1  2  88  99 | LEPOLPR |
| 14.1.21 | (Ji’oota 12’n darban keessatti) yeroon abbaan manaa kee hojii dhabaa ta’e turee? | Eeyyee  Lakki  Hin beeku  Deebii hin deebifne/dide/ | 1  2  3  88  99 | LEUNEMP |
| 14.1.22 | yoo deebiin gaaffii 11A’f ‘eeyyee’ ta’e, gaaffii itti aanu gaafadhu:  Mudannoon kun kan si mudate erga ulfa taate booda dhaa? | Eeyyee  Lakki  Hin beeku/hin yaadadhu  Deebii hin deebifne/dide/ | 1  2  88  99 | LEUNEMPR |
| 14.1.23 | (Ji’oota 12’n darban keessatti) wanti kan biroon baayyee si jeeqe turee? | Eeyyee  Lakki  Hin beeku  Deebii hin deebifne/dide/ | 1  2  88  99 | LEOTH |
| 14.1.24 | yoo deebiin gaaffii 12A’f ‘eeyyee’ ta’e, gaaffii itti aanu gaafadhu:  Mudannoon kun kan si mudate erga ulfa taate booda dhaa? | Eeyyee  Lakki  Hin beeku  Deebii hin deebifne/dide/ | 1  2  88  99 | LEOTHPR |

## Kutaa 3: Gaaffiilee Fayyaa Dhukubsataa (GFD)-9 fi yaalii

| Hikkaa: guyyaa baayyeef (2-6days) guyyoota walakkaadhaa oliif (7-11days) guyyoota hundaa hanga jechuun danda’amutti (12-14days) 98=hinbekuu | | | | |
| --- | --- | --- | --- | --- |
| Torban lamaan darban keessa, ammam rakkinoota armaan gaditti tarreeffamaniin dhiphatte? | | | | *11^th^ item |
| 1 | Torban lamaan darban keessa, Wantoota hojjechuuf fedhii yookiin gammachuu atti qabdu gadi aanaa ta’uun ammam si mudatee turtee? | Tasumaa/gonkuma | **0** |  |
|  |  | guyyaa baayyeef | **1** |  |
|  |  | guyyoota walakkaadhaa oliif | **2** |  |
|  |  | guyyoota hundaa hanga jechuun danda’amuutti | **3** |  |
| 2 | Torban lamaan darban keessa, gadi aantummaa, gammachuu dhabuu, yookiin abdii dhabuun ammam si mudatee turtee? | Tasumaa/gonkuma | **0** |  |
|  |  | guyyaa baayyeef | **1** |  |
|  |  | guyyoota walakkaadhaa oliif | **2** |  |
|  |  | guyyoota hundaa hanga jechuun danda’amuutti | **3** |  |
| 3 | Torban lamaan darban keessa, rakkoo hiribni si qabuu diiduu/giddu gidduudhaan daddamaquu yookiin hiribni sitti baayyachuun ammam si mudatee turee? | Tasumaa/gonkuma | **0** |  |
|  |  | guyyaa baayyeef | **1** |  |
|  |  | guyyoota walakkaadhaa oiliif | **2** |  |
|  |  | guyyoota hundaa hanga jechuun danda’amuutti | **3** |  |
| 4 | Torban lamaan darbe keessa, miirrri dadhabbii yookiin humna xiqqoo qabaachuu ammam si mudatee turee? | Tasumaa/gonkuma | **0** |  |
|  |  | guyyaa baayyeef | **1** |  |
|  |  | guyyoota walakkaadhaa oliif | **2** |  |
|  |  | guyyoota hundaa hanga jechuun danda’amuutti | **3** |  |
| 5 | Torban lamaan darbe keessa, fedhii nyaataa dhabuu yookiin garmalee nyaachuun ammam si mudatee turee? | Tasumaa/gonkuma | **0** |  |
|  |  | guyyaa baayyeef | **1** |  |
|  |  | guyyoota walakkaadhaa oliif | **2** |  |
|  |  | guyyoota hundaa hanga jechuun danda’amuutti | **3** |  |
| 6 | Torban lamaan darban keessa, waa’ee keetiif miirri hin taane sitti dhaga’amuun- yookiin akka waan kufteetti yookiinimmoo ofiikee yookiin maatii kee akka wanta gadi aansiteetti yaaddee ammam dhiphachaa turtee? | Tasumaa/gonkuma | **0** |  |
|  |  | guyyaa baayyeef | **1** |  |
|  |  | guyyoota walakkaadhaa oliif | **2** |  |
|  |  | guyyoota hundaa hanga jechuun danda’amuutti | **3** |  |
| 7 | Torban lamaan darbe keessa, rakkoo wantootaa xiyyeeffachuu/duukaa bu’uu/ dadhabuu, kan akka maxxansa oduu/gaazexaa/ dubbisuu yookiin televiyinii hordofuu ammam si mudatee turee? | Tasumaa/gonkuma | **0** |  |
|  |  | guyyaa baayyeef | **1** |  |
|  |  | Guyyoota walakkaa oliif | **2** |  |
|  |  | guyyoota hundaa hanga jechuun danda’amuutti | **3** |  |
| 8 | Torban lamaan darban keessa,hanga namoonni biroon sirratti hubatanutti garmalee suuta jettee deemuun yookiin dubachuun yookiin immoo faallaa isaa- haala isa kanan duraa irraa adda ta’een boqonnaa malee garmalee asii fi achi deddemuun ammam si mudatee turee? | Tasumaa/gonkuma | **0** |  |
|  |  | guyyaa baayyeef | **1** |  |
|  |  | Guyyoota walakkaadhaa oliif | **2** |  |
|  |  | guyyoota hundaa hanga jechuun danda’amuutti | **3** |  |
| 9 | Torban lamaan darban keessa, du’uutu naaf wayya yookiin karadhuma ta’een ofii kee irraan miidhaa ga’uuf ammam yaaddee turtee? | Tasumaa/gonkuma | **0** |  |
|  |  | guyyaa baayyeef | **1** |  |
|  |  | guyyoota walakkaadhaa oliiff | **2** |  |
|  |  | guyyoota hundaa hanga jechuun danda’amuutti | **3** |  |
|  | Waliigala gaaffii GFH1-GFD9 | ________ |  | GFD Waligalaa |
| 10 | Torban lamaan kanaan alatti, ji’oota 12’n darban kana keessatti, yeroo mudannoon torbee lamaa fi sana oli ta’e kan gammachuu dhabuu yookiin wantoota baayyeef fedhii dhabuu fi rakkoollee asiin olitti dubbanne baayyeen isaanii si mudatanii turanii? | Lakki  Eeyyee | **0** |  |
|  |  |  | **1** |  |
| *11 | Rakkoolee asiin olitti tuqaman keessaa tokkollee ta'u eyyee deebiin jedhu yoo kenname isa itti aanu gaafadhu.  Rakkoowwan kanneen irraa kan ka'e hojii kee hojjechuun, itti gaafatamummaa manaa kee bahuun yookiin namoota wajiin walii galteen jiraachuun ammam rakkisaa sitti ta'ee ture? | tasuma hin rakkanne | **0** |  |
|  |  | hanga ta'e rakkadheen ture | **1** |  |
|  |  | baay'ee rakkadheen ture | **2** |  |
|  |  | baay'ee badaan rakkadhee ture | **3** |  |

# Mujulii 3: Soorata fi wabii nyaataa duubartii qorannoo kanaaf barbaadamtuu

## Kutaa 1a: Soorata Duubartii Qorannoo Kanaaf Barbaadamtuu- Kan Sa’aa 24 Yaadachuu

Waan kaleessa sooratte, yeroo hirribaa kaatee hanga raftutti sigaafachuuf barbaada. Maaloo nyaataaf dhugaatii kan nama biro waliin qooddatte ta’e isas dabalii natti himi.

| **Lakk** | **Gaafii** | **Deebii** | **Variable** |
| --- | --- | --- | --- |
| 3.1.1 | Guyyaa yaadatte | [Dilbataa- Sanbataa] | RECALLDAY |
| 3.1.2 | Har’a soomaa jirtaa? | 1. eeyyee 2. lakki 3. hin ilaallatu   98. deebisuu didde | IWFASTING |
| 3.1.3 | Guyyoota muraasaaf nyaata hundaa soomaa jirtaa? | 1. eeyyee, hanga guyyaatti 2. eeyyee, hanga sa’aa 9 tti 3. eeyyee, hanga aduun dhiitutti 4. lakki   98. deebisuu didde | IWFASTTIME |
| 3.1.4 | Guyyaan har’aa nyaata yeroo baay’ee nyaattuun walmadaalaa/fakkaataa? | 1. eeyyee 2. lakki   98. deebisuu didde | IWNORMEAT |
| 3.1.5 | Lakki yoo ta’e ibsi:  [waa’ee dhukkubaa, jila,guyyaa ayyaanaa, kkf qoradhu] | (barreessi) | IWNORMEXP |

| **Yeroo guyyaa keessatti**  **(tokko filadhu)** | **Nyaata ykn dhugaatii** | **Ibsi [gosa nyaataa [description]** | **Nyaata akaakuubaay’ee/tokko qofa** | **Nama waliin qooddattee turtee?** | **Kan nama waliin qooddatte ta’e hangamtu qooddame:** | **Hammamtaa nyaata kanaa?**  **(tokko filadhu)** | **Hammamtaa nyaataaf qophaa’ee?**  98 = hin beeku | **Kan nyaatarraa hafe jiraa?** | **Eeyyee yoo ta’e hangamtu hafe?**  98 = hin beeku |
| --- | --- | --- | --- | --- | --- | --- | --- | --- | --- |
| 1=ganama (kaleessa aduun baatee hanga sa’aa 5:30), 2=sa’aa booda (~5:30- aduun dhiitutti),  3= galgala/ halkan (erga aduun dhiitee hanga aduun baatutti ganama)  4. hin yaadadhu | (barreessi) | (barreessi) | 1. nyaata akaakuu heddu  2.tokko qofa  3. hin beeku | 1. nyaata akaakuu heddu  2.tokko qofa  3. hin beeku | \|___\| M/F: <12mo  \|___\| M/F: 12-35mo  \|___\| M/F: 36mo-6yr  \|___\| M/F: 7-9yrs  \|___\| M: 10-12yr \|__\| F: 10-12yr  \|___\| M: 13-15yrs \|__\| F: 13-15yrs  \|___\| M: 16-19yrs \|__\| F:16-19yrs  \|___\| M: ≥20yrs \|__\| F: ≥20yrs | **A1**: biddeena  **A2**: biddeena  **A3** :biddeena  **B1**: Marsaa  **B2**: Marsaa  **B3**: Marsaa  **C1**: Rog-sadee  **C2**: Rog-sadee  **C3**: Rog-sadee  **D1**: Rog-arfee  **D2**: Rog-arfee/ Square  **D3**: Rog-arfee/ Square  **E**: 1 Birrii  **F1**: Cilfaa/Ladle  **F2**: cilfaa/Ladle  **F3**: cilfaa/Ladle  **G1**: xuwwee/Clay pots  **G2**: xuwwee/Clay pots  **G3**: xuwwee/Clay pots  **H0**:burcuqqoo/Glasses  **H1**:burcuqqoo/Glasses  **H2**:burcuqqoo/Glasses  **H3**:burcuqqoo/Glasses  **I1**: faldhaana /Spoons  **I2**: faldhaana /Spoons  **I3**: faldhaana/Spoons  **Pcs SM:** cabaa/Pieces  **Pcs MD:** cabaa/Pieces  **Pcs LG:** cabaa/Pieces | (lakkoofsaan) | 1. eeyyee 2. lakki 3. hin beeku | (lakkoofsaan) |
| 1. |  |  |  |  |  |  |  |  |  |
| 2. |  |  |  |  |  |  |  |  |  |
| 3. |  |  |  |  |  |  |  |  |  |

| Erga sooranni hundinuu barreeffame booda, **IF Column 4 = 1. Gosoota nyaataa** , erga nyaata hundaa barreessitee booda, duubatti deebi’iitii maal akka ofkeessaa qabu gaafadhu | | | |
| --- | --- | --- | --- |
| **Nyaata ykn dhugaatii** | **Maal ofkeessaa akka qabu** (barreeffamaan) | **Model** (same as above) | **Hanga/hamma/Amount** (numeric) |
|  | 1. |  |  |
|  | 2. |  |  |
|  | 3. |  |  |
|  | 4. |  |  |
|  | 1. |  |  |
|  | 2. |  |  |
|  | 3. |  |  |
|  | 4. |  |  |

##

## Kutaa 1b: Haala Soorata Dubartii Qo’annoof Barbaadamtu

Amma Waan kaleessa sooratte, yeroo hirribaa kaatee hanga raftutti sigaafachuuf barbaada. Maaloo nyaataaf dhugaatii kan ofiikeetiif qofa sooratte natti himi.

|  | Kaleessa soorata asii gadii nyaattee? | Kan kunuunsituun daa’imaa nyaatte? | Maddi nyaataa inni guddaan (codes below, skip if did not consume) | Variable name |
| --- | --- | --- | --- | --- |
|  | Midhaan nyaataa |  |  |  |
|  | xaafii (biddeena, marqaa, qixxaa bulluqa, caccabsaa) | 1=eeyyee  0=lakki | DTEFFFS | DTEFF |
|  | Boqqoolloo (nyaata boqqoolloo, marqaa ,shuummoo [nifroo], qorsoo[akaayii], daabboo/qixxaa. | 1=eeyyee  0=lakki | DMAIZEFS | DMAIZE |
|  | Misingaa (nyaata misingaa, marqaa, biddeena, boordee, shuummoo (nifro), qorsoo[akaayii (kolo) | 1=eeyyee  0=lakki | DSORGHUMFS | DSORGHUM |
|  | Garbuu (Marqaa, bulluqa, qorsoo[akaayii (kolo), daabboo (qixxaa) | 1=eeyyee  0=lakki | DBARLEYFS | DBARLEY |
|  | Qamadii (daabboo, marmaree, keekii[cakes) | 1=eeyyee  0=lakki | DWHEATFS | DWHEAT |
|  | Midhaan nyaataa kan biroo | 1=eeyyee  0=lakki | DCEREOTHFS | DCEREOTH |
|  |  |  |  |  |
|  | Midhaan Hiddi /Jirmii / nyaatamu |  |  |  |
|  | Hundee diimaa /Beets | 1=eeyyee  0=lakk | DBEETSFS | DBEETS |
|  | Mixaaxisha – kan haalluu burtukaanaa | 1=eeyyee  0=lakk | DORANGEPOTFS | DORANGEPOT |
|  | Mixaaxisha – haalluu biraa | 1=eeyyee  0=lakk | DOTHERPOTFS | DOTHERPOT |
|  | Dinnicha | 1=eeyyee  0=lakk | DPOTATOFS | DPOTATO |
|  | Godarree/ Yams | 1=eeyyee  0=lakk | DYAMFS | DYAM |
|  | Qooccoo/ Enset | 1=eeyyee  0=lakk | DENSETFS | DENSET |
|  | Kan Hiddi /Jirmii / nyaatamu biraa | 1=eeyyee  0=lakk | DROOTOTHFS | DROOTOTH |
|  |  |  |  |  |
|  | Midhaan Kan akka baaqelaa, atara,shunburaa kkf [Legumes] |  |  |  |
|  | Baaqelaa (boloqqee) | 1=eeyyee  0=lakk | DBEANSFS | DBEANS |
|  | Atara (field, cow, pigeon, shunburaa) | 1=eeyyee  0=lakk | DPEASFS | DPEAS |
|  | Missira /Lentils | 1=eeyyee  0=lakk | DLENTILFS | DLENTIL |
|  | Akurii Atara /Soybeans | 1=eeyyee  0=lakk | DSOBEANFS | DSOBEAN |
|  | Kan biraa | 1=eeyyee  0=lakk | DLEGOTHFS | DLEGOTH |
|  |  |  |  |  |
|  | Sanyii zayitaa |  |  |  |
|  | Talbaa/ Linseeds/Flax | 1=eeyyee  0=lakk | DLINSEEDFS | DLINSEED |
|  | Salixa/ Sesame | 1=eeyyee  0=lakk | DSESAMEFS | DSESAME |
|  | Nuugii/ Niger Seeds/Nug | 1=eeyyee  0=lakk | DNIGERFS | DNIGER |
|  | Kan biraa | 1=eeyyee  0=lakk | DOSEEDOTHFS | DOSEEDOTH |
|  |  |  |  |  |
|  | Fuduraalee /Vegetables |  |  |  |
|  | Raafuu gurraattii (e.g.Moringa/Shiferaw, qosxaa) | 1=eeyyee  0=lakk | DHGREENSFS | DHGREENS |
|  | raafuu (e.g., cabbage) | 1=eeyyee  0=lakk | DLIGGREENFS | DLIGGREEN |
|  | Fuduraalee Vitamin A qaban (kan akka kaarotii, buqqee/dubbaa) | 1=eeyyee  0=lakk | DVITAVEGFS | DVITAVEG |
|  | Timaatima/Tomato | 1=eeyyee  0=lakk | DTOMFS | DTOM |
|  | Qaariyaa/Pepper | 1=eeyyee  0=lakk | DPEPPERFS | DPEPPER |
|  | Qullubbii/Onion/shunkurt/ | 1=eeyyee  0=lakk | DONIONFS | DONION |
|  | Fuduraalee biroo | 1=eeyyee  0=lakk | DVEGOTHFS | DVEGOTH |
|  |  |  |  |  |
|  | Muduraalee/ Fruits (including juices) |  |  |  |
|  | Burtukaana, tiringoo fi loomii/ | 1=eeyyee  0=lakk | DCITRUSFS | DCITRUS |
|  | Muduraalee Vitamin A qaban (paappaayyaa, maangoo) | 1=eeyyee  0=lakk | DVITAFRUFS | DVITAFRU |
|  | Passion fruit, prim, enkoy,  anaanaas, guava | 1=eeyyee  0=lakk | DPASSIONFS | DPASSION |
|  | Abukaadoo/Avocado | 1=eeyyee  0=lakk | DAVOCADFS | DAVOCAD |
|  | Muuza/Banana | 1=eeyyee  0=lakk | DBANANAFS | DBANANA |
|  | Muduraalee biroo | 1=eeyyee  0=lakk | DFRUOTHFS | DFRUOTH |
|  |  |  |  |  |
|  | Foon |  |  |  |
|  | Foon loonii, foon hoolaa, foon | 1=eeyyee  0=lakk | DBEEFFS | DBEEF |
|  | Tiruu / Liver | 1=eeyyee  0=lakk | DLIVERFS | DLIVER |
|  | Foon biro kan akka garaacha, | 1=eeyyee  0=lakk | DBLOODFS | DBLOOD |
|  | Lukkuu/ handaaqqoo/Poultry | 1=eeyyee  0=lakk | DPOUTRYFS | DPOUTRY |
|  | Anqaakuu /killee/ | 1=eeyyee  0=lakk | DEGGSFS | DEGGS |
|  | Qurxummii gogfame | 1=eeyyee  0=lakk | DDRYFISHFS | DDRYFISH |
|  | Fish (all other types) | 1=eeyyee  0=lakk | DFISHFS | DFISH |
|  | Other meats and non-dairy animal products | 1=eeyyee  0=lakk | DMEATOTHFS | DMEATOTH |
|  |  |  |  |  |
|  | Oomishaalee aannanii/Dairy Products |  |  |  |
|  | Milk/annan | 1=eeyyee  0=lakki | DMILKFS | DMILK |
|  | Yoghurt/urgoo/ | 1=eeyyee  0=lakki | DYOGFS | DYOG |
|  | Cheese/ayibii/ittoo sa’aa | 1=eeyyee  0=lakki | DCHEESEFS | DCHEESE |
|  | Kan biraa | 1=eeyyee  0=lakki | DDAIRYOTHFS | DDAIRYOTH |
|  |  |  |  |  |
|  | Cooma/zayita |  |  |  |
|  | Fuduraalee zayitaa (e.g. suufii, Palm, jinjjibila) | 1=eeyyee  0=lakki | DVEGOILFS | DVEGOIL |
|  | Zayita fuduraalee kan jajjaboo ta’e | 1=eeyyee  0=lakki | DVEGFATSFS | DVEGFATS |
|  | Cooma loonii (ghee, dhadhaa) | 1=eeyyee  0=lakki | DANIFATSFS | DANIFATS |
|  |  |  |  |  |
|  | Kan biraa/ Others |  |  |  |
|  | Sukkaara/damma | 1=eeyyee  0=lakki | DSUGARFS | DSUGAR |
|  | Buna, shaayii | 1=eeyyee  0=lakki | DCOFFEEFS | DCOFFEE |
|  | Mi’eessitiita/Condiments | 1=eeyyee  0=lakki | DCONDIMENFS | DCONDIMEN |
|  | Soda, dhugaatii sukkaaraa | 1=eeyyee  0=lakki | DSODAFS | DSODA |
|  | Dhugaatii alkoolii/biiraa/diraaftii (kamiyyuu) | 1=eeyyee  0=lakki | DALCOHFS | DALCOH |

**Koodii maddaa**

1 = oomisha ofii; 2 = gabaa; 3. adamsuu, qurxummii kiyyeessuu, guuruu; 4.kennaa firaa/ollaa; 5.jijjiirraan humna namaa/nyaataan; 6. Gargaarsa nyaataa (e.g. WFP)

## Kutaa 2: Ji’oota Maatiin Nyaata Gahaa Itti Argatan

Amma itti dhiheenya nyaataa maatii keessanii waggaa keessatii kan ji’oota adda addaa sigaafachuun fedha. Gaafilee kanneeniif deebii yoo naafdeebistu, maaloo duubatti kan ji’a 12 yaadadhu, ji’a ammaa kanarraa jalqabii hanga waggaa darbee ji’a kanaatti.

**Gabatee 2.2: yeroo** maatiin nyaata gahaa itti argatan

|  | **Gaafii** | **Deebii** | **Maqaa Var.** |
| --- | --- | --- | --- |
| 2.2.1 | Ji’oota 12n darban keessatti, ji’oonni nyaata gahaa fedhii nyaataa maatii keessan itti guutuu dhabdan jiraa? | 1=eeyyee  0=lakki 98= hinbeeku | SGOOD |
| Eeyyee yoo jette, Ji’oota 12n darban keessatti, ji’oota kam turan yeroon nyaata gahaa fedhii nyaataa maatii keessan itti guutuu dhabdan? | | | |

***Ragaa guuraaf:*** *gaafiin kun nyaata kamiiyyuu madda kamirraayyuuni ilaallata, kan akka ofiin oomishame, bitame/jijjiirrame, nyaata gargaarsaa, ykn liqeeffatame. Ji’oota barreeffaman hindubbisiin. Deebii kennaan ji’oota garagaraa akka yaadatan yoofeete mala lakkaawwii waqtiilee fayyadami. Deebii kennaan waa’ee ji’oota 12n darban hundaa akka yaadu mirkaneessuuf qoradhu.*

|  | **Ji’oota** | **Deebii** |  |
| --- | --- | --- | --- |
| 2.2.2. | Amajjii | 1=eyyee  0=lakki 98=hin beeku | SJAN |
| 2.2.3. | Gurraandhala | 1=eyyee  0=lakki 98=hin beeku | SFEB |
| 2.2.4. | Bitootessa | 1=eyyee  0=lakki 98=hin beeku | SMAR |
| 2.2.5. | Ebla | 1=eyyee  0=lakki 98=hin beeku | SAPR |
| 2.2.6. | Caamsaa | 1=eyyee  0=lakki 98=hin beeku | SMAY |
| 2.2.7. | Waxabajjii | 1=eyyee  0=lakki 98=hin beeku | SJUN |
| 2.2.8. | Adoolessa | 1=eyyee  0=lakki 98=hin beeku | SJUL |
| 2.2.9. | Hagayya | 1=eyyee  0=lakki 98=hin beeku | SAUG |
| 2.2.10. | Fulbaana | 1=eyyee  0=lakki 98=hin beeku | SEPT |
| 2.2.11. | Onkololeessa | 1=eyyee  0=lakki 98=hin beeku | SOCT |
| 2.2.12. | Sadaasa | 1=eyyee  0=lakki 98=hin beeku | SNOV |
| 2.2.13. | Muddee | 1=eyyee  0=lakki 98=hin beeku | SDEC |

## Kutaa 3: Madaallii/Safartuu dhiheennatti argama fi dhabiinsa wabii nyaataa maatii

Torban afran darban keessatti waa’ee dhiheenna nyaata maatiikee irratti gaafii sigaafachuuf deema. Soorata kan akk nyaata idilee, mi’eessitoota fi nyaata kamiyyuu soorata maatii kee keessa jiru.

**Gabatee 3.3: Madaallii/safartuu dhiheennatti argama fi dhabiinsa wabii nyaataa maatii**

|  | **Gaafii** | **Deebii** | **Maqaa Variable** |
| --- | --- | --- | --- |
| 3.3.1 | Torban arfan darban keessatti ati ykn maatiin kee nyaata gahaa hinargannu jechuudhaan sodaattanii beektuu? | 1=eeyyee  0=lakki 98= hin bekuu | WSWORRY |
| 3.3.2 | Eeyyee yoo ta’e, yeroo hangamiif sodaan kun isinmuudate? | 1 = akka tasaa (torban arfan darbanitti al tokko /yeroo lama )  2 = darbee darbee ( torban arfan darbanitti yeroo 3 hand 10tti)  3 = yeroo baay’ee ( torban arfan darbanitti yeroo 10 ol ) | WSWORRYFRQ |
| 3.3.3 | Torban arfan darban keessatti , ati ykn maatiin kee hanqina qabeenyaatiin nyaata nyaachuu feetan osoo hin nyaatiin haftanii beektuu? | 1=eeyyee  0=lakki 98= hin bekuu | WSKIND |
| 3.3.4 | Eeyyee yoo ta’e , yeroo hangamiif wanti kun isinmuudate/qunname? | 1 = akka tasaa (torban arfan darbanitti al tokko /yeroo lama )  2 = darbee darbee ( torban arfan darbanitti yeroo 3 hand 10tti)  3 = yeroo baay’ee ( torban arfan darbanitti yeroo 10 ol ) | WSKINDFRQ |
| 3.3.5 | Torban arfan darban keessatti , ati ykn maatiin kee hanqina qabeenyaatiin kan ka’e gosa nyaataa muraasa qofa nyaattanii? | 1=Yes  0=No 98= hin bekuu | WSLIMITED |
| 3.3.6 | Eeyyee yoo ta’e , yeroo hangamiif wanti kun isinmuudate/qunname? | 1 = akka tasaa (torban arfan darbanitti al tokko /yeroo lama )  2 = darbee darbee ( torban arfan darbanitti yeroo 3 hand 10tti)  3 = yeroo baay’ee ( torban arfan darbanitti yeroo 10 ol ) | WSLIMITEDFRQ |
| 3.3.7 | Torban arfan darban keessatti , ati ykn maatiin kee hanqina qabeenyaatiin kan ka’e akaakuu nyaata biroo argachuu dhabuun nyaata nyaachuu hinfeene nyaattanii turtanii? | 1=eeyyee  0=lakki 98= hin bekuu | WSDISLIKE |
| 3.3.8 | Eeyyee yoo ta’e , yeroo hangamiif wanti kun isinmuudate/qunname? | 1 = akka tasaa (torban arfan darbanitti al tokko /yeroo lama )  2 = darbee darbee ( torban arfan darbanitti yeroo 3 hand 10tti)  3 = yeroo baay’ee ( torban arfan darbanitti yeroo 10 ol ) | WSDISLIKEFRQ |
| 3.3.9 | Torban arfan darban keessatti , ati ykn maatiin kee nyaatni gahaan waan hinjirreef nyaata nyaachuu feetan gaditti nyaattanii turtanii? | 1=eeyyee  0=lakki 98= hin bekuu | WSMALL |
| 3.3.10 | Eeyyee yoo ta’e, yeroo hangamiif wanti kun isin muudate/qunname? | 1 = akka tasaa (torban arfan darbanitti al tokko /yeroo lama )  2 = darbee darbee ( torban arfan darbanitti yeroo 3 hand 10tti)  3 = yeroo baay’ee ( torban arfan darbanitti yeroo 10 ol ) | WSMALLFRQ |
| 3.3.11 | Torban arfan darban keessatti , ati ykn maatiin kee nyaatni gahaan waan hinjirreef guyyaatti nyaata bicuu nyaattanii turtanii? | 1=eeyyee  0=lakki 98= hin bekuu | WSFEW |
| 3.3.12 | Eeyyee yoo ta’e , yeroo hangamiif wanti kun isin muudate/qunname? | 1 = akka tasaa (torban arfan darbanitti al tokko /yeroo lama )  2 = darbee darbee ( torban arfan darbanitti yeroo 3 hand 10tti)  3 = yeroo baay’ee ( torban arfan darbanitti yeroo 10 ol ) | WSFEWFRQ |
| 3.3.13 | Torban arfan darban keessatti , ati ykn maatiin kee hanqina qabeenyaa nyaata ittin argatturraa kan ka’e yeroo waan nyaatamu kamiyyuu itti dhabdan jiraa? | 1=eeyyee  0=lakki 98= hin bekuu | WSNOFOOD |
| 3.3.14 | Eeyyee yoo ta’e , yeroo hangamiif wanti kun isin muudate/qunname? | 1 = akka tasaa (torban arfan darbanitti al tokko /yeroo lama )  2 = darbee darbee ( torban arfan darbanitti yeroo 3 hand 10tti)  3 = yeroo baay’ee ( torban arfan darbanitti yeroo 10 ol ) | WSNOFOODFRQ |
| 3.3.15 | Torban arfan darban keessatti , ati ykn maatiin kee nyaatni gahaan waan hinjirreef halkan beela’aa raftanii beektuu? | 1=eeyyee  0=lakki 98= hin bekuu | WSLEEP |
| 3.3.16 | Eeyyee yoo ta’e , yeroo hangamiif wanti kun isin muudate/qunname? | 1 = akka tasaa (torban arfan darbanitti al tokko /yeroo lama )  2 = darbee darbee ( torban arfan darbanitti yeroo 3 hand 10tti)  3 = yeroo baay’ee ( torban arfan darbanitti yeroo 10 ol ) | WSLEEPFRQ |
| 3.3.17 | Torban arfan darban keessatti , ati ykn maatiin kee nyaatni gahaan waan hinjirreef halkaniif guyyaa tokko guutuu osoo nyaata kamiyyuu hin nyaatiin dabarsitanii beektuu? | 1=eeyyee  0=lakki 98= hin bekuu | WSNODAY |
| 3.3.18 | Eeyyee yoo ta’e , yeroo hangamiif wanti kun isin muudate/qunname? | 1 = akka tasaa (torban arfan darbanitti al tokko /yeroo lama )  2 = darbee darbee ( torban arfan darbanitti yeroo 3 hand 10tti)  3 = yeroo baay’ee ( torban arfan darbanitti yeroo 10 ol ) | WSNODAYFRQ |

## Kutaa 4: Nyaata Yeroo Ulfaa Fi daa’ima Hoosisan Laguu ta’an

|  |  | **Gaafii** | | | | **Yeroo Ulfaa** | | **Yeroo harma hoosiftu?** | | **Variable name** | |
| --- | --- | --- | --- | --- | --- | --- | --- | --- | --- | --- | --- |
| 3.4.1 |  | Naannoo keessanitti barmaatileen aadaa nyaata muraasa ykn gosa nyaataa dubartii ulfaa fi harma hoosistuuf dhoorku jiraa ? | | | | 1=eeyyee  0=lakki  98= hinbeeku | | 1=eeyyee  0=lakki98= hinbeeku | | FDTABOBF  FDTABOLAC | |
| **3.4.2** | **Eeyyee yoo ta’e, maaloo nyaata ykn akaakuu nyaataa lagataman/dhoorkaman tarreessi (hanga danda’meen maqaa nyaatichaa himi):** | | | | | | | | | | |
| **Akaakuu nyaataa** | **Yeroo ulfaa keessaa yeroo kam dhoorkaman?** | | **Maaliif dhoorkame?** | **yeroo daa’ima harma hoosisanis ni dhoorkamaa?** | **Yoo eyyee ta’e, Maaliif dhoorkame?** | | **Yeroo hinulfaa’in nyaata kana ni nyaattaa?** | | **Nyaata kana yeroo ulfaa/harma hoosiftu ni nyaattaa?** | | **Nyaata kana yeroo ulfaa ni dharraataa/barbaaddaa?** |
| 1. __________ | 1. Ulfa taatee yeroo hundaa 2. Jalqabarratti/jalqaba ulfaarratti 3. dhumarratti/xumurarratti 4. jidduutti 5. hin ilaallatu 6. hin beeku | | 1.ciniinsuun nijabaata jedhanii sodaachuu [ulfi ni guddata jedhanii sodaachuu]  2. bifti daa’imaa hintoluuf jedhanii sodaachuu  3. ulfi namarraa baha jedhanii sodaachuun  4. kanbiraa: (ibsi  __________)  5.hin beeku | 1=eeyyee  2=lakki  3. Hin beeku | ibsi _______ | | 1= yeroo hundaa  2= yeroo tokko tokko  3= akka tasaa  4 = gonkumaa  98=hin beeku | | 1=yeroo hundaa  2= yeroo tokko tokko  3= akka tasaa  4 =gonkumaa  98= hin beeku | | 1=eeyyee  2=lakki  98= hin beeku |
| FTPTYPE1 | FTPTYP1D | |  | FTPTYP1BF | FTPTYP1W / FTPTYP1WS | | FTPTYP1NC | | FTPTYP1PC | | FTPTYP1CR |
| 2. __________ | 1. Ulfa taatee yeroo hundaa 2. Jalqabarratti/jalqaba ulfaarratti 3. dhumarratti/xumurarratti 4. jidduutti 5. hin ilaallatu 6. hin beeku | | 1.ciniinsuun nijabaata jedhanii sodaachuu[ulfi ni guddata jedhanii sodaachuu]  2. bifti daa’imaa hintoluuf jedhanii sodaachuu  3. ulfi namarraa baha jedhanii sodaachuun  4. kanbiraa: (ibsi  __________)  5.hin beeku | 1=eeyyee  2=lakki  3. Hin beeku | Ibsi______ | | 1= yeroo hundaa  2= yeroo tokko tokko  3= akka tasaa  4 = gonkumaa  98=hin beeku | | 1=yeroo hundaa  2= yeroo tokko tokko  3= akka tasaa  4 =gonkumaa  98= hin beeku | | 1=eeyyee  2=lakki  98= hin beeku |
| FTPTYPE2 | FTPTYP2D | |  | FTPTYP2BF | FTPTYP2W / FTPTYP2WS | | FTPTYP2NC | | FTPTYP2PC | | FTPTYP2CR |
| 3. __________ | 1. Ulfa taatee yeroo hundaa 2. Jalqabarratti/jalqaba ulfaarratti 3. dhumarratti/xumurarratti 4. jidduutti 5. hin ilaallatu 6. hin beeku | | 1.ciniinsuun nijabaata jedhanii sodaachuu  2. bifti daa’imaa hintoluuf  3. ulfi namarraa baha jedhanii sodaachuun  4. kanbiraa: (ibsi  __________)  5.hin beeku | 1=eeyyee  2=lakki  3. Hin beeku | Ibsi____ | | 1= yeroo hundaa  2= yeroo tokko tokko  3= akka tasaa  4 = gonkumaa  98=hin beeku | | 1=yeroo hundaa  2= yeroo tokko tokko  3= akka tasaa  4 =gonkumaa  98= hin beeku | | 1=eeyyee  2=lakki  98= hin beeku |
| FTPTYPE3 | FTPTYP3D | |  | FTPTYP3BF | FTPTYP3W / FTPTYP3WS | | FTPTYP3NC | | FTPTYP3PC | | FTPTYP1CR |

|  | **Gaafii** | | **Deebii** | | **Variable** | |
| --- | --- | --- | --- | --- | --- | --- |
| 3.4.3 | Nyaatni biroo yeroo harma hoosisan dhoorkamu kan biroojiraa? | | 1.____________  2.____________  3____________ | | FTBF1  FTBF2  FTBF3 | |
| 3.4.4 | Naannoo keetitti barmaatileen dubartii ulfa taate ykn harma hoosiftu gosa nyaata irra caalaa akka nyaattu jajjabeessu jiraa ? | | 1=eeyyee  0=lakki  98= hin beeku | | FDTABMORE | |
| **3.4.5** | **Eeyyee yoo jette, maaloo nyaata /gosoota nyaataa akka sooratamaniif jajjabeeffaman naaf ibsi:** | | | | |  |
| **Nyaata/gosa yaataa** | **Yoom akka nyaatamuuf jajjabeefama?** | **Maaliif akka nyaatamu jajjabeeffaman?** | | **Nyaani kun Yeroo kamiiyyuu ni nyaatamaa?** | |  |
| _________ | 1. Ulfa taatee yeroo hundaa 2. Jalqabarratti 3. dhumarratti/xumurarratti 4. yeroo harma hoosistu | 1. da’uumsaaf akka haala mijeesuuf  2. daa’imni du’e akka hin dhalanne dhoorkuuf  3. daa’imni garaa keessatti akka garmalee hin guddanee/furdanneef  4.daa’ima fayya qabeessa akka ta’uuf  5.fayyaa haadhaa akka fooyyesuuf  6. kan biro ibsi______ | | 1=eeyyee  2=lakki | |  |
| FTETYPE1 | FTETYP1D | FTETYP1W | | FTETYP1NC | |  |
| 2.  ________ | 1. Ulfa taatee yeroo hundaa 2. Jalqabarratti 3. dhumarratti/xumurarratti 4. yeroo harma hoosistu | 1. da’uumsaf akka haala mijeesuuf  2. daa’imni du’e akka hin dhalanne dhoorkuuf  3. daa’imni garaa keessatti akka garmalee hin guddanee/furdanneef  4.daa’ima fayya qabeessa akka ta’uuf  5.fayyaa haadhaa akka fooyyesuuf  6. kan biro ibsi___ | | 1=eeyyee  2=lakki | |  |
| FTETYPE2 | FTETYP2D | FTETYP2W | | FTETYP2NC | |  |

|  | **Gaafii** | **Deebii** | **Variable name** |
| --- | --- | --- | --- |
| 3.4.6 | Erga ulfa taatee booda baay’inni nyaanni soorattu hagam jijjiirame ? | 1. kanduraanirra/kanbaramerra caalaa sooradha 2. kanduraanirra/kanbaramerraa gadiin sooradha 3. baay’inni nyaata sooradhuu hin jijjiirramne 4. hinbeeku | FTAMTCHANG |
| 3.4.7 | Erga ulfa taatee booda gosti nyaataa ati soorattu hagam jijjiirame ? | 1. kanduraanirra akaakuu nyaataa heddu nyaadha 2. nyaata dhoorkaman waan hin sooranneef kanduraanirra gasa nyaataa bicuun sooradha 3. gosuma nyaataa kanaan dura nyaadhu nyaadha [jijjiirramni hinjiru] 4. hinbeeku | FTVARCHG |

## Kutaa 5: Caatii Qama’uu

| **3.5.1** | Caatii qamaata? | Eeyyee/Lakki/dide(yoo lakki ta’e Kutaa kana irra darb) | **WCHATCH** |
| --- | --- | --- | --- |
| **3.5.2** | Eyee yoo ta’e, hangam qamaata? | Guyyaatti,ji’aatti,woggatti yeroo_________ | WCHATOFT |
| **3.5.3** | Yeroo tokkotti hangam qamaata | Zoorbaa(bundles)______________ | WCHATQWANT |
| **3.5.4** | Gabaadhaa bitte moo kan ofii oomishte qamaata? | 1.Kan ofii oomishe irraa qofa  2. bituudhaan qofa qama’a  3. lachuu(bittaaf oomisha)  4. kennaa  5. kan biroo[ibsi] | WCHATSRC |
| **3.5.5** | Osoo hin qamaaneen dura caatii niqulqulleesitaa? | Eeyyee/Lakki/hin beeku | WCHATCLEAN |

# Mujulii 14 -Madaallii sadarkaa xinsammuuf hawaasummaa

## Kutaa 6:- Madallii deeggarsa Hawaasaa Haadholii (MWHH)

Waa’ee deeggarsa namoota garagaraa irraa argattuu si gaaffachuu barbaada. Tokkoon tokkoon himoota armaan gaditti caqafamaniif mee waa’ee gargarsa argattuuf wanti sitti dhaga’amu ammam akka ta’e ni agarsiisa kan jettu ilaalcha qabdu kenni.

| **Lakk.** | **Gaaffilee** | **5= yeroo hundaa 4 = yeroo baayyee**  **3 = yeroo tokko tokko 2 = akka tasaa 1 = Tasuma 98=hinbekuu** |
| --- | --- | --- |
| 1 | Hiriyoota gaarii ta’an kan na deeggaran nan qaba | **1 2 3 4 5** |
| 2 | Maatiin koo yeroo hundaa na bukkee/faana/ ni dhaabatu | **1 2 3 4 5** |
| 3 | Abbaan manaa/Hiriyaan/ koo baayyee na gargaara | **1 2 3 4 5** |
| 4 | Abbaa manaa/hiriyaa/ koo waliin wal-dhabiinsa/ lola/ nan qaba | **1 2 3 4 5** |
| 5 | Abbaa manaa/Hiriyaa/ kootiin akkaan to’atamuun/hordofamuun/ natti dhaga’ama | **1 2 3 4 5** |
| 6 | Abba manaa/hiriyaa/ kootiin akkaan jallatamuun natti dhaga’amaa? | 1. **2 3 4 5** |

## Kutaa 7:- Meeshaa HITS kan hubama/miidhaa/ hiriya dhihoo kan qoru

Mee tokkoon tokkoo gochaawwan armaan gadii dhaggeffachuun baayinni/irra deddeebiin/ Hiriyaan/gargaaran/ kee gochaawwan kan si irran ga’u ammam akka ta’e haala armaan gadiitti mulifameen agarsiisi.

1=tasuma 2=akka tasaa 3=si’a tokko tokko 4=yeroo baayyee 5=irra caalaa/irra deddebiidhaan/ 98=hinbekuu

|  | **Gaaffilee** | **Qabxii** | **Koodii** |
| --- | --- | --- | --- |
| Hiriyaan kee ammam | | **1 2 3 4 5** |  |
| 1 | miidhaa qaamaa si irraan ga’a | **1 2 3 4 5** |  |
| 2 | si arrabsa yookiin si ceepha’a | **1 2 3 4 5** |  |
| 3 | si miidhuuf si sosodaachisa | **1 2 3 4 5** |  |
| 4 | sitti iyya yookiin si abaara | **1 2 3 4 5** |  |

# Mujulii 4: Ulfaa Fi Haala FayyaaDubartii Qorannoof barbaadamtuu

## Kutaa 1: Gamaaggama/Qorannaa Haala Fayyaa

Kanatti aansee gaafilee Waa’ee fayyaa keetii sigaafachuuf deema. Maaloo gaafilee itti aanuuf deebii deebisi.

|  | **Gaafii** | | **Deebii** | | **Variable name** |
| --- | --- | --- | --- | --- | --- |
| 4.1.1 | Torban lamaan darban keessatti, dhukkubni kamiiyyuu haata’usidhukkubee turee? (yoo lakki jette, gara kutaa 4.1.6 tti darbi ) | | 1=eeyyee  0=lakki 98= hinbeeku | | MANYILL |
| 4.1.2 | Eeyyee yoo jette, Tarban lamaan darban keessatti sababadhukkubaatiin guyyootameeqaaf hojii idileekee kan hojjachuu dadhabde? | | ___guyyaa (walakkaa guyyaas haata’u) | | MDAYS |
|  | Maaloo gaafii sadeen kanaa gadii waa’ee dhukkuba yeroo xumuraa si qabe naa deebisi: | | | | |
| 4.1.3 | Gargaarsa manaan alatti barbaaddee turtee? *(yoo lakki jette, gara gaafii 4.1.6 darbi)* | 1=eeyyee  0=lakki 98=hinbekuu | | MTREAT | |
| 4.1.4 | Yoo eeyyee jette, gargaarsa eessaa barbaadde? | - - 1. =ogeessa aadaa     2. = xabala     3. =falfala/tolchaa     4. =hojjattuu ekisteenshinii fayyaa     5. = hawaasaa keessatti nama sooressa (raabsaa qorichaa)     6. =mana qorichaa ykn faarmaasii dhuunfaa     7. =kilinika dhuunfaa     8. =dhaabilee fayyaa tolaan tajaajilan[bu’aaf hinhojjanne]     9. =dhaabbilee fayyaa hawaasaa (B/fayyaa, Hospitaala) | | MTREATWHERE | |
| 4.1.5 | Dhaabbanni fayyaa ati gargaarsa barbaachaaf deemte hagam fagaata? (kiiloo meetiraan) | kiiloo meetira _____ 98= hinbeeku | | MDISTANCE | |
| 4.1.6 | Halkan darbe saaphana siree jala raftee? | 1=eeyyee  0=lakki 2= saaphana sireeyyuu hin qabu 98= hinbeeku | | MNETCGIVER | |

## Kutaa 3: Ulfa kanaan Duraa

| 4.3.1 | Hanga ammaatti yeroo meeqa ulfoofte? *(ulfa ammaa kana dabalatee)* | yeroo____ ulfaaye  *yoo homaa hinulfoofne ta’e gara kutaa 4tti darbi*  **98= hin beeku** | MPREGNA |
| --- | --- | --- | --- |
| 4.3.2 | Hanga ammaatti daa’mman lubbuudhaan jiran/ dhalatan meeqa qabda? | Daa’imman______  **98= hin beeku** | MALIVEBORN |
| 4.3.3 | Daa’imni sijalaa du’ee beekaa (du’aniikandhalata dabalatee)? | 1=eeyyee  0=lakki **98= hin beeku** | MCHILDIE |
| 4.3.4 | Eeyyee yoo jette, hangam? | Daa’imman______ **98= hin beeku** | MNOCHILDIE |
| 4.3.5 | Ulfi sirraa bahuudhaan daa’ima dhabdee beektaa (otoo hinyaadiin ulfi namarraa bahuu)? | 1= eeyyee 2= lakki **98= hin beeku** | MMISCARRY |
| 4.3.6 | Eeyyee yoo jette, hangam? | Daa’imman______ | MNOMISCARY |
| 4.3.7 | Ulfi kee kan dhumaa yoom ture ? | waggaa_____________ **98= hin beeku** | MLASTPRG |
| 4.3.8 | Yeroo ulfa kee isa boodaa si’a meeqa kunuunsa da’uumsaan duraa argatte? | Si’a ________,yoo hjin jiruu ta’e 0 baressi  **98= hin beeku** | MNOANC |
| 4.3.9 | Yeroo ulfa sanaa, kiniina ayiranii [iron] siifkennamee ykn bitattee? (kiniinicha itti muldhisi/ittigarsiisi) | 1=eeyyee  0=lakki**98= hin beeku** | MANCIRON |
| 4.3.10 | Eeyyee yoo jette, yeroo ulfaa sana hunda keessatti, kiniinicha guyyoota meeqaaf fudhatte? | guyyoota____  **98= hin beeku** | MIRONDAYS |
| 4.3.11 | Yeroo ulfa kee isa boodaa, qoricha raammoo garaa fudhattee turtee? | 1=eeyyee  0=lakki**98= hin beeku** | MTREATWORM |
| 4.3.12 | Yeroo ulfa kee isa boodaa, qorannoon HIV siif godhamee turee? | 1=eeyyee  0=lakki**98= hin beeku** | MHIVTEST |
| 4.3.13 | Yeroo ulfa kee isa boodaa, waldhaansi farra HIV fudhattee turtee? | 1=eeyyee  0=lakki**98= hin beeku** | MART |
| 4.3.14 | Bu’aan/carraan ulfa kee boodaa maalture?  *(filannoo hindubbisiinif, deebii kennaan akka deebisu godhi)* | 1. lubbuudhaan dhalate 2. du’ee dhalate  3. ulfi narraa bahe 4. beekaatuma ofirraa baasise 98= hin beeku | MPREGOUT |
| 4.3.15 | Daa’ima kee isa boodaa, eessatti deesse? | 1. Dhaabbilee fayyaa hawaasaa /mootummaa. 2. dhaabbilee fayyaa bu’aaf hin hojjanne/miti mootummaa 3. dhaabbilee fayyaa dhuunfaa 4. deessistuu aadaa [TBA]  5. manatti (manakootitti/mana nama biroo)  6. kanbiraa (ibsi__________) | MDELIVER  MDELIVSPE |
| 4.3.16 | Ulfa kee isa boodaa garaa baqaqsanii hodhuudhaan deessee? | 1=eeyyee  0=lakki98= hin beeku | MCAESAR |
| 4.3.17 | Ulfa kee isa boodaa, qadhaabaadhaan [forceps] deessee? | 1=eeyyee  0=lakki98= hin beeku | MFORCEP |
| 4.3.18 | Dhiibbaa dhiigaa ulfaa’urraan dhufu sirratti argamee beekaa? | 1=eeyyee  0=lakki98= hin beeku | MPREGHYPT |

## Kutaa 4: Ulfa Ammaa

| 4.4.1 | Ati Amma ulfaa? | 1=eeyyee  0=lakki 98= hinbeeku | MCURPREG |
| --- | --- | --- | --- |
| 4.4.2 | Yoo lakki jette, ulfa kee maaltu addaan kute/maaltu muudate? | 1. osoo hingahii /du’ee dhalate 2. ulfi narraa bahe 3. beekaatuma ofirraa baasise  98= hinbeeku | MPREGTERM |
| 4.4.3 | Eeyyee yoo jette, torbaan meeqaaf ulfaan turte? | torbaan__________ 98= hinbeeku | MPREGMOS |
| 4.4.4 | Hanga ammaatti kunuunsa da’uumsa duraa argatte? | 1=eeyyee  0=lakki 98= hinbeeku | MNOANCC |
| 4.4.5 | Ulfa kanaaf Hanga ammaatti kunuunsa da’uumsa duraa meeqa yeroo meeqa fudhattee beekta? | Yeroo ________b 98 = hin beeku | MNOANCC |
| 4.4.6 | Yeroo ulfa kanaa, kiniinii ayiranii[iron] siif kennamee/bitattee turtee? (kiniinicha itti argisiisi) | 1=eeyyee  0=lakki 98= inbeeku | MANCIRONC |
| 4.4.7 | Eeyyee yoo jette, yeroo ulfa kanaa hanga ammaatti kiniinicha kan fudhatte guyyoota meeqaaf ? | guyyoota____  98= hinbeeku | MIRONDAYSC |
| 4.4.8 | Eeyyee yoo jette, kiniinicha fudhachuu hanga deessutti itti fuftaa? | 1=eeyyee 0= lakki  98= hinbeeku | MIRONRESTDAYS |
| 4.4.11 | Yeroo ulfa kanaa, qoricha raammoo garaa fudhattee turtee? | 1=eeyyee  0=lakki 98= hin beeku | MTREATWORMC |
| 4.4.12 | Yeroo ulfa kanaa, qorannoon HIV siif godhamee turee? | 1=eeyyee  0=lakki 98= hinbeeku | MHIVTESTC |
| 4.4.13 | Yeroo ulfa kee isa kanaa, qorannoon HIV siif godhamee turee? | 1=eeyyee  0=lakki 98= hinbeeku | MARTC |

# Mujulii 14 -Madaallii sadarkaa xinsammuuf hawaasummaa

## Kutaa 4 :-Madaalli amala muddannoowwan sodaachuu abbaa qabxii ja’a (six item state trait anxiety scale)

*Amma yeroo kana wanta sitti dhaga’ama jiru baruun barbaada, kana jechuunis,battala kanatti. Yeroon gaaffilee kana siif dubbisi na dhaggeeffachuun battala amma kanaa miirri sun sitti dhaga’amaa jiraachuu isaa yookiin dhiisuu isaa akkasumas miirri kun hangam tokko akka sitti dhaga’amaa jirus itti dabaluun akka natti himtu kabajaanin si gaafadha. Gaaffii kamifiyyuu deebii kee yeroo deebsitu yeroo dheeraa hin fudhatni haa ta’u malee miira yeroo ammaa sitti dhaga’amaa jiruuf deebii sirritti ni ibsa jeettu kenni.*

**1. Tasuma 2. Hamma tokko 3.jiddu galeessaan 4.baay’ee 5.*98= hinbekuu***

| **Lakk** | **Gaaffilee** | **Qabxii** | | | | **Koodii** |
| --- | --- | --- | --- | --- | --- | --- |
| 1 | Amma yeroo kana tasgabbiin/sabriin/ ammam sitti dhaga’amaa jiraa? | **1** | **2** | **3** | **4** |  |
| 2 | Amma yeroo kana dhipachuun ammam sitti dhaga’amaa jiraa? | **1** | **2** | **3** | **4** |  |
| 3 | Amma yeroo kana ariin ammam sitti dhaga’amaa jira? | **1** | **2** | **3** | **4** |  |
| 4 | Amma yeroo kana ammam sitti tolaa jiraa? | **1** | **2** | **3** | **4** |  |
| 5 | Amma yeroo kana ammam gammachuu qabdaa? | **1** | **2** | **3** | **4** |  |
| 6 | Amma yeroo kana ammam si yaaddessaa jira? | **1** | **2** | **3** | **4** |  |

## Kutaa 5: Sodaa ulfa waliin wal-qabate

Kanneen armaan gadii sodaawwan bebbekamoo dubartootni ulfaa kanaan dura(ergasii) himachaa turan keessaa yartuun dha. Dubartiin akkamiyyuu sodaawwan kana hunda al-tokkicha qabaachuun dirqama miti. Dubartootni tokko tokko tasuma qabaachuu dhiisuu danda’u. Mee deebii kee amma sii danda’ametti amanamummaadhaan naaf deebisi. sodaan kun sadarkaan/guddinni/ isaa ammam akka ta’e sirritti ibsuun akkamitti akka danda’amu yoo siif galuu baate illee, battalumaan murteessuun (osoo hin rakkatin) wanta sirrii sitti fakkaate himi.

**1.Tasuma 2. akka tasaa 3. yeroo tokko tokko 4. si’a baay’ee /irra caalaa 5. Badaa 6. *98= hinbekuu***

|  | Gaaffilee | qabxii | | | | | Variable |
| --- | --- | --- | --- | --- | --- | --- | --- |
| 1 | Waa’ee dhukkubbi ciniinsuu fi dhukkubbi yeroo da’umsaa ammam baayyistee yaaddoftaa? | **1** | **2** | **3** | **4** | 5 |  |
| 2 | Sababa ergasii deessee hin beekneef waa’ee da’umsaa ammam sodaachaa jirtaa? | **1** | **2** | **3** | **4** |  |  |
| 3 | Waa’ee yeroo ciniinsuu of-to’achuu dadhabuu fi badaan iyya jette sodaachuu ammam yaadda’aa jirtaa? | **1** | **2** | **3** | **4** |  |  |
| 4 | Daa’imichi hirina sammuu ni qabata yookiin miidhama sammuu ni qabaata jette ammam sodaachaa jirtaa? | **1** | **2** | **3** | **4** |  |  |
| 5 | Daa’imichi du’aa ta’ee dhalata, yookiin da’umsa irratti yookiin akkuma dhalateen du’a jettee ammam sodaachaa jirtaa? | **1** | **2** | **3** | **4** |  |  |
| 6 | Daa’imichi hirina qaamaa ni qabaata jettee ammam sodaachaa jirta yookiin immoo qaamni daa’imichaa wanta sirrii hin taane tokko ni qabaata jettee ammam yaada’aa jirtaa? | **1** | **2** | **3** | **4** |  |  |
| 7 | Daa’imni kee fayyaa ni dhaba yookiin dhibeen ni qabama jette ammam yaaddoftaa? | **1** | **2** | **3** | **4** |  |  |
| 8 | Erga deessee booda boci qaama keetii akka kanaan duraatti hin deebi’u jettee ammam yaadda’aa jirtaa? | **1** | **2** | **3** | **4** |  |  |
| 9 | Qaamni koo kan nama hin hawwanee ta’a jettee ammam dhimmamaa jirtaa? | **1** | **2** | **3** | **4** |  |  |
| 10 | Ulfaatinni qaama kootii badaa dabala jettee ammam yaadda’aa jirtaa? | **1** | **2** | **3** | **4** |  |  |
| 11 | Yeroo da’aumsa yookin baattala da’umsa booda Nan du’a jettee hangam takka soddaatta? | **1** | **2** | **3** | **4** | **5** |  |
| 12 | Da’umsa booda na dhukkuba yookin dhukkuba itin saaxilama jettee hangam takka sodaattha? | **1** | **2** | **3** | **4** | **5** |  |

# MOdujulii 15: Hordoffii Beekumsa Nyaataa

**Ragaa Sassaabaaf/duuf:** Himoota asii gadii hundaaf debii kennaaf dubbisiitii yoo himoonni hundinuu dhugaa ta’an 0= ykn1=yoo dhuga hin tanee sittiyaahimu fi deebii waraabii

**Gaafilee waa’ee soorataa fi beekumsa fayyaa**

| **Lakk.** | **Gaafii** | **Deebin gafatamma yoo dhugaa ta’e** | **Deebin gafatamma yoo soba ta’e** | **Hinbeeku** |
| --- | --- | --- | --- | --- |
| **15.1.1** | **Bodenna shirodhan yeroo hundaa nyachun fakenya nyaata adda adda nyaachuutti. Deebii : lakki** | 0 | 1 | **98** |
| **15.1.2** | **Dubartini ulfaa guyya si’a sadii nyaachuun fayya fi gudinna dai’ima gara kessa jiruuf gahadha. Deebii : lakki** | 0 | 1 | **98** |
| **15.1.3** | **Da’ima akka dhalatenni sa’a tokko kessatti harama jalqabsissittu moo, sa’a 12 kessatti harma jalqabsissittu gardha Deebii: sa’a tokko kesatti** | 0 | 1 | **98** |
| **15.1.4** | **Harmaa hosissun qofiti hangaa waga shanitti da’imaffi nyaata barbachiso hundaa argamisissa? Deebii: lakki** | 0 | 1 | **98** |
| **15.1.5** | **Hatti nyaata dabalatta da’ima isheettif umirri meqatti jalqabisisu qabdi? Deebii:ji’a ja’a** | 0 | 1 | **98** |

# Mujulii 14 -Madaallii sadarkaa xinsammuuf hawaasummaa

## Kutaa 2: Madaallii sadarkaa dhiphinaa/yaaddoo

Gaaffiileen armaan gadii yaadotaaf miira ji’a darbe keessa kessakee deddeebi’aa turaniif akkasumas sitti dhaga’amaa turan adda baasuu irratti xiyyeeffatu. Gaaffilee akkaataa ati itti yaadduu(yaada) fi sitti dhaga’amu(miira) sigaafataniif filannoowwan siif kennaman keessaa filadhuu deebisi.

1. **= Tasuma/gonkuma 1 = akka tasaa 2 = yeroo tokko tokko 3 = deddeebi’ee 4 = si’a baayyee/irra caalaa/ 98=hin beeku**

|  | Gaaffilee | **Filannoo** |
| --- | --- | --- |
| **1** | Ji’a darbe kana keessa, sababa wanti ati hin yaadne/tilmaamne/ si mudateef ammam jeeqamtee/mufattee/ beektaa? | **0 1 2 3 4** |
| **2** | Ji’a darbe kana keessa, wantoota jireenya kee keessatti barbaachisoo ta’an to’achuu akka dadhabuun ammam sitti dhaga’amee turee? | **0 1 2 3 4** |
| **3** | Ji’a darbe kana keessa, sodaa fi dhiphinni ammam sitti dhaga’amee turee? | **0 1 2 3 4** |
| **4** | Ji’a darbe kana keessa, dandeettii rakkoo dhuunfaa kee hiikuuf qabdu ilaalchisee ofitti amanamummaan ammam sitti dhaga’amee turee? | **0 1 2 3 4** |
| **5** | Ji’a darbe kana keessa, wantootni akka/haala/ yaaddetti siif adeemuun isanii ammam sitti dhaga’amee turee? | **0 1 2 3 4** |
| **6** | Ji’a darbe kana keessa, wantoota/haalota/ dandamachuu sirra ture hundaa ammam dandamachuu dadhabdee turtee? | **0 1 2 3 4** |
| 7 | Ji’a darbe kana keessa, rakkina jireenyaa simudate to’achuu danda’uun ammam sitti dhaga’ame turte? | **0 1 2 3 4** |
| 8 | Ji’a darbe kana keessa, wantoota hunda irra aanuu danda’uun ammam sitti dhaga’amee ture? | **0 1 2 3 4** |
| 9 | Ji’a darbe kana keessa, sababaa wantootni to’annaa keetiin ala ta’an irraan kan ka’e ariin ammam sitti dhaga’amee turee? | **0 1 2 3 4** |
| 10 | Ji’a darbe kana keessa, wantootni rakkisaa ta’an hanga mo’achuu dadhabuutti gahan natti danataniiru kan jedhu ammam sitti dhaga’amee turee? | **0 1 2 3 4** |

# Mujulii 9: Koornayaa Fi Murtee Kennuu

## Kutaa 1: Itti Dhiheenna, Abbummaa , Fi Too’annoo Oomisha Qonnaa

Amma gaafilee muraasa waa’ee kan maatii kee ilaallaatu sigaafadha . Jalqaba, maatiin kee wantoota gabatee armaan gadii keessatti eeraman akka qabdan beekuun barbaada , amma ykn waqtii darbe (ji’a jahaan darban). Sana booda maatii kee keessaa eenyu akka ittiin hojjatu ykn fayyadamu baruun barbaada, eenyutu qaba, fi isaanirratti Murtii kan Kennuu eennu akka ta’e

Gabatee 9.1

| **Lakk.** | **Gosa/akaakuu** | **Ni argamaa** (yoo lakki ta’e, gara gaaffii itti aanuutti darbi) | **Abbummaa**  **Koodii jalaa** | **Too’achuu (bituuf/fayyadamuuf/gurguruuf kan murteessu)**  **Koodii jalaa** | **Itti fayyadama galiirratti kan murteessu**  **Koodii jalaa** |
| --- | --- | --- | --- | --- | --- |
|  |  | 1. Eeyyee 0. lakki | 1. dubartii 2. Dhiira  3. lameenu  4. kanbiraa | 1. Dubartii 2. Dhiira  3. lameenu | 1. Dubartii 2. Dhiira  3. lameenu  4. hinsakkatta’amne |
| 9.1.1. | Lafa qonnaa | GLANDA | GLANDO | GLANDC | GLANDD |
| 9.1.2. | itti fayyadama lafa qonnaa |  |  | GLANDUSEC |  |
| 9.1.3. | lafa waan naannoo manatti oomishamuuf oolu | GGARDA | GGARDO | GGARDC | GGARDD |
| 9.1.4. | Lafa qonnaaf hin oollee(kan daldalaaf jireenyaaf oolu) | GNALANDA | GNALANDO | GNALANDC | GNALANDD |
| 9.1.5. | Midhaan nyaataa | GCEREALA | GCEREALO | GCEREALC | GCEREALD |
| 9.1.6. | Mukaa fuduraa | GBANANA | GBANANAO | GBANANAC | GBANANAD |
| 9.1.7. | Hiddaa/jirma | GROOTSA | GROOTSO | GROOTSC | GROOTSD |
| 9.1.8. | Midhaan gurgurtaaf oolan(buna,caatii KKF) | GCASHCA | GCASHCO | GCASHCC | GCASHCD |
| 9.1.9. | Muduraalee | GVEGA | GVEGO | GVEGC | GVEGD |
| 9.1.10. | Midhaan dhedhi (Atara, baaqelaa kkf) | GPULSA | GPULSO | GPULSC | GPULSD |
| 9.1.11 | Midhaan zeeyitaa | GOILSA | GOILSO | GOILSC | GOILSD |
| 9.1.12 | Mi’eesituu/kan baalli isaan fayyadu | GSPICA | GSPICO | GSPICC | GSPICD |
| 9.1.13 | Loon | GCATTLEA | GCATTLEO | GCATTLEC | GCATTLED |
| 9.1.14 | Farad,harree gangee | GMULEA | GMULEO | GMULEC | GMULED |
| 9.1.15 | Hoolaa/re’ee | GRUMINA | GRUMINO | GRUMINC | GRUMIND |
| 9.1.16 | Horsiisa lukkuu | GPOULTRYA | GPOULTRYO | GPOULTRYC | GPOULTRYD |
| 9.1.17 | Kannisa horsiisuu | GBEEHA | GBEEHO | GBEEHC | GBEEHD |
| 9.1.18 | Manaafiijaarsa biroo | GHOUSEA | GHOUSEO | GHOUSEC | GHOUSED |
| 9.1.19 | Meeshaalee qonnaa ammayyaa kan hin taane | GFEQUIPNMA | GFEQUIPNMO | GFEQUIPNMC | GFEQUIPNMD |
| 9.1.20 | Meeshaalee qonnaa kan ammayyaa | GFEQUIPMA | GFEQUIPMO | GFEQUIPMC | GFEQUIPMD |
| 9.1.21 | Meeshaalee qonnaan alaa kan galii fidan | GBUSEQUIPA | GBUSEQUIPO | GBUSEQUIPC | GBUSEQUIPD |

*koodii 1, itti gaafatamaa maatii 2.abbaa 3. Niitii duraa 4. Haadha 5. niitii lammaffaa 6. ga’essa dhala kan biraa 7. ga’essa dhiraa kan biraa 8. hin ilaallatu –[ kan ilaalatu hundaa filadhu]

## Kutaa 2: Itti Dhiheenna, Abbummaa, Fi Too’annoo Meeshaalee Yeroo Dheeraaf Turan

Amma waa’ee meeshaalee mana keessaa sigaafadha. Maaloo mee yoo meeshaleen kunneen amma mana keessan keessatti yoo jiraatan ta’e natti himuun, eenyu akka itti fayyadamu, kan eenyu akka ta’anii fi fayyadama isaanii eenyu akka too’atu.

| **Lakk. Gaafii** |  | **Ni argam** | **Baay’inna argamu**  **(laakkofsa)** | **Baay’inna kan hojjatuu**  **(laakkofsa)** | **Abbummaa(kan hin hojjanne)**  **Koodii kanaan gadii ilaali** | **Abbummaa(kan hojjatu)**  **Koodii kanaan gadii ilaali** | **Itti dhiheenna(kan hojjatu)**  **Koodii kanaan gadii ilaali** | **Itti fayyadama kan too’atu**  **(kan hojjatu)**  **Koodii kanaan gadii ilaali** |
| --- | --- | --- | --- | --- | --- | --- | --- | --- |
|  | **Meeshaalee** | (1=eeyyee ,0=lakki) lakki yoo ta’e, gabatee gara mirgaatti darbi |  |  | 1. dubartii  2. Dhiira  3. lameenu  4. kanbiraa |  |  | 1. dubartii  2. Dhiira  3. lameenu  4. kanbiraa |
| 2.1.1 | Raadiyoo | GRADIOA | GRADIOQ | GRADIOQF | GRADIONF | GRADIOO | GRADIOS | GRADIOC |
| 2.1.2 | Televiziyoona | GTVA | GTVQ | GTVQF | GTVNF | GTVO | GTVS | GTVC |
| 2.1.3 | Bilbila manaa/Bilbila kansarara dhaabbataa | GTELEA | GTELEQ | GTELEQF | GTELENF | GTELEO | GTELES | GTELEC |
| 2.1.4 | Bilbila mobaayilii | GMOBILA | GMOBILQ | GMOBILQF | GMOBILNF | GMOBILO | GMOBILS | GMOBILC |
| 2.1.5 | Biskileetii | GBICA | GBICQ | GBICQF | GBICNF | GBICO | GBICS | GBICC |
| 2.1.6 | Motor saayikilii | GMOTOA | GMOTOQ | GMOTOQF | GMOTONF | GMOTOO | GMOTOAS | GMOTOC |
| 2.1.7. | Baajaajii | GBAJAJAA | GBAJAJQ | GBAJAJQF | GBAJAJNF | GBAJAJO | GBAJAJS | GBAJAJC |
| 2.1.8 | Gaarii | GCARTA | GCARTQ | GCARTQF | GCARTNF | GCARTO | GCARTS | GCARTC |

*koodii 1. itti gaafatamaa maatii 2.abbaa 3. Niitii duraa 4. Haadha 5. niitii lammaffaa 6. ga’essa dhala kan biraa 7. ga’essa dhiraa kan biraa 8. hin beekamu –[ kan ilaalatu hundaa filadhu]

##

## Kutaa 3: Koornayaa Fi Yeroo Ramaduu/Qooduu

Kaleessa yeroo kee akkamitti akka itti fayyadamtee sigaafachuuf barbaada. Kaleessa yeroo kee akkamitti dabarsite, yeroo hirribaa kaaterraa hanga raftetti?

|  | | ***Filannoo tokko filadhu:*** | | |
| --- | --- | --- | --- | --- |
|  |  | **1.   hojii qonnaa midhaan** | | |
|  |  | **2.Hojii hormaata horii** | | |
|  |  | **3.   Hojii qonnaan alaa kan koo** | | |
|  |  | **4.Hojii qonnaan kan kafaltiin** | | |
|  |  | **5.Gatii Imala(dhaqaaf gala)** | | |
|  |  | **6.kafaltii adda addaatiif fayyaa dabalatee** | | |
|  |  | **7.Baruumsaaf ykn leenjiif** | | |
|  | | **8. maatii/mana keessatti ijoollee/hangafoota/dhukkubsattoota wajjiin**  **9. yeroo dhuunfaa(boqqonna, yeroo amantaa, yeroo hawaasummaa, yeroo nyaata)** | | |
| **Yeroo /sa’aa** | | **Dubartii qorannoo kanaaf barbaadamtuu** |  | |
| 00:00 –halkan walakkaa | | GTIMEF00 |  | |
| 7:00 AM | | GTIMEF01 |  | |
| 8:00 AM | | GTIMEF02 |  | |
| 9:00 AM | | GTIMEF03 |  | |
| 10:00 AM | | GTIMEF04 |  | |
| 11:00 AM | | GTIMEF05 |  | |
| 12:00 AM | | GTIMEF06 |  | |
| 1:00 AM ganama | | GTIMEF07 |  | |
| 2:00 AM | | GTIMEF08 |  | |
| 3:00 AM | | GTIMEF09 |  | |
| 4:00 AM | | GTIMEF10 |  | |
| 5:00 AM | | GTIMEF11 |  | |
| 6:00 **Guyyaa/saafaya** | | GTIMEF12 |  | |
| 7:00 PM | | GTIMEF13 |  | |
| 8:00 PM | | GTIMEF14 |  | |
| 9:00 PM | | GTIMEF15 |  | |
| 10:00 PM | | GTIMEF16 |  | |
| 11:00 PM | | GTIMEF17 |  | |
| 12:00 PM **galgala** | | GTIMEF18 |  | |
| 1:00 PM | | GTIMEF19 |  | |
| 2:00 PM | | GTIMEF20 |  | |
| 3:00 PM | | GTIMEF21 |  | |
| 4:00 PM | | GTIMEF22 |  | |
| 5:00 PM | | GTIMEF23 |  | |
|  | |  |  | |

# MuJulii 10: Hirmaannaa Hawaasummaa fiitti Dhiheenna Odeeffannoo

kutaa 1: Hirmaannaa hawaasummaafi itti gamadinsa isaa

Ati ykn miseensonni maatii keetii amma gareewwan hawaasummaa kan asii gaditti eeraman keessatti hirmaataa jirtaa/jiru

**Gabatee 10.1**

|  | **Garee** | Dubartii qo’annoo kanaaf Ijoo taate (1=eeyyee 0=lakki )98=hinbeku | Miseensota biro (1=eeyyee 0=lakki) 98=hinbeku |
| --- | --- | --- | --- |
| 10.1.1 | **Waldaa qusanaf liqi badiyaa** |  |  |
| 10.1.2 | **Garee waligargarssa dubartota** |  |  |
| 10.1.3 | Gareewwan amantaa | GFARMC | GFARMO |
| 10.1.4 | Gareewwan dargaggootaa | GWOMC | GWOMO |
| 10.1.5 | Waldaa qonan bultota/omishitota | GRELIGC | GRELIGO |
| 10.1.6 | Koree gandaa ( bulchiinsaa , fayyaa, kanbiroo) | GYOUTHC | GYOUTHO |
| 10.1.7 | Gareewwan maallaqaan walqabatan (iddirii, iqqubii , garee maallaqaa xixiqqoo kkf) | GHEALTHC | GHEALTHO |
| 10.1.8 | Garee biraa (ibsi___________) GOTHSPE | GKEBELEC | GKEBELEO |

## Kutaa 4: Sagantaaf Saaxilamuu fi Fudhatamummaa Isaa –MHCN and ENGINE Activities

Gabatee 10.4

| 1. At (miseensi maatii ke)ji’oota ja’an darban keessati hojiile armaan gadiitti hirmaate turte   0=lakki 1=eyyee 98=hin beeku  Yoo eyyee (hin bku ta’e) gara ‘C’ tti darbi | | 1. yoo lakki ta’e maaliif kan deeb ta’an hunda filadhu 0=hojichi hin ture   1=yeroon dhabe  2,sababa fageenya  3,Bayyee mi’ayyaa waan ta’eef  4,fedhii dhabuu  5,fayyida waan hin qabneef  6,kan biraa  98=hin beeku | 1. C. Maada odefanno (debii kan ta’an hunda filadhu)   1=hojjattoota extension qonnaa  2= hoogganaa qonnaa  3= ogeessa fayyaa loonii  4= marri hawaasaa  5=garee hawassuma /amnata  6=dhabata mitti motumma  7=Radiyoo  8=televiziyoona  9=barulle/gazexa  10=bilibila mobayela  11=barefamafi fakille adda adda  12= hiriyaa/firaa  13=kan biro  98=hinbekuu | D. ji’oota 6n darban keessatti yeroo ammamiif atti (miseensi )maatiike hojii irrati hirmaata | | 1. sababa hojiilee kanaatiin atti (miseensa maatii) ke keessa jijiiram ammala kan fide jiraa?   0=lakki  1,eyyeen  98=hin beeku | F. yoolakki ta’emaaliif (kan deebi Ta’an hunda filadhu  1,yeroo dhabuu  2,bayyee mi’aa ta’u  3,fedhii dhabuu  4,fayyida hin qabu  5,kan biro   1. 98=hin beeku |
| --- | --- | --- | --- | --- | --- | --- | --- |
|  |  |  |  | Lakkoofsa  (98 yoo lakki ta’e) | Koodii :  1=torban  2=ji’a  3=ji’a 6n darban |  |  |
| Waa’ee sagantaa/proojaktii enjiin/ENGINE dhageessee beektaa?  Yoo eyyee ta’ e gara C.tti darbi |  |  |  |  |  |  |  |
| Eeyyee yoo ta’e, ati kallattiidhaan fayyadamoo sagantaa ENGINE dha? |  |  |  |  |  |  |  |
| Yoo eeyyee ta’e ati namoota baay’inaan hubamanii fi kan sagantaa ENGINE gargaaramtuu dhaa ? |  |  |  |  |  |  |  |
| Odeefanno (leenjii) wa’ee  Fayyaa/sirna nyaataa fudhattee? |  |  |  |  |  |  |  |
| Odeefanno (leenjii)  Mata duree armaan gadiirratti fudhattee? |  |  |  |  |  |  |  |
| *Daa’imni kee akka fayyaa gaarii qabaatuuf akkamitti nhaachifta?* |  |  |  |  |  |  |  |
| *Harma hoosiftaa* |  |  |  |  |  |  |  |
| *Mallatttoo dhukkubaa kan yaala barbaachisu daa’ima keetii addaan baafachuu?* |  |  |  |  |  |  |  |
| *Yeroo ulfaa ofeeguu/ofkunuunsuu* |  |  |  |  |  |  |  |
| *daa’ima garaa kaasu yaaluu* |  |  |  |  |  |  |  |
| *Nyaata gara garaa* |  |  |  |  |  |  |  |
| *Nyaata madaalawaa ta’e akkamitti akka qophaa’u?* |  |  |  |  |  |  |  |
| *Bishaan akkamitti akka qulqullaa’u* |  |  |  |  |  |  |  |
| *Harka dhiqachuu* |  |  |  |  |  |  |  |
| Mana fincaanitti fayyadamuu |  |  |  |  |  |  |  |
| *Ittisa HIV/AIDS* |  |  |  |  |  |  |  |
| *Hojiiwwan asii gadii kamiiyyuu irratti hirmaattee?* |  |  |  |  |  |  |  |
| *Gorsa waa’ee nyaataa fi gargaarsa argachuuf gara mana yaalaa deemtee* |  |  |  |  |  |  |  |
| *Waa’ee sagantaa raadiyoo ’’shiiqanaat’’ dhaggeeffattee?* |  |  |  |  |  |  |  |
| *Marii hawaasummaa irratti hirmaachuu* |  |  |  |  |  |  |  |
| *Garee deeggarsaa dubartootaa irratti waa’ee sirna nyaataa/ qulqullina dhuunfaa barachuuf hirmaachuu* |  |  |  |  |  |  |  |
| *Agarsiisa oomisha boroo irratti hirmaachuu* |  |  |  |  |  |  |  |
| *Garee qusannaa dubartootaa irratti hirmaachuu* |  |  |  |  |  |  |  |
| Wantoota kanaan gaditti eeraman keessaa kamuuyyuu haata’u fudhattee? |  |  |  |  |  | Itti fayyadamaa jirtaa? |  |
| Meeshaa bishaan qulqulleessu? |  |  |  |  |  |  |  |
| Qoricha hanqina dhiigaa */Iron-folate supplements?* |  |  |  |  |  |  |  |
| *Qoricha farra raammoo garaa?* |  |  |  |  |  |  |  |
| *Vitamin A?* |  |  |  |  |  |  |  |
| *Ashaboo/soogidda ayoodinii qabu?* |  |  |  |  |  |  |  |
| *Nyaataa Kenaa (dakuu, Qincee,Sukaraa,zayita,kkf)?* |  |  |  |  |  |  |  |
| kitaaba waa’ee maatii kan ninisteera eegumsa fayyaatiin qophaa’ee fi dhaamsa barbaachisoo 64 qabu*?* |  |  |  |  |  |  |  |
| Daa’ima ji’a 6 oliitiif: daa’imni kee kan armaan gadii fudhatee: |  |  |  |  |  |  |  |
| *Qoricha raammoo garaa?* |  |  |  |  |  |  |  |
| *Vitamin A?* |  |  |  |  |  |  |  |
|  |  |  |  |  |  |  |  |

##

## Kutaa 5: Tajajila Fi Ittigamadinsa

| Gaafii | Debii | Filanno | Dherina dawanna darbe |
| --- | --- | --- | --- |
| Wagaa darbe kessa dhabile fayya daawattee turtee? | 1=eye  0=lakki | Yoo eye ta’e,  1=bufata fayya 2=kilikaa dhunfa  3=kanbiroo | daqiqa____ |
| Gara mana yalaa demuuf hangam sitti fudhata? |  |  | daqiqa____ |
| Yeroo gara mana yaalaa deemte hundatti sigammachiisee turee? | 1=eye  0=lakki--- yoo lakki jette, maaliif? | Yoo lakki ta’e  1=yeroo ga’a hinqabuu  2=odefanno gaha hinqabu  3= hanqina qarshii  4= amala badaa hojjattoota fayyaatiin  5= hin beeku |  |
| Waggaa darbe keessa ogeessota fayyaatiin manatti deeggarsa argattee beektaa? | 1=eye  0=lakki | Yoo eye ta’e,  1=hojjettu extension fayya  2= tokko shane 3=ogessa fayya  4=ogessa qonna 5=kan biro ibsi | daqiqa____ |
| Ji’otaa sadan darban keessa yeroo meeqa daawwannaa manatti aragatte? | 1= torabantti al tokko  2=ji’atti si’a 1-2  3=ji’a lamatti si’a tokko  4=ji’a sada’itti si’a tokko  5=ji’a saditti yeroo duwwaa |  |  |
| Dawanna manatti siif godhame gammaddee jirtaa | 1=eye  0=lakki | Yoo lakki ta’e  1=yeroo ga’a hinqabuu  2=odefanno gaha hinqabu  3= hanqina qarshii  4= amala badaa hojjattoota fayyaatiin  5= hin beeku |  |

# Mujulii 12 – Galii fi Baasii

## Kutaa 1: Madda Galii Matii Kan Biroo

Amma, maaloo maddoota biroo Kan maatiin kee adoolessa 2012(2004 E.C.)-waxabajjii 2013(2005 E.C.)Galii irraa argatan natti himi. Maaloo galii miseensoota maatii keetii hundaa itti dabalii natti himi*.*

Table 12.1

|  | **Gosa galii argatan** | **Galii hojii kanarraa argatame? (1=eeyyee , 0=lakki)** (lakki yoo jette, gara gabatee mirgaatti darbi) | **Galii kana argachuuf baasii bahe?** (1=eeyyee 0=lakki) | **Yoo eeyyee ta’e, baasii bahe eere/himi** | **Yoo eeyyee ta’e , baasii dimshaashaa (Birr)** | **Bifa galii argatte 1=*maallaqaan 2= kan maallaqaan hintaane*** | **Baay’ina (e.g. lakk. jooniyyaa, xaasaa)** | **safartuu (yoo maallaqaan ta’uu baate)** | **Gatii tokkoo (Birr)** | **Gatii dimshaashaa (Birr)** | **Galii dhumaa (Birr)** |
| --- | --- | --- | --- | --- | --- | --- | --- | --- | --- | --- | --- |
| 12.1.1 | Humna namaa hojii qonna birootiif oole (midhaan ykn loon) | MAGI | MAGC | MAGT | MAGCV | MAGF | MAGA | MAGU | MAGUV | MAGTV | MAGTVC |
| 12.1.2 | Nama hojii qonnaan alatti qaxarame – akka tasaa, yeroodhaaf, ykn sirnaan/mindaa (gargaaraa/tuu manaa, ijaarsa, kkf dabaltee.) | MNOAGI | MNOAGC | MNOAGT | MNOAGCV | MNOAGF | MNOAGA | MNOAGU | MNOAGUV | MNOAGTV | MNOAGTVC |
| 12.1.3 | Soorama/xuurataa | MPENSI | MPENSC | MPENST | MPENSCV | MPENSF | MPENSA | MPENSU | MPENSUV | MPENSTV | MPENSTVC |
| 12.1.4 | Gargaarsa mootummaarraa ,mitimootummaarraa /mootummoota gamtoomanii(nyaata, sanyii, ykn gargaarsa loonii) | MASSTI | MASSTC | MASSTT | MASSTCV | MASSTF | MASSTA | MASSTU | MASSTUV | MASSTTV | MASSTTVC |
| 12.1.5 | Galii nama biyya alaa irraa | MREMITI | MREMITC | MREMITT | MREMITCV | MREMITF | MREMITA | MREMITU | MREMITUV | MREMITTV | MREMITTVC |
| 12.1.6 | Gargaarsa fira dhioo/hiriyyaa irraa | MASRELI | MASRELC | MASRELT | MASRELCV | MASRELF | MASRELA | MASRELU | MASRELUV | MASRELTV | MASRELTVC |
| 12.1.7 | Kennaa | MGIFTI | MGIFTC | MGIFTT | MGIFTCV | MGIFTF | MGIFTA | MGIFTU | MGIFTUV | MGIFTTV | MGIFTTVC |
| 12.1.8 | Kan kireeffame (lafa, horii, meeshaalee) | MRENTI | MRENTC | MRENTT | MRENTCV | MRENTF | MRENTA | MRENTU | MRENTUV | MRENTTV | MRENTTVC |
| 12.1.9 | Muka ibiddaaf ta’uu gurguruun | MWOODI | MWOODC | MWOODT | MWOODCV | MWOODF | MWOODA | MWOODU | MWOODUV | MWOODTV | MWOODTVC |
| 12.1.10 | Xarbii/muka gurguruun | MPOLEI | MPOLEC | MPOLET | MPOLECV | MPOLEF | MPOLEA | MPOLEU | MPOLEUV | MPOLETV | MPOLETVC |
| 12.1.11 | Kasala gurguruun | MCHARCI | MCHARCC | MCHARCT | MCHARCCV | MCHARCF | MCHARCA | MCHARCU | MCHARCUV | MCHARCTV | MCHARCTVC |
| 12.1.12 | Hojii harkaan hojjataman gurguruun | MCRAFTI | MCRAFTC | MCRAFTT | MCRAFTCV | MCRAFTF | MCRAFTA | MCRAFTU | MCRAFTUV | MCRAFTTV | MCRAFTTVC |
| 12.1.13 | Nyaata/dhugaatii qophaa’aa gurguruu/ mana nyaataatti gurguruun | MFOODI | MFOODC | MFOODT | MFOODCV | MFOODF | MFOODA | MFOODU | MFOODUV | MFOODTV | MFOODTVC |
| 12.1.14 | Dhugaatii gurguruu-kan aadaa dabalatee | MBREWI | MBREWC | MBREWT | MBREWCV | MBREWF | MBREWA | MBREWU | MBREWUV | MBREWTV | MBREWTVC |
| 12.1.15 | Shaqaxa gurguruu | MSHOPI | MSHOPC | MSHOPT | MSHOPCV | MSHOPF | MSHOPA | MSHOPU | MSHOPUV | MSHOPTV | MSHOPTVC |
| 12.1.16 | Geejjiba | MBODAI | MBODAC | MBODAT | MBODACV | MBODAF | MBODAA | MBODAU | MBODAUV | MBODATV | MBODATVC |
| 12.1.17 | Gurgurtaa dirqoosha/haftee midhaanii /kompoostii /marga /mannoo | MHAYI | MHAYC | MHAYT | MHAYCV | MHAYF | MHAYA | MHAYU | MHAYUV | MHAYTV | MHAYTVC |
| 12.1.18 | Kobota gurguruu | MMANI | MMANC | MMANT | MMANCV | MMANF | MMANA | MMANU | MMANUV | MMANTV | MMANTVC |
| 12.1.19 | Foon gurguruun/ salegame meat | MGAMEI | MGAMEC | MGAMET | MGAMECV | MGAMEF | MGAMEA | MGAMEU | MGAMEUV | MGAMETV | MGAMETVC |
| 12.1.20 | lawzii/fuduraa gurguruun | MWILDI | MWILDC | MWILDT | MWILDCV | MWILDF | MWILDA | MWILDU | MWILDUV | MWILDTV | MWILDTVC |
| 12.1.21 | Albuuda baasuun (soogidda, warqee, cirracha, suphee, kkf) | MMINEI | MMINEC | MMINET | MMINECV | MMINEF | MMINEA | MMINEU | MMINEUV | MMINETV | MMINETVC |
| 12.1.22 | Dhakaa bocuun/qotuun baasuu | MSTONEI | MSTONEC | MSTONET | MSTONECV | MSTONEF | MSTONEA | MSTONEU | MSTONEUV | MSTONETV | MSTONETVC |
| 12.1.23 | Xuubii hojjachuun | MBRICKI | MBRICKC | MBRICKT | MBRICKCV | MBRICKF | MBRICKA | MBRICKU | MBRICKUV | MBRICKTV | MBRICKTVC |
| 12.1.24 | Maashinii daakuu | MGMILLI | MGMILLC | MGMILLT | MGMILLCV | MGMILLF | MGMILLA | MGMILLU | MGMILLUV | MGMILLTV | MGMILLTVC |
| 12.1.25 | Daldala xixiqqoo biro | MPETTYI | MPETTYC | MPETTYT | MPETTYCV | MPETTYF | MPETTYA | MPETTYU | MPETTYUV | MPETTYTV | MPETTYTVC |
| 12.1.26 | kan biroor (ibsi_____) MOTH1SPE | MOTH1I | MOTH1C | MOTH1T | MOTH1CV | MOTH1F | MOTH1A | MOTH1U | MOTH1UV | MOTH1TV | MOTH1TVC |
| 12.1.27 | kanbiroo (ibsi_____) MOTH2SPE | MOTH2I | MOTH2C | MOTH2T | MOTH2CV | MOTH2F | MOTH2A | MOTH2U | MOTH2UV | MOTH2TV | MOTH2TVC |

**Kutaa 3: Baasii kan biro**Gabatee 12.3

|  | Ji’a darbe dhumaa keessatti, maatiin kee bittaa ykn kafaltii kamiyyuu haata’u raawwatee turee [meeshaalee]? eeyyee…1  lakki …2 ►gara meeshaa itti aanutti 98= hinbekuu ►gara meeshaa itti aanutti | | Waliigalatti maatiin kee qarshii meeqa kafalan?  [BIRR] |
| --- | --- | --- | --- |
| 1 | Kibriita/kibrit | HEMATCH | HEMATCHB |
| 2 | Baatirii/tirriika | HEBATT | HEBATTB |
| 3 | Shaamaa/duungoo (xuwaafii), ixaana | HECANDL | HECANDLB |
| 4 | Saamunaa laawundarii/OMO/endod/ baala **besana** | HESOAPL | HESOAPLB |
| 5 | Saamunaa harkaa | HESOAPH | HESOAPHB |
| 6 | Meeshaale biro qulqullina dhuunfaatiif ittiin fayyadamnu (sandala, matent/qayyaa,) | HEPERSCR | HEPERSCRB |
| 7 | Kasala/cilee | HECHARC | HECHARCB |
| 8 | Muka qoraanii | HEFIREWD | HEFIREWDB |
| 9 | Keerosinii/gaazii adii/Kerosene | HEKEROS | HEKEROSB |
| 10 | Sigaaraa , tamboo , oofee/suret, gaya | HETOBAC | HETOBACB |
| 11 | Geejjiba | HETRANSP | HETRANSPB |

Gabatee12.3b

|  | Ji’oota 12n darban dhumaa keessatti, maatiin kee bittaa ykn kafaltii kamiyyuu haata’u raawwatee turee [meeshaalee]?  eeyyee…1  lakki …2 ►gara meeshaa itti aanutti 98= hinbekuu ►gara meeshaa itti aanutti | | Waliigalatti maatiin kee qarshii kee meeqa kafalan?  [BIRR] |
| --- | --- | --- | --- |
| 1 | uffata/kophee/uffata dhiiraa | HECLOTHM | HECLOTHMB |
| 2 | uffata/kophee/uffata dubartootaaf | HECLOTHW | HECLOTHWB |
| 3 | uffata/kophee/uffata ijoollee dhiiraatiif | HECLOTHB | HECLOTHBB |
| 4 | uffata/kophee/uffata ijoollee dubaraatiif | HECLOTHG | HECLOTHGB |
| 5 | Meeshaalee kushinaa (okkotee ittiin bilcheessan, kkf.) | HEKITCH | HEKITCHB |
| 6 | Linens (ansoolaai, fooxaa ,awusaa/baanaa) | HELINEN | HELINENB |
| 7 | Meeshaalee manaa/Furniture | HEFURN | HEFURNB |
| 8 | Ampuulii/Xomboora/chibboo-Lamp/torch | HELAMP | HELAMPB |
| 9 | Baasii ayyaanaaf oolu/Ceremonial expenses | HECEREM | HECEREMB |
| 10 | Gumaacha Iddir tiif | HEIDDIR | HEIDDIRB |
| 11 | Kennaa bataskaanaaf/masgiidaaf | HECHURCH | HECHURCHB |
| 12 | Kafaltii mana baruumsaatiif | HESCHO | HESCHOB |
| 13 | Gatii/Kafaltii eegumsa fayyaatiif | HEHELYR | HEHELYRB |

## Kutaa 4: Baasiiwwan Biroo

Gabatee 12.4: Bassi nyaata

| Torban darbe keessatti (guyyoota 7n) ati ykn maatii kee keessa namni soorata asii gaditti eeraman nyaate jiraa? *1=eeyyee 0=lakki 98=hinbekuu (gara gaffi itti anutti darbii)* | | | Waliigalatti torban darbe maatiin kee hagam sooratan? Hinjiruu=0 | | Hagamtu bittaan argame/dhufe?Hinjiruu=0 | | Hagam baafte? | Hagamtu oomisha keessanirraa argame/dhufe?Hinjiruu=0 | | Hagamtu mesha birottin jijirudhan dhufee? Hinjiruu=0 | | Hangamtu kennaa ykn madda birootirraa argame/ dhufe? | |
| --- | --- | --- | --- | --- | --- | --- | --- | --- | --- | --- | --- | --- | --- |
|  |  |  | Baay’ina | safartuu | Baay’ina | safartuu | Birrii | Baay’ina | safartuu | Baay’ina | safartuu | Baay’ina | safartuu |
|  | | **Midhaan [kan biilaa]** | | | | | | | | |  | | |
| Xaafii |  | |  |  |  |  |  |  |  |  |  |  |  |
| Qamadii |  | |  |  |  |  |  |  |  |  |  |  |  |
| Garbuu |  | |  |  |  |  |  |  |  |  |  |  |  |
| Boqqoolloo |  | |  |  |  |  |  |  |  |  |  |  |  |
| Mishingaa |  | |  |  |  |  |  |  |  |  |  |  |  |
| Daagijjaa/dagusa |  | |  |  |  |  |  |  |  |  |  |  |  |
|  | | **Sanyii baaqelaaf shumburaa -Pulses** | | | | | | | | | | | |
| Baaqelaa |  | |  |  |  |  |  |  |  |  | |  |  |
| Shumburaa |  | |  |  |  |  |  |  |  |  | |  |  |
| Atara |  | |  |  |  |  |  |  |  |  | |  |  |
| Missira |  | |  |  |  |  |  |  |  |  | |  |  |
| Boloqqee |  | |  |  |  |  |  |  |  |  | |  |  |
|  | | **Sanyii zayitaa /Oil Seeds** | | | | | | | | | | | |
| Nuugii |  | |  |  |  |  |  |  |  |  | |  |  |
| Talbaa |  | |  |  |  |  |  |  |  |  | |  |  |
|  | | **Muduraa fi fuduraa [Vegetables & Fruit]** | | | | | | | | | | | |
| Shunkurtii |  | |  |  |  |  |  |  |  |  | |  |  |
| Muuzii |  | |  |  |  |  |  |  |  |  | |  |  |
|  | | **Kan Hiddaa fi Jirmi nyaatamu [Tubers & Stems]** | | | | | | | | | | | |
| Dinnicha |  | |  |  |  |  |  |  |  |  | |  |  |
| Qooccoo/bullaa |  | |  |  |  |  |  |  |  |  | |  |  |
|  | | **Kan biroo [Others]** | | | | | | | | | | | |
| Foon |  | |  |  |  |  |  |  |  |  | |  |  |
| Aannan |  | |  |  |  |  |  |  |  |  | |  |  |
| Ittoosa’aa/ayibii |  | |  |  |  |  |  |  |  |  | |  |  |
| Hanqaaquu |  | |  |  |  |  |  |  |  |  | |  |  |
| Sukkaara |  | |  |  |  |  |  |  |  |  | |  |  |
| Soogidda/ashaboo |  | |  |  |  |  |  |  |  |  | |  |  |
|  | | **Kan sammuu namaa kakaasuu/si’eessituu [Stimulants]** | | | | | | | | | | | |
| Buna |  | |  |  |  |  |  |  |  |  | |  |  |
| Caatii/jimaa |  | |  |  |  |  |  |  |  |  | |  |  |

koodii: 1=Giraama, 2=seentimeetira, 3=Cm^3^, 4=lakkoofsa, 5=meetira, 6=mirree/xindii/pair, 7= saanduqa/saaxinii, 8=zurba 11=marama, 12=paakkoo/pack, 20=kg, 31=kubbaayyaa/cup, 32=leetira, 33=iskuweer meetira/ meter squared

# Mujulii 13: Qorannoo Laabraatorii fi Safara antirooppoomeetriikii

# [Anthropometric Measurements]

## Kutaa 1: Qorannoo Busaa – Dubartii qo’annaaf barbaadamtu/qo’atamtu

|  | **Gaafii** | **Deebii** | **Variable** |
| --- | --- | --- | --- |
| 13.1.1 | Bu’aa qorannoo dubartii qo’atamtu | C band 1=eeyye 0=lakki  Pan band 1=eeyyee 0=lakki  Pf band 1=eeyyee 0=lakki  0= negative  1=positive  2=qorannoon hinhojjatamne (sababni __________________) LCRAPCGR | LCCBCG  LCPANCG  LCPFCG  LCRAPCG |
| 13.1.2 | Yoo bu’aan qorannoo poozatiivii ta’e, dubartiin guyyoota 7n darban keessatti yaala dhukkuba busaa fudhattee turtee? | 1=eeyyee 0=lakki | LCTRTCG |
| 13.1.3 | Eeyye yoo jette, dubartiin yaala/qoricha kennameef xumurtee? | 1=eeyyee  0=lakki | LCMALATRTCG |
| Yoo bu’aan qorannoo dubartii qo’annaaf barbaadamtu poozatiivii ta’e *fi* yaala/qoricha kennameef hunda kaxumurte yoo ta’e*, gara dhaaba fayyaa dhihoo argamutti ergi. Yoosan ta’uu baate, wal’aansa/yaala sadarkaa duraaf kennamuu hundaa kenniif.* | | | |
| 13.1.4 | Qoricha/yaala maaltuu dubartiif kenname? | 0=homaa  1=yaala sadarkaa 1ffaa  2=gara mana yaalaatti ergamte | LCWHATRTCG |

## Kutaa 2: Antirooppoomeetriikii Dubartii Qo’annaaf barbaadamtu [Index Woman Anthropometry]

Gabatee 13.2

|  | **Gaafii** | **Deebii** | **Var. name** |
| --- | --- | --- | --- |
| 13.2.1 | Qubeewwan duraa maqaa dubartii qo’annaaf barbaadamtu |  | AMINIT |
| 13.2.2 | Guyyaa dhalootaa |  | AMYOB |
| 13.2.3 | Umrii waggaadhaan |  | AMYRS |
| 13.2.4 | Ulfa | 1=eeyyee  0=lakki | AMPREG |
| 13.2.5 | Ulfaa’ina 1 | . kg | AMWT1 |
| 13.2.6 | Ulfaa’ina 2 | . kg | AMWT2 |
| 13.2.7 | Ulfaa’ina 3 | . kg | AMWT3 |
| 13.2.8 | dheerina 1 | . cm | AMHT1 |
| 13.2.9 | Dheerina 2 | . cm | AMHT2 |
| 13.2.10 | Dheerina 3 | . cm | AMHT3 |
| 13.2.11 | MUAC 1 | . cm | AMMUAC1 |
| 13.2.12 | MUAC 2 | . cm | AMMUAC2 |
| 13.2.13 | MUAC 3 | . cm | AMMUAC3 |
| 13.2.14 | BP Systilic | .mm Hg |  |
|  | BP diastolic | .mm Hg |  |
| 13.2.15 | Olerguun /Referral / kennamee ? | 1=eeyyee  0=lakki | AMREF |

# Kutaa 5: safara hemocue

| Lakk | Gaaffii | Dubartti qo’atamttu | Da’ima qo’atamittu |
| --- | --- | --- | --- |
| 13.5.1 | Qubeewwan duraa maqaa | . | . |
| 13.5.2 | Hemocue hojjetame | 1=eye  0=lakki | 1=eye  0=lakki |
| 13.5.3 | Bu’a qoranna hemacue (g/dL) (yoo qorannon hingageffamne gaffi itti anutti darbii) | . | . |
| 13.5.4 | yoo qorannon hingageffamne mallif? |  |  |
| 13.5.5 | Oliergameraa/refered? | 1=eye  0=lakki | 1=eye  0=lakki |
