# Supplementary material for: Nutrition-specific and nutrition-sensitive factors associated with mid-upper arm circumference as a measure of nutritional status in pregnant Ethiopian women: Implications for programming in the first 1000 days
Source: PLoS One. 2019 Mar 26;14(3):e0214358. doi: 10.1371/journal.pone.0214358 (PMC6435172; doi:10.1371/journal.pone.0214358)
Supplement: S3 File — Questionnaires in Afan-Oromo, the language of questionnaire administration. (ZIP) [file pone.0214358.s003.zip › HH HEAD INTERVIEW-AFAN OROMO -FINAL.docx]

Afgaafii Qorannoo maatii Kan ENGINE - USAIDtiin gaggeeffamuu

**Gaaffiifi Deebii Itti Gaafatamaa Maatii**

**Qabeeyyii Fuula**

[Mujulii 1: Odeeffannoo Fi Haala Maatii 3](#_Toc380080256)

[Kutaa 1: Odeeffannoo Gaafif Deebii 3](#_Toc380080257)

[Kutaa 2: Waa’ee Beekumsa Itti Gaafatamaa Maatii 4](#_Toc380080258)

[Kutaa 3: Qulqullinaa fi Bishaan [water & sanitation] 4](#_Toc380080259)

[Mujulii 8: Wabii Nyaataa Maatii: 5](#_Toc380080260)

[Kutaa 1: Ji’oota Maatiin Nyaata Gahaa Itti Argatan 5](#_Toc380080261)

[Kutaa 2: Madaallii dhiheennatti argama fi dhabiinsa wabii nyaataa maatii 6](#_Toc380080262)

[Mujulii 9: Koornayaa Fi Murtee Kennuu 9](#_Toc380080263)

[Kutaa 1: Itti Dhiheenna, Abbummaa , Fi Too’annoo Oomisha Qonnaa 9](#_Toc380080264)

[Kutaa 2: Itti Dhiheenna, Abbummaa, Fi Too’annoo Meeshaalee Yeroo Dheeraaf Turan 11](#_Toc380080265)

[Kutaa 3: Koornayaa Fi Yeroo Ramaduu/Qooduu 13](#_Toc380080266)

[MuJulii 10: Hirmaannaa Hawaasummaa Fi itti Dhiheenna Odeeffannoo 14](#_Toc380080267)

[kutaa 1: Hirmaannaa hawaasummaafi sadarkaa itti gamadiinsaa 14](#_Toc380080268)

[Kutaa 2: Madda Odeeffannoo Qonnaa 15](#_Toc380080269)

[Kutaa 3: Madda Odeeffannoo sirna Nyaataa 16](#_Toc380080270)

[Kutaa 4- Fudhatiinsaa Fi Itti Fayyadama Sagantaa Hojii Oomisha Qonna 17](#_Toc380080271)

[Kutaa 5 Itti Dhiheenya Misooma Bu’uuraa 21](#_Toc380080272)

[Mujulii 11 – Oomisha Qonnaa 22](#_Toc380080273)

[Kutaa 1: Abbummaa fi Itti Fayyadama Lafaa 22](#_Toc380080274)

[Kutaa 2: Oomisha midhaanii 22](#_Toc380080275)

[Kutaa 3: Baasii Midhaaniif Bahe 29](#_Toc380080276)

[Kutaa 4: Tilmaama Horii 31](#_Toc380080277)

[Kutaa 5: Horsiisa loonii 32](#_Toc380080278)

[Kutaa 6: Baasii Horsiisa Horii 34](#_Toc380080279)

[Kutaa 7: Gabaa Oomisha Midhaanii Fi Loonii 35](#_Toc380080280)

[Kutaa 8: Teeknoolojii Qonnaatii Fi itti fayyadama isaa 36](#_Toc380080281)

[Kutaa 9: Humna Namaa/Nama Hojiilee Qonnaa hundaaf Qaxarame 38](#_Toc380080282)

[Mujulii 12 – Galii fi Baasii 41](#_Toc380080283)

[Kutaa 1: Madda Galii Maatii Kan Biroo 41](#_Toc380080284)

# Mujulii 1: Odeeffannoo Fi Haala Maatii

## Kutaa 1: Odeeffannoo Gaafif Deebii

**Gabatee 1.1**

| **Lakkoofsa** | **Gaafii** | **Deebii** | **Variable name** |
| --- | --- | --- | --- |
|  | Guyyaa gaafif deebii (gg/jj/ww) | // | HDATEINT |
|  | Marsaa doo’annoo (1-13) |  | HDTIMEPT |
|  | Lakkoofsa manaa [HH ID] |  | HHID |
|  | Aanaa | *(galmee irraa ilaali/buusi: Wolisoo, Goommaa, XirooAfataa)* | HWOREDA |
|  | Ganda | *(barreessi )* | HKEBELE |
|  | Gooxii / Garee | *(barreessi)* | HGOTE |
|  | GPS | “Qindeessaa GPS Barbaadi”[biinkoo/ (button) | HHGPS |
|  | Maqaa itti gaafatamaa Maatii | Maqaa guuttuu | HHDNAMA |
|  | Dubartii qorannoo kanaaf Deebii kennitu itti gaafatamtuu maatiidhaa? | 1. Eeyyee 0. lakki | HHDINDWA |
|  | Eenyummaa gaafataa- 1 |  | HID1A |
|  | Eenyummaa gaafataa- 2 |  | HID2A |
|  | Eenyummaa Too’ataa |  | HSIDA |
|  | Bu’aa gaaffiif deebii | 1. Xumurte  2. Hin xumure  3.hin argamne  4. Hin didde  5.eddoo isee beekuu hin dandeenne | HINTOUTA |

## Kutaa 2: Waa’ee Beekumsa Itti Gaafatamaa Maatii

| **Lakk.** | **Gaafii** | **Deebii** | **Variable name** |
| --- | --- | --- | --- |
| 1.2.1 | Itti gaafatamaan maatii maqaa isaa afaan naannootiin barreessuu danda’aa? (maaloo mee qoradhu) | 1=eeyyee , 0=lakki  98=Hinqoratamne | HIMWRITE |
| 1.2.2 | Itti gaafatamaa/tuun maatii Hima kanatti aanu dubbisuu dandeessii/’aa?  (fakii barreeffama naannoo) | 1=eeyyee , 0=lakki 98=Hinqoratamne | HIMREAD |
| 1.2.3 | Itti gaafatamaa/tuun maatii gaafii herregaa itti aanu sirriin deebisuu dandeessii/’aa?  *“Osoo killee qarshii 30tti gurgurtee handaaqqos qarshii 50tti osoo gurgurte, walitti qarshii meeqa qabda?"* | 1=eeyyee, 0=lakki. 98=Hinqoratamne | HINUM |

# Kutaa 3: Qulqullinaa fi Bishaan [water & sanitation]

Gabatee 3.1

| Harka kandhiqattu Yeroo kam ? (Deebiikennaan akka deebisu godhi, sana booda deebii gaafii hundaa armaan gaditti guuti .) | | | |
| --- | --- | --- | --- |
| 1.3.1 | Gonkumaa | 1=eeyyee  0= lakki  98= hin beeku | WNIL |
| 1.3.2 | Xuriin mul’atu yoo jiraate | 1=eeyyee  0= lakki  98= hin beeku | WDIRT |
| 1.3.3 | Erga manfincaanii fayyadamee booda/booliin/fincaaniin | 1=eeyyee  0= lakki  98= hin beeku | WTOILETUSE |
| 1.3.4 | Daa’ima boolii baye qulqulleessuun booda | 1=eeyyee  0= lakki  98= hin beeku | WCLEANCHILD |
| 1.3.5 | Nyaata qopheessuun dura | 1=eeyyee  0= lakki  98= hin beeku | WFOOD |
| 1.3.6 | Nyaata dhiheessuun dura | 1=eeyyee  0= lakki  98= hin beeku | WMEAL |
| 1.3.7 | Nyaachuun dura | 1=eeyyee  0= lakki  98= hin beeku | WEAT |
| 1.3.8 | Daa’ima nyaachisuun dura | 1=eeyyee  0= lakki  98= hin beeku | WFEEDBABY |
| 1.3.9 | Yeroo yaadadhu | 1=eeyyee  0= lakki  98= hin beeku | WREMIND |

# Mujulii 8: Wabii Nyaataa Maatii:

## Kutaa 1: Ji’oota Maatiin Nyaata Gahaa Itti Argatan

Amma dhiyeessii nyaataa maatii keessanii waggaa keessatii kan ji’oota adda addaa sigaafachuun fedha. Gaafilee kanneeniif deebii yoo naafdeebistu, maaloo duubatti kan ji’a 12 yaadadhu, ji’a ammaa kanarraa jalqabii hanga waggaa darbee ji’a kanaatti .

**Gabatee 8.1: yeroo** maatiin nyaata gahaa itti argatan

|  | **Gaafii** | **Deebii** | **Maqaa Var.** |
| --- | --- | --- | --- |
| 8.1.1 | Ji’oota 12n darban keessatti, ji’oonni nyaata gahaa fedhii nyaataa maatii keessan itti guutuu dhabdan jiraa? | 1=eeyyee  0=lakki 98= hinbeeku | SGOOD |
| Eeyyee yoo jette, Ji’oota 12n darban keessatti, ji’oota kam turan yeroon nyaata gahaa fedhii nyaataa maatii keessan itti guutuu dhabdan? | | | |

**Ragaa sasaabduuf*:*** *gaafiin kun nyaata kamiiyyuu madda kamirraayyuuni ilaallata , kan akka ofiin oomishame, bitame/jijjiirrame, nyaata gargaarsaa, ykn liqeeffatame. Ji’oota barreeffaman hindubbisiin. Deebii kennaan ji’oota garagaraa akka yaadatan yoofeete mala lakkaawwii waqtiilee fayyadami. Deebii kennaan waa’ee ji’oota 12n darban hundaa akka yaadu mirkaneessuuf qoradhu.*

|  | **Ji’a** | **Deebii** |  |
| --- | --- | --- | --- |
| 8.1.2. | Amajjii | 1=eyyee  0=lakki 98=hin beeku | SJAN |
| 8.1.3. | Gurraandhala | 1=eyyee  0=lakki 98=hin beeku | SFEB |
| 8.1.4. | Bitootessa | 1=eyyee  0=lakki 98=hin beeku | SMAR |
| 8.1.5. | Ebla | 1=eyyee  0=lakki 98=hin beeku | SAPR |
| 8.1.6. | Caamsaa | 1=eyyee  0=lakki 98=hin beeku | SMAY |
| 8.1.7. | Waxabajjii | 1=eyyee  0=lakki 98=hin beeku | SJUN |
| 8.1.8. | Adoolessa | 1=eyyee  0=lakki 98=hin beeku | SJUL |
| 8.1.9. | Hagayya | 1=eyyee  0=lakki 98=hin beeku | SAUG |
| 8.1.10. | Fulbaana | 1=eyyee  0=lakki 98=hin beeku | SEPT |
| 8.1.11. | Onkololeessa | 1=eyyee  0=lakki 98=hin beeku | SOCT |
| 8.1.12. | Sadaasa | 1=eyyee  0=lakki 98=hin beeku | SNOV |
| 8.1.13. | Muddee | 1=eyyee  0=lakki 98=hin beeku | SDEC |

## Kutaa 2: Madaallii dhiheennatti argama fi dhabiinsa wabii nyaataa maatii

Torban afran darban keessatti waa’ee dhiheenna nyaata maatiikee irratti gaafii sigaafachuuf deema. Soorata kan akka nyaata idilee, mi’eessitoota fi nyaata kamiyyuu soorata maatii kee keessa jiru.

**Gabatee 8.2: Madaallii/safartuu dhiheennatti argama fi dhabiinsa wabii nyaataa maatii**

|  | **Gaafii** | **Deebii** | **Variable name** |
| --- | --- | --- | --- |
| 8.2.1 | Turban arfan darban keessatti ati ykn maatiin kee nyaata gahaa hinargadhu jechuudhaan sodaattanii beektuu? | 1=eeyyee  0=lakki 98= hin beeku | SWORRYM |
| 82.2 | Eeyyee yoo ta’e, yeroo hangamiif sodaan kun isinmuudate? | 1 = akka tasaa (torban arfan darbanitti al tokko /yeroo lama )  2 = darbee darbee ( torban arfan darbanitti yeroo 3 hand 10tti)  3 = yeroo baay’ee ( torban arfan darbanitti yeroo 10 ol ) | SWORRYFRQM |
| 8.2.3 | Torban arfan darban keessatti , ati ykn maatiin kee hanqina qabeenyaatiin akaakuu nyaata nyaachuu feetan osoo hin nyaatiin haftanii beektuu? | 1=eeyyee  0=lakki 98= hin beeku | SKINDM |
| 8.2.4 | Eeyyee yoo ta’e , yeroo hangamiif wanti kun isinmuudate/qunname? | 1 = akka tasaa (torban arfan darbanitti al tokko /yeroo lama )  2 = darbee darbee ( torban arfan darbanitti yeroo 3 hand 10tti)  3 = yeroo baay’ee ( torban arfan darbanitti yeroo 10 ol ) | SKINDFRQM |
| 8.2.5 | Torban arfan darban keessatti , ati ykn maatiin kee hanqina qabeenyaatiin kan ka’e gosa nyaataa muraasa qofa nyaattanii? | 1=Yes  0=No 98= hin beeku | SLIMITEDM |
| 8.2.6 | Eeyyee yoo ta’e , yeroo hangamiif wanti kun isinmuudate/qunname? | 1 = akka tasaa (torban arfan darbanitti al tokko /yeroo lama )  2 = darbee darbee ( torban arfan darbanitti yeroo 3 hand 10tti)  3 = yeroo baay’ee ( torban arfan darbanitti yeroo 10 ol ) | SLIMITEDFRQM |
| 8.2.7 | Torban arfan darban keessatti , ati ykn maatiin kee hanqina qabeenyaatiin kan ka’e akaakuu nyaata biroo argachuu dhabuun nyaata nyaachuu hinfeene nyaattanii turtanii? | 1=eeyyee  0=lakki 98= hin beeku | SDISLIKEM |
| 8.2.8 | Eeyyee yoo ta’e , yeroo hangamiif wanti kun isinmuudate/qunname? | 1 = akka tasaa (torban arfan darbanitti al tokko /yeroo lama )  2 = darbee darbee ( torban arfan darbanitti yeroo 3 hand 10tti)  3 = yeroo baay’ee ( torban arfan darbanitti yeroo 10 ol ) | SDISLIKEFRQM |
| 8.2.9 | Torban arfan darban keessatti , ati ykn maatiin kee nyaatni gahaan waan hinjirreef nyaata nyaachuu feetan gaditti nyaattanii turtanii? | 1=eeyyee  0=lakki 98= hin beeku | SMALLM |
| 8.2.10 | Eeyyee yoo ta’e, yeroo hangamiif wanti kun isin muudate/qunname? | 1 = akka tasaa (torban arfan darbanitti al tokko /yeroo lama )  2 = darbee darbee ( torban arfan darbanitti yeroo 3 hand 10tti)  3 = yeroo baay’ee ( torban arfan darbanitti yeroo 10 ol ) | SMALLFRQM |
| 8.2.11 | Torban arfan darban keessatti , ati ykn maatiin kee nyaatni gahaan waan hinjirreef guyyaatti nyaata bicuu nyaattanii turtanii? | 1=eeyyee  0=lakki 98= hin beeku | SFEWM |
| 8.2.12 | Eeyyee yoo ta’e , yeroo hangamiif wanti kun isin muudate/qunname? | 1 = akka tasaa (torban arfan darbanitti al tokko /yeroo lama )  2 = darbee darbee ( torban arfan darbanitti yeroo 3 hand 10tti)  3 = yeroo baay’ee ( torban arfan darbanitti yeroo 10 ol ) | SFEWFRQM |
| 8.2.13 | Torban arfan darban keessatti , ati ykn maatiin kee hanqina qabeenyaa nyaata ittin argatturraa kan ka’e yeroo waan nyaatamu kamiyyuu itti dhabdan jiraa? | 1=eeyyee  0=lakki 98= hin beeku | SNOFOODM |
| 8.2.14 | Eeyyee yoo ta’e , yeroo hangamiif wanti kun isin muudate/qunname? | 1 = akka tasaa (torban arfan darbanitti al tokko /yeroo lama )  2 = darbee darbee ( torban arfan darbanitti yeroo 3 hand 10tti)  3 = yeroo baay’ee ( torban arfan darbanitti yeroo 10 ol ) | SNOFOODFRQM |
| 8.2.15 | Torban arfan darban keessatti , ati ykn maatiin kee nyaatni gahaan waan hinjirreef halkan beela’aa raftanii beektuu? | 1=eeyyee  0=lakki 98= hin beeku | SLEEPM |
| 8.2.16 | Eeyyee yoo ta’e , yeroo hangamiif wanti kun isin muudate/qunname? | 1 = akka tasaa (torban arfan darbanitti al tokko /yeroo lama )  2 = darbee darbee ( torban arfan darbanitti yeroo 3 hand 10tti)  3 = yeroo baay’ee ( torban arfan darbanitti yeroo 10 ol ) | SLEEPFRQM |
| 8.2.17 | Torban arfan darban keessatti , ati ykn maatiin kee nyaatni gahaan waan hinjirreef halkaniif guyyaa tokko guutuu osoo nyaata kamiyyuu hin nyaatiin dabarsitanii beektuu? | 1=eeyyee  0=lakki 98= hin beeku | SNODAYM |
| 8.2.18 | Eeyyee yoo ta’e, yeroo hangamiif wanti kun isin muudate/qunname? | 1 = akka tasaa (torban arfan darbanitti al tokko /yeroo lama )  2 = darbee darbee ( torban arfan darbanitti yeroo 3 hand 10tti)  3 = yeroo baay’ee ( torban arfan darbanitti yeroo 10 ol ) | SNODAYFRQM |

#

# Mujulii 9: Koornayaa Fi Murtee Kennuu

## Kutaa 1: Itti Dhiheenna, Abbummaa , Fi Too’annoo Oomisha Qonnaa

Amma gaafilee muraasa waa’ee maatii kee ilaallaatu sigaafadha . Jalqaba, maatiin kee wantoota gabatee armaan gadii keessatti eeraman akka qabdan beekuun barbaada , amma ykn waqtii darbe (ji’a jahaan darban). Sana booda maatii kee keessaa eenyu akka ittiin hojjatu ykn fayyadamu baruun barbaada, eenyutu qaba, fi isaanirratti Murtii kan Kennuu eenyu akka ta’e

Gabatee 9.1

| **Lakk.** | **Gosa/akaakuu** | **Ni argamaa** (yoo lakki ta’e, gara gabatee mirgaatti darbi) | **Abbummaa** | **Too’achuu (bituuf/fayyadamuuf/gurguruuf kan murteessu)** | **Itti fayyadama galiirratti kan murteessu** |
| --- | --- | --- | --- | --- | --- |
|  |  | 1. Eeyyee 0. lakki | 1. dubartii 2. Dhiira  3. lameenu  4. kanbiraa | 1. Dubartii 2. Dhiira  3. lameenu | 1. Dubartii 2. Dhiira  3. lameenu  4. hinsakkatta’amne |
| 9.1.1. | Lafa qonnaa | GLANDA | GLANDO | GLANDC | GLANDD |
| 9.1.2. | itti fayyadama lafa qonnaa |  |  | GLANDUSEC |  |
| 9.1.3. | lafa waan naannoo manatti oomishamuuf oolu | GGARDA | GGARDO | GGARDC | GGARDD |
| 9.1.4. | Lafa qonnaaf hin oollee(kan daldalaaf jireenyaaf oolu) | GNALANDA | GNALANDO | GNALANDC | GNALANDD |
| 9.1.5. | Midhaan nyaataa | GCEREALA | GCEREALO | GCEREALC | GCEREALD |
| 9.1.6. | Mukaa fuduraa | GBANANA | GBANANAO | GBANANAC | GBANANAD |
| 9.1.7. | Hiddaa/jirma | GROOTSA | GROOTSO | GROOTSC | GROOTSD |
| 9.1.8. | Midhaan gurgurtaaf oolan(buna,caatii KKF) | GCASHCA | GCASHCO | GCASHCC | GCASHCD |
| 9.1.9. | Muduraalee | GVEGA | GVEGO | GVEGC | GVEGD |
| 9.1.10. | Midhaan dheedhii (Atara, baaqelaa kkf) | GPULSA | GPULSO | GPULSC | GPULSD |
| 9.1.11. | Midhaan zeeyitaa | GOILSA | GOILSO | GOILSC | GOILSD |
| 9.1.12. | Mi’eesituu/kan baalli isaan fayyadu | GSPICA | GSPICO | GSPICC | GSPICD |
| 9.1.13. | Loon | GCATTLEA | GCATTLEO | GCATTLEC | GCATTLED |
| 9.1.14. | Farda,harree, gangee | GMULEA | GMULEO | GMULEC | GMULED |
| 9.1.15. | Hoolaa/re’ee | GRUMINA | GRUMINO | GRUMINC | GRUMIND |
| 9.1.16. | Horsiisa lukkuu | GPOULTRYA | GPOULTRYO | GPOULTRYC | GPOULTRYD |
| 9.1.17. | Kannisa horsiisuu | GBEEHA | GBEEHO | GBEEHC | GBEEHD |
| 9.1.18. | Manaa fi ijaarsa biroo | GHOUSEA | GHOUSEO | GHOUSEC | GHOUSED |
| 9.1.19. | Meeshaalee qonnaa ammayyaa kan hin taane | GFEQUIPNMA | GFEQUIPNMO | GFEQUIPNMC | GFEQUIPNMD |
| 9.1.20. | Meeshaalee qonnaa kan ammayyaa | GFEQUIPMA | GFEQUIPMO | GFEQUIPMC | GFEQUIPMD |
| 1.9.21. | Meeshaa fayyidaa qonnaan alaatiif oolan | GBUSEQUIPA | GBUSEQUIPO | GBUSEQUIPC | GBUSEQUIPD |

*koodii 1. itti gaafatamaa maatii 2.abbaa 3. Niitii duraa 4. Haadha 5. niitii lammaffaa 6. kan biraa 7. Hundaa 8. hin beekamu –[ kan ilaalatu hundaa filadhu]

## Kutaa 2: Itti Dhiheenna, Abbummaa, Fi Too’annoo Meeshaalee Yeroo Dheeraaf Turan

Amma waa’ee meeshaalee mana keessaa sigaafadha. Maaloo mee yoo meeshaleen kunneen amma mana keessan keessatti yoo jiraatan ta’e natti himuun, eenyu akka itti fayyadamu, kan eenyu akka ta’anii fi fayyadama isaanii eenyu akka too’atu.

| **Lakk. Gaafii** |  | **Ni argama** | **Baay’inna argamu**  **(laakkofsa)** | **Baay’inna kan hojjatuu**  **(laakkofsa)** | **Abbummaa(kan hin hojjanne)**  **Koodii kanaan gadii ilaali** | **Abbummaa(kan hojjatu)**  **Koodii kanaan gadii ilaali** | **Itti dhiheenna(kan hojjatu)**  **Koodii kanaan gadii ilaali** | **Itti fayyadama kan too’atu**  **(kan hojjatu)**  **Koodii kanaan gadii ilaali** |
| --- | --- | --- | --- | --- | --- | --- | --- | --- |
|  | **Meeshaalee** | (1=eeyyee ,0=lakki) lakki yoo ta’e, gabatee gara mirgaatti darbi |  |  | 1. dubartii  2. Dhiira  3. lameenu  4. kanbiraa |  |  | 1. dubartii  2. Dhiira  3. lameenu  4. kanbiraa |
| 2.1.1 | Raadiyoo | GRADIOA | GRADIOQ | GRADIOQF | GRADIONF | GRADIOO | GRADIOS | GRADIOC |
| 2.1.2 | Televiziyoona | GTVA | GTVQ | GTVQF | GTVNF | GTVO | GTVS | GTVC |
| 2.1.3 | Bilbila manaa/Bilbila kansarara dhaabbataa | GTELEA | GTELEQ | GTELEQF | GTELENF | GTELEO | GTELES | GTELEC |
| 2.1.4 | Bilbila mobaayilii | GMOBILA | GMOBILQ | GMOBILQF | GMOBILNF | GMOBILO | GMOBILS | GMOBILC |
| 2.1.5 | Biskileetii | GBICA | GBICQ | GBICQF | GBICNF | GBICO | GBICS | GBICC |
| 2.1.6 | Motor saayikilii | GMOTOA | GMOTOQ | GMOTOQF | GMOTONF | GMOTOO | GMOTOAS | GBODAC |
| 2.1.7. | Baajaajii | GBAJAJAA | GBAJAJQ | GBAJAJQF | GBAJAJNF | GBAJAJO | GBAJAJS | GBAJAJC |
| 2.1.8 | Gaarii | GCARTA | GCARTQ | GCARTQF | GCARTNF | GCARTO | GCARTS | GCARTC |

*koodii 1. itti gaafatamaa maatii 2.abbaa 3. Niitii duraa 4. Haadha 5. niitii lammataa 6. kan biraa 7. Hundaa 8. hin beekamu –[ kan ilaalatu hundaa filadhu]

## Kutaa 3: Koornayaa Fi Yeroo Ramaduu/Qooduu

Kaleessa yeroo kee akkamitti akka itti fayyadamtee sigaafachuu barbaada. Kaleessa yeroo kee akkamitti dabarsite, yeroo hirribaa kaaterraa hanga raftetti?

|  | | ***Filannoo tokko filadhu:*** | | |
| --- | --- | --- | --- | --- |
|  |  | 1.   hojii qonnaa midhaan irratti | | |
|  |  | 2.Hojii hormaata horii | | |
|  |  | 3.   Hojii qonnaan alaa kan dhuunfaa koo irratti | | |
|  |  | 4.Hojii qonnaan alaa irratti kafaltiidhaan | | |
|  |  | 5.karaa deemeen ture(imala irra ture)(dhaqaaf gala) | | |
|  |  | 6.Bittaa meeshaa/tajaajila adda addaa (fayyaaa dabalatee) | | |
|  |  | 7.Baruumsaaf ykn leenjii irratti | | |
|  | | 8. hojiii mana keessaa ykn ijoollee/hangafoota/dhukkubsattoota wajjiin  9. yeroo dhuunfaa(boqqonna, yeroo amantaa, yeroo hawaasummaa, yeroo nyaata) | | |
| **Yeroo /sa’aa** | | **Itti Gaafatamaa Manaa/abbaa manaa** |  | |
| 00:00 –halkan walakkaa | | GTIMEM00 |  | |
| 7:00 AM | | GTIMEM01 |  | |
| 8:00 AM | | GTIMEM02 |  | |
| 9:00 AM | | GTIMEM03 |  | |
| 10:00 AM | | GTIMEM04 |  | |
| 11:00 AM | | GTIMEM05 |  | |
| 12:00 AM | | GTIMEM06 |  | |
| 1:00 AM ganama | | GTIMEM07 |  | |
| 2:00 AM | | GTIMEM08 |  | |
| 3:00 AM | | GTIMEM09 |  | |
| 4:00 AM | | GTIMEM10 |  | |
| 5:00 AM | | GTIMEM11 |  | |
| 6:00 **Guyyaa/saafaya** | | GTIMEM12 |  | |
| 7:00 PM | | GTIMEM13 |  | |
| 8:00 PM | | GTIMEM14 |  | |
| 9:00 PM | | GTIMEM15 |  | |
| 10:00 PM | | GTIMEM16 |  | |
| 11:00 PM | | GTIMEM17 |  | |
| 12:00 PM **galgala** | | GTIMEM18 |  | |
| 1:00 PM | | GTIMEM19 |  | |
| 2:00 PM | | GTIMEM20 |  | |
| 3:00 PM | | GTIMEM21 |  | |
| 4:00 PM | | GTIMEM22 |  | |
| 5:00 PM | | GTIMEM23 |  | |
|  | |  |  | |

# MuJulii 10: Hirmaannaa Hawaasummaa Fi itti Dhiheenna Odeeffannoo

# **kutaa 1: Hirmaannaa hawaasummaafi sadarkaa itti gamadiinsaa**

Ati ykn miseensonni maatii keetii amma gareewwan hawaasummaa kan asii gaditti eeraman keessatti hirmaataa jirtaa/jiru?

**Gabatee 10.1**

|  | **Garee** | Dhiira ijoo (1=eeyyee 0=lakki )  98= hin beeku | Miseensota biro (1=eeyyee 0=lakki)98= hin beeku |
| --- | --- | --- | --- |
| 10.1.1. | Waldaa liqii fi qusanoo badiya | GFARMM | GFARMO |
| 10.1.2. | Garee gargarsaa dubartootaa | GWOMM | GWOMO |
| 10.1.3. | Gareewwan amantaa | GRELIGM | GRELIGO |
| 10.1.4. | Gareewwan dargaggootaa | GYOUTHM | GYOUTHO |
| 10.1.5. | Waldaa qonan bultota/omishtota | GHEALTHM | GHEALTHO |
| 10.1.6. | Koree gandaa ( bulchiinsaa , fayyaa , kanbiroo) | GKEBELEM | GKEBELEO |
| 10.1.7. | Garee maallaqaan walqabatan (iddirii, iqqubii , garee maallaqaa xixiqqoo kkf) | GFINM | GFINO |
| 10.1.8 | Garee biraa (ibsi___________) GOTHSPE | GOTHM | GOTHO |

## Kutaa 2: Madda Odeeffannoo Qonnaa

Ji’oota sadan dhumaa darban keessatti, ati (kunuunsaan) odeeffannoo waa’ee qonnaa kamiiyyuu haata’uu (fakkeenyaaf, gorsa waa’ee lafa kunuunsuu, fayyadama xaa’oo, facaasuu/dhaabuu) maddoota asii gaditti eeraman irra argattee beektaa?

**Gabatee 10.2**

| **Madda odeeffannoo** | **Deebii** | **Variable** |  |
| --- | --- | --- | --- |
| Raadiyoo | 1=eeyyee  0=lakki | IARADIO |  |
| Gaazexaa | 1=eeyyee  0=lakki | IANPAPER |  |
| Bilbila mobaayilii | 1=eeyyee  0=lakki | IAMOBPHONE |  |
| Televiziyoona | 1=eeyyee  0=lakki | IATV |  |
| Poostara(beeksis fakii qabu)/liifleetii/barraffama gabaabduu kan raabsamtu | 1=eeyyee  0=lakki | IAPOSTER |  |
| Waldaa liqii fi qusanoo badiya | 1=eeyyee  0=lakki | IAGFG |  |
| Garee maallaqaan walqabatan (iddirii, iqqubii , garee maallaqaa xixiqqoo kkf) | 1=eeyyee  0=lakki | IAGSG |  |
| Waldaa qonan bultota/omishtota | 1=eeyyee  0=lakki |  |  |
| Garee amantaa | 1=eeyyee  0=lakki |  |  |
| Hojjattota misooma qonnaa (DA) ykn misooma ekisteenshinii qonnaa | 1=eeyyee  0=lakki | IAAGENTS |  |
| Hojjataa mana kuusaa | 1=eeyyee  0=lakki | IAGAC |  |
| Daldaltoota gabaadha bitan/kan qinxaaboon gurguran | 1=eeyyee  0=lakki | IAGMB |  |
| Dhaabbilee mitimootummaa (kanbiyya keessaa ykn biyya alaa) | 1=eeyyee  0=lakki | IANGO |  |
| Marii hawaasaa | 1=eeyyee  0=lakki | IAGCC |  |
| Tokko-shanee | 1=eeyyee  0=lakki | IAG125 |  |
| kanbiroo (maatii/ollaa/hiriyaa) | 1=eeyyee  0=lakki | IAOTHER |  |

##

## Kutaa 3: Madda Odeeffannoo sirna Nyaataa

Ji’oota sadeen darban keessatti, maddoota asii gaditti eeraman irraa odeeffannoo kamiyyuu haata’u sirna nyaata irratti ofiikeetiif ykn maatii keettiif argattee turte?

**Gabatee 10.3**

| Raadiyoo | 1=eeyyee 0=lakki | **Variable** |
| --- | --- | --- |
| Gaazexaa | 1=eeyyee  0=lakki | INRADIO |
| Bilbila mobaayilii | 1=eeyyee  0=lakki | INNPAPER |
| Televiziyoona | 1=eeyyee  0=lakki | INMOBPHONE |
| Poostara(beeksis fakii qabu)/liifleetii/barraffama gabaabduu kan raabsamtu | 1=eeyyee  0=lakki | INTV |
| Garee hawaasumma ykn kan amantaa [garee fayyaadhaan walqabate | 1=eeyyee  0=lakki | INPOSTER |
| Ejantii Ekisteenshinii fayyaa(**VHT**, gargaartota fayyaa hawaasaa , barreessitoota fayyaa) | 1=eeyyee  0=lakki | INGP |
| Komitee gandaa | 1=eeyyee  0=lakki | INAGENTS |
| Dhaabbilee fayyaa[kan hawwaasaa ykn dhuunfaa | 1=eeyyee  0=lakki | INKHC |
| Dhaabbilee mitimootummaa (kanbiyya keessaa ykn biyya alaa) | 1=eeyyee  0=lakki | INFAC |
| Marii hawaasaa | 1=eeyyee  0=lakki | INNGO |
| Tokko-shanee | 1=eeyyee  0=lakki | INCC |
| kanbiroo (maatii/ollaa/hiriyaa) | 1=eeyyee  0=lakki | INOTHER |

## Kutaa 4- Fudhatiinsaa Fi Itti Fayyadama Sagantaa Hojii Oomisha Qonna

| 1. At (miseensi maatii ke)ji’oota ja’an darban keessati hojiile armaan gadiitti hirmaate turte   0=lakki 1=eyyee 98=hin beeku  Yoo eyyee (hin bku ta’e) gara ‘C’ tti darbi | | 1. yoo lakki ta’e maaliif kan deeb ta’an hunda filadhu 0=hojichi hin ture   1=yeroon dhabe  2,sababa fageenya  3,Bayyee mi’ayyaa waan ta’eef  4,fedhii dhabuu  5,fayyida waan hin qabneef  6,kan biraa  98=hin beeku | 1. C. Maada odefanno (debii kan ta’an hunda filadhu)   1=hojjattoota extension qonnaa  2= hoogganaa qonnaa  3= ogeessa fayyaa loonii  4= marri hawaasaa  5=garee hawassuma /amnata  6=dhabata mitti motumma  7=Radiyoo  8=televiziyoona  9=barulle/gazexa  10=bilibila mobayela  11=barefamafi fakille adda adda  12= hiriyaa/firaa  13=kan biro  98=hinbekuu | D. ji’oota 6n darban keessatti yeroo ammamiif atti (miseensi )maatiike hojii irrati hirmaata | | 1. sababa hojiilee kanaatiin atti (miseensa maatii) ke keessa jijiiram ammala kan fide jiraa?   0=lakki  1,eyyeen  98=hin beeku | F. yoolakki ta’emaaliif (kan deebi Ta’an hunda filadhu  1,yeroo dhabuu  2,bayyee mi’aa ta’u  3,fedhii dhabuu  4,fayyida hin qabu  5,kan biro   1. 98=hin beeku |
| --- | --- | --- | --- | --- | --- | --- | --- |
|  |  |  |  | Lakkoofsa  (98 yoo lakki ta’e) | Koodii :  1=torban  2=ji’a  3=ji’a 6n darban |  |  |
| Waa’ee sagantaa AGP dhageessee beektaa?  Yoo eyyee ta’ e gara C.tti darbi |  |  |  |  |  |  |  |
| Eeyyee yoo ta’e, maatiin keessan kallattiidhaan sagantaa AGP fayyadamoodhaa? |  |  |  |  |  |  |  |
| Odeefanno (leenjii) wa’ee qonnaa/ qonna hammayyaa fudhattee beektaa? |  |  |  |  |  |  |  |
| Odeefanno (leenjii) wa’ee  Sanyii haaraa adda addaa: |  |  |  |  |  | Odeeffannoo/leenjii argateen Gosa sanyii addaa fayyadamtee? |  |
| *Xaafii* |  |  |  |  |  |  |  |
| *Garbuu* |  |  |  |  |  |  |  |
| *Boqqoolloo* |  |  |  |  |  |  |  |
| *Mishingaa* |  |  |  |  |  |  |  |
| *Baaqelaa ykn atara* |  |  |  |  |  |  |  |
| *Shunkurtii* |  |  |  |  |  |  |  |
| *Timaatima* |  |  |  |  |  |  |  |
| *Kaarotii* |  |  |  |  |  |  |  |
| *Goommana/raafuu* |  |  |  |  |  |  |  |
| *Kan biro [ibsi]* |  |  |  |  |  |  |  |
| Odeefanno (leenjii) wa’ee asii gadiirratti fudhattee? |  |  |  |  |  | Odeeffannoo/leenjii argatteen haala itti hojjattu jijjiirtee? |  |
| *Oomisha dammaa (Apiary management/apiculture* |  |  |  |  |  |  |  |
| *Gabbiisa horii xixiqqoo kan akk hoolaaf re’ee* |  |  |  |  |  |  |  |
| *Gabbisa loonii* |  |  |  |  |  |  |  |
| *Kunuunsa loonii (fayyaa loonii,loon nyaachisuu]* |  |  |  |  |  |  |  |
| *Oomisha loon irraa argamu products (ittoo sa;aa, aannan, dhadhaa, itittuu)* |  |  |  |  |  |  |  |
| *Itti fayyadame bishaanii fi jallisii* |  |  |  |  |  |  |  |
| *Oomisha boroo* |  |  |  |  |  |  |  |
| Karaa qindaayeen ilbiisota too’achuu |  |  |  |  |  |  |  |
| *Tarkaanfii Ittisa biyyoo* |  |  |  |  |  |  |  |
| *- kaaba kaabuu (embarkment along the contour which is made of soil or stones)* |  |  |  |  |  |  |  |
| *- Stone bunds* |  |  |  |  |  |  |  |
| *- soil bunds* |  |  |  |  |  |  |  |
| *Kunuunsa biyyoo* |  |  |  |  |  |  |  |
| *Dhiqama biyyoo* |  |  |  |  |  |  |  |
| *Xaa’oo keemikaalaa fayyadamuu* |  |  |  |  |  |  |  |
| *Sararaan facaasuu* |  |  |  |  |  |  |  |
| *Gabaarratti/iddoo/wiirtuu itti sassaaban* |  |  |  |  |  |  |  |
| *Tooftaa gabaa irraatti* |  |  |  |  |  |  |  |
| *Gatii too’achuu* |  |  |  |  |  |  |  |
| *Gamtaa qotee bultootaa/VSLA* |  |  |  |  |  |  |  |
| Kan armaan gadii hojjattee/ilaaltee/doo’attee: |  |  |  |  |  | Beekumsa argatte hojii qonnaa keetiif oolchitee? |  |
| *Leenjii qotee bultootaa* |  |  |  |  |  |  |  |
| *Agarsiisa qonnaa* |  |  |  |  |  |  |  |
| Sanyii haaraa qonnaa adda addaa fudhattee beektaa? |  |  |  |  |  | Gosoota fayyadamtee/ fayyadamuuf karoora qabdaa? |  |
| *Xaafii* |  |  |  |  |  |  |  |
| *Garbuu* |  |  |  |  |  |  |  |
| *Boqqoolloo* |  |  |  |  |  |  |  |
| *Mishingaa* |  |  |  |  |  |  |  |
| *Baaqelaa ykn atara* |  |  |  |  |  |  |  |
| *Shunkurtii* |  |  |  |  |  |  |  |
| *Timaatima* |  |  |  |  |  |  |  |
| *Kaarotii* |  |  |  |  |  |  |  |
| *Goommana/raafuu* |  |  |  |  |  |  |  |
| *Kan biro [ibsi]* |  |  |  |  |  |  |  |
| *Kan armaan gadii kamiyyuu fudhattee :* |  |  |  |  |  |  |  |
| *Xaa’oo keemikaalaa* |  |  |  |  |  |  |  |
| *Lukkuu* |  |  |  |  |  |  |  |
| *Re’ee* |  |  |  |  |  |  |  |
| *Loon* |  |  |  |  |  |  |  |
| *Loon biraa (ibsi )* |  |  |  |  |  |  |  |
| *Sooyiraa biroo (ibsi)* |  |  |  |  |  |  |  |
| *Kan jallisiif fayyadu* |  |  |  |  |  |  |  |
| *Horsiisa kanniisaa* |  |  |  |  |  |  |  |
| *Liqii maaykiroo qonnaaf oolu* |  |  |  |  |  |  |  |
| *Liqii maaykiroo waan biraatiif oolu* |  |  |  |  |  |  |  |
| *Ati ykn hawaasni keessan gargaarsa asii gadii argatanii:* |  |  |  |  |  |  |  |
| *Iddoo gabaan itti gaggeeffamu* |  |  |  |  |  |  |  |
| *Tooftaalee gabaa* |  |  |  |  |  |  |  |
| *Gatii too’achuu* |  |  |  |  |  |  |  |
| *Waldaalee (VSLA, farmer)* |  |  |  |  |  |  |  |
| *Liqii maaykiroo interpiraayizii* |  |  |  |  |  |  |  |

**Sagalee olqabii dubbisi:** Amma waa’ee itti dhiheenna hojjattoota ogeessa qonnaa/loonii qabduun sigaafachuu barbaada

| **Gaafii** | **Deebii** |  |
| --- | --- | --- |
| Ji’oota 12n darban keessatti hojjattoota ogeessa qonnaa waliin walqunnamtee beektaa? | Eeyyee 1  lakki 2 |  |
| Ji’oota 12n darban keessatti hojjattoota ogeessa qonnaa yeroo meeqa isin doo’atan? | baay’ina lakkoosa walqunnamte |  |
| Do’annoo kanatti gammaddee? | Baay’een itti walii gala  walii gala  hin murteessu  ittiin walii hingalu  Baay’een ittiin walii hingalu | 1. Dhabiinsa yeroo 2. Dhabiinsa odeeffannoo gahaa 3. Lakkoofsa doo’annoo muraasa ta’a 4. Hin beeku |
| Saalli hojjattaan/tuun ogeessa qonnaa yeroo xumuraatiif isin doo’ate/tte maali? | Dhiira 1  Dubara 2 |  |
| Ji’oota 12n darban keessatti hojjattoota ogeessa loonii waliin walqunnamtee beektaa? | Eeyyee 1  lakki 2 |  |
| Ji’oota 12n darban keessatti hojjattoota ogeessa loonii yeroo meeqa isin doo’atan? | baay’ina lakkoosa walqunnamte |  |
| Do’annoo kanatti gammaddee? | Baay’een itti walii gala  walii gala  hin murteessu  ittiin walii hingalu  Baay’een ittiin walii hingalu | 1. Dhabiinsa yeroo 2. Dhabiinsa odeeffannoo gahaa 3. Lakkoofsa doo’annoo muraasa ta’a 4. Hin beeku |
| Saalli hojjattaan/tuun ogeessa loonii yeroo xumuraatiif isin doo’ate/tte maali? | Dhiira 1  Dubara 2 |  |

## Kutaa 5 Itti Dhiheenya Misooma Bu’uuraa

| Mana keetirraa miilaan gara dhaabbilee eeraman kanneenitt si’a tokko deemtee deebi’uuf hangam sijalaa fudhata? | |
| --- | --- |
| Mana baruumsa sad.1ffaa dhihoo | **Yeroo fudhate [sa’aa; daqiiqaa}** |
| Kilinika dhihoo |  |
| Kilinika beelladaa dhihoo |  |
| Karaa guddaa isa dhihoo |  |
| Karaa miilaa guddaa isa dhihoo |  |
| Gabaa naannoo dhihoo |  |
| Gabaa guddaa naannoo dhihoo |  |
| Waajjira hojjataa misooma qonnaa dhihoo |  |
| Dhaabbata leenjii qonnaa dhihoo |  |
| Magaalaa aanaa dhihoo |  |
| Baankii daldalaa dhihoo |  |
| Dhaabbata maaykiroo faayinaansii dhihoo |  |

# Mujulii 11 – Oomisha Qonnaa

## Kutaa 1: Abbummaa fi Itti Fayyadama Lafaa

Jalqaba deebii kennaan akka haala itti fayyadama lafaa maatii isaa kan bara **2013**Amajjii – Waxabajjii akka ibsu gaafadhuu deebii gabatee asiigaditti guuti. Sanaan booda akka haala itti fayyadama lafaa maatii isaa kan bara **2012, Adoolessaa-Muddee akka ibsu gaafachuun deebii gabatee kanatti aanutti guuti.**

***Gabatee ???***

|  |  | **Amajjii – waxabajjii 2013**  **lakkoofsa safartuu *** | | **Adoolessa – muddee 2012**  **lakkoofsa safartuu *** | |
| --- | --- | --- | --- | --- | --- |
| 11.1.1. | Lafa dhuunfaa kan qotame | LANDOWN6 | LANDOWN6U | LANDOWN12 | LANDOWN12U |
| 11.1.2. | Lafa kireeffatame/ergifatame kan qotame | LANDRENTIN6 | LANDRENTIN6U | LANDRENTIN12 | LANDRENTIN12U |
| 11.1.3. | kan namaan walitti qaban kan qotame | LANDRENTOUT6 | LANDRENTOUT6U | LANDRENTOUT12 | LANDRENTOUT12U |
| 11.1.4. | Dimshaasha lafa qotamee | *LANDSHARE6* | *LANDSHARE6U* | *LANDSHARE12* | *LANDSHARE12U* |
| 11.1.5. | Lafa dhuunfaa kan hin qotamne | LANDARAB6 | LANDARAB6U | LANDARAB12 | LANDARAB12U |
| 11.1.6. | Lafa kireeffame | LANDCULT6 | LANDCULT6U | LANDCULT12 | LANDCULT12U |
| 11.1.7 | Lafa odoo/booroo manaa | LANDGARD6 | LANDGARD6U | LANDGARD12 | LANDGARD12U |
| 11.1.8 | Lafa qabdu dimshaashaan | *LANDTOT6* | *LANDTOT6U* | *LANDTOT12* | *LANDTOT12U* |

*filannoon safartuu kan dabalatu: hektaara, skiweer meetira, Timad, , facaasaa, sangaa,kan biroo]

## Kutaa 2: Oomisha midhaanii

**Ragaa sasaabduuf:** jalqaba, midhaan maatiin ji’a Adoolessa bara 2012 hanga Waxabajjii 2013tti oomishan addaan baasi. . Sanaan booda, deebii kennaan akka waa’ee oomisha midhaanii ji’a Amajjii hanga Waxabajjii 2013tti [waqtii darbe] yaadatu gaafachuun gabatee armaan gaditti guuti. Sanaan booda, deebii kennaan akka waa’ee oomisha midhaanii ji’a Adooleessa hanga Muddee bara 2013tti yaadatu gaafachuun ammas gabatee armaan gaditti guuti.

**Gabatee 11.2: Oomisha Midhaanii**

| **Gosa** | **Lakk. gaafii** | | **midhaan** | | | | **Kan ji’oota 12 darban keessatti gahan? (1=eeyyee, 0=lakki)** | | |  | | **Bal’ina[gatii]**  **Area Value)** | | **Bal’ina**  **[safartuu]**  **Area (unit)** | **Waliin makanii facaasuu**  **Intercropped (1=eeyye, 0=lakki)** | | | **Eeyyee yoo jette,midhaan ijoo 2ffaa main 2nd crop?** | **Oomisha wolitti qabuu [harvest]** | | | | | | | | | | |  | **Kan hojiirra oole [DISPOSITION]** | | | | | | | | | | | **Gatii tokko tokkon isaanii safartuun (Birr))** | | **Gatii dimshaashaa kan oomishaa (Birr)** | **Too’annoo [bituuf/fayyadamuuf/gurguruuf/ kan murtee kennu**  **Lakkoofsa eenyummaa galmee miseensota maatii** | | **Itti fayyadama galiirratti kan murteessu**  **Lakkoofsa eenyummaa galmee miseensota maatii** | |
| --- | --- | --- | --- | --- | --- | --- | --- | --- | --- | --- | --- | --- | --- | --- | --- | --- | --- | --- | --- | --- | --- | --- | --- | --- | --- | --- | --- | --- | --- | --- | --- | --- | --- | --- | --- | --- | --- | --- | --- | --- | --- | --- | --- | --- | --- | --- | --- | --- |
|  |  |  |  |  |  |  |  |  |  | **waqiitti** | |  |  |  |  |  |  |  | **Midhaan hin sassaabamne dhibbantaan/percent** | | **midhha** | | **Jooniyaa/quumxa abbaa kiilograama meeqaa fayyadamte**  **?**  **Tokko filadhu 1=abbaa KG 100**  **2.Abbaa Kg 50**  **3.abbaa Kg 25**  **4. lakkofsan**  **5. Zorbaa**  **6. kanbiroo** | | **Ulfinna safartuu (kg)**  **Dhiheessa** | | | **Dimshaasha oomisha wolitti qabamee(Kg)** | |  | **Baay’inna maatiin nyaate safartuudhaan Number units consumed by HH** | | **Baay’inni gurgurame safartuudhaan** | **Baay’inni miidhame/bade,nyaata horiitiif kan oole** | | **Baay’’inna sanyiidhaaf olkaa’ame/kuufamee** | | | **Baay’inna namaaf kennamee kafaltiif ykn jijjiiraan kenname** | | |  |  |  |  |  |  |  |
| Midhaan/cereals | 11.2.1 | | Boqqoollo | | | |  | | |  | |  | |  |  | | |  |  | | | |  | |  | | |  | |  |  | |  |  | |  | | |  | | |  | |  |  | |  | |
|  |  |  |  |  |  |  |  |  |  |  | |  | |  |  | | |  |  | | | |  | |  | | |  | |  |  | |  |  | |  | | |  | | |  | |  |  | |  | |
|  | 11.2.2 | | Xaafii | | | |  | | |  | |  | |  |  | | |  |  | | | |  | |  | | |  | |  |  | |  |  | |  | | |  | | |  | |  |  | |  | |
|  |  |  |  |  |  |  |  |  |  |  | |  | |  |  | | |  |  | | | |  | |  | | |  | |  |  | |  |  | |  | | |  | | |  | |  |  | |  | |
|  | 11.2.3 | | Qamadii | | | |  | | |  | |  | |  |  | | |  |  | | | |  | |  | | |  | |  |  | |  |  | |  | | |  | | |  | |  |  | |  | |
|  |  |  |  |  |  |  |  |  |  |  | |  | |  |  | | |  |  | | | |  | |  | | |  | |  |  | |  |  | |  | | |  | | |  | |  |  | |  | |
|  | 11.2.4 | | Garbuu | | | |  | | |  | |  | |  |  | | |  |  | | | |  | |  | | |  | |  |  | |  |  | |  | | |  | | |  | |  |  | |  | |
|  |  |  |  |  |  |  |  |  |  |  | |  | |  |  | | |  |  | | | |  | |  | | |  | |  |  | |  |  | |  | | |  | | |  | |  |  | |  | |
|  | 11.2.5 | | Mishingaa | | | |  | | |  | |  | |  |  | | |  |  | | | |  | |  | | |  | |  |  | |  |  | |  | | |  | | |  | |  |  | |  | |
|  |  |  |  |  |  |  |  |  |  |  | |  | |  |  | | |  |  | | | |  | |  | | |  | |  |  | |  |  | |  | | |  | | |  | |  |  | |  | |
|  |  |  |  |  |  |  |  |  |  |  | |  | |  |  | | |  |  | | | |  | |  | | |  | |  |  | |  |  | |  | | |  | | |  | |  |  | |  | |
|  | 11.2.6 | | Ajjaa /”Aja” | | | |  | | |  | |  | |  |  | | |  |  | | | |  | |  | | |  | |  |  | |  |  | |  | | |  | | |  | |  |  | |  | |
|  |  |  |  |  |  |  |  |  |  |  | |  | |  |  | | |  |  | | | |  | |  | | |  | |  |  | |  |  | |  | | |  | | |  | |  |  | |  | |
|  |  |  |  |  |  |  |  |  |  |  | |  | |  |  | | |  |  | | | |  | |  | | |  | |  |  | |  |  | |  | | |  | | |  | |  |  | |  | |
|  | 11.2.7 | | Midhaan biroo *ibsi______*  *(LCLOTH)* | | | |  | | |  | |  | |  |  | | |  |  | | | |  | |  | | |  | |  |  | |  |  | |  | | |  | | |  | |  |  | |  | |
|  |  |  |  |  |  |  |  |  |  |  | |  | |  |  | | |  |  | | | |  | |  | | |  | |  |  | |  |  | |  | | |  | | |  | |  |  | |  | |
| Jirmi/hiddi kan nyaatamu | 11.2.8 | | Mixaaxisha/beets | | | |  | | |  | |  | |  |  | | |  |  | | | |  | |  | | |  | |  |  | |  |  | |  | | |  | | |  | |  |  | |  | |
|  |  |  |  |  |  |  |  |  |  |  | |  | |  |  | | |  |  | | | |  | |  | | |  | |  |  | |  |  | |  | | |  | | |  | |  |  | |  | |
|  | 11.2.9 | | Dinnicha | | | |  | | |  | |  | |  |  | | |  |  | | | |  | |  | | |  | |  |  | |  |  | |  | | |  | | |  | |  |  | |  | |
|  |  |  |  |  |  |  |  |  |  |  | |  | |  |  | | |  |  | | | |  | |  | | |  | |  |  | |  |  | |  | | |  | | |  | |  |  | |  | |
|  | 11.2.11 | | Kaazaavaa | | | |  | | |  | |  | |  |  | | |  |  | | | |  | |  | | |  | |  |  | |  |  | |  | | |  | | |  | |  |  | |  | |
|  |  |  |  |  |  |  |  |  |  |  | |  | |  |  | | |  |  | | | |  | |  | | |  | |  |  | |  |  | |  | | |  | | |  | |  |  | |  | |
|  | 11.2.12 | | Mixaaxisha haalluu Sweet potato/yam – orange | | | |  | | |  | |  | |  |  | | |  |  | | | |  | |  | | |  | |  |  | |  |  | |  | | |  | | |  | |  |  | |  | |
|  |  |  |  |  |  |  |  |  |  |  | |  | |  |  | | |  |  | | | |  | |  | | |  | |  |  | |  |  | |  | | |  | | |  | |  |  | |  | |
|  | 11.2.13 | | Mixaaxisha adii | | | |  | | |  | |  | |  |  | | |  |  | | | |  | |  | | |  | |  |  | |  |  | |  | | |  | | |  | |  |  | |  | |
|  |  |  |  |  |  |  |  |  |  |  | |  | |  |  | | |  |  | | | |  | |  | | |  | |  |  | |  |  | |  | | |  | | |  | |  |  | |  | |
|  | 11.2.14 | | Godarree | | | |  | | |  | |  | |  |  | | |  |  | | | |  | |  | | |  | |  |  | |  |  | |  | | |  | | |  | |  |  | |  | |
|  |  |  |  |  |  |  |  |  |  |  | |  | |  |  | | |  |  | | | |  | |  | | |  | |  |  | |  |  | |  | | |  | | |  | |  |  | |  | |
|  | 11.2.15 | | Atara makoodii | | | |  | | |  | |  | |  |  | | |  |  | | | |  | |  | | |  | |  |  | |  |  | |  | | |  | | |  | |  |  | |  | |
|  |  |  |  |  |  |  |  |  |  |  | |  | |  |  | | |  |  | | | |  | |  | | |  | |  |  | |  |  | |  | | |  | | |  | |  |  | |  | |
|  | 11.2.16 | | Shunkurtii [Garlic] | | | |  | | |  | |  | |  |  | | |  |  | | | |  | |  | | |  | |  |  | |  |  | |  | | |  | | |  | |  |  | |  | |
|  |  |  |  |  |  |  |  |  |  |  | |  | |  |  | | |  |  | | | |  | |  | | |  | |  |  | |  |  | |  | | |  | | |  | |  |  | |  | |
|  | 11.2.17 | | Kan biroo *ibsi______*  *(LROTH)* | | | |  | | |  | |  | |  |  | | |  |  | | | |  | |  | | |  | |  |  | |  |  | |  | | |  | | |  | |  |  | |  | |
|  |  |  |  |  |  |  |  |  |  |  | |  | |  |  | | |  |  | | | |  | |  | | |  | |  |  | |  |  | |  | | |  | | |  | |  |  | |  | |
| Legumes | 11.2.18 | | Baaqela | | | |  | | |  | |  | |  |  | | |  |  | | | |  | |  | | |  | |  |  | |  |  | |  | | |  | | |  | |  |  | |  | |
|  |  |  |  |  |  |  |  |  |  |  | |  | |  |  | | |  |  | | | |  | |  | | |  | |  |  | |  |  | |  | | |  | | |  | |  |  | |  | |
|  | 11.2.19 | | Boloqqee | | | |  | | |  | |  | |  |  | | |  |  | | | |  | |  | | |  | |  |  | |  |  | |  | | |  | | |  | |  |  | |  | |
|  |  |  |  |  |  |  |  |  |  |  | |  | |  |  | | |  |  | | | |  | |  | | |  | |  |  | |  |  | |  | | |  | | |  | |  |  | |  | |
|  | 11.2.20 | | Shunburaa[Chick pea | | | |  | | |  | |  | |  |  | | |  |  | | | |  | |  | | |  | |  |  | |  |  | |  | | |  | | |  | |  |  | |  | |
|  |  |  |  |  |  |  |  |  |  |  | |  | |  |  | | |  |  | | | |  | |  | | |  | |  |  | |  |  | |  | | |  | | |  | |  |  | |  | |
|  | 11.2.21 | | Atara | | | |  | | |  | |  | |  |  | | |  |  | | | |  | |  | | |  | |  |  | |  |  | |  | | |  | | |  | |  |  | |  | |
|  |  |  |  |  |  |  |  |  |  |  | |  | |  |  | | |  |  | | | |  | |  | | |  | |  |  | |  |  | |  | | |  | | |  | |  |  | |  | |
|  | 11.2.22 | | Missira | | | |  | | |  | |  | |  |  | | |  |  | | | |  | |  | | |  | |  |  | |  |  | |  | | |  | | |  | |  |  | |  | |
|  |  |  |  |  |  |  |  |  |  |  | |  | |  |  | | |  |  | | | |  | |  | | |  | |  |  | |  |  | |  | | |  | | |  | |  |  | |  | |
|  |  |  | | |  |  | | |  |  |  |  |  |  |  |  |  |  |  |  |  |  |  |  |  |  |  |  |  |  |  |  |  |  |  |  |  |  |  |  |  |  |  |  |  |  |  |  |
|  |  |  | | |  |  | | |  |  |  |  |  |  |  |  |  |  |  |  |  |  |  |  |  |  |  |  |  |  |  |  |  |  |  |  |  |  |  |  |  |  |  |  |  |  |  |  |
|  |  |  | | |  |  | | |  |  |  |  |  |  |  |  |  |  |  |  |  |  |  |  |  |  |  |  |  |  |  |  |  |  |  |  |  |  |  |  |  |  |  |  |  |  |  |  |
|  |  |  | | |  |  | | |  |  |  |  |  |  |  |  |  |  |  |  |  |  |  |  |  |  |  |  |  |  |  |  |  |  |  |  |  |  |  |  |  |  |  |  |  |  |  |  |
|  | 11.2.23 | | | Akurii Atara [Soya beans] | | | |  | | |  | |  | | |  |  | | |  | |  | |  | |  |  | |  | | |  | | |  | |  |  | |  |  | |  | | |  | |  |
|  |  |  |  |  |  |  |  |  | | |  | |  | | |  |  | | |  | |  | |  | |  |  | |  | | |  | | |  | |  |  | |  |  | |  | | |  | |  |
|  | 11.2.24 | | | Abishii suunqoo | | | |  | | |  | |  | | |  |  | | |  | |  | |  | |  |  | |  | | |  | | |  | |  |  | |  |  | |  | | |  | |  |
|  |  |  |  |  |  |  |  |  | | |  | |  | | |  |  | | |  | |  | |  | |  |  | |  | | |  | | |  | |  |  | |  |  | |  | | |  | |  |
|  | 11.2.25 | | | kanbiroo *ibsi_____*  *(LLOTH)* | | | |  | | |  | |  | | |  |  | | |  | |  | |  | |  |  | |  | | |  | | |  | |  |  | |  |  | |  | | |  | |  |
|  |  |  |  |  |  |  |  |  | | |  | |  | | |  |  | | |  | |  | |  | |  |  | |  | | |  | | |  | |  |  | |  |  | |  | | |  | |  |
| Midhaan gurgurtaaf oomishamu/cash crop | 11.2.26 | | | Buna | | | |  | | |  | |  | | |  |  | | |  | |  | |  | |  |  | |  | | |  | | |  | |  |  | |  |  | |  | | |  | |  |
|  |  |  |  |  |  |  |  |  | | |  | |  | | |  |  | | |  | |  | |  | |  |  | |  | | |  | | |  | |  |  | |  |  | |  | | |  | |  |
|  |  |  |  |  |  |  |  |  | | |  | |  | | |  |  | | |  | |  | |  | |  |  | |  | | |  | | |  | |  |  | |  |  | |  | | |  | |  |
|  | 11.2.27 | | | Caatii/jimaa | | | |  | | |  | |  | | |  |  | | |  | |  | |  | |  |  | |  | | |  | | |  | |  |  | |  |  | |  | | |  | |  |
|  |  |  |  |  |  |  |  |  | | |  | |  | | |  |  | | |  | |  | |  | |  |  | |  | | |  | | |  | |  |  | |  |  | |  | | |  | |  |
|  | 11.2.28 | | | Jirbii | | | |  | | |  | |  | | |  |  | | |  | |  | |  | |  |  | |  | | |  | | |  | |  |  | |  |  | |  | | |  | |  |
|  |  |  |  |  |  |  |  |  | | |  | |  | | |  |  | | |  | |  | |  | |  |  | |  | | |  | | |  | |  |  | |  |  | |  | | |  | |  |
|  | 11.2.29 | | | shankoora | | | |  | | |  | |  | | |  |  | | |  | |  | |  | |  |  | |  | | |  | | |  | |  |  | |  |  | |  | | |  | |  |
|  |  |  |  |  |  |  |  |  | | |  | |  | | |  |  | | |  | |  | |  | |  |  | |  | | |  | | |  | |  |  | |  |  | |  | | |  | |  |
|  | 11.2.30 | | | Qooccoo/enset | | | |  | | |  | |  | | |  |  | | |  | |  | |  | |  |  | |  | | |  | | |  | |  |  | |  |  | |  | | |  | |  |
|  |  |  |  |  |  |  |  |  | | |  | |  | | |  |  | | |  | |  | |  | |  |  | |  | | |  | | |  | |  |  | |  |  | |  | | |  | |  |
|  | 11.2.31 | | | Geeshoo | | | |  | | |  | |  | | |  |  | | |  | |  | |  | |  |  | |  | | |  | | |  | |  |  | |  |  | |  | | |  | |  |
|  |  |  |  |  |  |  |  |  | | |  | |  | | |  |  | | |  | |  | |  | |  |  | |  | | |  | | |  | |  |  | |  |  | |  | | |  | |  |
|  |  |  |  |  |  |  |  |  | | |  | |  | | |  |  | | |  | |  | |  | |  |  | |  | | |  | | |  | |  |  | |  |  | |  | | |  | |  |
|  | 11.2.37 | | | Tamboo | | | |  | | |  | |  | | |  |  | | |  | |  | |  | |  |  | |  | | |  | | |  | |  |  | |  |  | |  | | |  | |  |
|  |  |  |  |  |  |  |  |  | | |  | |  | | |  |  | | |  | |  | |  | |  |  | |  | | |  | | |  | |  |  | |  |  | |  | | |  | |  |
|  | 11.2.32 | | | kanbiroo *ibsi_____*  *(LCCOTH)* | | | |  | | |  | |  | | |  |  | | |  | |  | |  | |  |  | |  | | |  | | |  | |  |  | |  |  | |  | | |  | |  |
|  |  |  |  |  |  |  |  |  | | |  | |  | | |  |  | | |  | |  | |  | |  |  | |  | | |  | | |  | |  |  | |  |  | |  | | |  | |  |
| Kuduraa | 11.2.33 | | | Timaatima | | | |  | | |  | |  | | |  |  | | |  | |  | |  | |  |  | |  | | |  | | |  | |  |  | |  |  | |  | | |  | |  |
|  |  |  |  |  |  |  |  |  | | |  | |  |  |  |  |  | | |  | |  | |  | |  |  | |  | | |  | | |  | |  |  | |  |  | |  | | |  | |  |
|  | 11.2.34 | | | Barbaree | | | |  | | |  | |  |  |  |  |  | | |  | |  | |  | |  |  | |  | | |  | | |  | |  |  | |  |  | |  | | |  | |  |
|  |  |  |  |  |  |  |  |  | | |  | |  |  |  |  |  | | |  | |  | |  | |  |  | |  | | |  | | |  | |  |  | |  |  | |  | | |  | |  |
|  | 11.2.35 | | | Buqqee /dubbaa | | | |  | | |  | |  |  |  |  |  | | |  | |  | |  | |  |  | |  | | |  | | |  | |  |  | |  |  | |  | | |  | |  |
|  |  |  |  |  |  |  |  |  | | |  | |  |  |  |  |  | | |  | |  | |  | |  |  | |  | | |  | | |  | |  |  | |  |  | |  | | |  | |  |
|  | 11.2.36 | | | Kaarotii | | | |  | | |  | |  |  |  |  |  | | |  | |  | |  | |  |  | |  | | |  | | |  | |  |  | |  |  | |  | | |  | |  |
|  |  |  |  |  |  |  |  |  | | |  | |  |  |  |  |  | | |  | |  | |  | |  |  | |  | | |  | | |  | |  |  | |  |  | |  | | |  | |  |
|  | 11.2.37 | | | Shunkurtii | | | |  | | |  | |  |  |  |  |  | | |  | |  | |  | |  |  | |  | | |  | | |  | |  |  | |  |  | |  | | |  | |  |
|  |  |  |  |  |  |  |  |  | | |  | |  |  |  |  |  | | |  | |  | |  | |  |  | |  | | |  | | |  | |  |  | |  |  | |  | | |  | |  |
|  | 11.2.38 | | | Salaaxaa | | | |  | | |  | |  |  |  |  |  | | |  | |  | |  | |  |  | |  | | |  | | |  | |  |  | |  |  | |  | | |  | |  |
|  |  |  |  |  |  |  |  |  | | |  | |  | | |  |  | | |  | |  | |  | |  |  | |  | | |  | | |  | |  |  | |  |  | |  | | |  | |  |
|  | 11.2.39 | | | Raafuu daraaraa/embraangoo | | | |  | | |  | |  |  |  |  |  | | |  | |  | |  | |  |  | |  | | |  | | |  | |  |  | |  |  | |  | | |  | |  |
|  |  |  |  |  |  |  |  |  | | |  | |  |  |  |  |  | | |  | |  | |  | |  |  | |  | | |  | | |  | |  |  | |  |  | |  | | |  | |  |
|  | 11.2.40 | | | Raafuu/goommana | | | |  | | |  | |  |  |  |  |  | | |  | |  | |  | |  |  | |  | | |  | | |  | |  |  | |  |  | |  | | |  | |  |
|  |  |  |  |  |  |  |  |  | | |  | |  |  |  |  |  | | |  | |  | |  | |  |  | |  | | |  | | |  | |  |  | |  |  | |  | | |  | |  |
|  |  |  |  |  |  |  |  |  | | |  | |  |  |  |  |  | | |  | |  | |  | |  |  | |  | | |  | | |  | |  |  | |  |  | |  | | |  | |  |
|  | 11.2.41 | | | Raafuu gurraatti/abashaa | | | |  | | |  | |  |  |  |  |  | | |  | |  | |  | |  |  | |  | | |  | | |  | |  |  | |  |  | |  | | |  | |  |
|  |  |  |  |  |  |  |  |  | | |  | |  |  |  |  |  | | |  | |  | |  | |  |  | |  | | |  | | |  | |  |  | |  |  | |  | | |  | |  |
|  | 11.2.42 | | | Shifarraaw/Shiferaw | | | |  | | |  | |  |  |  |  |  | | |  | |  | |  | |  |  | |  | | |  | | |  | |  |  | |  |  | |  | | |  | |  |
|  |  |  |  |  |  |  |  |  | | |  | |  |  |  |  |  | | |  | |  | |  | |  |  | |  | | |  | | |  | |  |  | |  |  | |  | | |  | |  |
|  | 11.2.42 | | | Qoosxaa | | | |  | | |  | |  |  |  |  |  | | |  | |  | |  | |  |  | |  | | |  | | |  | |  |  | |  |  | |  | | |  | |  |
|  |  |  |  |  |  |  |  |  | | |  | |  |  |  |  |  | | |  | |  | |  | |  |  | |  | | |  | | |  | |  |  | |  |  | |  | | |  | |  |
|  | 11.2.43 | | | Kuduraalee baala gurraacha qaban kan biro o  Ibsi *_____ (LDGOTH)* | | | |  | | |  | |  |  |  |  |  | | |  | |  | |  | |  |  | |  | | |  | | |  | |  |  | |  |  | |  | | |  | |  |
|  |  |  |  |  |  |  |  |  | | |  | |  |  |  |  |  | | |  | |  | |  | |  |  | |  | | |  | | |  | |  |  | |  |  | |  | | |  | |  |
|  | 11.2.44 | | | Kuduraalee baala adii qaban kan biro o  Ibsi *_____ (LLGOTH)* | | | |  | | |  | |  |  |  |  |  | | |  | |  | |  | |  |  | |  | | |  | | |  | |  |  | |  |  | |  | | |  | |  |
|  |  |  |  |  |  |  |  |  | | |  | |  |  |  |  |  | | |  | |  | |  | |  |  | |  | | |  | | |  | |  |  | |  |  | |  | | |  | |  |
|  | 11.2.45 | | | Kuduraalee biroo *ibsi______*  *(LVOTH)* | | | |  | | |  | |  |  |  |  |  | | |  | |  | |  | |  |  | |  | | |  | | |  | |  |  | |  |  | |  | | |  | |  |
|  |  |  |  |  |  |  |  |  | | |  | |  |  |  |  |  | | |  | |  | |  | |  |  | |  | | |  | | |  | |  |  | |  |  | |  | | |  | |  |
| Fuduraalee | 11.2.46 | | | Maangoo | | | |  | | |  | |  | | |  |  | | |  | |  | |  | |  |  | |  | | |  | | |  | |  |  | |  |  | |  | | |  | |  |
|  |  |  |  |  |  |  |  |  | | |  | |  |  |  |  |  | | |  | |  | |  | |  |  | |  | | |  | | |  | |  |  | |  |  | |  | | |  | |  |
|  |  |  |  |  |  |  |  |  | | |  | |  |  |  |  |  | | |  | |  | |  | |  |  | |  | | |  | | |  | |  |  | |  |  | |  | | |  | |  |
|  | 11.2.47 | | | Abokaadoo | | | |  | | |  | |  |  |  |  |  | | |  | |  | |  | |  |  | |  | | |  | | |  | |  |  | |  |  | |  | | |  | |  |
|  |  |  |  |  |  |  |  |  | | |  | |  | | |  |  | | |  | |  | |  | |  |  | |  | | |  | | |  | |  |  | |  |  | |  | | |  | |  |
|  | 11.2.48 | | | Muuza | | | |  | | |  | |  |  |  |  |  | | |  | |  | |  | |  |  | |  | | |  | | |  | |  |  | |  |  | |  | | |  | |  |
|  |  |  |  |  |  |  |  |  | | |  | |  |  |  |  |  | | |  | |  | |  | |  |  | |  | | |  | | |  | |  |  | |  |  | |  | | |  | |  |
|  |  |  |  |  |  |  |  |  | | |  | |  |  |  |  |  | | |  | |  | |  | |  |  | |  | | |  | | |  | |  |  | |  |  | |  | | |  | |  |
|  | 11.2.49 | | | Anaanaasii/ Pineapple | | | |  | | |  | |  |  |  |  |  | | |  | |  | |  | |  |  | |  | | |  | | |  | |  |  | |  |  | |  | | |  | |  |
|  |  |  |  |  |  |  |  |  | | |  | |  |  |  |  |  | | |  | |  | |  | |  |  | |  | | |  | | |  | |  |  | |  |  | |  | | |  | |  |
|  | 11.2.50 | | | Burtukaana | | | |  | | |  | |  |  |  |  |  | | |  | |  | |  | |  |  | |  | | |  | | |  | |  |  | |  |  | |  | | |  | |  |
|  |  |  |  |  |  |  |  |  | | |  | |  |  |  |  |  | | |  | |  | |  | |  |  | |  | | |  | | |  | |  |  | |  |  | |  | | |  | |  |
|  | 11.2.51 | | | Appililii/ Apples | | | |  | | |  | |  |  |  |  |  | | |  | |  | |  | |  |  | |  | | |  | | |  | |  |  | |  |  | |  | | |  | |  |
|  |  |  |  |  |  |  |  |  | | |  | |  |  |  |  |  | | |  | |  | |  | |  |  | |  | | |  | | |  | |  |  | |  |  | |  | | |  | |  |
|  |  | | |  | | | |  | | |  | |  |  |  |  |  | | |  | |  | |  | |  |  | |  | | |  | | |  | |  |  | |  |  | |  | | |  | |  |
|  |  |  |  |  |  |  |  |  | | |  | |  |  |  |  |  | | |  | |  | |  | |  |  | |  | | |  | | |  | |  |  | |  |  | |  | | |  | |  |
|  | 11.2.53 | | | Paappaayyaa | | | |  | | |  | |  |  |  |  |  | | |  | |  | |  | |  |  | |  | | |  | | |  | |  |  | |  |  | |  | | |  | |  |
|  |  |  |  |  |  |  |  |  | | |  | |  |  |  |  |  | | |  | |  | |  | |  |  | |  | | |  | | |  | |  |  | |  |  | |  | | |  | |  |
|  | 11.2.54 | | | Loomii | | | |  | | |  | |  |  |  |  |  | | |  | |  | |  | |  |  | |  | | |  | | |  | |  |  | |  |  | |  | | |  | |  |
|  |  |  |  |  |  |  |  |  | | |  | |  |  |  |  |  | | |  | |  | |  | |  |  | |  | | |  | | |  | |  |  | |  |  | |  | | |  | |  |
|  | 11.2.55 | | | mandariina | | | |  | | |  | |  |  |  |  |  | | |  | |  | |  | |  |  | |  | | |  | | |  | |  |  | |  |  | |  | | |  | |  |
|  |  |  |  |  |  |  |  |  | | |  | |  |  |  |  |  | | |  | |  | |  | |  |  | |  | | |  | | |  | |  |  | |  |  | |  | | |  | |  |
|  | 11.2.56 | | | Roman | | | |  | | |  | |  |  |  |  |  | | |  | |  | |  | |  |  | |  | | |  | | |  | |  |  | |  |  | |  | | |  | |  |
|  |  |  |  |  |  |  |  |  | | |  | |  |  |  |  |  | | |  | |  | |  | |  |  | |  | | |  | | |  | |  |  | |  |  | |  | | |  | |  |
|  | 11.2.57 | | | Tringoo | | | |  | | |  | |  |  |  |  |  | | |  | |  | |  | |  |  | |  | | |  | | |  | |  |  | |  |  | |  | | |  | |  |
|  |  |  |  |  |  |  |  |  | | |  | |  |  |  |  |  | | |  | |  | |  | |  |  | |  | | |  | | |  | |  |  | |  |  | |  | | |  | |  |
|  | 11.2.58 | | | Zayituna | | | |  | | |  | |  |  |  |  |  | | |  | |  | |  | |  |  | |  | | |  | | |  | |  |  | |  |  | |  | | |  | |  |
|  |  |  |  |  |  |  |  |  | | |  | |  |  |  |  |  | | |  | |  | |  | |  |  | |  | | |  | | |  | |  |  | |  |  | |  | | |  | |  |
|  | 11.2.59 | | | Gishxaa/Gishita | | | |  | | |  | |  |  |  |  |  | | |  | |  | |  | |  |  | |  | | |  | | |  | |  |  | |  |  | |  | | |  | |  |
|  |  |  |  |  |  |  |  |  | | |  | |  |  |  |  |  | | |  | |  | |  | |  |  | |  | | |  | | |  | |  |  | |  |  | |  | | |  | |  |
|  |  |  |  |  |  |  |  |  | | |  | |  |  |  |  |  | | |  | |  | |  | |  |  | |  | | |  | | |  | |  |  | |  |  | |  | | |  | |  |
|  | 11.2.60 | | | Fuduraalee biroo ibsi*_____*  *(LFOTH)* | | | |  | | |  | |  |  |  |  |  | | |  | |  | |  | |  |  | |  | | |  | | |  | |  |  | |  |  | |  | | |  | |  |
|  |  |  |  |  |  |  |  |  | | |  | |  |  |  |  |  | | |  | |  | |  | |  |  | |  | | |  | | |  | |  |  | |  |  | |  | | |  | |  |
| Midhaan zayitaa | 11.2.61 | | | Qoobboo/guuloo | | | |  | | |  | |  | | |  |  | | |  | |  | |  | |  |  | |  | | |  | | |  | |  |  | |  |  | |  | | |  | |  |
|  |  |  |  |  |  |  |  |  | | |  | |  | | |  |  | | |  | |  | |  | |  |  | |  | | |  | | |  | |  |  | |  |  | |  | | |  | |  |
|  | 11.2.62 | | | Talbaa | | | |  | | |  | |  | | |  |  | | |  | |  | |  | |  |  | |  | | |  | | |  | |  |  | |  |  | |  | | |  | |  |
|  |  |  |  |  |  |  |  |  | | |  | |  | | |  |  | | |  | |  | |  | |  |  | |  | | |  | | |  | |  |  | |  |  | |  | | |  | |  |
|  | 11.2.63 | | | Nuugii/ /neug | | | |  | | |  | |  | | |  |  | | |  | |  | |  | |  |  | |  | | |  | | |  | |  |  | |  |  | |  | | |  | |  |
|  |  |  |  |  |  |  |  |  | | |  | |  | | |  |  | | |  | |  | |  | |  |  | |  | | |  | | |  | |  |  | |  |  | |  | | |  | |  |
|  | 11.2.64 | | | Sanyii jirbii/Cotton Seed | | | |  | | |  | |  | | |  |  | | |  | |  | |  | |  |  | |  | | |  | | |  | |  |  | |  |  | |  | | |  | |  |
|  |  |  |  |  |  |  |  |  | | |  | |  | | |  |  | | |  | |  | |  | |  |  | |  | | |  | | |  | |  |  | |  |  | |  | | |  | |  |
|  | 11.2.65 | | | Lawzii/lewuz | | | |  | | |  | |  | | |  |  | | |  | |  | |  | |  |  | |  | | |  | | |  | |  |  | |  |  | |  | | |  | |  |
|  |  |  |  |  |  |  |  |  | | |  | |  | | |  |  | | |  | |  | |  | |  |  | |  | | |  | | |  | |  |  | |  |  | |  | | |  | |  |
|  | 11.2.66 | | | Saliixa/Rapeseed | | | |  | | |  | |  | | |  |  | | |  | |  | |  | |  |  | |  | | |  | | |  | |  |  | |  |  | |  | | |  | |  |
|  |  |  |  |  |  |  |  |  | | |  | |  | | |  |  | | |  | |  | |  | |  |  | |  | | |  | | |  | |  |  | |  |  | |  | | |  | |  |
|  | 11.2.67 | | | Bordaa /Suufii | | | |  | | |  | |  | | |  |  | | |  | |  | |  | |  |  | |  | | |  | | |  | |  |  | |  |  | |  | | |  | |  |
|  |  |  |  |  |  |  |  |  | | |  | |  | | |  |  | | |  | |  | |  | |  |  | |  | | |  | | |  | |  |  | |  |  | |  | | |  | |  |
|  | 11.2.68 | | | Salixii/Sesame | | | |  | | |  | |  | | |  |  | | |  | |  | |  | |  |  | |  | | |  | | |  | |  |  | |  |  | |  | | |  | |  |
|  |  |  |  |  |  |  |  |  | | |  | |  | | |  |  | | |  | |  | |  | |  |  | |  | | |  | | |  | |  |  | |  |  | |  | | |  | |  |
|  | 11.2.69 | | | Midhaan zayita kan biro  Ibsi ______  (LOSOTH) | | | |  | | |  | |  | | |  |  | | |  | |  | |  | |  |  | |  | | |  | | |  | |  |  | |  |  | |  | | |  | |  |
|  |  |  |  |  |  |  |  |  | | |  | |  | | |  |  | | |  | |  | |  | |  |  | |  | | |  | | |  | |  |  | |  |  | |  | | |  | |  |
| **Mi’eessitoota** | 11.2.70 | | | **Zinjibil/ganjabeeloo** | | | |  | | |  | |  | | |  |  | | |  | |  | |  | |  |  | |  | | |  | | |  | |  |  | |  |  | |  | | |  | |  |
|  |  |  |  | **Irdii** | | | |  | | |  | |  | | |  |  | | |  | |  | |  | |  |  | |  | | |  | | |  | |  |  | |  |  | |  | | |  | |  |
|  |  |  |  | **Kororimaa/ Cardamom** | | | |  | | |  | |  | | |  |  | | |  | |  | |  | |  |  | |  | | |  | | |  | |  |  | |  |  | |  | | |  | |  |
|  |  |  |  | **Dinbilaala/Coriander** | | | |  | | |  | |  | | |  |  | | |  | |  | |  | |  |  | |  | | |  | | |  | |  |  | |  |  | |  | | |  | |  |
|  |  |  |  | **Kan biroo ibsi** | | | |  | | |  | |  | | |  |  | | |  | |  | |  | |  |  | |  | | |  | | |  | |  |  | |  |  | |  |  |  |  | |  |

## Kutaa 3: Baasii Midhaaniif Bahe

Maaloo mee Amajii bara 2013 hanga Mudde bara 2013tti waa’ee baasii hundaa kan maatiin keessan bittaa cicoleeomishaa midhaaaniitiif baaftan natti him.

Gabatee 11.3

|  | **Gosa Soyiraa/ciicata [Input type]** | **Amajii ’13-Mudde’13 kan fayyadamte(1=eeyyee,0=lakki) 98 hin bekku** | **Amajii ’13-Mudde’13 kan argate(1=eeyyee,0=lakki) 98 hin bekku** | **midhaan1** | **Gatii 1** | **Madda 1** | **Midhaan 2** | **Gatii 2** | **Madda 2** | **Midhaan 3** | **Gatii 3** | **Madda 3** | **Gatii walgala (qarshi)** |
| --- | --- | --- | --- | --- | --- | --- | --- | --- | --- | --- | --- | --- | --- |
| 11.3.1 | Sanyii –baratamaa | ESEED12U |  | ESEED12 | ESEED1 | ESEED1C | ESEED1S | ESEED2 | ESEED2C | ESEED2S | ESEED3 | ESEED3C |  |
|  | Sanyii- fooyya’aa | ESEEDI12U |  | ESEEDI12 | ESEEDI1 | ESEEDI1C | ESEEDI1S | ESEEDI2 | ESEEDI2C | ESEEDI2S | ESEEDI3 | ESEEDI3C |  |
|  | Sanyii diqaalaa | ESEEDH12U |  | ESEEDH12 | ESEEDH1 | ESEEDH1C | ESEEDH1S | ESEEDH2 | ESEEDH2C | ESEEDH2S | ESEEDH3 | ESEEDH3C |  |
| 11.3.2 | Facaasuu /dhaabuu -baramaa | EPLANT12U |  | EPLANT12 | EPLANT1 | EPLANT1C | EPLANT1S | EPLANT2 | EPLANT2C | EPLANT2S | EPLANT3 | EPLANT3C |  |
|  | Facaasuu /dhaabuu - fooyya’aa | EPLANTI12U |  | EPLANTI12 | EPLANTI1 | EPLANTI1C | EPLANTI1S | EPLANTI2 | EPLANTI2C | EPLANTI2S | EPLANTI3 | EPLANTI3C |  |
| 11.3.3 | Xaa’oo namni tolche/inorganic fertilizer | EINORG12U |  | EINORG12 | EINORG1 | EINORG1C | EINORG1S | EINORG2 | EINORG2C | EINORG2S | EINORG3 | EINORG3C |  |
| 11.3.4 | Xaa’oo baala tortore /foliar fertilizer | EFOL12U |  | EFOL12 | EFOL1 | EFOL1C | EFOL1S | EFOL2 | EFOL2C | EFOL2S | EFOL3 | EFOL3C |  |
| 11.3.5 | Dhoqqee /Dikee horii | EMANURE12U |  | EMANURE12 | EMANURE1 | EMANURE1C | EMANURE1S | EMANURE2 | EMANURE2C | EMANURE2S | EMANURE3 | EMANURE3C |  |
| 11.3.6 | Bishaan jallisii | EIRRIG12U |  | EIRRIG12 | EIRRIG1 | EIRRIG1C | EIRRIG1S | EIRRIG2 | EIRRIG2C | EIRRIG2S | EIRRIG3 | EIRRIG3C |  |
| 11.3.7 | Kompoostii | EMULCH12U |  | EMULCH12 | EMULCH1 | EMULCH1C | EMULCH1S | EMULCH2 | EMULCH2C | EMULCH2S | EMULCH3 | EMULCH3C |  |
| 11.3.8 | Midhaan kunuunsuu fi gabbisuuf dhaabamu/cover crop | ECOVER12U |  | ECOVER12 | ECOVER1 | ECOVER1C | ECOVER1S | ECOVER2 | ECOVER2C | ECOVER2S | ECOVER3 | ECOVER3C |  |
| 11.3.9 | Qoricha Farra ilbiisotaa | EPEST12U |  | EPEST12 | EPEST1 | EPEST1C | EPEST1S | EPEST2 | EPEST2C | EPEST2S | EPEST3 | EPEST3C |  |
| 11.3.10 | Qoricha Farra biqiltuu | EHERB12U |  | EHERB12 | EHERB1 | EHERB1C | EHERB1S | EHERB2 | EHERB2C | EHERB2S | EHERB3 | EHERB3C |  |
| 11.3.11 | Lafa kireeffatame | ERENT12U |  | ERENT12 | ERENT1 | ERENT1 C | ERENT1S | ERENT2 | ERENT2C | ERENT2S | ERENT3 | ERENT3C |  |
| 11.3.12 | Kuusaa/magaazana-sorage | ESTORAGE12U |  | ESTORAGE12 | ESTORAGE1 | ESTORAGE1C | ESTORAGE1S | ESTORAGE2 | ESTORAGE2C | ESTORAGE2S | ESTORAGE3 | ESTORAGE3C |  |
| 11.3.13 | hojii hojjachuuf/gaggeessuf | EPROCESS12U |  | EPROCESS12 | EPROCESS1 | EPROCESS1C | EPROCESS1S | EPROCESS2 | EPROCESS2C | EPROCESS2S | EPROCESS3 | EPROCESS3C |  |
| 11.3.14 | Baasii geejiba oomisha – iddoo oomishaatirraa gara manaatti | ETPORT12U |  | ETPORT12 | ETPORT1 | ETPORT1C | ETPORT1S | ETPORT2 | ETPORT2C | ETPORT2S | ETPORT3 | ETPORT3C |  |
| 11.3.15 | Baasii geejibaoomisha – mana irraa gara iddoo warshaatti/gabaatti | ETPMARK12U |  | ETPMARK12 | ETPMARK1 | ETPMARK1C | ETPMARK1S | ETPMARK2 | ETPMARK2C | ETPMARK2S | ETPMARK3 | ETPMARK3C |  |
| 11.3.16 | Waan horiin harkifamu [gaarii,oboloo] kireeffachuu | EANIM12U |  | EANIM12 | EANIM1 | EANIM1C | EANIM1S | EANIM2 | EANIM2C | EANIM2S | EANIM3 | EANIM3C |  |
| 11.3.17 | Meeshaalee qonnaaf ta’an kireeffachuu (fakkenyaaf., tiraaktara , boorsaa dugdaa, meeshaa qoricha iittiin biifan) | EIMP12U |  | EIMP12 | EIMP1 | EIMP1C | EIMP1S | EIMP2 | EIMP2C | EIMP2S | EIMP3 | EIMP3C |  |
| 11.3.18 | Kanbiroo (ibsi_____) EOTHSPE | EOTH12U |  | EOTH12 | EOTH1 | EOTH1C | EOTH1S | EOTH2 | EOTH2C | EOTH2S | EOTH3 | EOTH3C |  |

Koodii: 1. Jiddu gala qorannoo qonnaa 2. Waajjira qonnaa 3. Ollaa/hiriyyaa 4. Univarsiitii 5. Suuqii 6. Kan ergifatame

## Kutaa 4: Tilmaama Horii

Maaloo mee horii amma maatiin keessan qaban natti himi. *[Inni /isheen horii kan gurgurtuu yoo ta’e gatii loonii gabaa ammaa tilmaamuuf akka danda’an gaafii deebisaa gargaari]*

| 11.4.1 | Waggaa tokko darbe keessatti Maatiin keessan horii bitanii/qabaatanii/guguranii beekuu horsiisa kannisaatis dabalatee? | 1. Eeyyee 0. Lakki 98. hinbeeku | HHLIVESTOCK |
| --- | --- | --- | --- |

Gabatee 11.4: Abumma horii yeroo amma

|  | **Gosa horii** | **Baay’ina**  (yoo ‘’0’’ ta’e , gara gabatee mirgaatti darbi) | **Gatii dimshaashaa (Birr)** | **Kan eenyu akka ta’e (koodii asii gadii ilaali)** |
| --- | --- | --- | --- | --- |
| 11.4.2 | Qotiyyoo abashaa | ELBULLQ | ELBULLV | ELBULLO |
| 11.4.3 | Qotiyyoo faranjii | EBULLQ | EBULLV | EBULLO |
| 11.4.4 | Korma diqaalaa | ECBULLQ | ECBULLV | ECBULLO |
| 11.4.5 | Sa’a abashaa | ELCOWQ | ELCOWV | ELCOWO |
| 11.4.6 | Sa’a faranjii | ECOWQ | ECOWV | ECOWO |
| 11.4.7 | Goromsa abashaa | ELHEIQ | ELHEIV | ELHEIO |
| 11.4.8 | Goromsa farajii | EHEIQ | EHEIV | EHEIO |
| 11.4.9 | Sa’a diqaalaa | ECCOWQ | ECCOWV | ECCOWO |
| 11.4.10 | Jabbii abashaa | ELCALVQ | ELCALVV | ELCALVO |
| 11.4.11 | Jabbii faranjii | ECALVQ | ECALVV | ECALVO |
| 11.4.12 | Jabbii diqaalaa | ECCALVQ | ECCALVV | ECCALVO |
| 11.4.13 | Gaala | ECAMELQ | ECAMELV | ECAMELO |
| 11.4.14 | Hoolaa | ESHEEPQ | ESHEEPV | ESHEEPO |
| 11.4.15 | Re’ee abashaa | ELGOATQ | ELGOATV | ELGOATO |
| 11.4.16 | Re’ee faranjii | EGOATQ | EGOATV | EGOATO |
| 11.4.17 | Harree | EDONKQ | EDONKV | EDONKO |
| 11.4.18 | Farda | EHORSQ | EHORSV | EHORSO |
| 11.4.19 | Gaangee | EMULEQ | EMULEV | EMULEO |
| 11.4.20 | Lukkuu abashaa | ELCHICKQ | ELCHICKV | ELCHICKO |
| 11.4.21 | Lukkuu fooyya’aa | EIMCHICKQ | EIMCHICKV | EIMCHICKO |
| 11.4.22 | Horisiisa sanyii sinbiraa biroo [Gogorrii, sololiyaa,daakkiyyee kkf biraa (ibsi___) EOTHPOU | EOTHPOUQ | EOTHPOUV | EOTHPOUO |
| 11.4.23 | Horsiisa kanniisaa aadaa | EBEEQ | EBEEV | EBEEO |
| 11.4.24 | Horsiisa kanniisaa hammayyaa | EMBEEQ | EMBEEV | EMBEEO |
| 11.4.25 | Beelladoota biraa (sibsi___) EOTHLIV | EOTHLIVQ | EOTHLIVV | EOTHLIVO |

Kan eenyu akka ta’e: 1. Duraa bu’a mattii 2.abbaa manaa 3. Nitti duraa 4. Hadha5. Nitt lamafa 6. Kan fira biroo (ijoollee dabalatee) 7. Hin ilalatuu

## Kutaa 5: Horsiisa loonii

Gabate 11.5 Maaloo mee waa’ee horsiisa horii maatii keetii kan Amajii bara 2013 – Mudee 2013 jiru nutti himi.

|  | **Gosa** | **Oomisha** | **Safara** | **Baay’na oomishame/horsiifame safartuun** | **Baay’ina sooratame safartuun** | **Baay’ina gurgurame safartuun** | **Baay’inna kan namaaf kenname /bade/hatame safartuun** | **Baay’inna kan du’e** | **Gatiin tokkoon isaa (Birr)** |
| --- | --- | --- | --- | --- | --- | --- | --- | --- | --- |
| 11.5.1 | Horii | Loon lubbuun jiran |  | LCANIB |  | LCANIS | LCANIL |  | LCANID |
| 11.5.2 |  | Foon | LCBEEFU | LCBEEFP | LCBEEFC | LCBEEFS | LCBEEFL |  |  |
| 11.5.3 |  | Aanan | LCMILKU | LCMILKP | LCMILKC | LCMILKS | LCMILKL |  |  |
| 11.5.4 |  | Dhadhaa | LCBUTU | LCBUTP | LCBUTC | LCBUTS | LCBUTL |  |  |
| 11.5.5 |  | Baaduu | LCCHEEU | LCCHEEP | LCCHEEC | LCCHEES | LCCHEEL |  |  |
| 11.5.6 |  | Gogaa | LCHIDESU | LCHIDESP | LCHIDESC | LCHIDESS | LCHIDESL |  |  |
| 11.5.7 |  | Dhoqqee loonii/kobota | LCCATTU | LCCATTP | LCCATTC | LCCATTS | LCCATTL |  |  |
| 11.5.8 |  | Itittuu(ittoo sa’aa) | LCYOGU | LCYOGP | LCYOGC | LCYOGS | LCYOGL |  |  |
| 11.5.9 |  | kanbiroo(ibsi_______) LCOTHSPE | LCOTHU | LCOTHP | LCOTHC | LCOTHS | LCOTHL |  |  |
| 11.5.10 | Gaala | Gaala lubbuun jiru | LCAMU | LCAMP | LCAMC | LCAMS | LCAML |  | LCAMD |
| 11.5.11 | Hoolaa | Hoolota lubbuun jiran |  | LSANIB |  | LSANIS | LSANIL |  | LSANID |
| 11.5.12 |  | Foon | LSMEATU | LSMEATP | LSMEATC | LSMEATS | LSMEATL |  |  |
| 11.5.13 |  | Dabbasaa/suufii hoolaa | LSWOOLU | LSWOOLP | LSWOOLC | LSWOOLS | LSWOOLL |  |  |
| 11.5.14 |  | Kal’ee/erbee | LSKINU | LSKINP | LSKINC | LSKINS | LSKINL |  |  |
| 11.5.15 |  | Shishii/kokorii hoolaa | LSMANU | LSMANP | LSMANC | LSMANS | LSMANL |  |  |
| 11.5.16 | Re’ee | Re’oota lubbuun jiran |  | LGANIB |  | LGANIS | LGANIL |  | LGANID |
| 11.5.17 |  | Foon | LGMEATU | LGMEATP | LGMEATC | LGMEATS | LGMEATL |  |  |
| 11.5.18 |  | Aanan | LGMILKU | LGMILKP | LGMILKC | LGMILKS | LGMILKL |  |  |
| 11.5.19 |  | Kal’ee/erbee | LGSKINU | LGSKINP | LGSKINC | LGSKINS | LGSKINL |  |  |
| 11.5.20 |  | Shishii/ kokorii re’ee | LGMANU | LGMANP | LGMANC | LGMANS | LGMANL |  |  |
| 11.5.21 | Horsiisa lukkuu | Kan lubbuun jiran |  | LPOBIRDB | LPOBIRDC | LPOBIRDS | LPOBIRDL |  | LPOBIRDD |
| 11.5.22 |  | Hanqaaquu | LPOEGGSU | LPOEGGSP | LPOEGGSC | LPOEGGSS | LPOEGGSL |  | LPOEGGSD |
| 11.5.23 |  | Kosii lukkuu/Bird manure | LPOMANU | LPOMANP | LPOMANC | LPOMANS | LPOMANL |  |  |
| 11.5.24 | Harree | Harroota lubbuun jiran |  | LDANIB |  | LDANIS | LDANIL |  | LDANILD |
| 11.5.25 | Gaangee | Gaangee lubbuun jiran |  | LMANIB |  | LMANIS | LMANIL |  | LMANID |
| 11.5.26 | Farda | faradoo lubbuun jiran |  | LHANIB |  | LHANIS | LHANIL |  | LHANID |
|  |  | Faandoo Harree ,Gaangee and Farda |  |  |  |  |  |  |  |
| 11.5.27 | Hormaata kanniisaa | Damma | LHONEYU | LHONEYP | LHONEYC | LHONEYS | LHONEYL |  | LHONEYV |
| 11.5.28 |  | Gagaa |  |  |  |  |  |  |  |
| 11.5.29 |  | gurmuu kanniisaa/ colony |  |  |  |  |  |  |  |
|  |  | Safafi dama/Propolis |  |  |  |  |  |  |  |
| 11.5.30 | Kanbiroo (ibsi _______) LOTHSPE | | LOTHU | LOTHP | LOTHC | LOTHS | LOTHL |  | LOTHV |

## Kutaa 6: Baasii Horsiisa Horii

Maaloo mee Amajii bara 2013 hanga Mudee bara 2013tti waa’ee baasii hundaa hojiilee horsisaa horii tiif baaftannatti himaa.

Gabatee 11.6

|  | **Hojii** | **Ramaddii/gita sooyiraa/baasii** | **Gosa sooyiraa/gaasii-/input type** | **Deddeebi waggaatti/frequency** | **Baay’inna yeroo tokkoon tokko isaa itti fayyadamte** | **Safara** | **Gatii safartu tokkoo (Birr)** | **Gatii dimshaashaa (Birr)** |
| --- | --- | --- | --- | --- | --- | --- | --- | --- |
| 11.6.1 | Nyaata horiitiif /nyaachisuuf | Mannoo/ nyaata horiif Bitame | Galabaa/citaa /ibbiqii | LFSTRAWF | LFSTRAWQ | LFSTRAWU | LFSTRAWV | LFSTRAWS |
| 11.6.2 |  |  | Dirqoosha/okaya | LFHAYF | LFHAYQ | LFHAYU | LFHAYV | LFHAYS |
| 11.6.3 |  |  | Midhaan nyaata horiif oolu | LFGRAINF | LFGRAINQ | LFGRAINU | LFGRAINV | LFGRAINS |
| 11.6.4 |  |  | Huuba/ hansaraa boqqoolloo ) | LFCONCF | LFCONCQ | LFCONCU | LFCONCV | LFCONCS |
| 11.6.5 |  |  | Soogidda/ashaboo | LFSALTF | LFSALTQ | LFSALTU | LFSALTV | LFSALTS |
| 11.6.6 |  |  | Haftee midhaanii /crop residue/ | LFCRRESF | LFCRRESQ | LFCRRESU | LFCRRESV | LFCRRESS |
| 11.6.7 |  |  | Marga | LFGRASSF | LFGRASSQ | LFGRASSU | LFGRASSV | LFGRASSS |
| 11.6.8 |  |  | Kanbiroo (ibsi ) LFOTHSPE | LFOTHF | LFOTHQ | LFOTHU | LFOTHV | LFOTHS |
|  | Horsiisa kanniisaa | Gaagura | Kanniisa | LBEEHF | LBEEHQ | LBEEHU | LBEEHV | LBEEHS |
|  |  |  | Gaagura bareechuuf | LFRAMEF | LFRAMEQ | LFRAMEU | LFRAMEV | LFRAMS |
|  |  |  | Mootii addaan baasaa | LQUEXF | LQUEXQ | LQUEXU | LQUEXV | LQUEXS |
|  |  | Nyaata | sukkaara | LSUGF | LSUGQ | LSUGU | LSUGV | LSUGS |
|  |  |  | Nyaata Abaaboo | LFLOWSF | LFLOWSQ | LFLOWSU | LFLOWSV | LFLOWSS |
|  |  | To’annoo ilbiisaa | Wanta ilbiisota ittiin too’atan | LPESTF | LPESTQ | LPESTU | LPESTV | LPESTS |
|  |  | Oomisha dammaa | Meeshaa hiddaa ofirraa iitisan | LPROCTF | LPROCTQ | LPROCTU | LPROCTV | LPROCTS |
|  |  |  | Aara aarsuu | LSMOKEF | LSMOKEQ | LSMOKEU | LSMOKEV | LSMOKES |
|  |  |  | Meeshaa ittiin qulqulleesinu | LSMOKEF | LSMOKEQ | LSMOKEU | LSMOKEV | LSMOKES |
|  |  | Kanniisa | Jiisaa/Icii/colony | LCOLONF | LCOLONQ | LCOLONU | LCOLONV | LCOLONS |
| 11.6.9 | Eegumsa Fayyaa horii | Qoricha | Raammoo garaa qulqulleessuu | LHMED1F | LHMED1Q | LHMED1U | LHMED1V | LHMED1S |
| 11.6.10 |  |  | LHMED2T | LHMED2F | LHMED2Q | LHMED2U | LHMED2V | LHMED2S |
| 11.6.11 |  |  | LHMED3T | LHMED3F | LHMED3Q | LHMED3U | LHMED3V | LHMED3S |
| 11.6.12 |  | Talaallii | LVACC1T | LVACC1F | LVACC1Q | LVACC1U | LVACC1V | LVACC1S |
| 11.6.13 |  |  | LVACC2T | LVACC2F | LVACC2Q | LVACC2U | LVACC2V | LVACC2S |
| 11.6.14 |  | Tajaajila beelladaa | Qoricha Farra silmii | LVET1F | LVET1Q | LVET1U | LVET1V | LVET1S |
| 11.6.15 |  |  | LVET2T | LVET2F | LVET2Q | LVET2U | LVET2V | LVET2S |
| 11.6.16 |  |  | LVET3T | LVET3F | LVET3Q | LVET3U | LVET3V | LVET3S |
| 11.6.17 | Marfeen dorrobsiisuu/Artificial insemination |  |  | LSEM1F | LSEM1Q | LSEM1U | LSEM1V | LSEM1S |
| 11.6.18 |  |  |  | LSEM2F | LSEM2Q | LSEM2U | LSEM2V | LSEM2S |
| 11.6.19 | Tajaajila kormaa/bull service |  |  | LBULLF | LBULLQ | LBULLU | LBULLV | LBULLS |
| 11.6.20 | Bittaa horii/loonii |  | LPL1T | LPL1F | LPL1Q | LPL1U | LPL1V | LPL1S |
| 11.6.21 |  |  | LPL2T | LPL2F | LPL2Q | LPL2U | LPL2V | LPL2S |
| 11.6.22 |  |  | LPL3T | LPL3F | LPL3Q | LPL3U | LPL3V | LPL3S |
| 11.6.23 |  |  | LPL4T | LPL4F | LPL4Q | LPL4U | LPL4V | LPL4S |
| 11.6.24 | Mooraa ijaaruu/”beret” |  | LBARNT | LBARNF | LBARNQ | LBARNU | LBARNV | LBARNS |
| 11.6.25 | Mooraa qulqulessu |  | LMAINT1T | LMAINT1F | LMAINT1Q | LMAINT1U | LMAINT1V | LMAINT1S |
| 11.6.26 |  |  | LMAINT2T | LMAINT2F | LMAINT2Q | LMAINT2U | LMAINT2V | LMAINT2S |
| 11.6.27 | Geejjiba horiifi oomisha horiirraa argamu |  | LTRANSPT | LTRANSPF | LTRANSPQ | LTRANSPU | LTRANSPV | LTRANSPS |
| 11.6.28 | Oomisha aananiif annanirraa argaman omishuu |  | LPROCEST | LPROCESF | LPROCESQ | LPROCESU | LPROCESV | LPROCESS |
| 11.6.29 | Kanbiroo (ibsi ) LOTH1SPE |  | LOTH1T | LOTH1F | LOTH1Q | LOTH1U | LOTH1V | LOTH1S |
| 11.6.30 | Kanbiroo (ibsi ) LOTH2SPE |  | LOTH2T | LOTH2F | LOTH2Q | LOTH2U | LOTH2V | LOTH2S |

## Kutaa 7: Gabaa Oomisha Midhaanii Fi Loonii

Amma iddoo maatiin kee oomisha midhaanii ykn loonii ji’oota 12n jechuun Amajii bara 2013 hanga Mudee bara 2013 keessatti yeroo baay’ee itti gurguran sigaafachuun barbaada.

Gabatee 11.7

|  | **Gaafii** | **1= iddoo qonnaarratti/oorruumarraatti**  **2=gabaa dhihootti**  **3=gabaa fagoo /guddaarratti**  **4=karaa garee qoteebultootaattiin**  **5. kanbiroo**  **6. hojiirra hinoolu**  **7=hinbeekkamu** | **Variable name** |
| --- | --- | --- | --- |
| 11.7.1 | Yeroo baay’ee midhaan nyaataa kee eessatti gugurta? |  | PFOOD |
| 11.7.2 | Yeroo baay’ee midhaan gurgurtaaf oomishaman [buna, caatii, baala shaayii, jirbii kkf] eessatti gurgurta? |  | PCROP |
| 11.7.3 | Yeroo baay’ee loon/lukkuu eessatti gugurta?? |  | PANIMAL |
| 11.7.4 | Yeroo baay’ee aanan eessatti gugurta?? |  | PMILK |
| 11.7.5 | Yeroo baay’ee foon horii eessatti gugurta?? |  | PMEAT |
| 11.7.6 | Yeroo baay’ee oomisha /bu’aalee horii nyaataaf hin oolle eessatti gugurta?? |  | PNONFOOD |

##

## Kutaa 8: Teeknoolojii Qonnaatii Fi itti fayyadama isaa

Ati ykn miseensi maatii kee kan biroo ji’oota 12n jechuun Amajjii 2013 hanga mudde 2013 keessatti gochoota kanneen raawwataniiru ykn meeshaalee kanneenitti fayyadamaniiru?

Gabate 11.8

|  | **Gochoota** | **Maatii /miseensi kamiyyuu fayyadame**(1=eeyyee 0=lakki) 98= hin bekuu | **Var. Name** |
| --- | --- | --- | --- |
| 11.8.1 | Oorruu/lafa osoo hinqotiin waqtii tokko ykn sanaa ol bulchuu/akka turu gochuu |  | TFALLOW |
| 11.8.2 | Lafa qulqulleessuuf ibiddaan gubuu |  | TBURN |
| 11.8.3 | Midhaan tarreen/sararaan dhaabuu/facaasuu |  | TROWS |
| 11.8.4 | Midhaan gosa adda addaa walkeessa dhaabuun/facaasuun fayyadamuu |  | TINTERCROP |
| 11.8.5 | Lafa tokkorratti Midhaan jijjiiruun facaasuu |  | TCROPROTA |
| 11.8.6 | Midhaan /sanyii filatamaa/fooyya’aa fayyadamuu |  | TIMPROSEED |
| 11.8.7 | Midhaan oongee dandamatu fayyadamuu |  | TDROUGHTC |
| 11.8.8 | Midhaan aadaa hintaane fayyadamuu |  | TNONTRADCROP |
| 11.8.9 | Horii hinfooyyofne kunuusaa/eegaa /horsiisaa ture |  | TNONIMPSTOCK |
| 11.8.10 | Horii fooyya’aa kunuusaa/eegaa /horsiisaa ture |  | TIMPSTOCK |
| 11.8.11 | Cakii/makotkochaa/ qabata dheeraa qabu fayyadame |  | TLONGHOE |
| 11.8.11 | Gaarii harkaan dhiibamu fayyadamuu |  | TWBARROW |
| 11.8.12 | Meeshaa qonnaa biro fayyadame (akaafaa, kkf) |  | TANOTHER |
|  |  |  | TANOTHER |
| 11.8.14 | Xaa’oo namni tolche fayyadame |  | TINORGAN |
| 11.8.15 | Xaa’oo uumamaa fayyadame |  | TORGANIC |
| 11.8.16 | Keemikaalota qonnaa fayyadame (fakkeenyaaf., farra ilbiisotaa, farra aramaa ) |  | TAGROCHEMFI |
| 11.8.17 | Keemikaalota qonnaa kuusaaf/gootaraa fayyadamu/ergamidhaal galee booda (fakenyaf-.farra ilbiisotaa) |  | TAGROCHEMST |
| 11.8.18 | Karaa aadaatiin ilbiisota too’achuu (fakenyaf.daaraa, fincaan, barbarree) |  | TLOCALPEST |
| 11.8.19 | Karaa qindaayeen ilbiisota too’achuu/too’annaa ilbiisotaa qindaa’e fayyadame |  | TPESTMAG |
|  |  |  | TSOILINT |
| 11.8.21 | too’annaa eegusa biyyoo qindaa’e fayyadame/soil fertility |  |  |
| 11.8.22 | Jallisii fayyadame |  | TIRRIGAT |
| 11.8.23 | Kan Horiin harkifamuun qote |  | TANPLOW |
| 11.8.24 | Kan horiin harkifamuun arame aramamuun |  | TANWEED |
| 11.8.25 | Qonna mekaanaayizdii fayyadame /meeshaa jabanaatiin qote/tiraaktaraan/ |  | TMECPLOW |
| 11.8.26 | Meeshaa jabanaatiin haame[kombaaynarii] |  | TMECHARV |
| 11.8.27 | Horii ykn lukkuu talaalchise |  | TVACPOUTRY |
| 11.8.28 | Nyaata loonii ykn lukkuu fooyya’aan fayyadame (kan bitame/manatti qophaa’e) |  | TIMPFEED |
| 11.8.29 | Horsiisa qurxummii [acquaculture] |  | TAQUACUL |
| 11.8.30 | Tooftaalee gogsuu fooyya’e fayyadamuu (fakenya- afata, sharaa, madardariyaa, lafa simintoo) |  | TIMPDRY |
| 11.8.31 | Mala kuusuu kan fooyya’e (fakenya dogoogoo/gumbii, bidiruu nyaata loonii itti kuusan/gandaa, qooxii nyaata horii irra kaahan ) |  | TIMPSTORA |
| 11.8.32 | Teeknoolojii fooyya’aa qindeessuuf fayyadamuu (fakenyaf., ahiiduu/mawqaat) |  | TIMPPROC |
| 11.8.33 | Gatii Dabalata oomisha qonna kamiyyuurratti[midhaan ykn loon |  | TADDVAL |
| 11.8.34 | Mala geejjibaa fooyya’aa bishaan,qoraan, oomisha midhaan ykn loon fe’uuf fayyadame (fakenyaaf baayskilii, motorsaayikilii, gaarii harkaan dhiibamu) |  | TIMPTRANS |
| 11.8.35 | Sirna Gabaa fooyya’e fayyadame (gareedhaan guguruu ykn oorruurratti osoo hintaane gabaatti gurguruu dabalatee ) |  | TIMPMARKT |
| 11.8.36 | Mala hammayya kanniisa horsiisuu |  | TIMPBEEHV |
| 11.8.37 | Nyaata kan akka sukkaaraa kanniisa nyaachisuu |  | TIMPFEEDB |
| 11.8.38. | Kanniisa horsiisuuf ilbiisota too’achuu |  | TIMPFEEDB |
| 11.8.39. | Kan biroo(ibsi___________) TOTHSPE |  | TOTH |

## Kutaa 9: Humna Namaa/Nama Hojiilee Qonnaa hundaaf Qaxarame

Gabatee 11.9

| 11.9.1 | Ji’oota 12n darban dhumaa keessatti, maatiin kee hojjataa/tuu oomisha qonnaa ykn horsiisa looniitiif qaxaranii turanii ? | 1=eeyyee  0=lakki 98= hinbeeku  Yoo lakki jette, kutaa itti aanutti darbi | LABOUR |
| --- | --- | --- | --- |

Eeyyee yoo jette maaloo humana namaa hunda maatiin keessan yeroo sanaatti itti fayyadame natti himi .

|  | | **Hojii** | **Hojii kanaffi humnaa mattii ittiofayadamtee?** (1=eeyyee , 0=lakki 98= hinbekuu)   - - - 1. **eyy** | **Yoo eyyee ta’e humnaa nama meqaa (guyya walaka ni laka’amma)** | **Daboo fayadamte turtee** | **Yoo eyyee ta’e humnaa nama meqaa (guyya walaka ni laka’amma)** | **Hojii kanneeniif nama qaxartanii ?** (1=eeyyee , 0=lakki) | **Yoo eyyee ta’e humnaa nama meqaa (guyya walaka ni laka’amma)** | **Eeyyee yoo jette, gatii dimshaashaa (Birr)** | |
| --- | --- | --- | --- | --- | --- | --- | --- | --- | --- | --- |
|  | **Hojiilee midhaaniin walqabatan** | | | | | | | | |  |
| 11.9.2 | | Facaasuun/dhaabuun duratti lafa qopheessuu (d, first ploughinaggala ciruu abalatee , dkkf) |  |  |  |  |  | LCPREPH | LCPREPV | |
| 11.9.3 | | Dhaabuu /facaasuu |  |  |  |  |  | LCPLANTH | LCPLANTV | |
| 11.9.4 | | Aramuu |  |  |  |  |  | LCWEEDH | LCWEEDV | |
| 11.8.5 | | Haamuu |  |  |  |  |  | LCHARVH | LCHARVV | |
| 11.9.6 | | Bishaan obaasuu/dikee irra facaasuu /keemikaalota qonnaa fayyadamuu |  |  |  |  |  | LCIRRIH | LCIRRIV | |
| 11.9.7 | | Kan biroo(ibsi_____) LCROTHSPE |  |  |  |  |  | LCROTHH | LCROTHV | |
| **Hojiilee loonin/horsiisa looniitin walqabatan** | | | | | | | | | |  |
| 11.9.8 | | Loon Marga dheechisuu |  |  |  |  |  | LLGRAZEH | LLGRAZEV | |
| 11.9.9 | | Loon bishaan obaasuu |  |  |  |  |  | LLWATERH | LLWATERV | |
| 11.9.10 | | Itti biifuu/spraying/Dipping |  |  |  |  |  | LLSPRAYH | LLSPRAYV | |
| 11.9.11 | | Aanan elmuufi omishuu |  |  |  |  |  | LLMILKH | LLMILKV | |
|  | | Horsisa kanisaa |  |  |  |  |  |  |  | |
| 11.9.12 | | Kan biroo (ibsi_____) LLOTHSPE |  |  |  |  |  | LLOTHH | LLOTHV | |

# Mujulii 12 – Galii fi Baasii

## Kutaa 1: Madda Galii Maatii Kan Biroo

*Amma, maaloo maddoota biroo Kan maatiin kee adoolessa 2012-waxabajjii 2013 galii irraa argatan natti himi.*

*Maaloo galii miseensoota maatii keetii hundaa itti dabalii natti himi.*

Table 12.1

|  | **Gosa galii argatan** | **Galii hojii kanarraa argatame? (1=eeyyee , 0=lakki)** (lakki yoo jette, gara gabatee darbi) | **Galii kana argachuuf baasii bahe?** (1=eeyyee 0=lakki) | **Yoo eeyyee ta’e, baasii bahe eere/himi** | **Yoo eeyyee ta’e , baasii dimshaashaa (Birr)** | **Bifa galii argatte 1=*maallaqaan 2= kan maallaqaan hintaane*** | **Baay’ina (e.g. lakk. jooniyyaa, xaasaa)** | **safartuu (yoo maallaqaan ta’uu baate)** | **Gatii tokkoo (Birr)** | **Gatii dimshaashaa (Birr)** | **Galii dhumaa (Birr)** |
| --- | --- | --- | --- | --- | --- | --- | --- | --- | --- | --- | --- |
| 12.1.1 | Humna namaa hojii qonna birootiif oole (midhaan ykn loon) | MAGI | MAGC | MAGT | MAGCV | MAGF | MAGA | MAGU | MAGUV | MAGTV | MAGTVC |
| 12.1.2 | Nama hojii qonnaan alatti qaxarame – akka tasaa, yeroodhaaf, ykn sirnaan/mindaa (gargaaraa/tuu manaa , ijaarsa, kkf dabaltee .) | MNOAGI | MNOAGC | MNOAGT | MNOAGCV | MNOAGF | MNOAGA | MNOAGU | MNOAGUV | MNOAGTV | MNOAGTVC |
| 12.1.3 | Soorama/xuurataa | MPENSI | MPENSC | MPENST | MPENSCV | MPENSF | MPENSA | MPENSU | MPENSUV | MPENSTV | MPENSTVC |
| 12.1.4 | Gargaarsa mootummaarraa ,mitimootummaarraa /mootummoota gamtoomanii(nyaata, sanyii, ykn gargaarsa loonii) | MASSTI | MASSTC | MASSTT | MASSTCV | MASSTF | MASSTA | MASSTU | MASSTUV | MASSTTV | MASSTTVC |
| 12.1.5 | Galii nama biyya alaa irraa | MREMITI | MREMITC | MREMITT | MREMITCV | MREMITF | MREMITA | MREMITU | MREMITUV | MREMITTV | MREMITTVC |
| 12.1.6 | Gargaarsa fira dhihoo/hiriyyaa irraa | MASRELI | MASRELC | MASRELT | MASRELCV | MASRELF | MASRELA | MASRELU | MASRELUV | MASRELTV | MASRELTVC |
| 12.1.7 | Kennaa | MGIFTI | MGIFTC | MGIFTT | MGIFTCV | MGIFTF | MGIFTA | MGIFTU | MGIFTUV | MGIFTTV | MGIFTTVC |
| 12.1.8 | Kan kireeffame (lafa, horii, meeshaalee) | MRENTI | MRENTC | MRENTT | MRENTCV | MRENTF | MRENTA | MRENTU | MRENTUV | MRENTTV | MRENTTVC |
| 12.1.9 | Muka ibiddaaf ta’uu gurguruun | MWOODI | MWOODC | MWOODT | MWOODCV | MWOODF | MWOODA | MWOODU | MWOODUV | MWOODTV | MWOODTVC |
| 12.1.10 | Xarbii/muka gurguruun | MPOLEI | MPOLEC | MPOLET | MPOLECV | MPOLEF | MPOLEA | MPOLEU | MPOLEUV | MPOLETV | MPOLETVC |
| 12.1.11 | Kasala gurguruun, kobota gurguruun | MCHARCI | MCHARCC | MCHARCT | MCHARCCV | MCHARCF | MCHARCA | MCHARCU | MCHARCUV | MCHARCTV | MCHARCTVC |
| 12.1.12 | Hojii harkaan hojjataman gurguruun | MCRAFTI | MCRAFTC | MCRAFTT | MCRAFTCV | MCRAFTF | MCRAFTA | MCRAFTU | MCRAFTUV | MCRAFTTV | MCRAFTTVC |
| 12.1.13 | Nyaata/dhugaatii qophaa’aa gurguruu/ mana nyaataatti gurguruun | MFOODI | MFOODC | MFOODT | MFOODCV | MFOODF | MFOODA | MFOODU | MFOODUV | MFOODTV | MFOODTVC |
| 12.1.14 | Dhugaatii gurguruu-kan aadaa dabalatee | MBREWI | MBREWC | MBREWT | MBREWCV | MBREWF | MBREWA | MBREWU | MBREWUV | MBREWTV | MBREWTVC |
| 12.1.15 | Shaqataa gurguruu | MSHOPI | MSHOPC | MSHOPT | MSHOPCV | MSHOPF | MSHOPA | MSHOPU | MSHOPUV | MSHOPTV | MSHOPTVC |
| 12.1.16 | Geejjiba | MBODAI | MBODAC | MBODAT | MBODACV | MBODAF | MBODAA | MBODAU | MBODAUV | MBODATV | MBODATVC |
| 12.1.17 | Gurgurtaa dirqoosha/haftee midhaanii /kompoostii /marga /mannoo | MHAYI | MHAYC | MHAYT | MHAYCV | MHAYF | MHAYA | MHAYU | MHAYUV | MHAYTV | MHAYTVC |
| 12.1.18 | Kobota gurguruu | MMANI | MMANC | MMANT | MMANCV | MMANF | MMANA | MMANU | MMANUV | MMANTV | MMANTVC |
| 12.1.19 | Foon gurguruun | MGAMEI | MGAMEC | MGAMET | MGAMECV | MGAMEF | MGAMEA | MGAMEU | MGAMEUV | MGAMETV | MGAMETVC |
| 12.1.20 | lawzii/fuduraa gurguruun | MWILDI | MWILDC | MWILDT | MWILDCV | MWILDF | MWILDA | MWILDU | MWILDUV | MWILDTV | MWILDTVC |
| 12.1.21 | Albuuda baasuun (soogidda, warqee, cirracha, suphee, kkf) | MMINEI | MMINEC | MMINET | MMINECV | MMINEF | MMINEA | MMINEU | MMINEUV | MMINETV | MMINETVC |
| 12.1.22 | Dhakaa bocuun/qotuun baasuu | MSTONEI | MSTONEC | MSTONET | MSTONECV | MSTONEF | MSTONEA | MSTONEU | MSTONEUV | MSTONETV | MSTONETVC |
| 12.1.23 | Xuubii hojjachuun | MBRICKI | MBRICKC | MBRICKT | MBRICKCV | MBRICKF | MBRICKA | MBRICKU | MBRICKUV | MBRICKTV | MBRICKTVC |
| 12.1.24 | Maashinii daakuu | MGMILLI | MGMILLC | MGMILLT | MGMILLCV | MGMILLF | MGMILLA | MGMILLU | MGMILLUV | MGMILLTV | MGMILLTVC |
| 12.1.25 | Daldala xixiqqoo biroo | MPETTYI | MPETTYC | MPETTYT | MPETTYCV | MPETTYF | MPETTYA | MPETTYU | MPETTYUV | MPETTYTV | MPETTYTVC |
| 12.1.26 | Othekan biroor (ibsi_____) MOTH1SPE | MOTH1I | MOTH1C | MOTH1T | MOTH1CV | MOTH1F | MOTH1A | MOTH1U | MOTH1UV | MOTH1TV | MOTH1TVC |
| 12.1.27 | kanbiroo (ibsi_____) MOTH2SPE | MOTH2I | MOTH2C | MOTH2T | MOTH2CV | MOTH2F | MOTH2A | MOTH2U | MOTH2UV | MOTH2TV | MOTH2TVC |

**Kutaa 4: Baasii nyaataa**

Gabatee12.4

|  | Ji’oota 12n darban dhumaa keessatti, maatiin kee bittaa ykn kafaltii kamiyyuu haata’u raawwatee turee [meeshaalee]?  eeyyee…1  lakki …2 ►gara meeshaa itti aanutti 98= hinbekuu ►gara meeshaa itti aanutti | | Waliigalatti maatiin kee qarshii kee meeqa kafalan?  [BIRR] |
| --- | --- | --- | --- |
| 1 | uffata/kophee/uffata dhiiraa | HECLOTHM | HECLOTHMB |
| 2 | uffata/kophee/uffata dubartootaaf | HECLOTHW | HECLOTHWB |
| 3 | uffata/kophee/uffata ijoollee dhiiraatiif | HECLOTHB | HECLOTHBB |
| 4 | uffata/kophee/uffata ijoollee dubaraatiif | HECLOTHG | HECLOTHGB |
| 5 | Meeshaalee kushinaa (okkotee ittiin bilcheessan, kkf.) | HEKITCH | HEKITCHB |
| 6 | Linens (ansoolaai, fooxaa ,awusaa/baanaa) | HELINEN | HELINENB |
| 7 | Meeshaalee manaa/Furniture | HEFURN | HEFURNB |
| 8 | Ampuulii/Xomboora/chibboo-Lamp/torch | HELAMP | HELAMPB |
| 9 | Baasii ayyaanaaf oolu/Ceremonial expenses | HECEREM | HECEREMB |
| 10 | Gumaacha Iddir tiif | HEIDDIR | HEIDDIRB |
| 11 | Kennaa bataskaanaaf/masgiidaaf | HECHURCH | HECHURCHB |
| 12 | Kafaltii mana baruumsaatiif | HESCHO | HESCHOB |
| 13 | Gatii/Kafaltii eegumsa fayyaatiif | HEHELYR | HEHELYRB |
